# A Genetic and Chemical Perspective on Symbiotic Recruitment of Cyanobacteria of the Genus *Nostoc* into the Host Plant *Blasia pusilla* L.

**DOI:** 10.3389/fmicb.2016.01693

**Published:** 2016-11-01

**Authors:** Anton Liaimer, John B. Jensen, Elke Dittmann

**Affiliations:** ^1^Department of Arctic and Marine Biology, Faculty of Biosciences, Fisheries and Economics, UiT-The Arctic University of NorwayTromsø, Norway; ^2^Department of Microbiology, Institute for Biochemistry and Biology, University of PotsdamPotsdam, Germany

**Keywords:** Cyanobacteria, secondary metabolites, symbiosis, *Blasia*, *Nostoc*, allelopathy

## Abstract

Liverwort *Blasia pusilla L.* recruits soil nitrogen-fixing cyanobacteria of genus *Nostoc* as symbiotic partners. In this work we compared *Nostoc* community composition inside the plants and in the soil around them from two distant locations in Northern Norway. STRR fingerprinting and 16S rDNA phylogeny reconstruction showed a remarkable local diversity among isolates assigned to several *Nostoc* clades. An extensive web of negative allelopathic interactions was recorded at an agricultural site, but not at the undisturbed natural site. The cell extracts of the cyanobacteria did not show antimicrobial activities, but four isolates were shown to be cytotoxic to human cells. The secondary metabolite profiles of the isolates were mapped by MALDI-TOF MS, and the most prominent ions were further analyzed by Q-TOF for MS/MS aided identification. Symbiotic isolates produced a great variety of small peptide-like substances, most of which lack any record in the databases. Among identified compounds we found microcystin and nodularin variants toxic to eukaryotic cells. Microcystin producing chemotypes were dominating as symbiotic recruits but not in the free-living community. In addition, we were able to identify several novel aeruginosins and banyaside-like compounds, as well as nostocyclopeptides and nosperin.

## Introduction

Multicellular nitrogen-fixing cyanobacteria of the genus *Nostoc* are a common component of terrestrial microbial communities in a wide range of habitats, including subpolar and hot arid zones. They are characterised by a versatile physiology and display one of the most complex life cycles among the bacteria. Nitrogen fixation in these microorganisms is confined to specialized cells, heterocysts, which appear in semi-regular pattern between chains of vegetative cells, where the photosynthesis takes place. In addition, the life cycle of *Nostoc* includes resting cells, akinetes, and motile multicellular filaments hormogonia, the dispersal units. Heterocystous cyanobacteria, primarily from the genus *Nostoc* are also engaged in a number of symbiotic interactions with plants and fungi (Meeks et al., 2002; Adams and Duggan, 2008). In such interactions, motile hormogonia serve as the infection units, whose differentiation is triggered by the host at initial stages of the infection process. The cyanobionts are housed either outside cells, in specialized cavities in the talli, as in liverworts and hornworts, or in roots as in cycads, or intracellular in symbiotic organs as in the angiosperms *Gunnera* sp. (Johansson and Bergman, 1992) or fungi of the genus *Geosyphon* (Kluge and Kape, 1992; Kluge, 1994). In these symbioses, both the hosts and the cyanobacterial partners are rather promiscuous, when the plant partners accept a wide range of *Nostoc* and a *Nostoc* strains isolated from one plant species is capable of infection phylogenetically distant hosts (Johansson and Bergman, 1994). The symbiotically competent *Nostoc* isolates were found along the entire *Nostoc* lineage of cyanobacteria (Papaefthimiou et al., 2008a) and do not show any patterns suggesting host specialization. The only exception is water-fern *Azolla-Nostoc* symbiosis, where the association is obligate and species specific for both and the partners are inseparable through the entire life cycle of the host (Papaefthimiou et al., 2008b; Zheng et al., 2009). The liverworts, hornworts and *Gunnera* produce the symbiotic structure before infection, and motile *Nostoc* filaments, hormogonia, enter these structures’ slime pores (Duckett et al., 1977; Rodgers and Stewart, 1977). Upon internalization, the heterocyst formation is usually up-regulated in symbiotic associations from about 4-10% in free-living cyanobacteria, to 25-60% when in symbiosis, although it seems likely that at least some of the extra heterocysts are non-functional (Meeks and Elhai, 2002). So far, no benefit was revealed for cyanobacteria to enter symbiotic interactions. Rather, formation of excess heterocysts diminishes propagation potential of eventually released cyanobionts.

Heterocystous cyanobacteria, including *Nostoc* are known as a rich source of biologically active secondary metabolites (Dembitsky and Rezanka, 2005; Rezanka and Dembitsky, 2006). Several compounds were used as drug leads, of which tumor inhibitors cryptophycins reached to the stage II clinical trials (Field et al., 2014). Analysis of the genome of *N. punctiforme* PCC73102 alone showed presence of 11 gene clusters possibly involved in non-ribosomal peptide, polyketide, and hybrid peptide-polyketide metabolites, most of which are still unknown (Liaimer et al., 2011). Further, over ten cryptic bacteriocin gene clusters with yet unexplored potential were predicted from the genomic sequence (Wang et al., 2011). Our recent findings show that at least some of these products, a cryptic polyketide compound and nostopeptolides are involved in the life cycle regulation and transition from motile stage to vegetative growth (Liaimer et al., 2011, 2015). Plant partners have a great impact on the production of secondary metabolites, by both down-regulating biosynthesis of metabolites observed in free-living state, and by inducing production of a great number unknown products (Liaimer et al., 2015). Therefore, genus *Nostoc*, with its complex life cycle and diverse interspecies interactions, represents a unique model for uncovering biological roles of secondary metabolites and discovery of novel compounds.

In their natural habitats, cyanobacteria will find themselves competing for resources with other organisms sharing similar environmental requirements, which include other cyanobacteria, microalgae and even higher plants. To date, only a very limited number of the cyanobacterial allelochemicals has been identified and mainly from freshwater cyanobacteria (Leflaive and Ten-Hage, 2007; Leão et al., 2009). These include cyanobacterin, a chlorinated γ-lactone produced by *Scytonema hofmanni* that inhibits other cyanobacteria and green microalgae (Mason et al., 1982); fisherellin A, an eynediyne-containing photosystem II inhibitor produced by *Fisherella muscicola* (Hagmann and Jüttner, 1996); the hapalindoles, small metabolites that have been isolated from *Hapalosiphon* (Moore et al., 1984; Thanh Doan et al., 2000) that show inhibitory effects against several microorganisms and the nostocyclamides, relatively small cyclic peptides produced by *Nostoc* sp. that inhibit cyanobacteria and microalgae (Todorova et al., 1995; Jüttner et al., 2001). The allelochemicals play important roles in phytoplankton succession, bloom formation and competition; several studies have investigated environmental factors that may modulate allelopathic events. The production of allelochemicals are controlled by the presence of competitors (Kearns and Hunter, 2000; Vardi et al., 2002; Jang et al., 2007), bacteria that degrade allelochemical compounds (Hulot and Huisman, 2004), light intensity, temperature, nutrient levels and pH (De Nobel et al., 1998; Ray and Bagchi, 2001; de Figueiredo et al., 2006).

**FIGURE 1:**
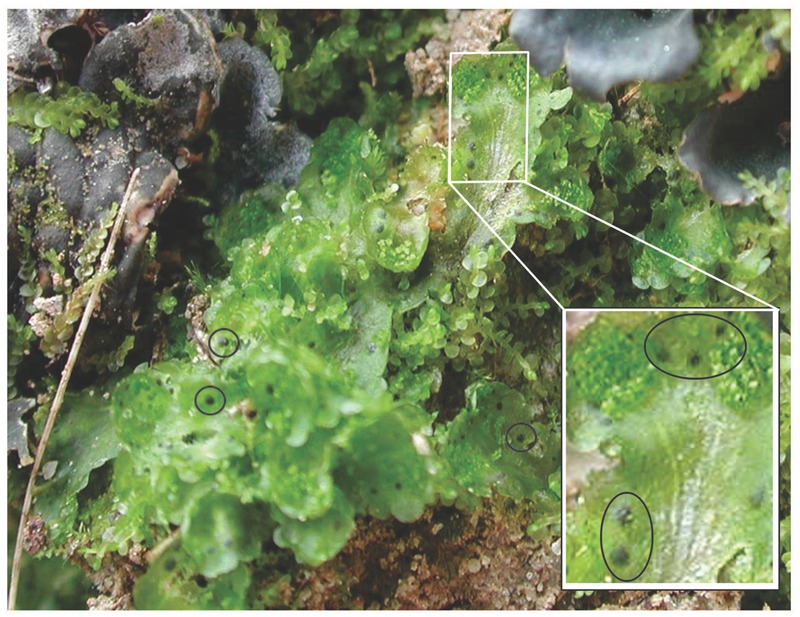
***Blasia pusilla* L. in its natural habitat.** Symbiotic cavities, auricles, housing *Nostoc* are encircled. Cyanolichens *Peltigera* sp. shares the same environment with *B. pusilla*.

In this study, we investigated symbiotic isolates obtained from *Blasia pusilla* L. naturally occurring in the various habitats in Troms region, Northern Norway. In these endophytic associations, the *Nostoc* colonies are housed in special cavities located on the ventral surface of the gametophyte (**Figure 1**). At the first steps we sorted the isolates by STRR-genome fingerprinting, followed by phylogeny reconstruction. The main goal of this work was, however, the chemical diversity in the *Nostoc* community inside the plant and in the soil around it, with an emphasis on small peptides based on whole cell and growth medium MALDI-TOF MS chemical typing. In addition, the fragmentation patterns of most prominent peaks were analyzed for the identification of peptides, when possible. Thus, we have aligned the genetic and chemical profiles among symbiotically competent *Nostoc*. We also tested the interstrain interactions by allelopathy assays in order to find possible explanations for differences between symbiotic and free-living communities. Having in mind the applied aspects of cyanobacterial secondary metabolites, we tested the isolates’ ability to inhibit different human cell lines, including two types of human cancer cells.

## Materials and Methods

### Isolation and Cultivation of Cyanobacteria

The plant material was collected on the territory of a plant school on Kvaløya island (69,64° N 18,73°E) near Tromsø and at an inland location near Skibotn field station (69,28°N 20,50°E), Northern Norway. The distance between the two sampling sites is approximately 81 km. Isolation of nitrogen-fixing cyanobacteria from soil and plants symbiotic organs was carried out as described earlier (West and Adams, 1997). The soil isolates were picked from the serial dilution plates. In order to suppress growth of algae and fungi the medium was supplemented with 20 mg/L cycloheximide for the first steps of isolation. All isolates were cultivated at room temperature in liquid or solidified BG11_0_ medium (Stanier et al., 1971) under continuous illumination with light intensity of 60 μmol/sec^∗^m^2^.

### Genotyping, Sequencing

The cyanobacterial samples were pipetted onto Whatman FTA classic cards (Sigma-Aldrich) and treated according to manufacturer’s instructions thereafter. STRR fingerprinting using 1, 2 mm FTA paper circles was performed according to Rasmussen and Svenning (1998). The first two letters in isolate label stand for location, KV-for Kvaløya, and SK for Skibotn, third letter J or S are random letters separating plant individuals, and F stands for free-living isolates from soil. The numbers stand for the order in which the isolate was picked from a given sample. Amplification of 16S rDNA and sequencing were done as described in Elvebakk et al. (2008). Sequences of 16S rDNA were deposited in the database of National Center for Biotechnology Information, NCBI, and assigned accession numbers EU022706 to EU022742 (Supplementary Table S1).

### Alignment and Phylogenetic Analysis

Twenty-five 16S rRNA gene sequence of the genotypes defined in this study and 45 additional cyanobacterial 16S rRNA gene sequences available in the NCBI database (Supplementary Table S1) were used in the phylogeny reconstruction. The total data set of 16S sequences was aligned (Supplementary Figure S5) with the computer program Bioedit (Hall, 1999), producing sequence alignment which correspond to the *Escherichia coli* sequence region between positions 26 to 1522 (Neefs et al., 1990). The MEGA6 software program was used making phylogenetic analysis (Tamura et al., 2007). The tree was constructed based on 16S rRNA gene sequences by using the neighbor-joining method (NJ) algorithm with bootstrap values calculating from 1000 iterations. The Kimura two-parameter model was used to estimate evolutionary distance among the species (Kimura, 1980). Three non-heterocystous cyanobacterial 16S rRNA gene sequences (from *Arthrospira platensis, Lyngbya* sp. PCC 7419, *Oscillatoria* sp.) were used as outgroup.

The software program DOTUR (Schloss and Handelsman, 2005) was used to define operational taxonomic units (OTUs) at a threshold of 98% sequence similarity on the distance matrix obtained with DNAdist from the Phylip 3.66 package (Felsenstein, 1993).

### Bioactivity Assays

Allelopathy assays were carried out as follows: a freshly plated lawn of indicator strain on solid BG11_0_ agar (1,2 % agar) was overlaid by a thin layer of semisolid (0,7%) medium, inoculation loop sized agar blocks with tester strains were placed on the top agar. Petri dishes were kept under standard cultivation conditions until visible results, in most cases 2-3 weeks.

Cyanobacterial extracts for cell viability assays and antibacterial activity testing were obtained from cyanobacteria grown in 1 l medium under standard conditions. The cyanobacteria were collected by centrifugation at 6000 × *g*. The pellets were re-suspended in 50 ml methanol:water and sonicated with Sonifier250 (Branson); 3 cycles of 3 min each, 40% duty cycle, output control 3. The cell debris was removed by centrifugation. The extracts were dried under vacuum, washed out by 2 ml 50% methanol, and brought to dryness in 1,5 ml Eppendorf tubes. The resulted powder was dissolved to a concentration 50 mg/ml. The effect of cyanobacterial extracts on human cell viability was tested against two adherent cancer cell lines: human melanoma A2058 (American Type Culture Collection, ATCC CRL-11147) and human colon carcinoma HT29 (ATCC HTB-38). In addition, adherent, non-malignant lung fibroblasts MRC5 (ATCC CCL-171) were used as toxicity control. Initially a selection of extracts was tested against human melanoma A2058 with the crude extract concentration of 500 μg/mL. Four positively tested extracts were tried further against human melanoma A2058, human colon carcinoma HT29 and lung fibroblasts MRC5 with four concentrations ranging from 33 μg/mL to 250 μg/mL. Antibacterial activity was assayed against *E. coli* ATCC 25922, *Staphylococcus aureus* ATCC 25923, multi resistant *S. aureus* (MRSA) ATCC 33591 and *Enterococcus faecalis* ATCC 29212. The assays were performed at MabCent, University of Tromsø, Norway, following established procedures described in Hanssen et al. (2012) and Ingebrigtsen et al. (2016).

### Mass Spectrometry

Lyophylized samples of cell pellets and supernatants were dissolved in water:acetonitril:ethanol (1:1:1) and analyzed by MALDI-TOF on a MALDI micro MX^TM^ from Micromass Waters. A 10 mg/ml α-Cyano-4-hydroxycinnamic acid (CHCA) matrix in 49,5% acetonitril, 49,5% Etanol, and 10 μl 0,1% TFA was used. The instrument contained a nitrogen laser giving a 337 nm output. The ions were accelerated with a voltage of 20 kV. A delayed extraction of 500 ns and ion suppression up to 500 Da was used. The MS was used in the positive ion-detection and reflector mode. Data were collected using variable laser power, optimized for each sample. For tandem mass spectrometry samples containing peptides in water:acetonitril:ethanol (1:1:1) were added formic acid to 0.1%. The samples were analyzed in positive mode using a Q-TOF Ultima mass spectrometer (Micromass/Waters) with a nanospray source. Samples were injected with a flow of 0.5 μl/min. Mass spectra were acquired in continuum mode. The list of peptides of interest meant for the fragmentation analyses was manually defined in the equipment’s current settings. Collision energy for fragmentation was manually set for each peptide.

An outline over the workflow implemented in this study, covering steps from sample collection to bioactivity analyses is presented in Supplementary Figure S4.

## Results

### Isolation of Cyanobacteria, Genotyping, and Phylogeny Reconstruction

**FIGURE 2:**
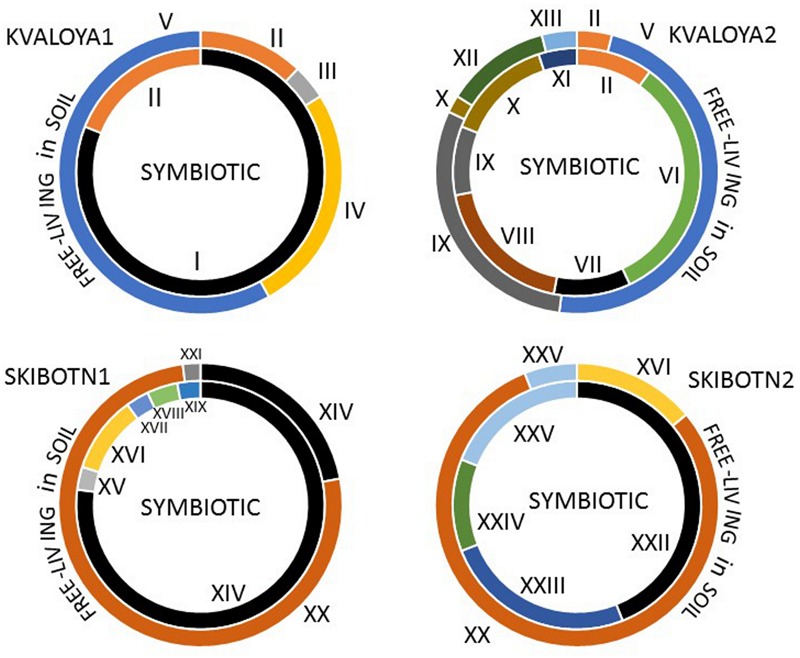
**Distribution and abundance of Nostocacean STRR-genotypes in four sampled *B. pusilla* plants from two locations in Northern Norway, Kvaloya island, and Skibotn valley.** The symbiotic genotypes are shown in inner circles, and the free-living genotypes found in soil around the plants are shown in outer circles. The genotypes producing microcystins are in black. The STRR- type fingerprints are shown in Supplementary Figure S1.

**FIGURE 3:**
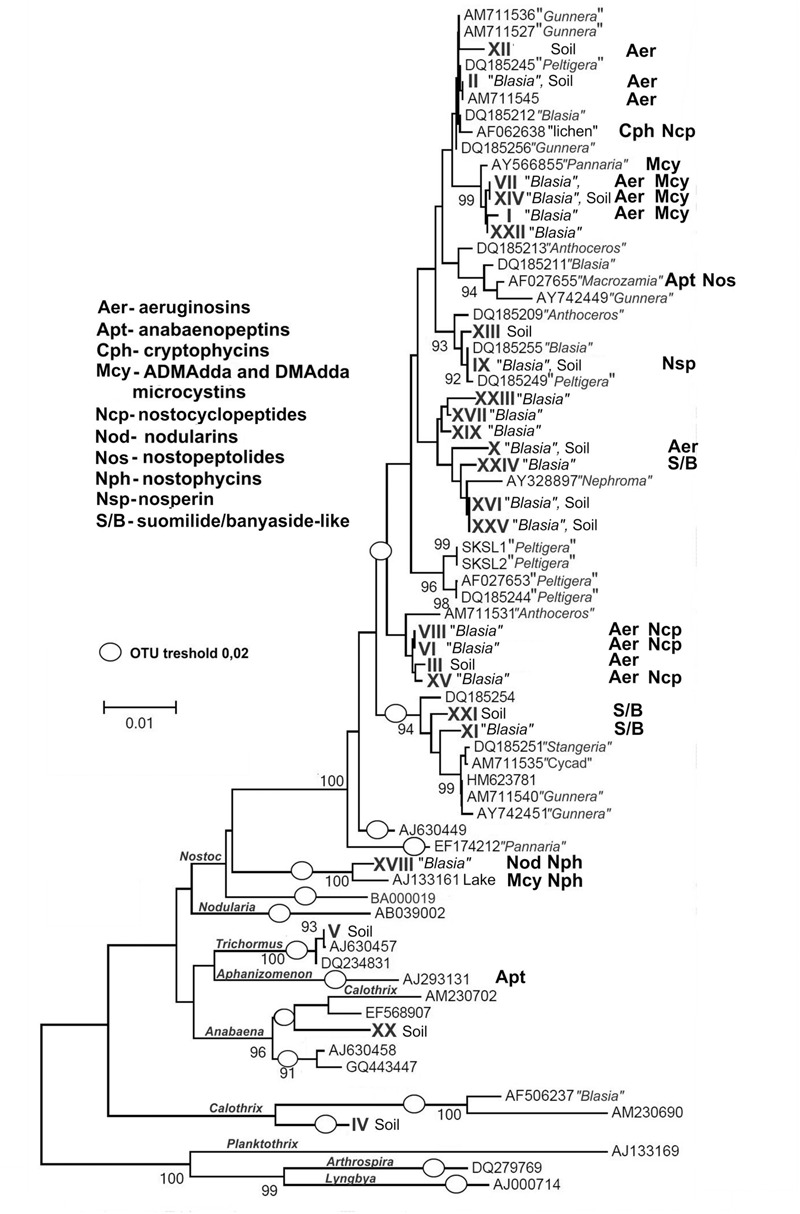
**Phylogenetic tree showing the relationship among the 25 cyanobacterial STRR-genotypes found in this study (roman numbers) and 45 additional cyanobacterial 16S rRNA gene sequences.** The tree was constructed based on 16S rRNA gene sequences by using the neighbor-joining method (NJ) algorithm with bootstrap values calculating from 1000 iterations. The Kimura two-parameter model was used to estimate evolutionary distance among the species (Kimura, 1980). Three non-heterocystous cyanobacterial 16S rRNA gene sequences (from *Arthrospira platensis, Lyngbya* sp. PCC 7419, *Oscillatoria* sp.) were used as outgroup. The scale bar corresponds to 0.01 nucleotide substitutions and (O) define operational taxonomic units (OTUs) at a threshold of 98% sequence similarity on the distance matrix. Distribution of secondary metabolites identified in this study or known from previous works are indicated next to the source strain.

The two selected collection sites were chosen for their difference in history. Kvaløya site is an agricultural land, while Skibotn site is a natural habitat. In addition, the sites are separated from each other by natural hinders like fjords and mountains. Most of the cyanobiont colonies extracted from individual symbiotic organs, auricles, gave rise to free-living colonies on solid BG11_0_ medium. All symbioses born colonies consisted of only one type of cyanobacteria, no mixed colonies were observed. The number of colonies to be taken for genotype sorting was limited to two hundred from each patch of *B. pusilla*, comprising in total approximately 800 isolates from four *B. pusilla* talli. All isolates were sorted into genotypes by STRR fingerprinting according to Rasmussen and Svenning (1998) (Supplementary Figure S1, **Figure 2**). In addition, the same amount of *Nostocaceae* colonies was collected from the soil to which talli were attached. Heterocystous cyanobacteria in soils were found at densities in a range from 0.5*10^4^ to 6*10^6^ CFU per cm^3^ soil. These values were not considered as accurate cell density estimates, since colonies in serial dilutions could rise from both single cells, as well as from multicellular filament or aggregates. The majority of the isolated cyanobacteria were successfully maintained in standard BG11_0_ medium in liquid with the a few exceptions. Isolates from Kvaloya samples were assigned to 13 STRR-genotypes, and isolates from Skibotn were assigned to 12 genotypes, respectively (Supplementary Figure S1, **Figure 2**). Individual genotypes were designated with consecutive roman numbers. Sequencing of several random representatives from each genotype showed no variability in 16S sequences within an STRR-genotype, except for symbiotic genotype XI from Kvaloya and soil isolate XXI, which were first treated as the same genotype but were separated later due to differences in 16S rDNA sequences specific for each collection site. There were two immediate observations from the genotype sorting. Firstly, there were notable differences in the community composition between two sample sets from the same collection site. Very few symbiotic isolates were common for both plants from the same location, while the soil shared only the dominant genotypes but not the minor components (**Figure 2**). The second observation was that the symbiotic sets of genotypes were only partially overlapping with the soil sets of genotypes for all sites and patches. Indeed, most of the isolates found in symbioses were not found outside the plant. In three samples, Kvaloya1, Kvaloya2, and Skibotn2, genotypes which were the most abundant *in planta*, i.e., I, VI, and XXII, respectively, were not found in soil communities, whilst the dominating genotypes in soil, IV and V from Kvaloya and XX from Skibotn where not found as symbionts. However, the latter three were not assigned to *Nostoc*, but belonged to *Calothrix, Trichormus*, and *Anabaena*, respectively, according to 16S rDNA sequences and the phylogenetic tree (Supplementary Table S1, **Figure 3**). The remainder of the isolates was placed within *Nostoc* as shown in **Figure 3**, contained within two closely related OTUs. These two clades combined correspond to the “cluster I *Nostoc*” defined by Papaefthimiou et al. (2008a) and contain *Nostoc* sequences originated from various terrestrial ecosystems and a variety of symbiotic associations, ranging from lichens to *Gunnera* species. The only outlier in the phylogeny presented here was genotype XVIII, forming a distinct clade with an aquatic microcystin producing *Nostoc* sp. 152 (Sivonen et al., 1990).

### MALDI-TOF MS Profiling and Metabolite Identification

Lyophilized cell pellets and filtered supernatants from all cultivable genotypes were subjected to MALDI-TOF MS analyses. In our preliminary studies we have noticed that lyophylization of the material prior to analyses aids to enhancement of peptide signals while the signals from chlorophyll and its derivatives stay low. We considered peaks with intensities over 5% relative to the highest observed. Our records include 254 individual peaks, representing 169 different compounds, in the range from 500 to 2000 Da. Several of the peaks were recognized as Na and K adducts commonly generated in ionization process in the MALDI-TOF MS analysis (Jemal et al., 1997; Mortier et al., 2004). The list of reproducibly occurring prominent peaks is presented in **Table 1**; the MALDI-TOF profiles are shown in Supplementary Figure S2. The most prominent ions were further subjected to fragmentation by Q-TOF MS. In this work we present fragmentation patterns (Supplementary Figure S3) of the compounds, which we were able to identify.

**Table 1:** Summary of the ions observed by MALDI-TOF screening of the cells and growth media of cyanobacterial isolates.

GENOTYPE Origin (example isolate)	Prominent peaks m/z H+(Na^+^, K^+^)	
	Cell-bound	Growth medium
I	(, 642/644)	615, 791
*Blasia*	859, Aer**875**, Aer**889** (911, 927), (949, 965)	Aer875, Aer**889** (911,927), 949, 967
(KVS1)	Mcy1009, Mcy**1023**, 1047, 1061, 1123, 1137	Mcy**1009**, Mcy**1023**, 1061, 1105

II		558, 600, 636, 688, 699, 715, 767, 782
*Blasia*, Soil	Aer843, Aer**865** (887, 903)	800, Aer843, Aer**865** (887, 903)
(KVS11)		(1042, 1058)

III	593, 605, (, 642), (765, 781)	515, 575, (765,781)
Soil	Aer**851** (873, 889), 953 (, 991), **969** (991,	813/815, **827/829, 843, 861**,
(KVSF4)	1007)	953 (975,991), **969 (991, 1007)**

V Soil (KVSF7)	555/557, 591, 607/609, 617, 628, **633, 1034**	580, 597 954, **980**, 1010, 1036

VI	593, 604/606 (626/628, **642/644**), Ncp757	615, Ncp (779), 791
*Blasia*	(779, **795**)	805 **(827, 843)**, 823 **(845, 861)**, Ae**r889**
(KVJ2)	Aer**889** (911, 927)	

VII	791	656
*Blasia*	847, Aer875, Aer**889** (911, 927), 981	(818, 834), **872**, 964, 992
(KVJ3)	Mcy1009, Mcy**1023**, 1061, 1099, 1156	Mcy**1009**, Mcy**1023**, 1061, 1083, 1105

VIII	593, (642/644), Ncp (, 795)	543, 599, Ncp757 **(779, 795)**
*Blasia* (KVJ4)	Aer**889** (911, **927**)	805 **(827, 843)**, (**845**, 861), **(856, 872)**, Aer889

IX *Blasia*, Soil (KVJ10)	504, **512/514, 526/528**, 542, 590, **605, (626/628)**	504, 532, Nsp564, **580, 610, 626**, 703, 719, 726, 989

X	536, 560, 642, 725	**575** (597,), **591** (**613**, 629),
*Blasia*, Soil	Aer**865** (887, 903)	Aer865 (887,903)
(KVJ18)	1193, 1272	1161, 1177, 1193

XI	593, **633, 642, 683, 721**	550, 650, (779, 795), **(813**, 829)
*Blasia*, Soil (KVJ20)	(,829), SB899, SB**927**, SB**997**, SB**1045** (1067, **1083**), SB1115 (1137, **1153**)	1029 (1041, **1057**), (1212, 1228), **(1268, 1284)**

XII	**531** (, 569), 615, **753**	**531 (553, 569), 631** (653, 669)
Soil (KVJF4)	**833**, Aer**851, (953, 969), (991, 1007)**	Aer851, 887, 901, **(953, 969)**, 969 **(991, 1007)**

XIV	604/606 (626/628, **642/644**)	547, 592, 791, 796 **(818, 834)**
*Blasia*, Soil	Aer875, Aer**889** (911, 927)	872, Aer**889** (911, 927), 950, 966, 980, 992
(SKS1)	Mcy1009, Mcy**1023**, 1061	Mcy1009, 1018, Mcy**1023** (1045,) 1061, 1105

XV	604/606 (626/628, 642/644), 781, 791	**791**
*Blasia* (SKS2)	Aer**889** (911, 927), 1113	Aer**889** (911, 927)

XVI	575, 612, 771 **(793, 809)**	561, **(793**, 809)
*Blasia*, Soil	(877, 893)	**811, 827** (849, 865)
(SKS3)	1019 **(1041, 1057)**	1025, **(1041, 1057)**, 1075

XVIII *Blasia* (SKS8)	**508, 524/526, 568, 591/593**, 639, **657**, Nod**839**, Nod**853**, 1241, 1244, Nph1259 **(1281, 1297)**	ND

XX	568, 580, (597/599, **613/615**), 793, 955	**515, 561, 587**, 625
Soil (SKSF1)	(1337, 1353), (1499, 1515), (1588, 1604), (1823, 1839)	**858**

XXI Soil	**593**, 605, (626/628, **642/644**), (**683**, 697), 705/707, **719/721**, 735, 789 (811)	705/707, 719/721, 743/745, **768**
(SKSF3)	SB**829**, 847,SB**899**, SB**927**, SB**997**	811 (833, 849), 829 (851), **931 (953, 969), 947 (969, 985)**
	1017, SB1045 (1067,1083) 1099, SB1115 (1137, **1153**)	**1029** (1051), SB1045 (1067)

XXIII	(626/628, **642/644**), 688, 704	518
*Blasia* (SKJ2)	744 **(766, 782)**	**701**

XXIV *Blasia*	**593, 621, 693**, 721, 766, **782**	610, **656**, 750,766, **782**, 800, 829, 861, 909 (931, 947), SB**927** (949, 965)
(SKJ4)	SB829, 909, 913, SB**927** (949, 965), SB**997** SB1029, SB**1045** (1067, **1083**), SB1115 (1137, **1153**), **1668**	SB1029 (1051, 1067), SB1045 **(1067, 1083)**

XXV	593, 621, 771 (793, **809**)	534, 549, 744
*Blasia*, Soil	893	
(SKJ6)	1019 (1041, **1057**)	**(1041, 1057), 1075**

*The order corresponds to the line up from top to bottom in the phylogenetic tree shown in **Figure 3**. Only ions reproducibly detected in tree biological replicates are listed. The MALDI-TOF spectra are shown in Supplementary Figure S2.*

By conducting Q-TOF analyses followed by comparison with fragmentation patterns of known cyanobacterial metabolites, we were able to identify a number of metabolites (**Table 2**, Supplementary Figure S3).

**Table 2:** Summary of the compounds found in symbiotic *Nostoc* isolates assigned to known secondary metabolites produced by cyanobacteria.

m/z H^+^	Genotype	Diagnostic features	Reference
Aeruginosins:			
851	III, XII	Neutral loss of 176 or 162 Da	
865	II, X	Fragmentation pattern similar to Aeruginosin865	Kapuscik et al., 2013
875	I, VII, XIV,		
889	I, VI, VII, VIII, XIV, XV		
Banyaside/Suomilide -like			
899, 927, 997	X, XI, XXI, XXIV	Fragmentation pattern similar to Suomilide and Banyaside	Fujii et al., 2000; Schindler et al., 2010
1045, 1115 (sulfated)		Neutral loss 80 Da	
Microcystins:			
1009, 1023	I,VII, XIV, XXII	Fragmentation pattern match	Oksanen et al., 2004
Nodularins			
839, 853	XVIII	Fragmentation pattern match	Mazur-Marzec et al., 2006
Nosperin			
564	IX	Fragmentation pattern match	Kampa et al., 2013
Nostocyclopeptide			Golakoti et al., 2001
757	VI, VIII, XI	Neutral losses of meP, Q, I, Y, G, S	
Nostophycin			Fujii et al., 2000
1259	XVIII	Fragmentation pattern match from fragment m/z 889 Da and lower	

Dominant ions m/z 1009 and 1023 produced by closely related I, VII, XIV, and XXII were identified as ADMAdda-microcystins found earlier in *Nostoc* from lichens and an aquatic *Nostoc* sp. 152. The fragmentation profiles matched the microcystin variants described in Oksanen et al., 2004, including diagnostic ions at m/z 155, 265, and 627 Da (Supplementary Figures S3G,H). Two nodularins with protonated ion masses 839 and 853 (Supplementary Figures S3I,J), were found in isolates of genotype XVIII (*Nostoc* sp. SKS8). These compounds were assigned due to combination of diagnostic ions m/z 209, 227, and 253 Da. The same strain produced a peptide with the mass m/z 1259, the fragmentation pattern of which was identical to that reported for nostophycin m/z 889 Da (Fujii et al., 2000) starting with the ion peak m/z 889 Da and lower. The fragmentation pattern suggests an additional side chain with three or four amino acids (Supplementary Figure S3K).

A peptide with m/z 865 has previously been reported from a number of terrestrial *Nostoc* strains (Hrouzek et al., 2011). The structure of the compound has been recently characterized as glucuronated aeruginosin 865 (Kapuscik et al., 2013). The fragmentation patterns of m/z 865 detected in this work in genotypes I, II, VI, VIII, and XIV and that of aeruginosin 865 were identical (Supplementary Figure S3B). Taking into account the overall similarity in MS/MS spectra of the remainder peptides showing loss of 176 or 162 Da, namely m/z 851 in gtIII, and m/z 875 and m/z 889 in several isolates, we may conclude that those compounds are also aeruginosins (Supplementary Figures S3A-D). The absence of fragment ions diagnostic of un-glycosylated aeruginosins in fragmentation patterns of glycosylated and glucuronated aeruginosins may be due to the influence of the side chains. A similar effect of side chains was described for ADMAdda-microcystins whose fragmentation patterns do not display diagnostic ions of more common and well-studied Adda-microcystins (Sivonen et al., 1990).

Isolates assigned to genotypes XI and XXI produced an array of substances ranging from m/z 829 Da to m/z 1045 Da sharing similar fragmentation patterns (Supplementary Figure S3L). The lower parts of fragmentation spectra were nearly identical to that of glycosidic peptide suomilide from *Nodularia spumigena* (Fujii et al., 2000). A prominent fragment ion of m/z 610 was present in all fragmentation spectra. An ion with this mass was also recorded in natural and synthetic banyaside, a peptide structurally similar to suomilide but produced in *Nostoc* (Schindler et al., 2010). The fragmentation patterns of the variants with masses over 1000 Da showed a neutral loss of 80 Da, corresponding to a sulfate group, also indicative of suomilide and banyasides (Fujii et al., 2000; Schindler et al., 2010).

Cell and medium extracts of several genotypes contained a peptide with protonated mass m/z 757 Da, but observed mostly as sodium and potassium adducts as m/z 779 Da and 795 Da. The fragmentation pattern displayed multiple losses corresponding to metPro, Gly, Gln, Ile, Ser, and Tyr or dipeptides composed of these amino acids (Supplementary Figure S3F), which proved to be the exact composition and molecular mass of nostocyclopeptide a1, found in lichen isolate *Nostoc* sp. ATCC53789 (Golakoti et al., 2001).

The last compound that could be identified was nosperin m/z 564 Da (Supplementary Figure S3E) found in supernatant of genotype IX (*Nostoc* sp. KVJ10), an unusual product of NRPS biosynthesis involving trans-acyltransferase originating from a lichen cyanobiont (Kampa et al., 2013).

**FIGURE 4:**
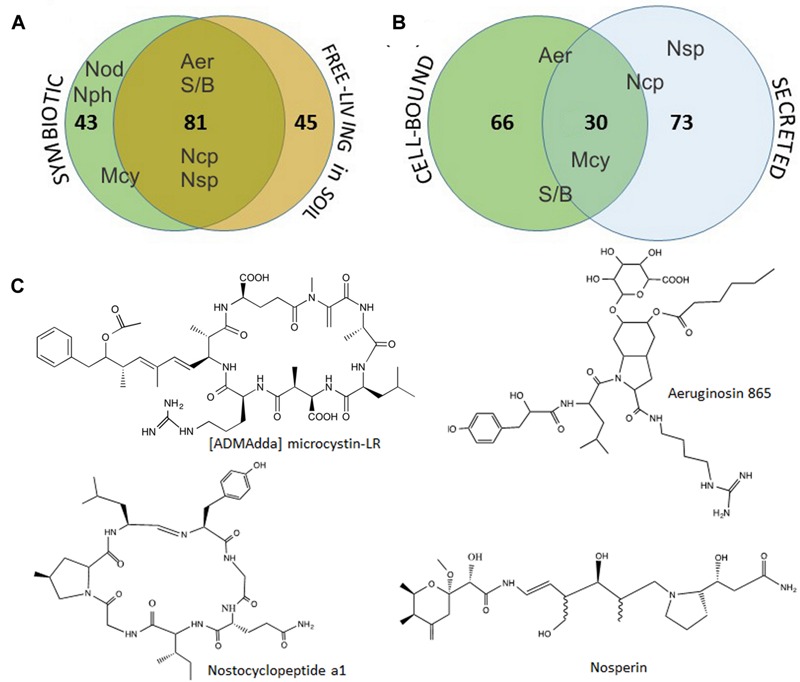
**Cumulative distribution of the 169 metabolites detected in *Nostoc* isolates obtained from symbiotic association with *B. pusilla* or from soil at the sampling site **(A)**, and the metabolites occurrence as cell-bound or secreted **(B)**.** Aer- aeruginosins, Mcy- [ADMAdda] microcystins, Ncp-nostocyclopeptide, Nod-nodularin, Nph-nostophycin, Nsp-nosperin, S/B-suomilide/banyaside-like compounds. The placement of the compound abbreviation shows it‘s tendency to be found as common for both sample sets/fraction or more specific to one. Selected examples of identified compounds, [ADMAdda] microcystin-LR, aeruginosin 865, nostocyclopeptide a1 and nosperin, are shown in **(C)**.

The identified compounds observed in cell samples are also indicated on the phylogenetic tree (**Figure 3**). Examples of the chemical structures found in symbiotic *Nostoc* in this work are shown in **Figure 4C**.

Further, we have compared the compound lists found in symbiotic isolates and those found in soil. Nearly half of the metabolites (80 out of 169) were common for both groups of isolates. Moreover, all the major metabolites, including identified aeruginosins, suomilide/banyaside, nostocyclopeptide, and microcystins were found in both sets of samples (**Figure 4A**; **Table 1**). The differences in compound lists were largely due to peaks uniquely found only in particular strains. Producers of most major metabolites were equally represented in free-living and symbiotic sets of samples, except for microcystin and nodularin producers. Notably, microcystin producers were found in all four plant samples, but only in one soil sample (Skibotn1, **Figure 2**). Moreover, in the three plant samples Kvaloya1, Skibotn1 and 2, microcystin producing strains had the highest share. Nodularin producer was found only in symbiotic state in Skibotn1 sample (**Figure 2**).

When comparing metabolite lists from cell-extract with the secreted metabolome, we noted that the overlap between two lists is rather small. Out of 169 compounds only 30 where found in both fractions (**Figure 4B**). Analyzing MALDI-TOF profiles, we noted that microcystins, when produced, were found with equal intensities in both sets of samples. Aeruginosins and suomilide compounds were most pronounced in cell extracts, while the opposite was true for nostocyclopeptides (**Figure 4B**; Supplementary Figure S2). The sets of major cell-bound compounds were shared by several genotypes. While the peak lists of prominent extracellular metabolites were nearly always unique for each particular genotype, when the dually targeted compounds were subtracted (**Table 1**). This was especially pronounced in the mass range from 500 to 800 Da.

### Bioactivities of Isolated *Nostoc*

**FIGURE 5:**
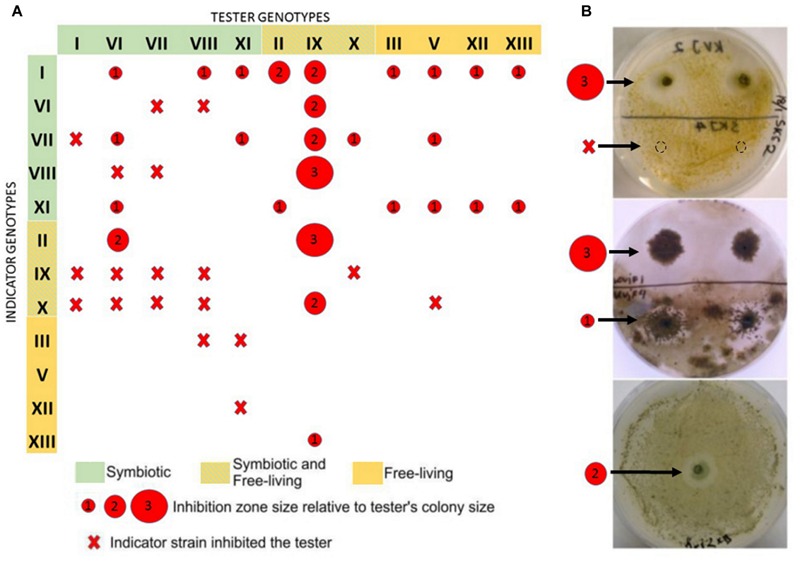
**Presentation of allelopathic interactions between cyanobacterial isolates from the Kvaloya sampling site A.** Summary of allelopathic interaction. **(A)** Summary table. The red circles represent the size of inhibition zone caused by the tester strains relative to its colonies radius, where 1 stands for inhibition zone up to 1- colony radius, 2- up to two radius, 3- from two radius sizes and larger. Red cross indicates that the indicator strain did not allow growth of the tester. **(B)** Examples of allelopathic assays conducted in this study.

We used two-layer agar bloc assay for detecting inhibitory activities among our isolates (**Figure 5B**). No pronounced inhibitory activity was found within the set of isolates from Skibotn. In contrast, cultivable *Nostoc* from Kvaloya site displayed a web of negative interactions between them (**Figure 5A**). Ten out of 12 cross-tested isolates showed inhibitory activity against at least one strain. Genotypes IX and VI showed the most powerful and broad range inhibition activity. Isolates with the most negative interactions directed against them were found only *in planta*. In order to rule out cyanophage infection as a cause of death, we have tested agar blocks from inhibition zones on new indicator lawn and did not observe any spreading (not shown). The cellular extract from the most active isolates, were tested on four clinical bacterial isolates (*E. faecalis, E. coli, S. aureus*, MRSA). The results of MIC test for all isolates were negative (not shown).

**FIGURE 6:**
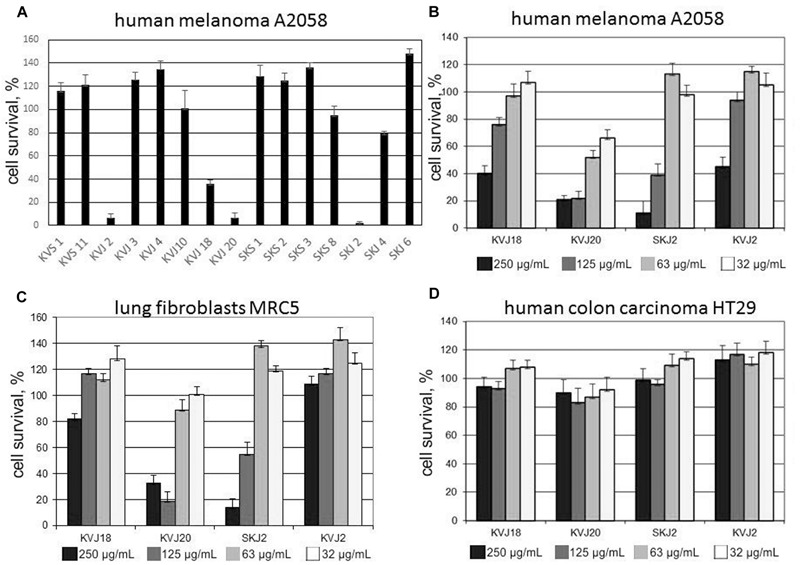
**Cytotoxic activity of cell extract from a selection of *Nostoc* isolated in this study. (A)** Cell survival test on human melanoma A2058 cell line applied 500 μg/mL crude extract from a selection of cyanobacterial isolates. **(B-D)** Cell survival tests performed with cell extracts of *Nostoc* sp. KVJ10, *Nostoc* sp. KVJ20, *Nostoc* sp SKJ2, and *Nostoc* sp. KVJ2 against human melanoma A2058, lung fibroblasts MRC5, and human colon carcinoma HT29 cell lines, respectively, at four different crude extract concentrations. Error bars-standard deviation of three technical replicates.

Cytotoxicity tests were performed with total extracts from all isolates showing vigorous growth in culture. Initial screening was performed only with A2068 metastatic human melanoma cell line (**Figure 6A**). Extracts from four isolates, KVJ2 (genotypeVI), KVJ18 (X), KVJ20 (XI), and SKJ2 (XXIII) were notably inhibiting cell growth (**Figure 6A**). These isolates were further tested against MRC5 fibroblasts and HT29 human colon cancer cell lines. The latter was not effected by any of the extracts. MRC5 cells were inhibited in dosage dependent manner by KVJ20 and SKJ2 extracts (**Figures 6B-D**). Further characterization of cytotoxic properties of these strains is a subject of separate study.

## Discussion

### Comments on the Genetic Diversity of *Nostoc* Recruited in Symbiosis

Here we report a difference in the cyanobiont diversity found even in two individual talli from the same location (**Figure 2**, Supplementary Figure S1). Our observations are in line with the previous report on cyanobacterial diversity in *B. pusilla* in England (West and Adams, 1997). The bryophyte host displayed promiscuity in recruitment of cyanobacterial partners, which were acquired locally. It is also likely that most of the cyanobacteria assigned to *Nostoc* would be able to act as a symbiont in this association. Several indications tell in support of general symbiotic competence in *Nostoc*. Most of the free-living isolates were able to infect *B. pusilla* under laboratory conditions (West and Adams, 1997). Free-living *Nostoc* isolates in our study are not phylogenetically distinct from symbiotic isolates, and it has been shown in several phylogeny reconstructions that symbiotic *Nostoc* are distributed through the entire *Nostoc* lineage (Enderlin and Meeks, 1983; Rasmussen and Svenning, 2001; Svenning et al., 2005; Papaefthimiou et al., 2008a). Epiphytic nitrogen-fixing cyanobacteria associated with bryophytes has been the topic of several recent publications and there is growing evidence that such associations are a widespread phenomenon (Bay et al., 2013; Sprent and Meeks, 2013). These new finding also tell in favor of symbiotic competence in genus *Nostoc* as rather general feature.

Nevertheless, our data also suggests that certain strains, microcystin producers in our case, are recruited more frequent than the others. Nilsson et al. (2005) have shown that in artificial association with rice roots, there was always one strain, which would outcompete others in the infection process. The feature was ascribed to faster hormogonia spreading, rather than ability to respond on plant elicited chemoattractant or inhibition of competitor strains. In natural population of Chilean *Gunnera magelanica* only one haplotype of *Nostoc* was found in an individual plant on the background of relatively high diversity of cyanobionts found in the population (Fernandez-Martinez et al., 2013). Obviously, there are yet unidentified selection processes taking place in each individual infection event. In our further discussion, we will argue that allelopathic interactions and the ability to produce certain secondary metabolites, at least in part, may be involved in shaping up symbiome in the host plant *B. pusilla*.

### Allelopathic Interactions and Biological Activities of Cyanobacterial Extract

Allelopathic activities among cyanobacteria have been reported several times in the past. Therefore, we found it worthwhile to test whether the differences between free-living and symbiotic communities can be explained by such interactions. None of the extracts inhibited other bacteria, suggesting that the inhibitory action observed is rather specific against cyanobacteria. Thus, it is feasible to assume that allelopathic pressure from the free-living competitors can in part explain the differences between sets of genotypes found in symbiosis and outside. The absence of detectable allelopathic activity among Skibotn isolates can be explained by the fact that the site is a natural habitat with little disturbance from human activities, the Skibotn community thus can be considered as settled. In contrast, Kvaloya site is a part of a garden center where new soil with presumably new cyanobacteria is added every vegetation season, thus the observed interactions could reflect on-going succession in the soil community. Until now, there has not been pointed out a clear benefit for cyanobacteria in entering into symbioses with plants. Protection from negative allelopathic interactions may be one of such benefits; then an individual genotype outcompeted as free-living can be still present at a given location in a symbiotic state. Persistence of defined cyanobiont genotypes in *B. pusilla* through several years at the same location (Costa et al., 2001) tells in support of this hypothesis.

Cyanobacteria are considered as potentially valuable source of biologically active compounds for pharmaceutical applications (Singh et al., 2011; Tan, 2013). Hrouzek et al. (2011) previously reported the potential of symbiotic *Nostoc* as a source of cytotoxic compounds. In their screening of terrestrial *Nostoc*, 60% of strains of symbiotic origin possessed the property; however, the fraction of symbiotic isolates was very small in the total set. Therefore, we were tempted to conduct an initial cytotoxicity screening of *Nostoc* isolated in this study. Cytotoxicity tests were performed with total extracts from all cultivable isolates. Initial screening was performed only with A2068 metastatic human melanoma cell line. Extracts from four isolates, KVJ2 (genotypeVI), KVJ18 (X), KVJ20 (XI), and SKJ2 (XXIII) were notably inhibiting cell growth (**Figure 6**). These isolates were further tested against MRC5 fibroblasts and HT29 human colon cancer cell lines. The latter was not effected by any of the extracts, while both A2068 and MRC5 cells were inhibited in dosage dependent manner by KVJ20 and SKJ2 extracts (**Figure 6**). The results suggest a general cytotoxicity of KVJ20 and SKJ2. Further characterization of cytotoxic properties of these strains and their potential as sources of new drug lead compounds will be a subject of separate study.

### Chemical Diversity of Symbiotic *Nostoc*

In this work we aimed to describe what bioactive potential symbiotic *Nostoc* have concerning their ability to produce small peptides, both associated with cellular material as well as secreted into the medium. Our results suggest that there is no obvious segregation, in regards of produced metabolites, between strains isolated from symbiotic association and from soil. Indeed, the cumulative overlap constituted nearly a half of the metabolites (**Figure 4A**). All observed major metabolites are included in the common set, including microcystins, aeruginosins nostocyclopetide, nosperin. For all samples combined, we noted that only 30 out of 169 recorded compounds are found in both cell-extracts and in the growth medium (**Figure 4B**). In fact, there are slightly more metabolites found as secreted. It can be speculated that indeed most of the small peptides are meant for secretion and are involved in extracellular functions such as communication, antibiotic activities and nutrient binding. Another immediate observation from peptide profiling of cells and media, is that many isolates have common sets of intracellular peptides (see **Table 1**). Such grouping became less apparent when the extracellular contents were compared. Most of the isolates show a unique extracellular peptide repertoire, especially in the range between 500 and 800 Da (**Table 1**). It is feasible to suggest that lower diversity and lower strain specificity in intracellular small peptides is related to their involvement in cellular processes, which are common and conserved among relatively close *Nostoc* isolates. Studies in quorum sensing and antibiotic production showed that secreted molecules, including small peptides, involved in communication and protection mechanisms, are often strain or species specific (Waters and Bassler, 2005; Fajardo and Martinez, 2008). A number of substances were found in both fractions. Even though there is a possibility of leakage from the cells, we would rather suggest that those substances are indeed truly dually targeted. This is supported by the fact that in the same samples, we have observed peaks that were detected exclusively in cells or vice versa only in supernatants (**Table 1**). Interestingly, the destination of the same compound may differ from isolate to isolate, when a compound in some strains is found solely as cell-bound while being dually targeted in others (**Table 1**).

In this study we did not aim to structurally elucidate individual peptides. However, we explored the possibility of assigning at least some of the compounds to previously described secondary metabolites (**Table 2**). We conducted extensive literature and database search in order to match observed masses to the existing records. The majority of the peaks could not be assigned to any candidate compound. In many cases when we would find a molecular weight match, the fragmentation patterns would not confirm our findings. We have also found that accumulated knowledge on cyanobacterial secondary metabolites is biased towards aquatic samples, which limited the possibility of precise identification in our study. There are still very few comprehensive studies of secondary metabolites in terrestrial (Hrouzek et al., 2011) or symbiotic cyanobacteria (Oksanen et al., 2004; Gehringer et al., 2010). Likewise, most of the compounds characterized in the literature, are obtained from the cell-extracts, creating a bias against secreted metabolites. This may be one of the reasons that almost none of the products found solely in the media was assigned to a known group of metabolites. This opens up an unexplored and prospective mine, since extracellular targeted biochemicals are most likely involved in interactions, and thus potentially possess activities desired, for example, in development of new antibiotics.

Nevertheless, the available reference data allowed us to identify a few previously reported compounds (**Table 2**, Supplementary Figure S3). Producers of cytotoxic microcystins and nodularins have been previously found in symbiotic associations in lichens and cycads (Oksanen et al., 2004; Gehringer et al., 2010). By identifying microcystins and nodularins in this work, we broaden the host range accepting potentially harmful cyanobionts. *B. pusilla* is a much better suitable model for studying the fate and regulation of cyanotoxins in association with eukaryotes. A further work with this interaction model may add more information on the biological role of microcystins and possible ways of manipulating their production.

There is an interesting group of compounds which include m/z 875 and 889 produced by genotypes I, VI, VII, XIV and XV, m/z 865 from II and X, and m/z 851 from III and XII. Fragmentation patterns of all these molecules share a common feature of a loss of either 176 or 162 Da, which is indicative of the presence of glucuronic acid or a hexose, respectively (Reinhold et al., 1995; Kapuscik et al., 2013). Glycosylation of non-ribosomal peptide and hybrid polyketide-peptide antibiotics in other bacteria is well known and was found to be important for the biological activity of such compounds (Walsh et al., 2001). Glycosylated aeruginosins described, until recently, exclusively in non-heterocystous cyanobacteria *Planctothrix* sp. were found in the same mass range as in our samples (Ishida et al., 2009). An antinflammatory peptide with m/z 865 has been previously reported from a number of terrestrial *Nostoc* strains (Hrouzek et al., 2011; Kapuscik et al., 2013). The structure of the compound was characterized as glucuronated aeruginosin-865 (Kapuscik et al., 2013). Our results show that production of aeruginosins is wide spread through *Nostoc* lineage. The very same is true for suomilide/banyaside like compounds, considered as related to aeruginosins (Pluotno and Carmeli, 2005).

Several *Nostoc* strains, found both in symbioses and in soil (genotypes VI, VIII, XI) produced nostocyclopeptide a1, which was found in cell material but was more pronounced in the media. Nostocyclopeptides are structurally similar to nostopeptolides, the gene cluster organization of *Ncp* and *Nos* operons share many common features, and there is a high degree of homology between key genes in the operons (Becker et al., 2004; Liu et al., 2014). This may suggest a similar biological function of the two compounds. In our recent work we showed that nostopeptolides are involved in regulation of the motile stage, hormogonia, acting as a suppressor of hormogonia formation (Liaimer et al., 2015), which may be also the function of nostocyclopeptides.

### Phylogeny and Symbiotic Competence in the Context of Chemodiversity

In this study, we isolated over twenty genetically distinct symbiotic strains from genus *Nostoc.* Each of the isolate possessed a unique secondary metabolite profile. Each symbiotic organ, auricle, was infected by a single clone, which makes the plant partner to an ideal fishing devise for isolation of nearly clean cyanobacterial strains from soils.

During the last decade several groups attempted to find relationships between phylogenetic affiliation of cyanobacterial strains and their ability to produce certain secondary metabolites (Martin et al., 1993; Dawson, 1998; Ersmark et al., 2008). Microcystins, hepatotoxins that inhibit eukaryotic protein phosphatase activity, are produced by a variety of unicellular and filamentous cyanobacteria. Consequently, this group of compounds deserved a thorough attention also in the phylogeny studies. It has been suggested that microcystin gene cluster arose very early in cyanobacterial evolution (Rantala et al., 2004). The same authors hypothesized that the wide but mosaic distribution of *mcy* genes in most cyanobacterial lineages is most likely due to the loss of the function rather than repeated gain through the horizontal gene transfer. However, the possibility of the latter is not excluded and is debated (Mikalsen et al., 2003; Tooming-Klunderud et al., 2008). The long time prevailing hypothesis on the function of microcystins was protection from eukaryotic grazers and/or allelopathic action against eukaryotic competitors (Leflaive and Ten-Hage, 2007). However, the ancient origin of the gene cluster and predominantly intracellular localization of microcystins suggest that these functions are a secondary effect of these compounds. The latest evidence tells for possible involvement of microcystins in responses to oxidative stress (Zilliges et al., 2011). In our results, the microcystin producing genotypes, I, VII, XIV and XXII are found to be dominant in symbiosis, while not found outside the plant. These four isolates are phylogenetically related to the lichen cyanobiont (**Figure 3**) Our results also indicate that production of microcystins does not give a competitive advantage at these particular soil environments. Contrary to our expectations, the plant-host did not discriminate potentially toxic cyanobionts. For the majority of plant-microbe interactions, it has been reported that the host plants elicit an oxidiative burst during the early stages of infection processes (Mehdy, 1994; Wojtaszek, 1997). Even though this aspect of infection process in cyanobacterial-plant symbioses has not been elucidated, it is feasible to hypothesize that microcystin producers are better fit upon entry into the plant partner due to oxidative stress protective mechanisms aided by microcystins, and probably, structurally similar nodularins.

In this study we also showed a wide spread and greater diversity of aeruginosins, and related to them suomilide and banyaside-like compounds. These glycopeptides where found at nearly all branches on the *Nostoc* phylogeny, and are constituvely produced in culture, making this genus to a unique source of glycosylated NRPS products for bioactivity trials. *Nostoc* sp. KVJ10 (genotype IX) was found as a source of nosperin, an unusual NRPS product, belonging to pederin family. Secondary metabolites of this type has been described only from the microorganisms involved in symbiotic interaction (Kampa et al., 2013). *Nostoc* sp. KVJ10 and the cyanolichen isolate are to date the only source bacteria which are easily maintained in culture, and therefore represent a valuable model for investigation of biological role of nosperin, and pederin like compounds in general.

Due to the lack of reference data, we were not able to assign the majority of detected peptides to any known peptide classes. Cyanobacteria isolated from symbiotic association with plants and fungi were found to produce a wide spectrum of secondary metabolites. In addition to variety of microcystins from cyanolichens (Kaasalainen et al., 2012) and nodularins from a cycad (Gehringer et al., 2012) there were few other reports suggesting a broad repertoire of secondary metabolites and small peptides in symbiotic *Nostoc*. Nostopeptolides and anabaenopeptins were found in *Nostoc punctiforme* PCC73102 (Welker and von Dohren, 2006; Rouhiainen et al., 2010) originally isolated from *Macrozamia* sp., additionally this particular strain is rich in gene clusters coding for NRPS and hybrid NRPS-PKS metabolites (Ehrenreich et al., 2005) as well ribosomally synthesizes bacteriocin-like and microviridin products (Wang et al., 2011). *Nostoc* sp. ATCC53789 isolated from a cyanolichen has been extensively studied as a source of biologically active cryptophycins. A number of compounds belonging to all major cyanobacterial peptide classes have been described from free-living *Nostoc* (Rezanka and Dembitsky, 2006). From the mass spectra described in our study one may expect even greater range of unknown compounds with diverse biological activities to be discovered. Even though we noticed the dominance of microcystin producers in *B. pusilla* samples in this study, strains with very different metabolites are also accepted in symbiosis. At this point we may not say that there is a distinct cyanobiont selection pattern based on secondary metabolites identity. It implies that compounds potentially harmful for eukaryotic hosts are either contained within symbiotic organs, or their production is suppressed on regulatory or altered on biosynthetic level, or they are degraded by the plant. Symbiotic plant partners, such as *B. pusilla* and *Gunnera* sp. were shown to interfere with secondary metabolite production in *N. punctifome* (Liaimer et al., 2015). The fate of cyanobacterial small peptides in symbiotic interactions will definitely be the focal point of our further investigations. Based on our findings we suggest that the accessory features, secondary metabolites in particular, do not have great influence on symbiotic competence, rather, it is has an impact on a strains fate in the free-living community. It is likely that the cyanobiont discrimination is determined by the host’s ability to manipulate a particular strain, and the key to this manipulation lies in the core genome of the *Nostoc*. In either scenario, the mechanisms of such interactions are intriguing and may have application beyond the field of plant-cyanobacterial symbioses.

## Conclusion

In this work we demonstrated a high genetic and chemodiversity of symbiotic nitrogen-fixing cyanobacteria in liverwort *B. pusilla* L. from two site in Northern Norway. We showed also that the host plant does not discriminate cyanotoxin producers, contrary, those were the dominant group in symbioses. The toxin production did not provide competitive advantage in free-living community. Cyanobacteria with strong allelopathic abilities can be a valuable source for compounds with cyanostatic activities. Additionally we showed that several isolates contain yet unidentified cytotoxic substances which can be used in anticancer drug development. Most of the peptide peaks observed in our study represent compounds with no records in the literature, thus making terrestrial cyanobacteria to perspective poorly explored source of novel natural products. For example, we showed a greater diversity and wide distribution of glycosylated NRPS products in this phylum. This study opens up for several intriguing question in regards to the fate and role of small peptides in plant symbioses.

## Author Contributions

AL: Design of the concept and experimental approach, sample collection, isolation, and cultivation of strains, analyses of STRR fingerprints, sequencing, allelopathy trials, MALDI-TOF analyses, interpretation of MS/MS data, figure preparation, manuscript writing, and guiding technical personnel. JJ: Design of the concept and experimental approach, phylogeny reconstruction and interpretation, manuscript writing, figure preparation, and guiding technical personnel. ED: Design of the concept and experimental approach, interpretation of MS/MS data, literature search, figure preparation, and manuscript writing.

## Conflict of Interest Statement

The authors declare that the research was conducted in the absence of any commercial or financial relationships that could be construed as a potential conflict of interest.
