# Supplementary material for: A Genetic and Chemical Perspective on Symbiotic Recruitment of Cyanobacteria of the Genus Nostoc into the Host Plant Blasia pusilla L
Source: Front Microbiol. 2016 Nov 1;7:1693. doi: 10.3389/fmicb.2016.01693 (PMC5088731; doi:10.3389/fmicb.2016.01693)
Supplement: Supplementary file 1 [file Table_1.PDF]

Table S1.
List of the cyanobacterial isolates and respective 16S rDNA sequences used in this study. Genotypes of our own isolates are designated according to Fig.2 in the main text. Several genotypes include individual isolates from different sources and therefore are represented by several GenBank records.

Genotype	Accession	Organism	Source/Habitat	Country	Reference
I	EU022725	*Nostoc* sp. KVS1	*Blasia pusilla*, plant school, Kvaloya	Norway	This study
II	EU022732	*Nostoc* sp. KVS11	*Blasia pusilla*, plant school, Kvaloya (also found in *Gunnera magellanica*)	Norway	This study
	EU022733	*Nostoc sp.* KVSF*1*	Free-Living, Soil, Kvaloya plant school	Norway	This study
	EU022734	*Nostoc* sp. KVJ1	*Blasia pusilla*, plant school, Kvaloya	Norway	This study
	EU022735	*Nostoc* sp. KVJF15	Free-Living, Soil, Kvaloya plant school	Norway	This study
III	EU022741	*Nostoc* sp. KVSF4	Free-Living, Soil, plant school, Kvaloya	Norway	This study
IV	EU022730	*Calothrix* sp. KVSF5	Free-Living, Soil, plant school, Kvaloya (lost in culture)	Norway	This study
V	EU022720	*Anabaena* sp. KVSF 7	Free-Living, Soil, plant school, Kvaloya	Norway	This study
	EU022721	*Anabaena* sp. KVJF17	Free-Living, Soil, plant school, Kvaloya	Norway	This study
VI	EU022712	*Nostoc* sp. KVJ2	*Blasia pusilla*, plant school, Kvaloya	Norway	This study
VII	EU022722	*Nostoc* sp. KVJ3	*Blasia pusilla*, plant school, Kvaloya	Norway	This study
VIII	EU022711	*Nostoc* sp. KVJ4	*Blasia pusilla*, plant school, Kvaloya	Norway	This study
IX	EU022708	*Nostoc* sp. KVJ10	*Blasia pusilla*, plant school, Kvaloya	Norway	This study
	EU022709	*Nostoc* sp. KVJF1	Free-Living, Soil, plant school, Kvaloya	Norway	This study
X	EU022728	*Nostoc* sp.KVJ18	*Blasia pusilla*, plant school, Kvaloya	Norway	This study
	EU022729	*Nostoc* sp. KVJF16	Free-Living, Soil, plant school, Kvaloya	Norway	This study
XI	EU022731	*Nostoc* sp.KVJ20	*Blasia pusilla*, plant school, Kvaloya	Norway	This study
XII	EU022710	*Nostoc* sp.KVJF4	Free-Living, Soil, Kvaloya plant school	Norway	This study
XIII	EU022740	*Nostoc* sp. KVJF8	Free-Living, Soil, Kvaloya plant school	Norway	This study
XIV	EU022738	*Nostoc* sp. SKS1	*Blasia pusilla*, Skibotn	Norway	This study
	EU022739	*Nostoc* sp.SKSF2	Free-Living, Soil, Skibotn	Norway	This study
XV	EU022714	*Nostoc* sp. SKS2	*Blasia pusilla*, Skibotn	Norway	This study
XVI	EU022736	*Nostoc* sp. SKS3	*Blasia pusilla*, Skibotn	Norway	This study
	EU022737	*Nostoc* sp. SKJF2	Free-Living, Soil, Skibotn	Norway	This study
XVII	EU022707	*Nostoc* sp. SKS5	*Blasia pusilla*, Skibotn *(*lost in culture)	Norway	This study

**XVIII**	EU022706	*Nostoc* sp. SKS8	*Blasia pusilla*, Skibotn	Norway	This study
**XIX**	EU022724	*Nostoc* sp. SKS9	*Blasia pusilla*, Skibotn (lost in culture)	Norway	This study
**XX**	EU022718	*Anabaena* sp. SKSF1	Free-Living, Soil, Skibotn	Norway	This study
	EU022719	*Anabaena* sp. SKJF11	Free-Living, Soil, Skibotn	Norway	This study
**XXI**	EU022723	*Nostoc* sp. SKSF3	Free-Living, Soil, Skibotn	Norway	This study
**XXII**	EU022742	*Nostoc* sp.SKJ1	*Blasia pusilla*, Skibotn	Norway	This study
**XXIII**	EU022717	*Nostoc* sp.SKJ2	*Blasia pusilla*, Skibotn	Norway	This study
**XXIV**	EU022715	*Nostoc* sp.SKJ4	*Blasia pusilla*, Skibotn	Norway	This study
**XXV**	EU022716	*Nostoc* sp.SKJ6	*Blasia pusilla*, Skibotn	Norway	This study
	EU022713	*Nostoc* sp.SKJF1	Free-Living, Soil, Skibotn	Norway	This study
	EU022726	*Nostoc* sp. SKSL1	*Peltigera canina*, Skibotn (was not isolated in culture)	Norway	This study
	EU022727	*Nostoc* sp. SKSL2	*Peltigera scabrosa*, Skibotn (was not isolated in culture)	Norway	This study
	AB039002	*Nodularia spumigena* PCC73104	Pasteur Culture Collection, Paris	France	1
	AF027653	*Nostoc* sp.TDI#AR94	Peltigera membranacea, Eagle Ridge, Vancouver, BC	Canada	2
	AF027655	*Nostoc punctiforme* PCC73102	Pasteur Culture Collection, originally isolated from a cycad, Paris	France	2
	AF062638	*Nostoc* sp. ATCC53789	University of Hawaii at Manoa, Honolulu	USA	3
	AF506237	cf. *Calothrix* sp.'muscicolous cyanobiont 5'	*Blasia pusilla*	Finland	4
	AJ000714	*Lyngbya aestuarii* PCC 7419	Pasteur Culture Collection, Paris	France	5
	AJ133161	*Nostoc* sp. 152	Lake Sääksjärvi	Finland	6
	AJ133169	*Planktothrix agardhii* NIVA-CYA 128	Lake Vesijärvi	Finland	6
	AJ293131	*Aphanizomenon flos-aquae* NIES-81	Lake Kasumigama	Japan	7
	AJ630449	*Nostoc esaphicum* X	Field, Chelčice	Czech Republic	8
	AJ630457	*Trichormus variabilis* str. 'GREIFSWALD'	Unknown	Germany	8
	AJ630458	*Anabaena augustumalis* str. 'SHCMIDKE JAHNKE/4a'	Rostock	Germany	8
	AM230690	*Calothrix* sp.BECID4	Brackish water, Baltic sea, Helsinki	Finland	9
	AM230702	*Calothrix* sp.BECID18	Sediment, sublittoral of the Baltic sea, Helsinki	Finland	9
	AM711527	*Nostoc* sp.0GU36S02	Stem gland of Gunnera tinctoria, Achill Island	Ireland	10
	AM711531	*Nostoc* sp.A13	Thallus of Anthoceros laevis, Tuscany	Italy	10
	AM711535	*Nostoc* sp.Ev1	Coralloid root , Emilia-Romanga, Bologna	Italy	10
	AM711536	*Nostoc* sp.Gm	Stem gland of *Gunnera manicata*, Siena, Tuscany	Italy	10
	AM711540	*Nostoc* sp.8926	Stem gland of *Gunnera hamiltonii*	New Zealand	10
	AM711545	*Nostoc* sp.Lukesova 1/86	Sample of oak forest mineral soil layer, Netolice, South Bohemia	Czech	10

			Republic	
AY328897	*Nostoc* sp.113.5	*Nephroma articum*, Helsinki	Finland	11
AY566855	*Nostoc* sp. IO-102-I	*Pannaria pezizoides* collected from mosses on a rock, Sysmä	Finland	12
AY742449	*Nostoc* sp. 8963	*Gunnera prorepens*	New Zealand	13
AY742451	*Nostoc* sp. 9229	*Gunnera monoika*	New Zealand	13
DQ185201	*Nostoc punctiforme* SAG 71.79	Soil	France	14
DQ185211	*Nostoc* sp. 'Mollenhauer 1:1-115'	*Blasia pusilla*	Germany	14
DQ185212	*Nostoc* sp. 'Mollenhauer 1:1-125'	*Blasia pusilla*	Germany	14
DQ185213	*Nostoc* sp. 'Mollenhauer 1:1-150b'	*Anthoceros* sp.2	Germany	14
DQ185244	*Nostoc* sp. '*Peltigera degeni* cyanobiont'	*Peltigera degenii*	Canada	14
DQ185245	*Nostoc* sp. '*Peltigera didactyla* 3 cyanobiont'	*Peltigera didactyla*	Poland	14
DQ185249	*Nostoc* sp. '*Peltigera rufescens* 5 cyanobiont'	*Peltigera rufescens*	Poland	14
DQ185251	*Nostoc* sp. SAG 36.92	*Stangeria paradoxa*	England	14
DQ185254	*Nostoc muscorum* SAG 57.79	Soil	France	14
DQ185255	*Nostoc punctiforme* SAG 65.79	*Blasia pusilla*	Germany	14
DQ185256	*Nostoc punctiforme* SAG 68.79	*Gunnera manicata*	Germany	14
DQ234831	*Trichormus variabilis* str. KCTC AG10178	Unknown	Korea	15
DQ279769	*Arthrospira platensis* str. Sp-9	Unknown	China	16
EF174212	*Nostoc* sp. '*Pannaria* aff.*leproloma* cyanobiont' 1a NZ	*Pannaria* sp.aff. *leproloma*	New Zealand	17
EF568907	*Anabaena* sp. XPORK36C	Planctonic and benthic habitats, Baltic Sea, Helsinki	Finland	18
BA000019	*Nostoc* sp. PCC 7120	Robert Haselkorn, University of Chicago	USA	19
GQ443447	*Anabaena cylindrica* UTAD_A212	Rice paddy, Mondego River Basin	Portugal	20
HM623781	*Nostoc* sp. UAM308	Rock surface of calcareous river with brackish water, Amir River, Murcia	Spain	21

1. T. Ishida, M. M. Watanabe, J. Sugiyama, A. Yokota, Evidence for polyphyletic origin of the members of the orders of Oscillatoriales and Pleurocapsales as determined by 16S rDNA analysis. *FEMS microbiology letters* **201**, 79 (Jul 10, 2001).
2. V. P. W. Miao, A. Rabenau, A. Lee, Cultural and molecular characterization of photobionts of Peltigera membranacea. *Lichenologist* **29**, 571 (Nov, 1997).
3. D. Hoffmann, J. M. Hevel, R. E. Moore, Direct Submission. *Submitted, Chemistry, University of Hawaii at Manoa*, (1998).
4. J. Rikkinen, I. Oksanen, K. Lohtander, Lichen guilds share related cyanobacterial symbionts. *Science* **297**, 357 (Jul 19, 2002).
5. U. Nubel, F. Garcia-Pichel, G. Muyzer, PCR primers to amplify 16S rRNA genes from cyanobacteria. *Applied and environmental microbiology* **63**, 3327 (Aug, 1997).

6. C. Lyra *et al.*, Molecular characterization of planktic cyanobacteria of Anabaena, Aphanizomenon, Microcystis and Planktothrix genera. *International journal of systematic and evolutionary microbiology* **51**, 513 (Mar, 2001).
7. M. Gugger *et al.*, Phylogenetic comparison of the cyanobacterial genera Anabaena and Aphanizomenon. *International journal of systematic and evolutionary microbiology* **52**, 1867 (Sep, 2002).
8. P. Rajaniemi *et al.*, Phylogenetic and morphological evaluation of the genera Anabaena, Aphanizomenon, Trichormus and Nostoc (Nostocales, Cyanobacteria). *International journal of systematic and evolutionary microbiology* **55**, 11 (Jan, 2005).
9. L. M. Sihvonen *et al.*, Strains of the cyanobacterial genera Calothrix and Rivularia isolated from the Baltic Sea display cryptic diversity and are distantly related to Gloeotrichia and Tolypothrix. *Fems Microbiol Ecol* **61**, 74 (Jul, 2007).
10. D. Papaefthimiou *et al.*, Differential patterns of evolution and distribution of the symbiotic behaviour in nostocacean cyanobacteria. *International journal of systematic and evolutionary microbiology* **58**, 553 (Mar, 2008).
11. I. Oksanen, K. Lohtander, K. Sivonen, J. Rikkinen, Repeat-type distribution in trnL intron does not correspond with species phylogeny: comparison of the genetic markers 16S rRNA and trnL intron in heterocystous cyanobacteria. *International journal of systematic and evolutionary microbiology* **54**, 765 (May, 2004).
12. I. Oksanen *et al.*, Discovery of rare and highly toxic microcystins from lichen-associated cyanobacterium Nostoc sp. strain IO-102-I. *Applied and environmental microbiology* **70**, 5756 (Oct, 2004).
13. M. M. Svenning, T. Eriksson, U. Rasmussen, Phylogeny of symbiotic cyanobacteria within the genus Nostoc based on 16S rDNA sequence analyses. *Archives of microbiology* **183**, 19 (Jan, 2005).
14. H. E. O'Brien, J. Miadlikowska, F. Lutzoni, Assessing host specialization in symbiotic cyanobacteria associated with four closely related species of the lichen fungus Peltigera. *Eur J Phycol* **40**, 363 (Nov, 2005).
15. G. G. Choi, Oh, H.M., Phylogenetic diversity of Nostocaceae, inferres from 16S rRNA gene and cpcBA-intergentic spacer sequence analyses. *Direct submission, Unpublished*, (2005).
16. L. Yang, Cao, X., Wang, Z., Chen, X., Li, X. Huang, H. and Xu, B., Application of cpcHID operon to the systematic classification study of Spirulina (Arthrospira) platensis. *Direct submission, Unpublished*, (2005).
17. A. Elvebakk, D. Papaefthimiou, E. H. Robertsen, A. Liaimer, Phylogenetic patterns among Nostoc cyanobionts within Bi- and tripartite lichens of the genus Pannaria. *J Phycol* **44**, 1049 (Aug, 2008).
18. K. Halinen *et al.*, Genetic diversity in strains of the genus Anabaena isolated from planktonic and benthic habitats of the Gulf of Finland (Baltic Sea). *Fems Microbiol Ecol* **64**, 199 (May, 2008).
19. T. Kaneko *et al.*, Complete genomic sequence of the filamentous nitrogen-fixing Cyanobacterium anabaena sp strain PCC 7120. *DNA Res* **8**, 205 (Oct 31, 2001).
20. V. Galhano *et al.*, Morphological, biochemical and molecular characterization of Anabaena, Aphanizomenon and Nostoc strains (Cyanobacteria, Nostocales) isolated from Portuguese freshwater habitats. *Hydrobiologia* **663**, 187 (Mar, 2011).
21. P. Mateo *et al.*, Life cycle as a stable trait in the evaluation of diversity of Nostoc from biofilms in rivers. *Fems Microbiol Ecol* **76**, 185 (May, 2011).
