# Supplementary material for: A Genetic and Chemical Perspective on Symbiotic Recruitment of Cyanobacteria of the Genus Nostoc into the Host Plant Blasia pusilla L
Source: Front Microbiol. 2016 Nov 1;7:1693. doi: 10.3389/fmicb.2016.01693 (PMC5088731; doi:10.3389/fmicb.2016.01693)

FigS1. Summary of STRR genotypes of the isolates from *Blasia pusilla* L. and soil to which the plants were attached. Two plants from Kvaløya island and two plants from Skibotn location were studied. The number of isolates was 200 for each induvidual plant and soil sample. The abundance of the genotypes is shown in Fig2 in the main text.

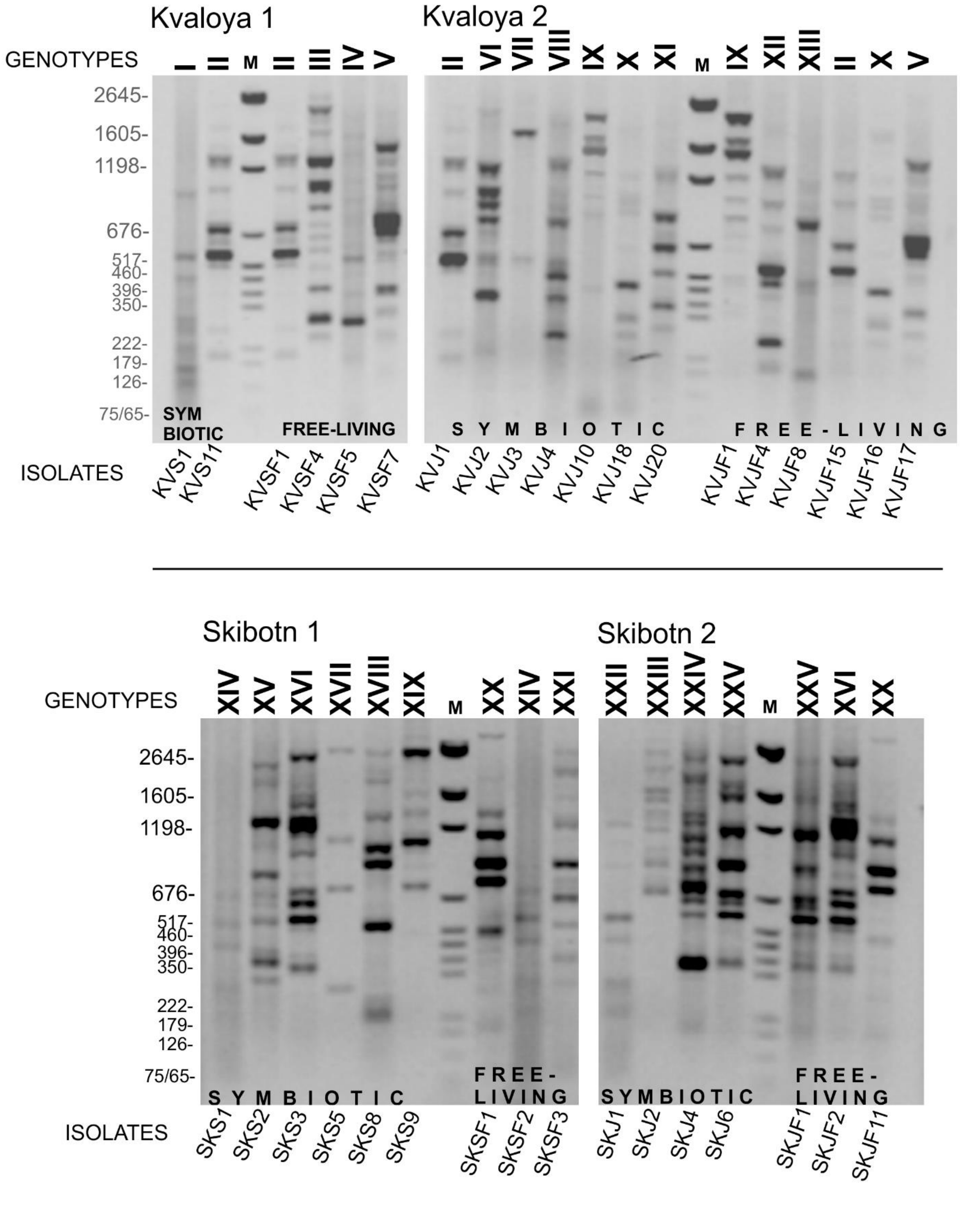

Supplement: Supplementary file 2 [file Image_1.PDF]
