# Supplementary material for: A Genetic and Chemical Perspective on Symbiotic Recruitment of Cyanobacteria of the Genus Nostoc into the Host Plant Blasia pusilla L
Source: Front Microbiol. 2016 Nov 1;7:1693. doi: 10.3389/fmicb.2016.01693 (PMC5088731; doi:10.3389/fmicb.2016.01693)

Fig S2.

MALDI-TOF profiles of cyanobacterial strains isolated in this study. Profiles obtained from the lyophilised cell pellets are to the left, and profiles generated from lyophilised growth medium are to the right. The results are summarezed in the Table 1 in the main text. The profiles are presented in the order of genotype numbering.

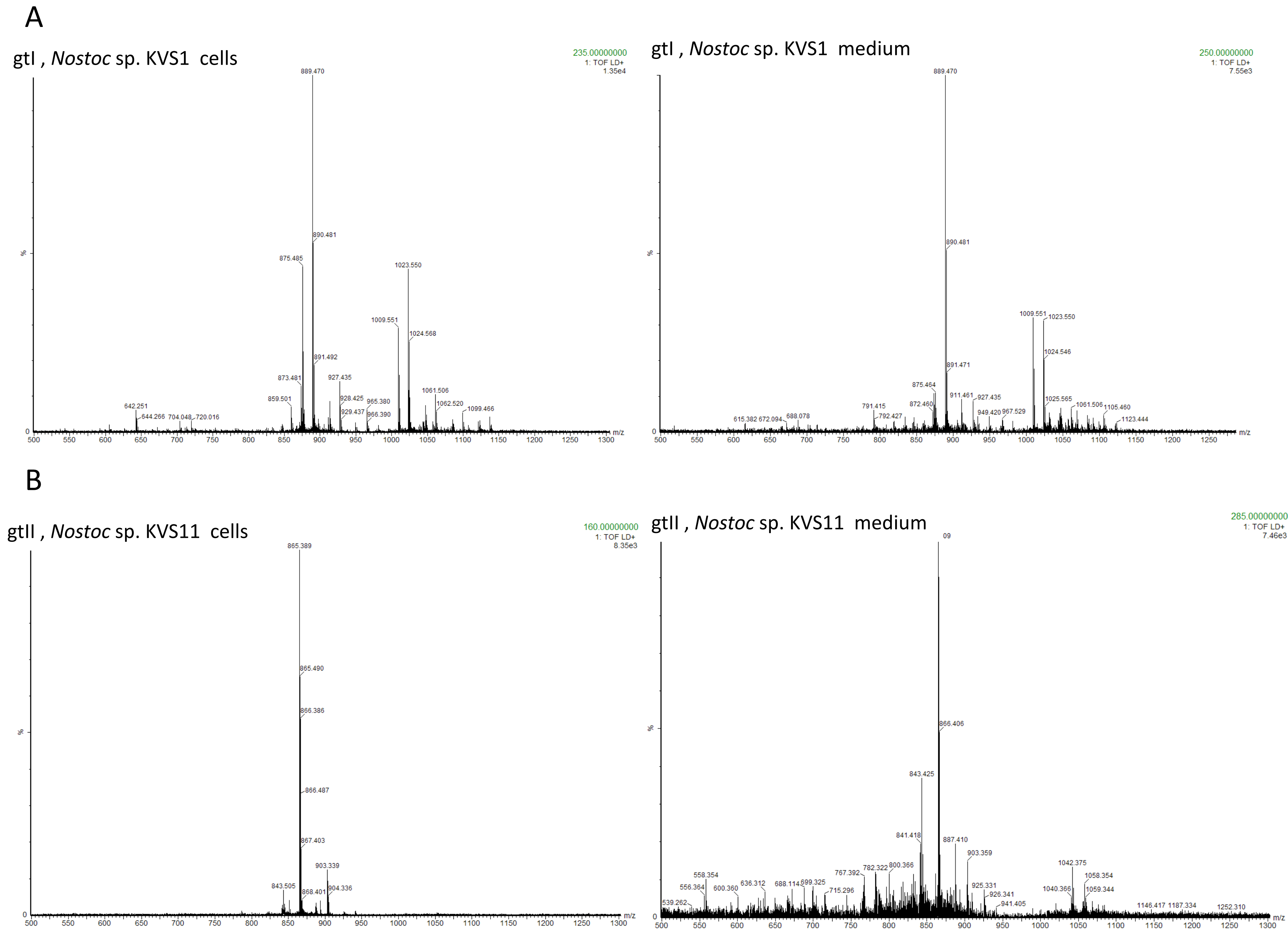
A
gtI , Nostoc sp. KVS1 cells
235.00000000
1: TOF LD+
1.35e4
889.470
890.481
875.485
1023.550
1009.551
1024.568
891.492
927.435
873.481
859.501
928.425
965.380
929.437
966.390
1061.506
1062.520
1099.466
642.251
644.266
704.048
720.016
gtI , Nostoc sp. KVS1 medium
250.00000000
1: TOF LD+
7.55e3
889.470
890.481
1009.551
1023.550
1024.546
891.471
875.464
872.460
911.461
927.435
949.420
967.529
1025.565
1061.506
1105.460
1123.444
791.415
792.427
615.382
672.094
688.078
B
gtII , Nostoc sp. KVS11 cells
160.00000000
1: TOF LD+
8.35e3
865.389
865.490
866.386
866.487
867.403
903.339
843.505
868.401
904.336
gtII , Nostoc sp. KVS11 medium
285.00000000
1: TOF LD+
7.46e3
09
866.406
843.425
841.418
887.410
903.359
1042.375
1058.354
1040.366
1059.344
925.331
926.341
941.405
767.392
782.322
800.366
558.354
556.364
600.360
636.312
688.114
699.325
715.296
539.262
1146.417
1187.334
1252.310
%
m/z
0

C

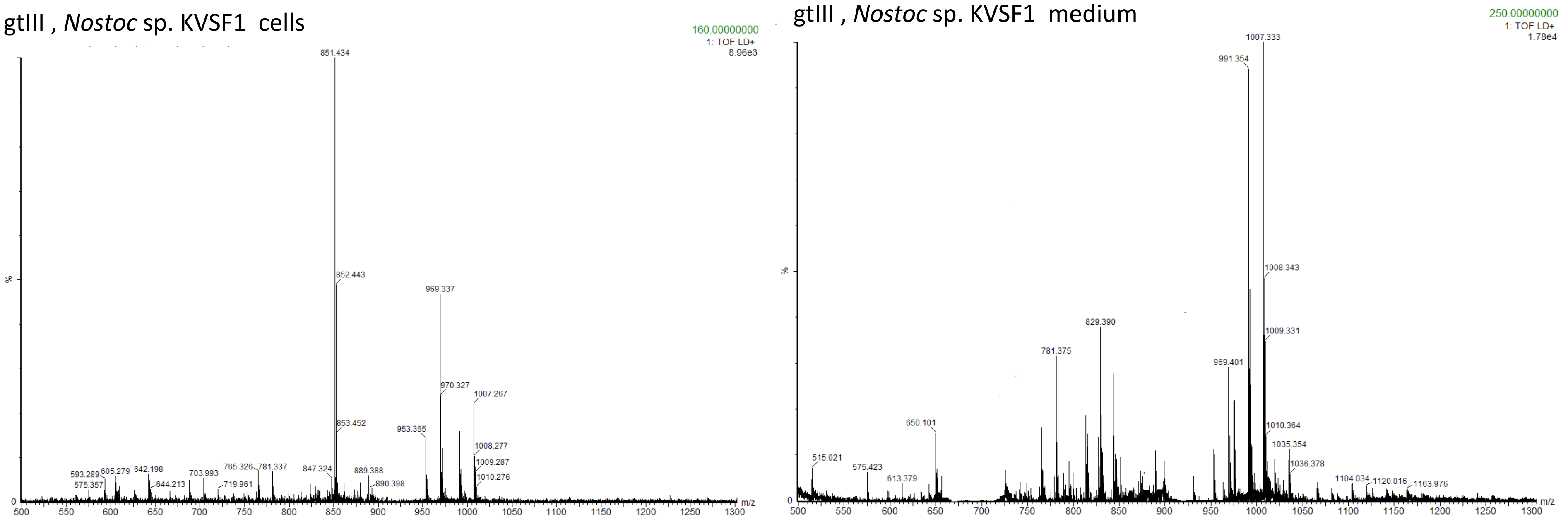
gtIII , *Nostoc* sp. KVSF1 cells
160.00000000
1: TOF LD+
8.96e3
851.434
852.443
969.337
970.327
1007.267
853.452
953.365
1008.277
1009.287
1010.276
593.289
605.279
642.198
575.357
644.213
703.993
765.326
781.337
719.961
847.324
889.388
890.398
%
m/z
gtIII , *Nostoc* sp. KVSF1 medium
250.00000000
1: TOF LD+
1.78e4
1007.333
991.354
1008.343
1009.331
829.390
781.375
969.401
650.101
1010.364
1035.354
1036.378
515.021
575.423
613.379
1104.034
1120.016
1163.976
%
m/z

D

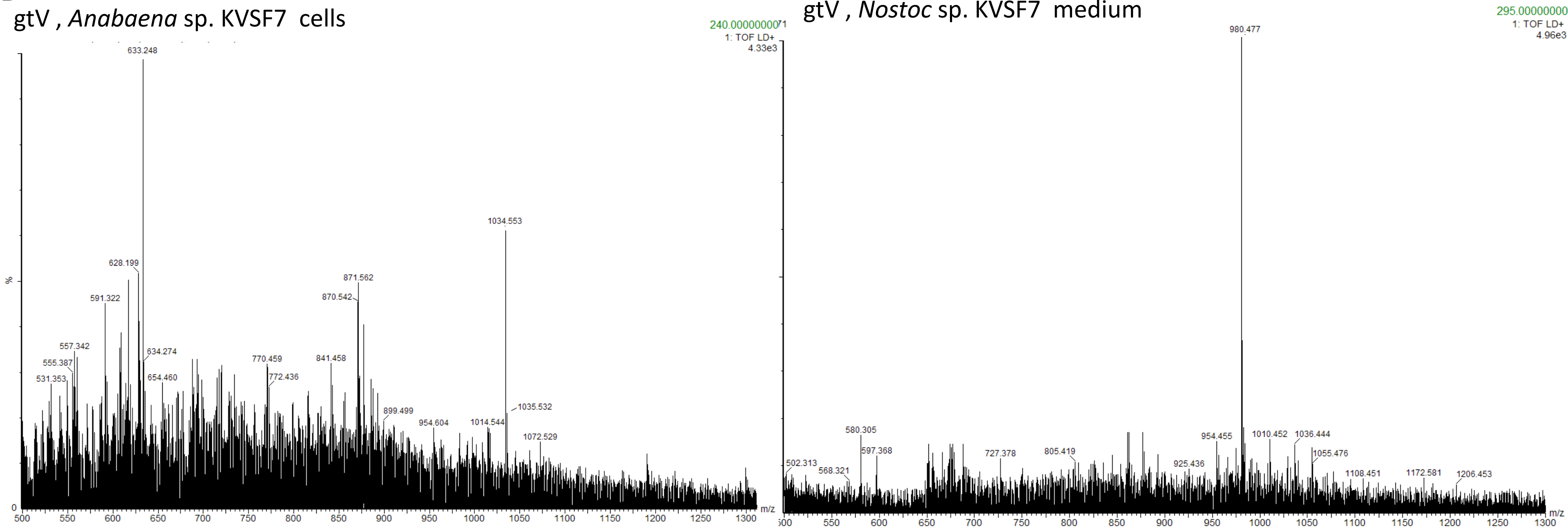
gtV , *Anabaena* sp. KVSF7 cells
240.00000000
1: TOF LD+
4.33e3
633.248
1034.553
628.199
871.562
591.322
870.542
557.342
634.274
555.387
770.459
841.458
531.353
654.460
772.436
899.499
1035.532
954.604
1014.544
1072.529
%
m/z
gtV , *Nostoc* sp. KVSF7 medium
295.00000000
1: TOF LD+
4.96e3
980.477
580.305
597.368
954.455
1010.452
1036.444
1055.476
502.313
568.321
727.378
805.419
925.436
1108.451
1172.581
1206.453
m/z

E

gtVI , *Nostoc* sp. KVJ2 cells

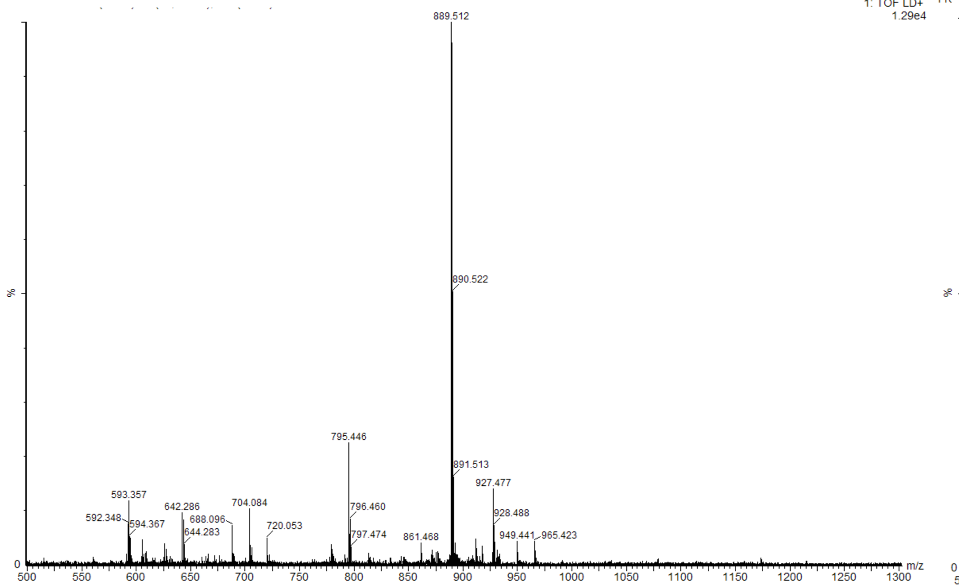

gtVI, *Nostoc* sp. KVJ2 medium

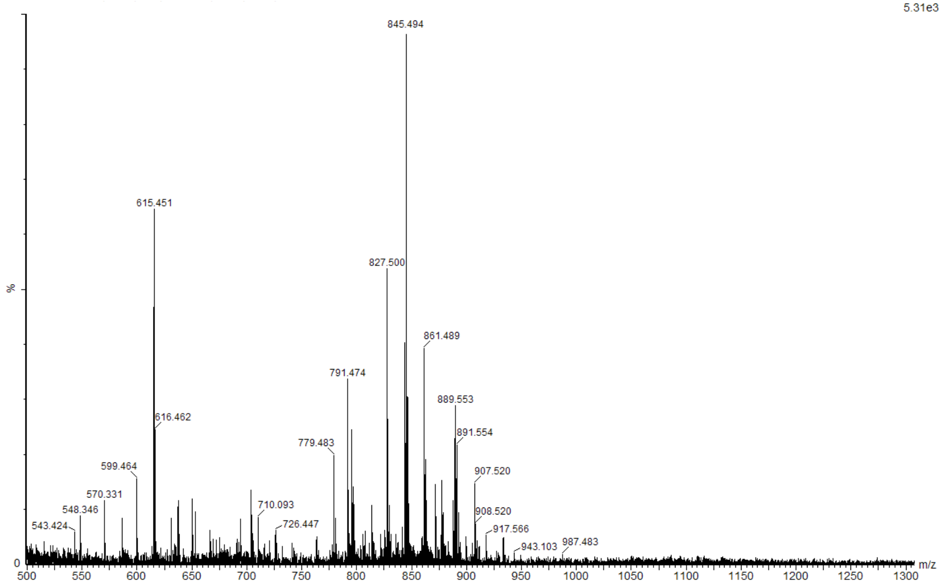

F

gtVII , *Nostoc* sp. KVJ3 cells

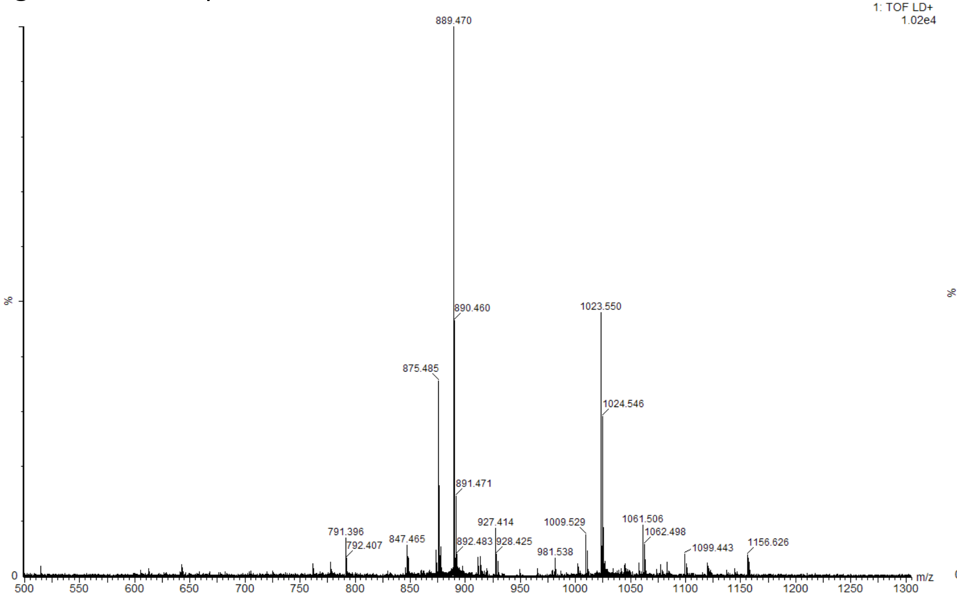

gtVII, *Nostoc* sp. KVJ3 medium

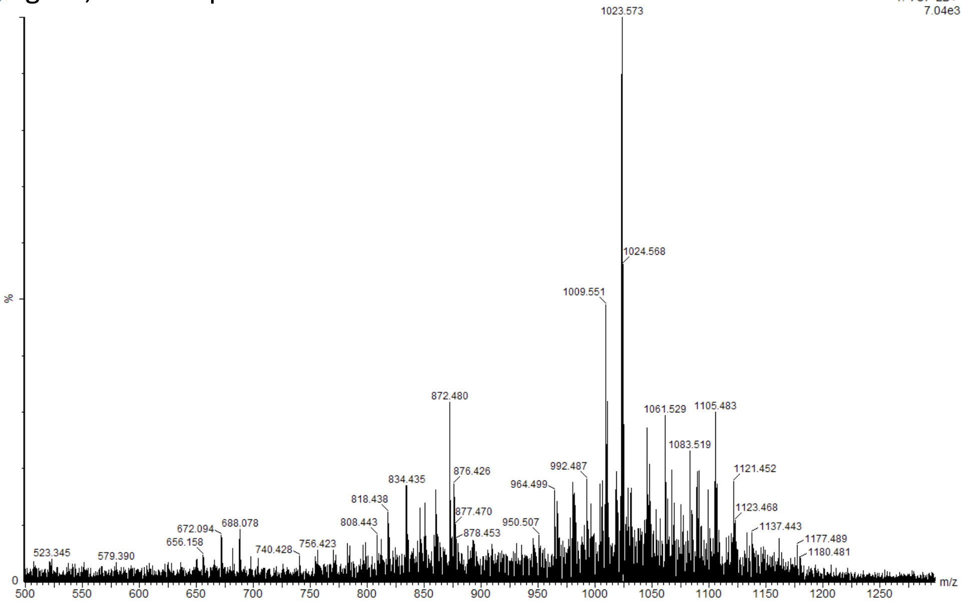

G

gtVIII , *Nostoc* sp. KVJ4 cells

gtVIII, *Nostoc* sp. KVJ4 medium

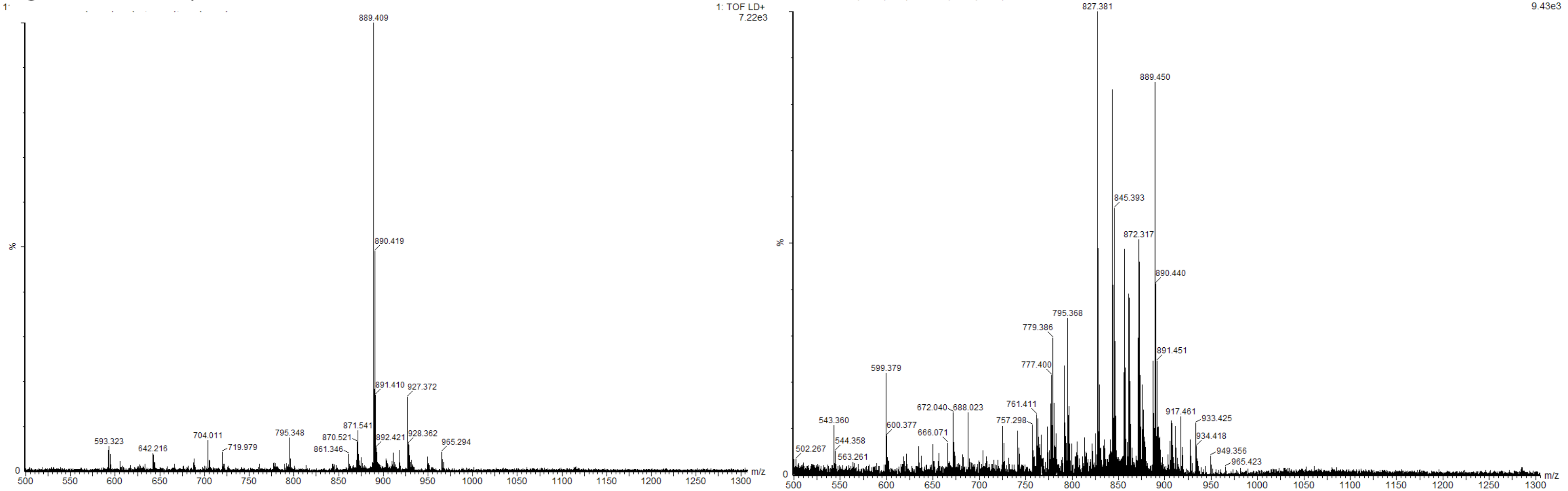

H

gtIX , *Nostoc* sp. KVJ10 cells

gtIX, *Nostoc* sp. KVJ10 medium

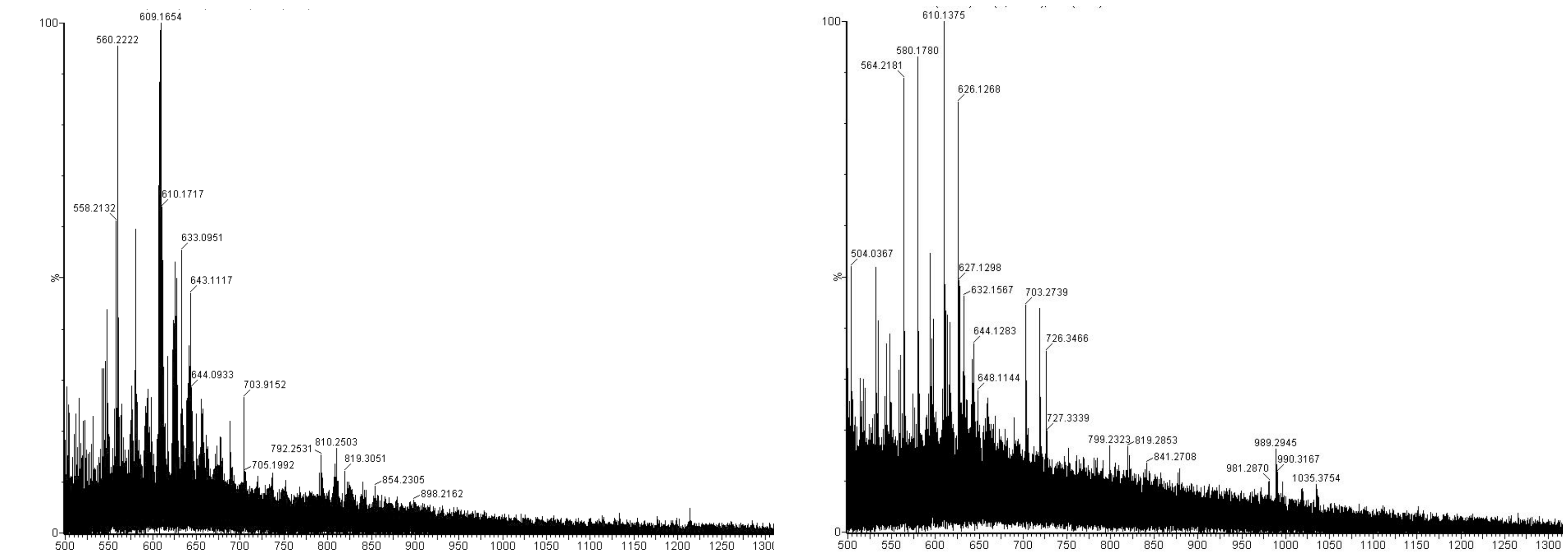

I

gtX , *Nostoc* sp. KVJ18 cells

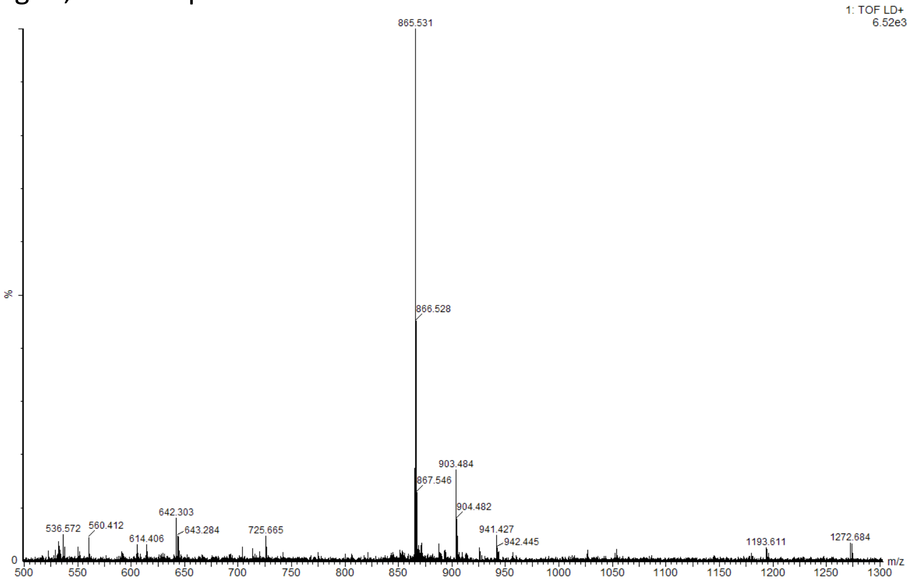

gtX, *Nostoc* sp. KVJ18 medium

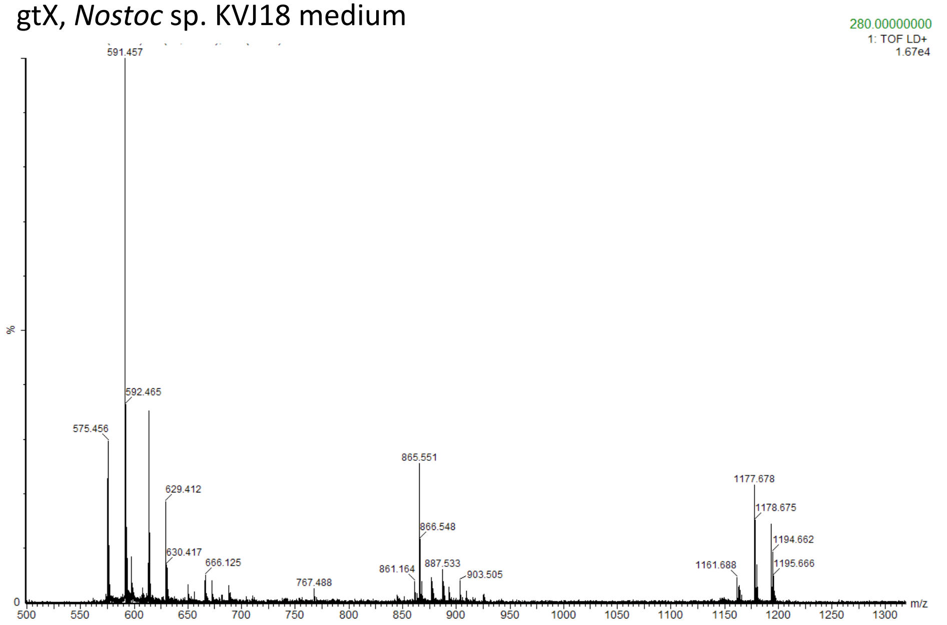

J

gtXI , *Nostoc* sp. KVJ20 cells

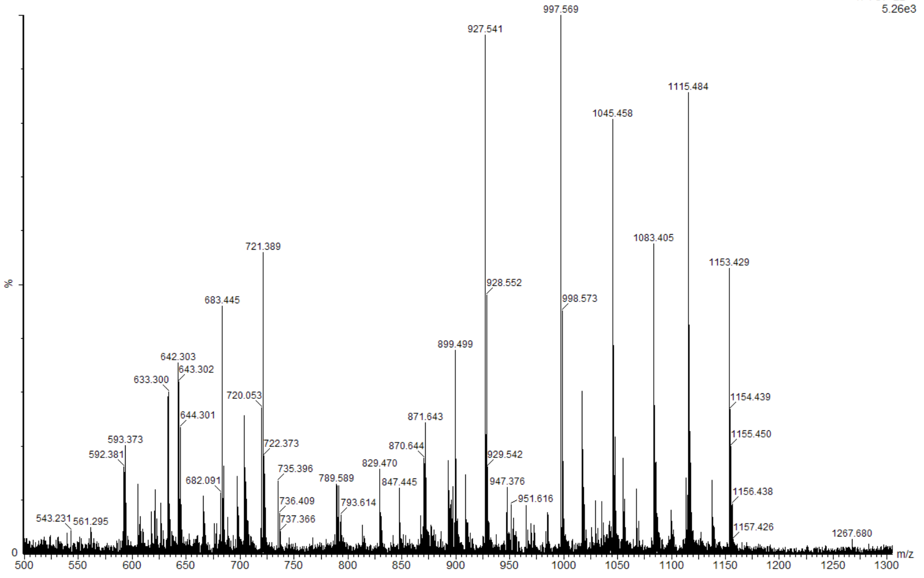

gtXI, *Nostoc* sp. KVJ20 medium

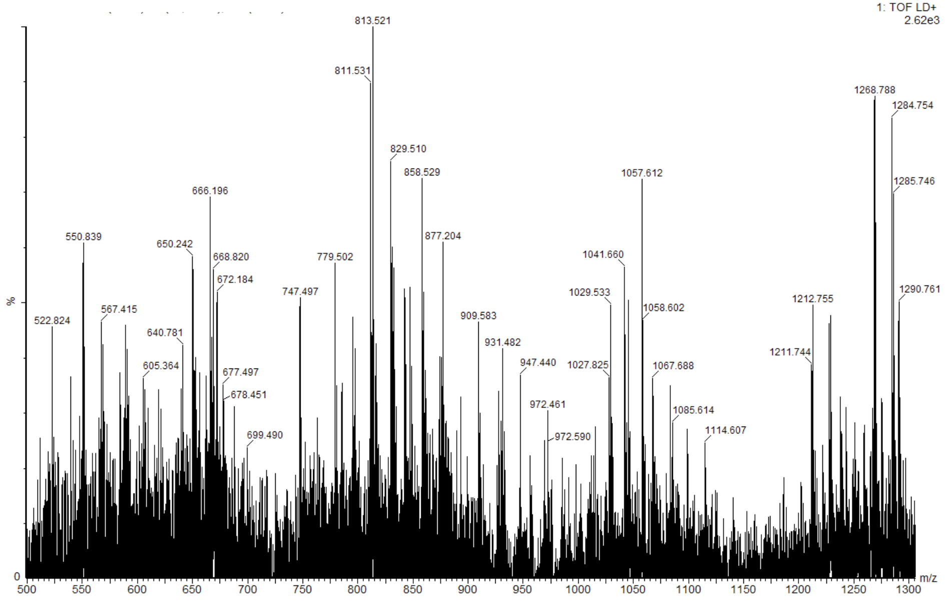

K

gtXII , *Nostoc* sp. KVJF4 cells

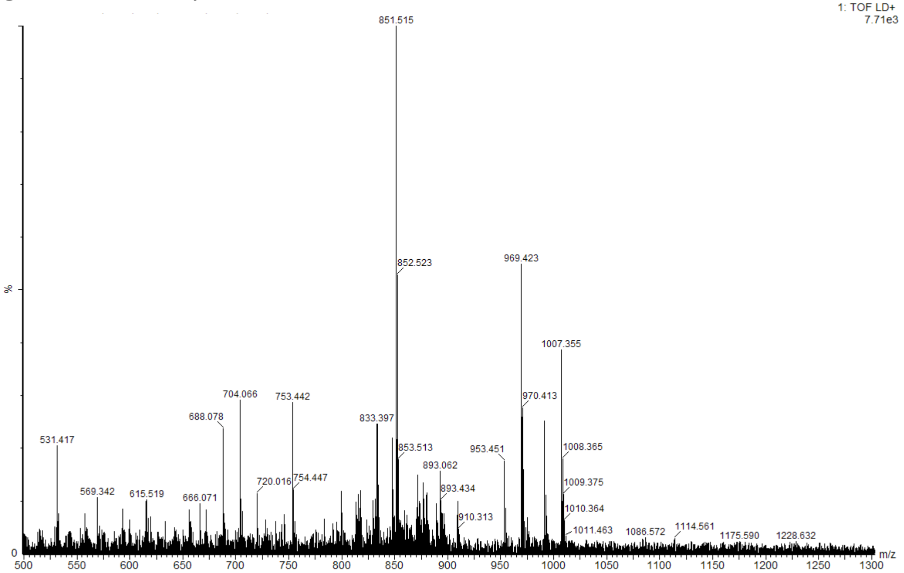

gtXII, *Nostoc* sp. KVJF4 medium

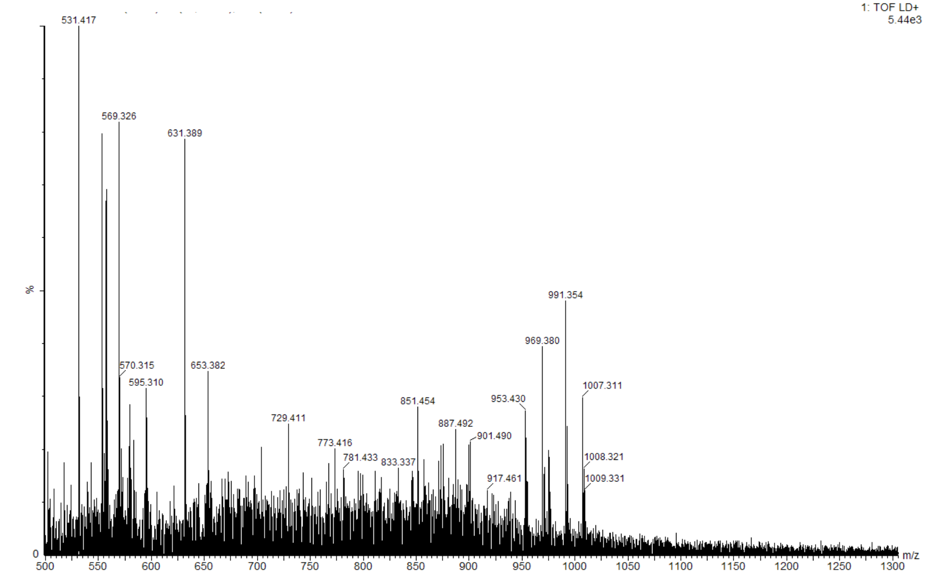

L

gtXIV , *Nostoc* sp. SKS1 cells

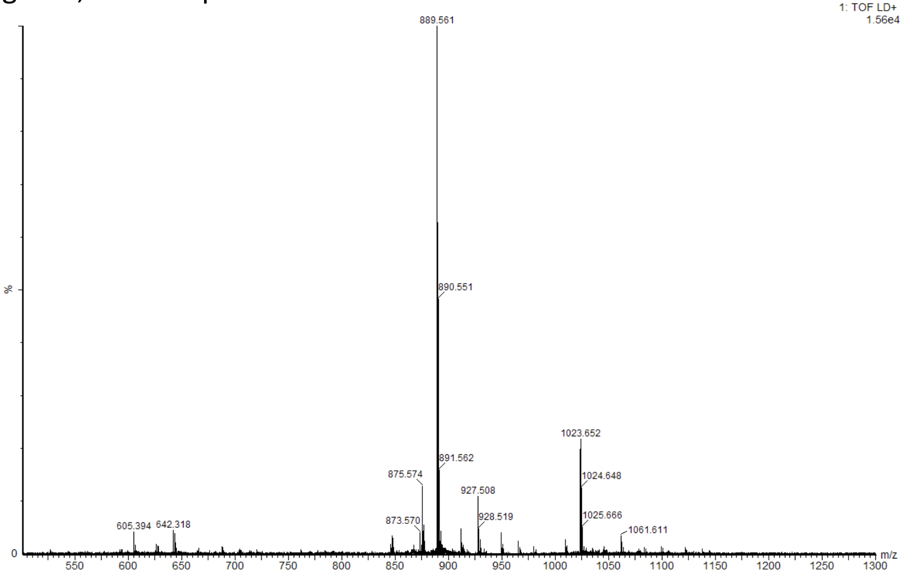

gtXIV, *Nostoc* sp. SKS1 medium

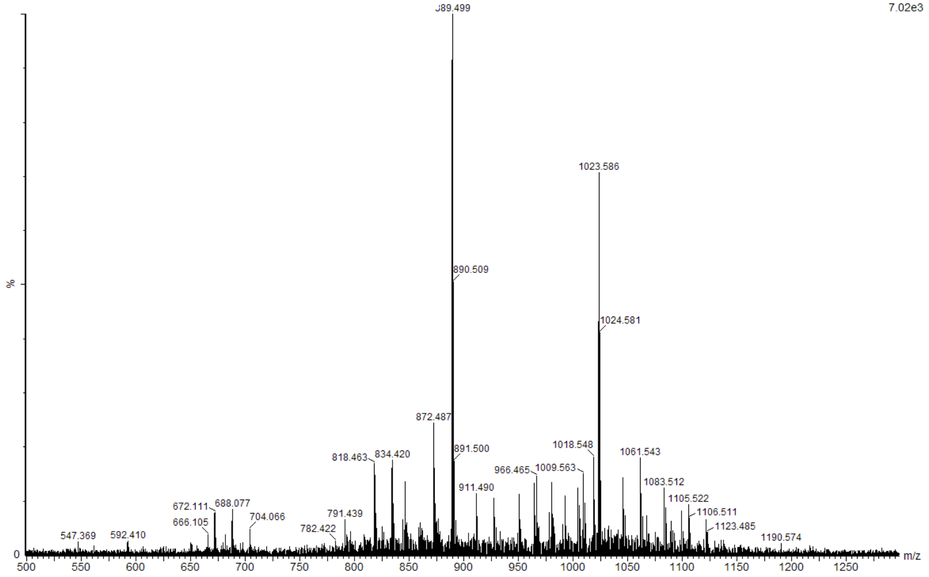

M

gtXV , *Nostoc* sp. SKS2 cells

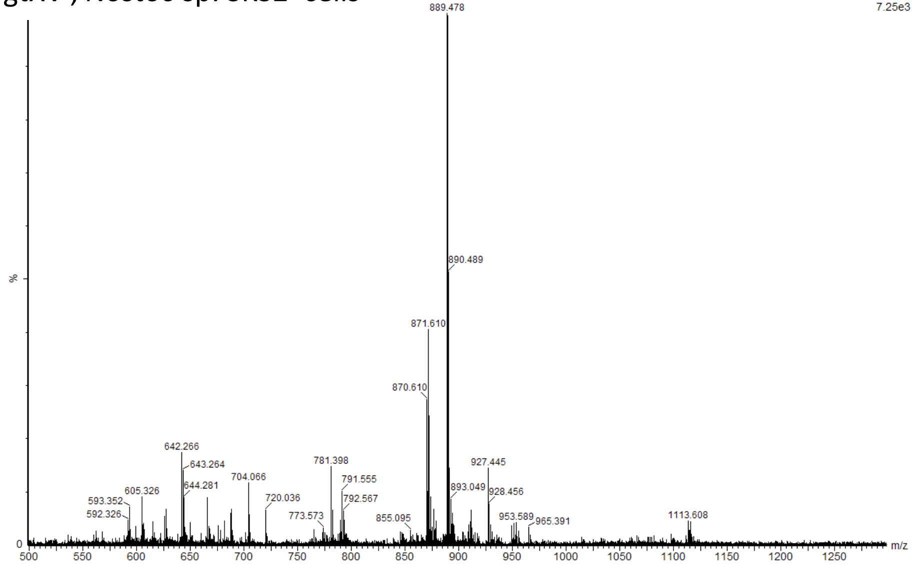

gtXV, *Nostoc* sp. SKS2 medium

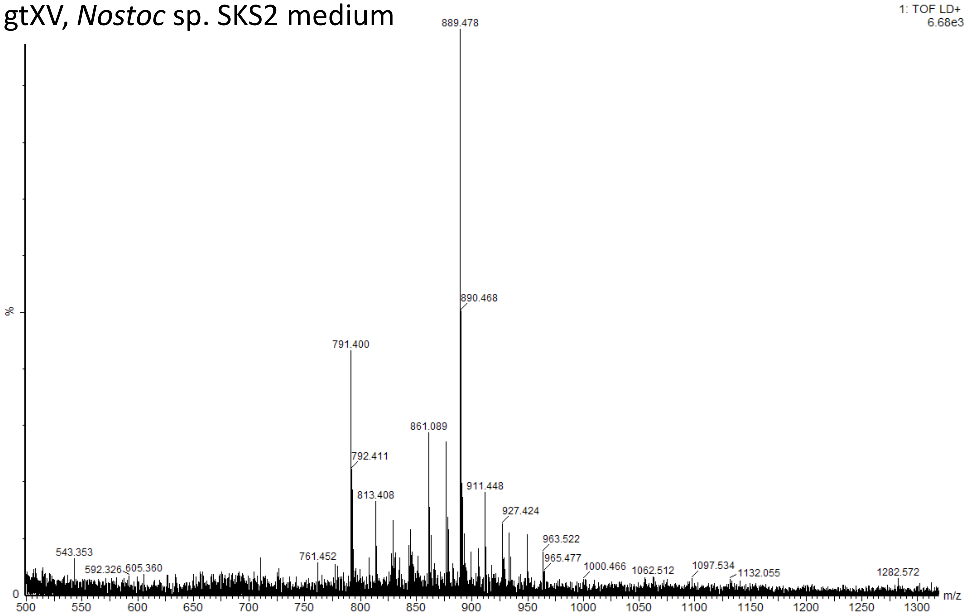

N

gtXVI , *Nostoc* sp. SKS3 cells

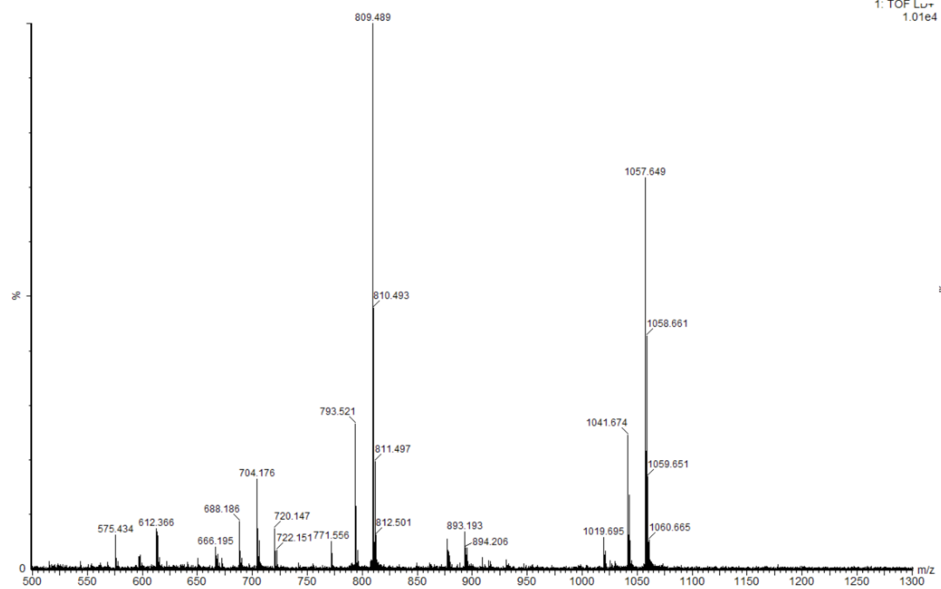

gtXVI, *Nostoc* sp. SKS3 medium

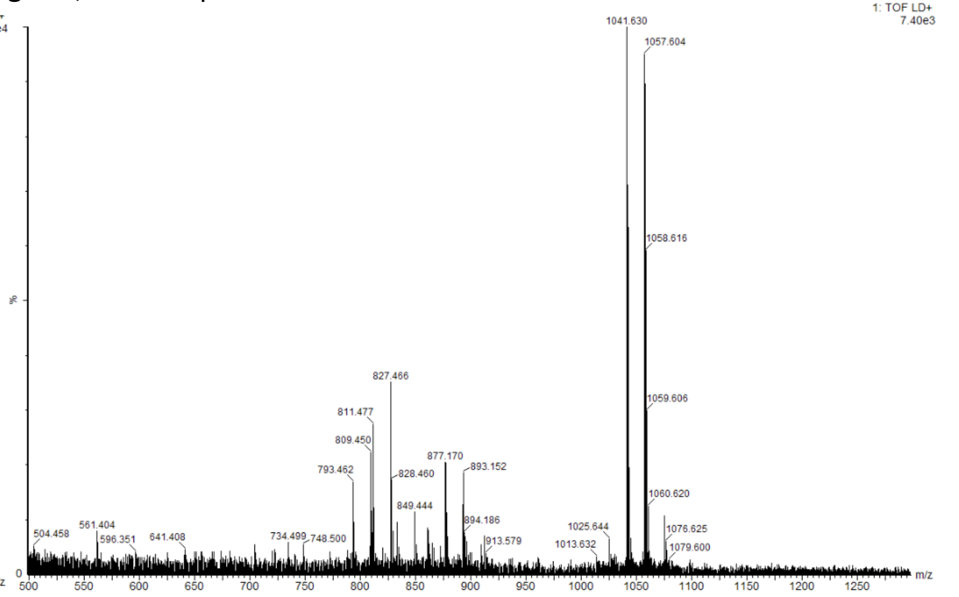

O

gtXX , *Anabaena* sp. SKSF1 cells

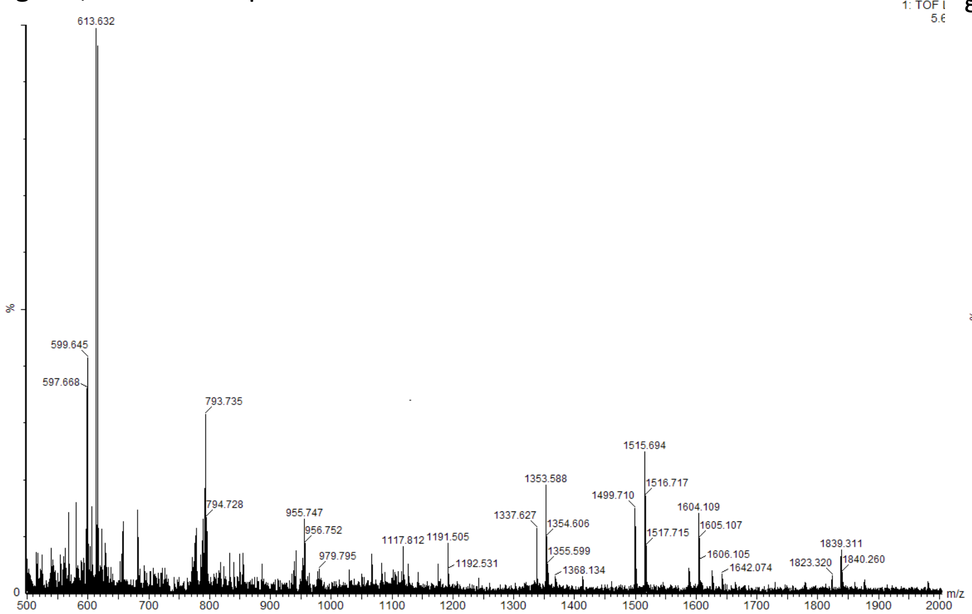

gtXX, *Anabaena* sp. SKSF1 medium

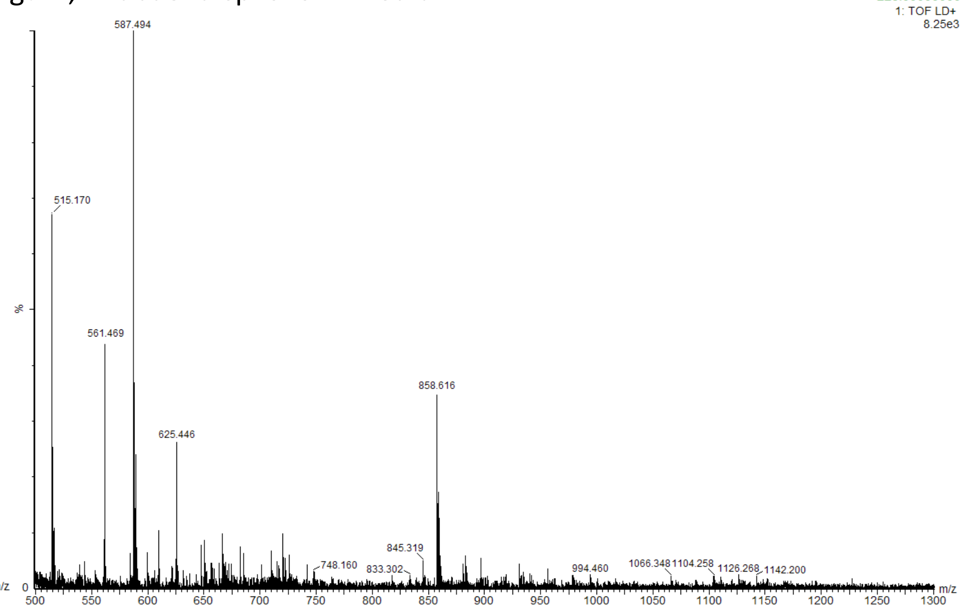

P

gtXVIII , *Nostoc* sp. SKS8 cells

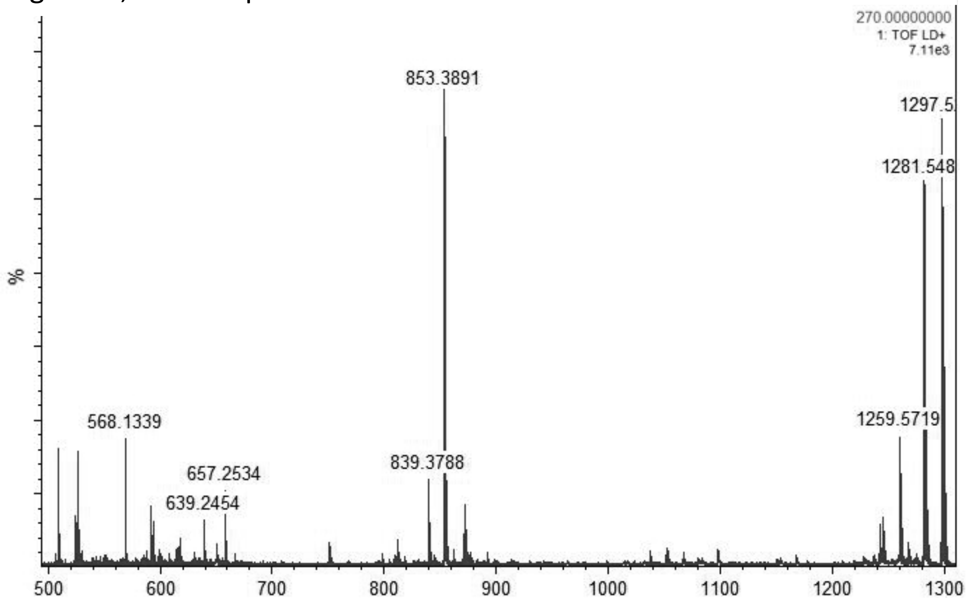

Q

gtXXI , *Nostoc* sp. SKSF3 cells

gtXXI, *Nostoc* sp. SKSF3 medium

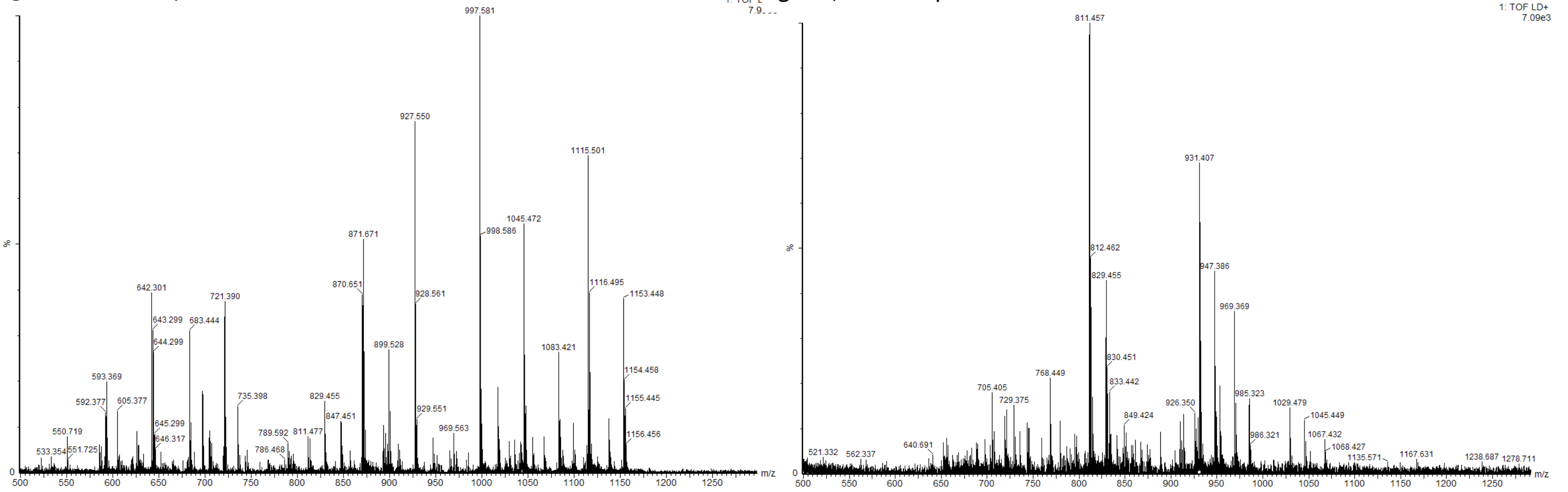

R

gtXXIII , *Nostoc* sp. SKJ2 cells

gtXXIII, *Nostoc* sp. SKJ2 medium

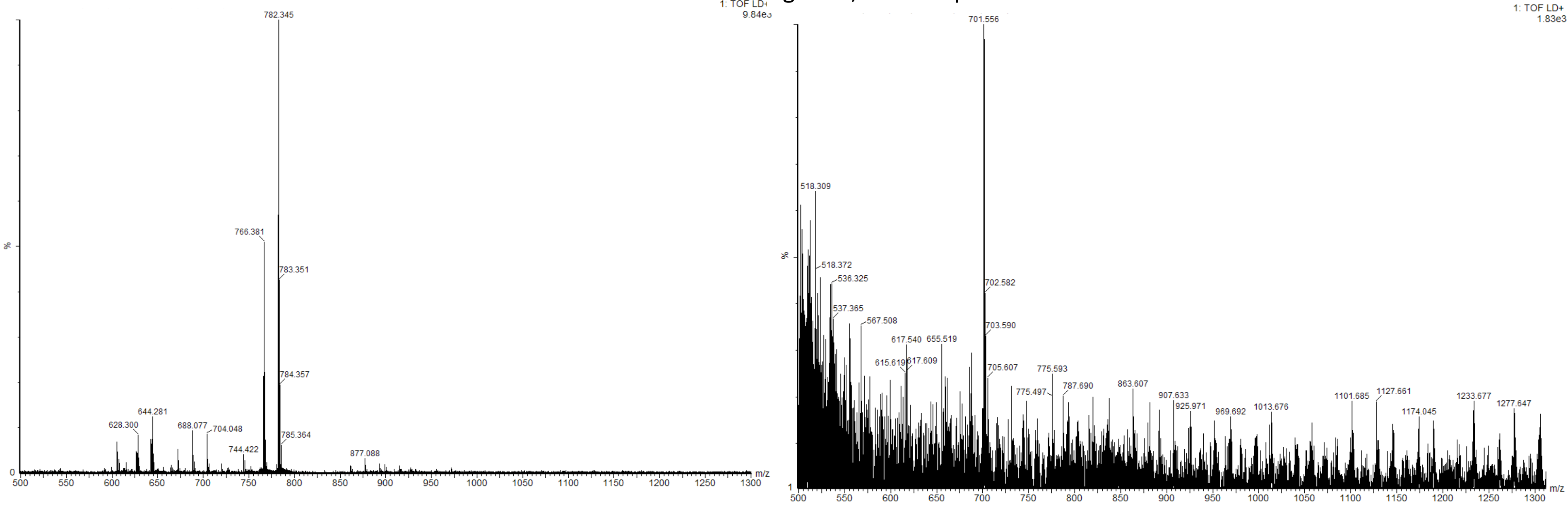

R

gtXXIV , *Nostoc* sp. SKJ4 cells

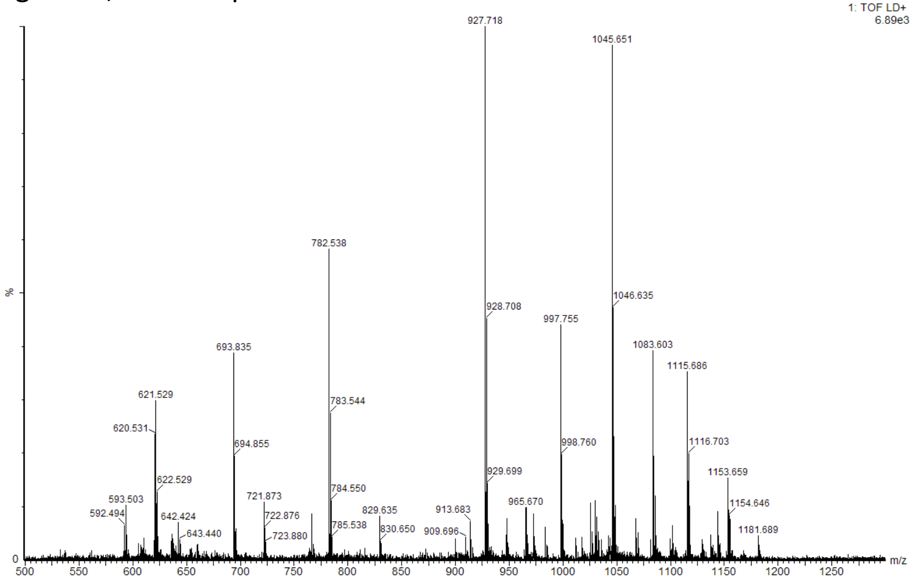

gtXXIV, *Nostoc* sp. SKJ4 medium

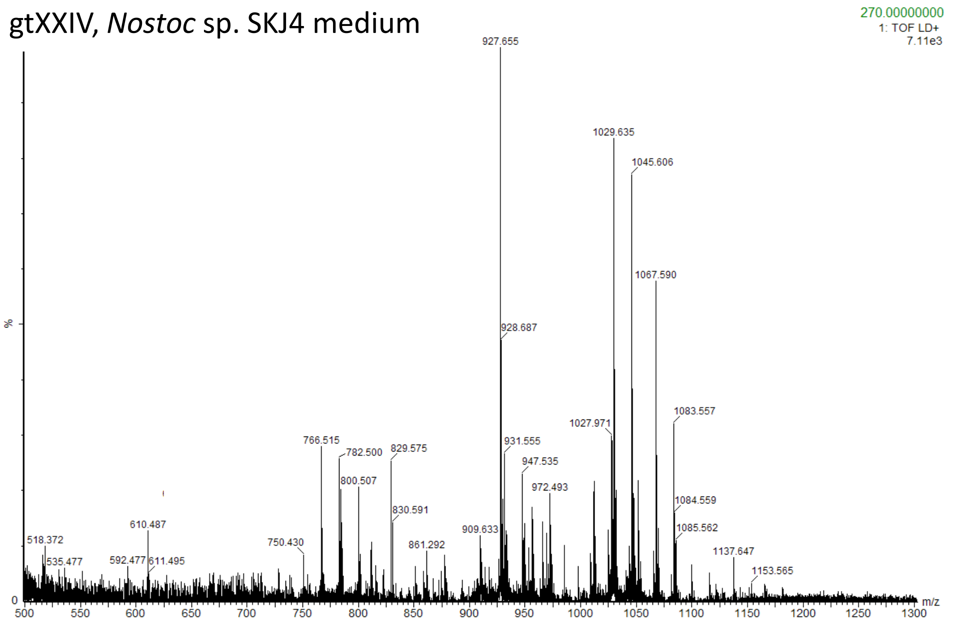

S

gtXXV , *Nostoc* sp. SKJ6 cells

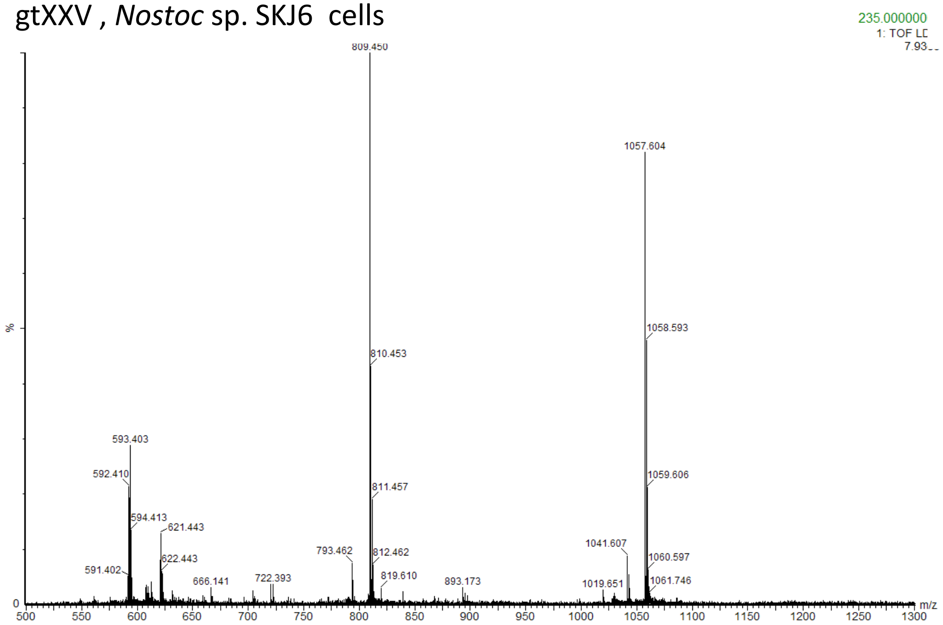

gtXXV, *Nostoc* sp. SKJ6 medium

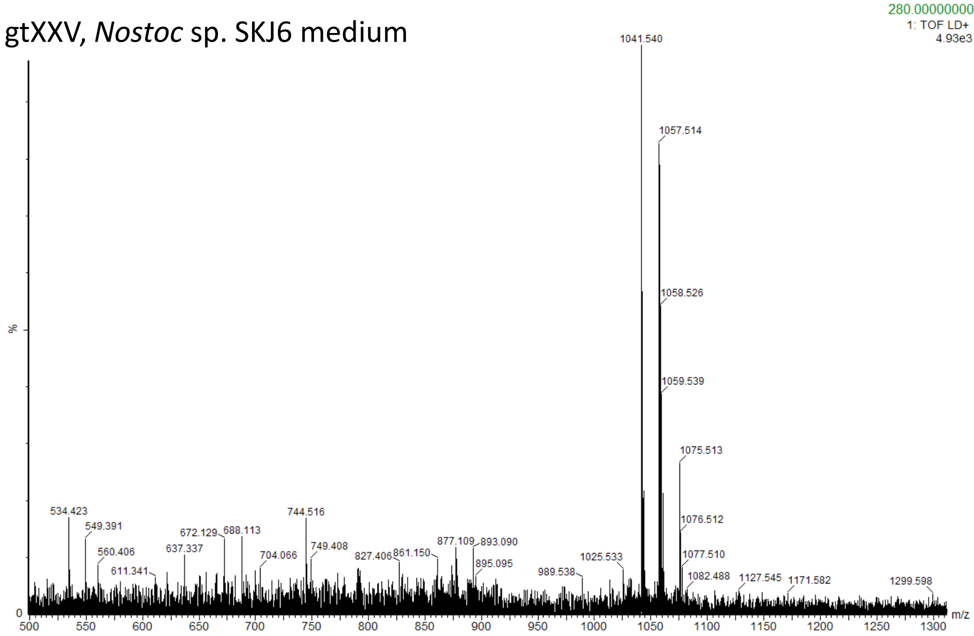

Supplement: Supplementary file 3 [file Image_2.PDF]
