# Supplementary material for: A Genetic and Chemical Perspective on Symbiotic Recruitment of Cyanobacteria of the Genus Nostoc into the Host Plant Blasia pusilla L
Source: Front Microbiol. 2016 Nov 1;7:1693. doi: 10.3389/fmicb.2016.01693 (PMC5088731; doi:10.3389/fmicb.2016.01693)
Supplement: Supplementary file 4 [file Image_3.PDF]

Fig S3.
Examples of fragmentation profiles(MS/MS) of identified compounds recorded in this study obtained by Q-TOF analyses. Aeruginosins-A-D, Nosperin-E, Nostocyclopeptide a1-F, Microcystins-G-H, Nodularins-I-J, Nostophycin-K, Suomilide/Banyaside-like-L. The ion matching reference/diagnostic ions of known compounds are marked by *.

A

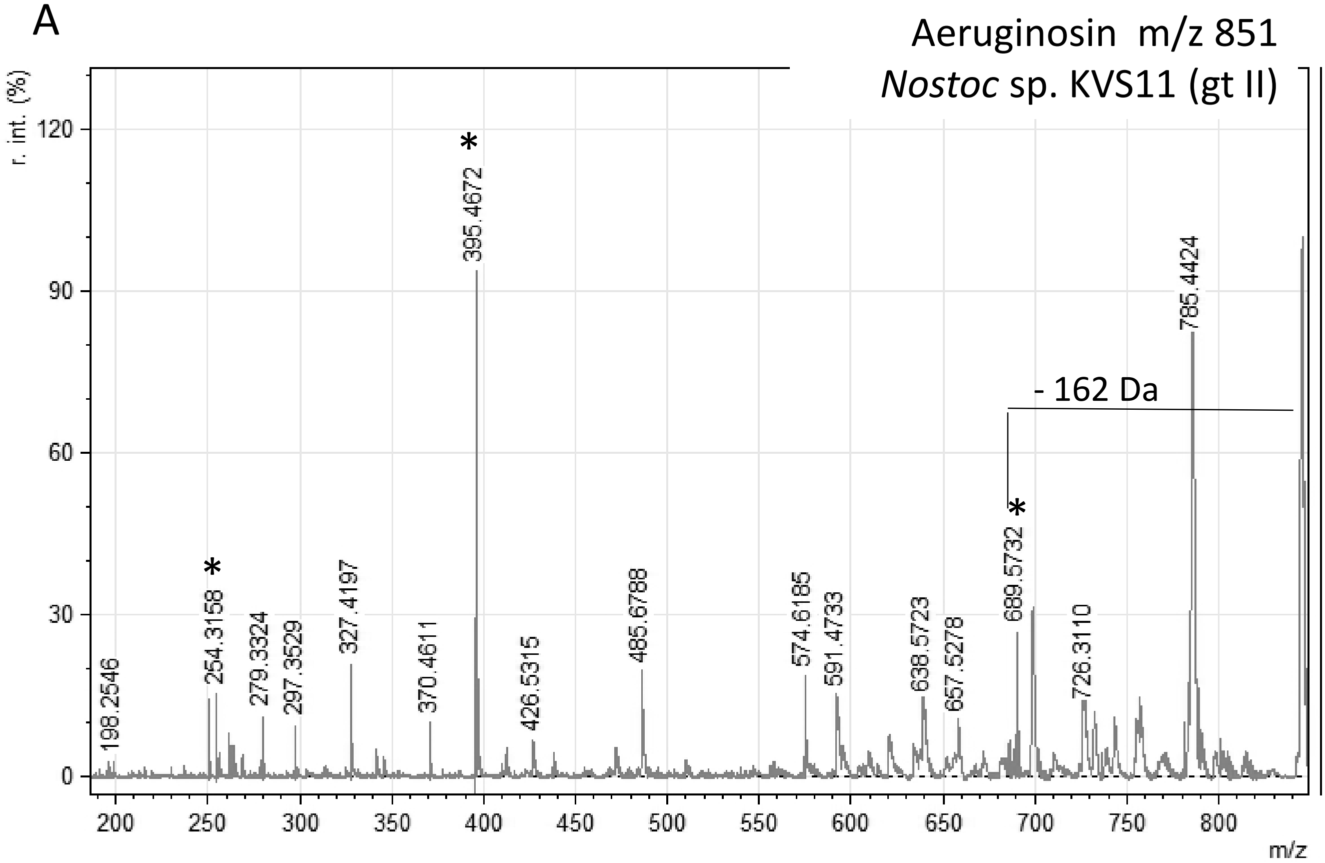

B

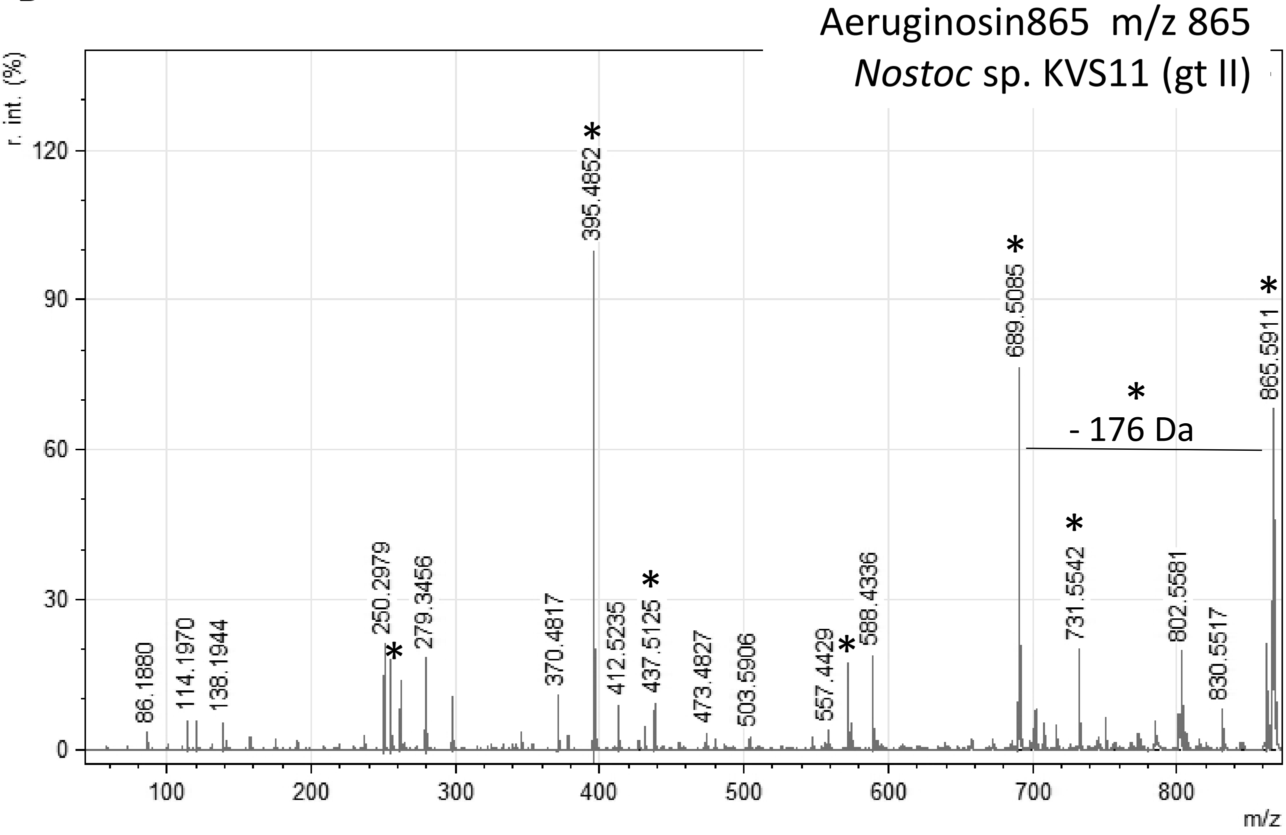

Reference Aeruginosin865 in Kapuscik et al., 2013

C

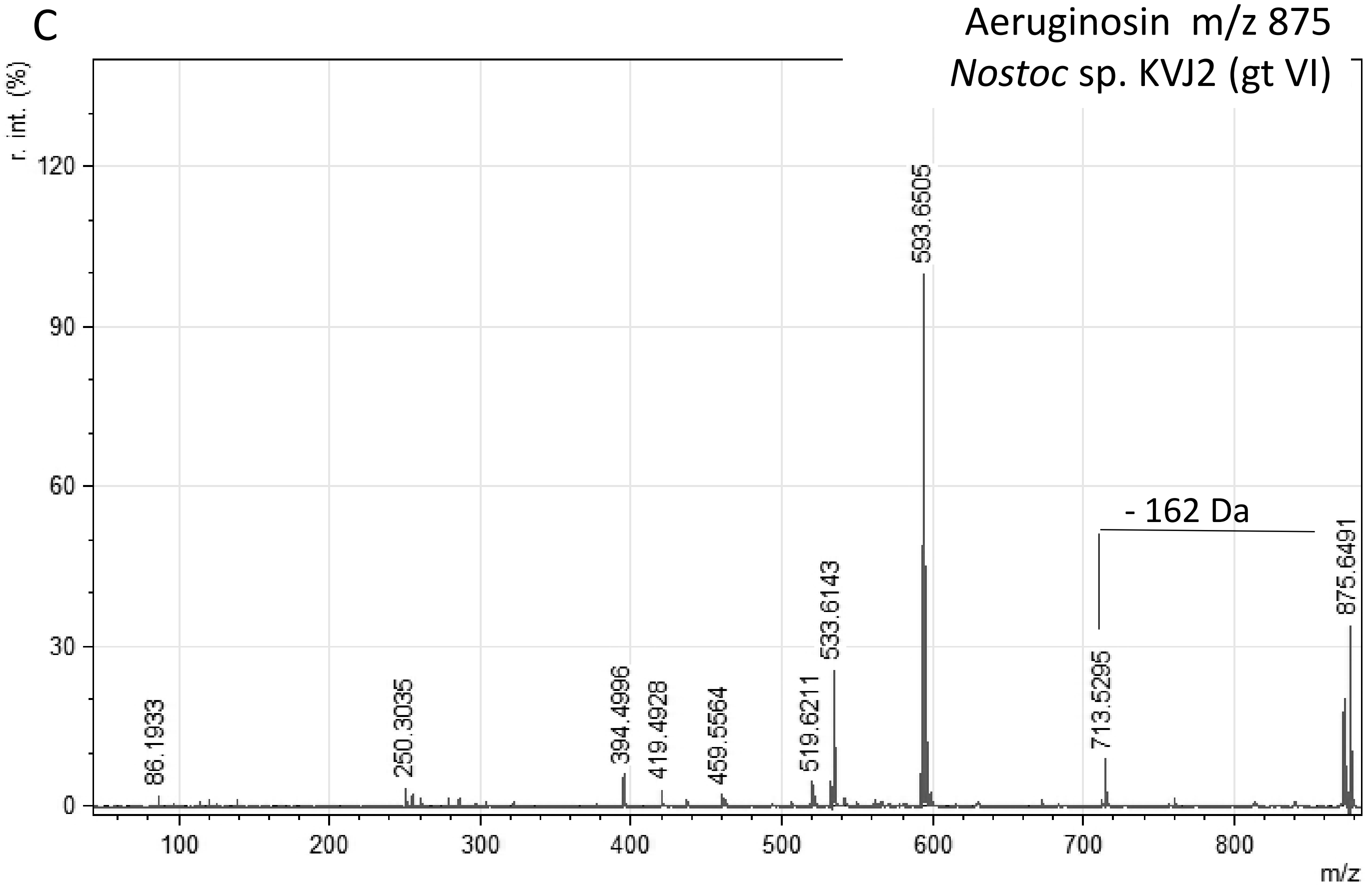

Aeruginosin m/z 875
Nostoc sp. KVJ2 (gt VI)
r. int. (%)
120
90
60
30
0
593.6505
- 162 Da
875.6491
533.6143
713.5295
394.4996
250.3035
419.4928
459.5564
519.6211
86.1933
100
200
300
400
500
600
700
800
m/z

D

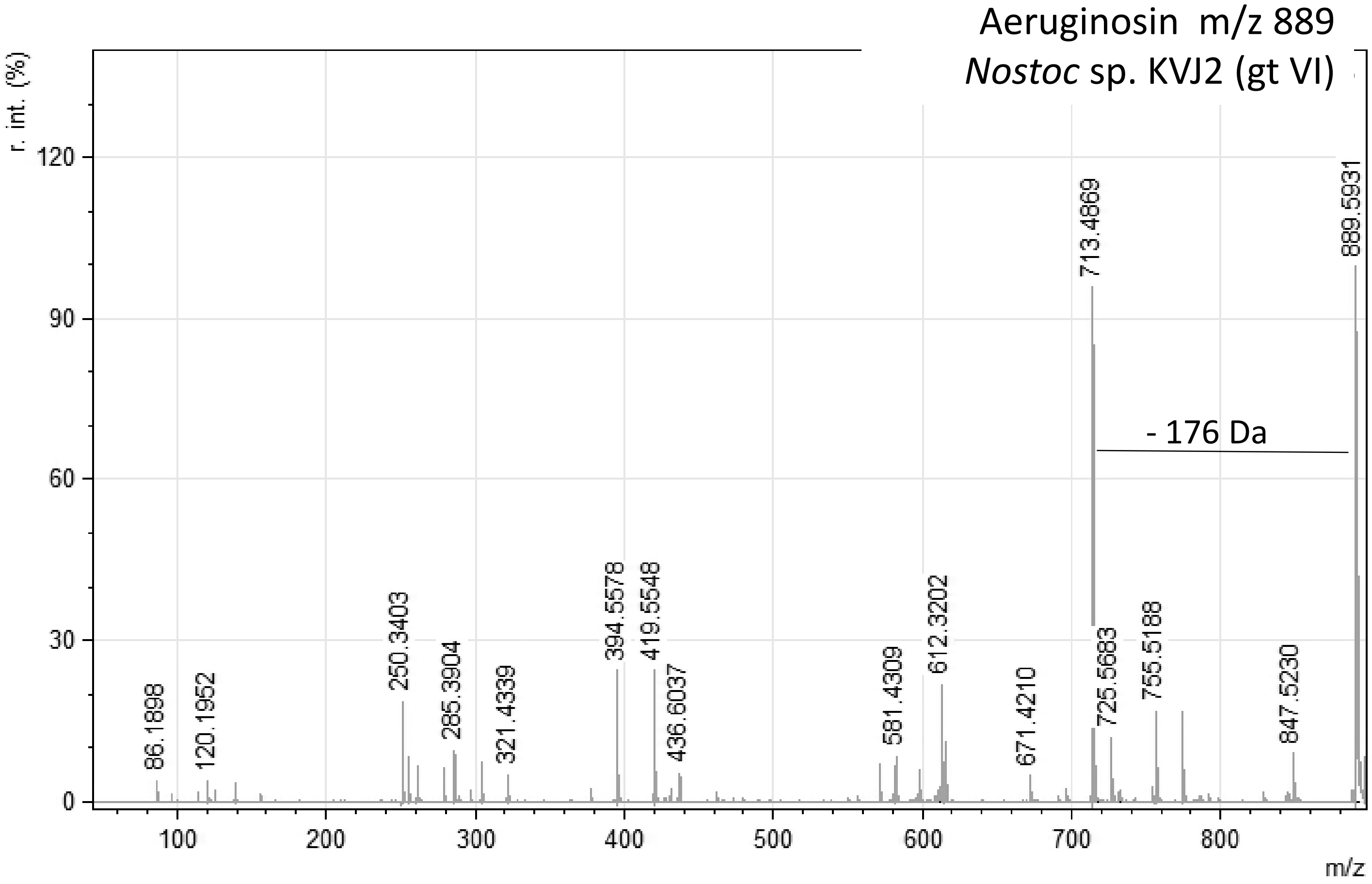

Aeruginosin m/z 889
Nostoc sp. KVJ2 (gt VI)
r. int. (%)
120
90
60
30
0
889.5931
713.4869
- 176 Da
250.3403
394.5578
419.5548
612.3202
755.5188
725.5683
285.3904
321.4339
436.6037
581.4309
671.4210
847.5230
86.1898
120.1952
100
200
300
400
500
600
700
800
m/z

E

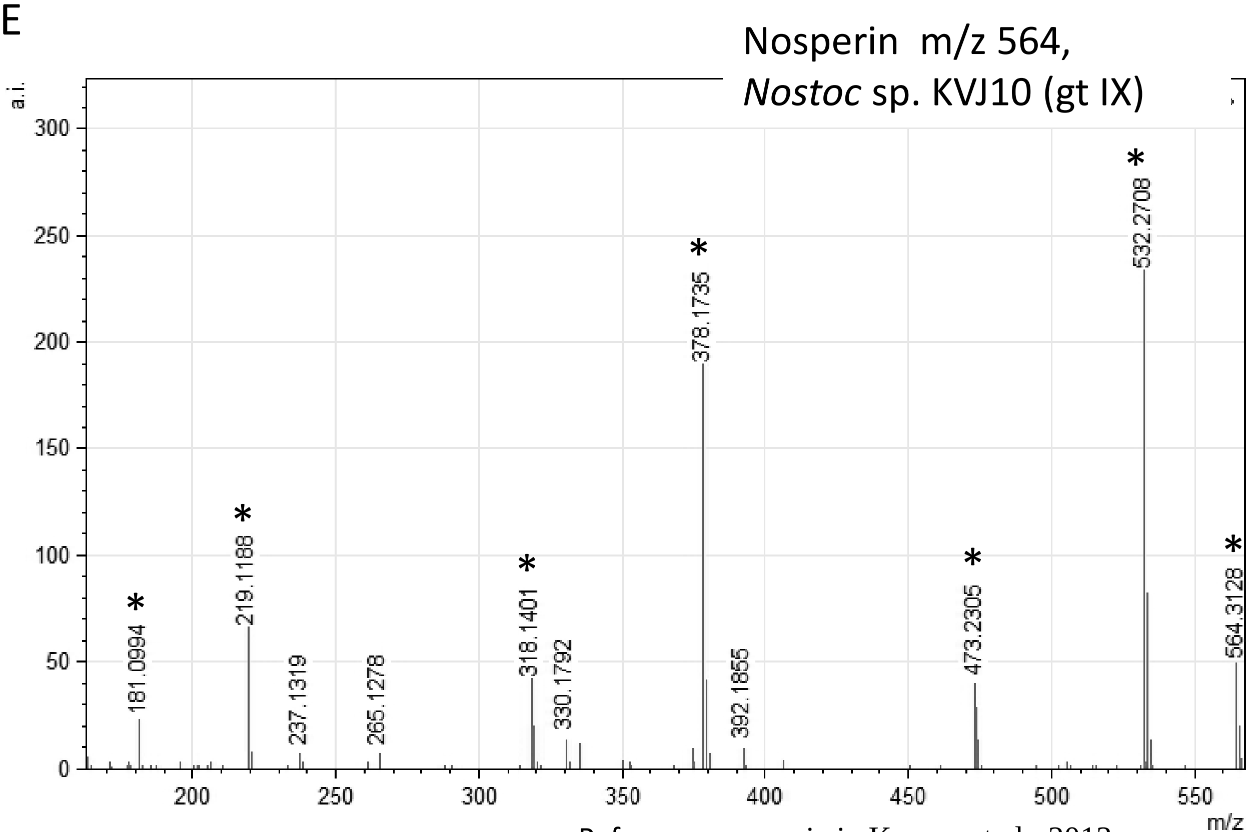

Reference nosperin in Kampa et al., 2013

F

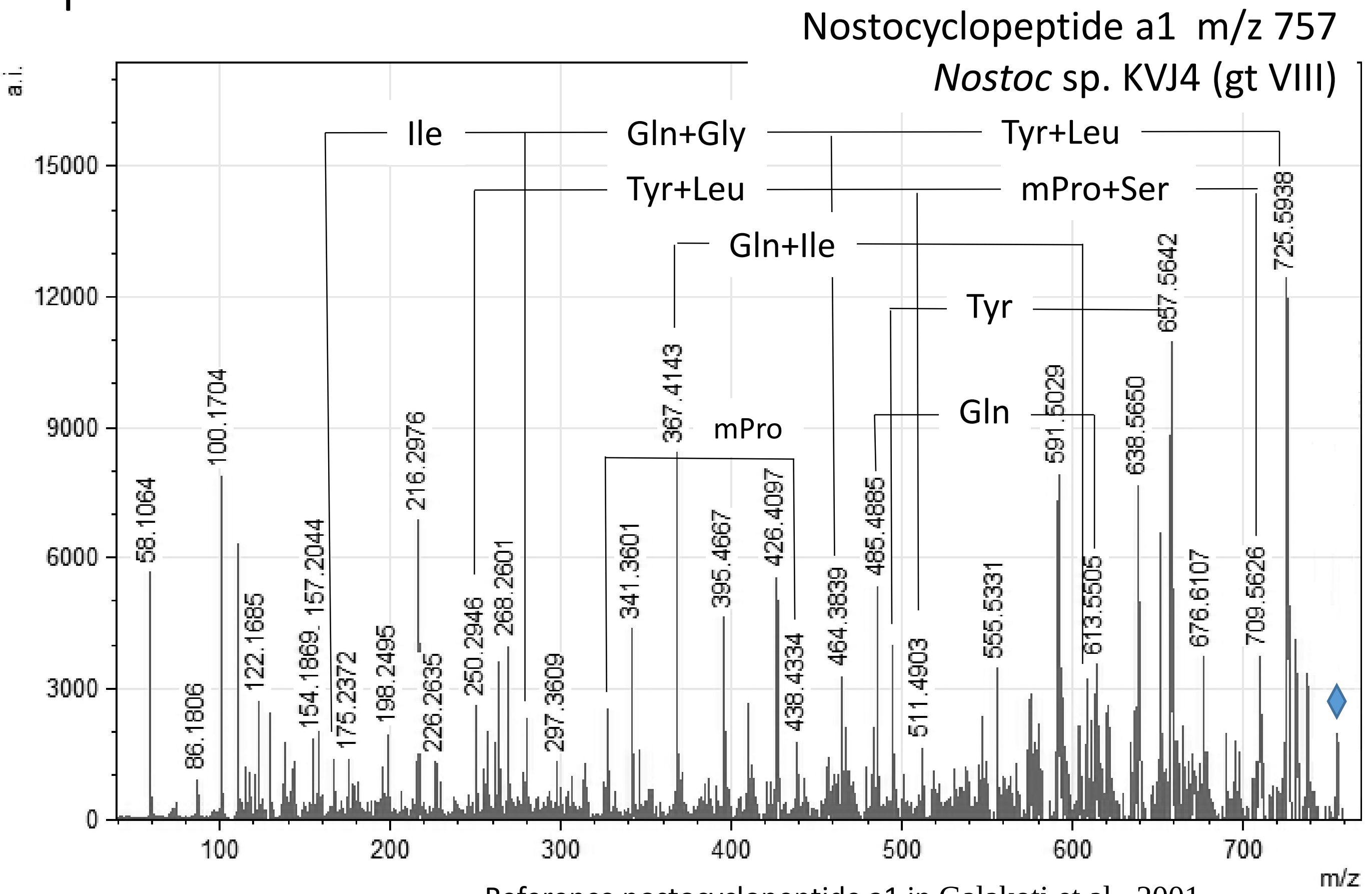

Reference nostocyclopeptide a1 in Galakoti et al., 2001

G

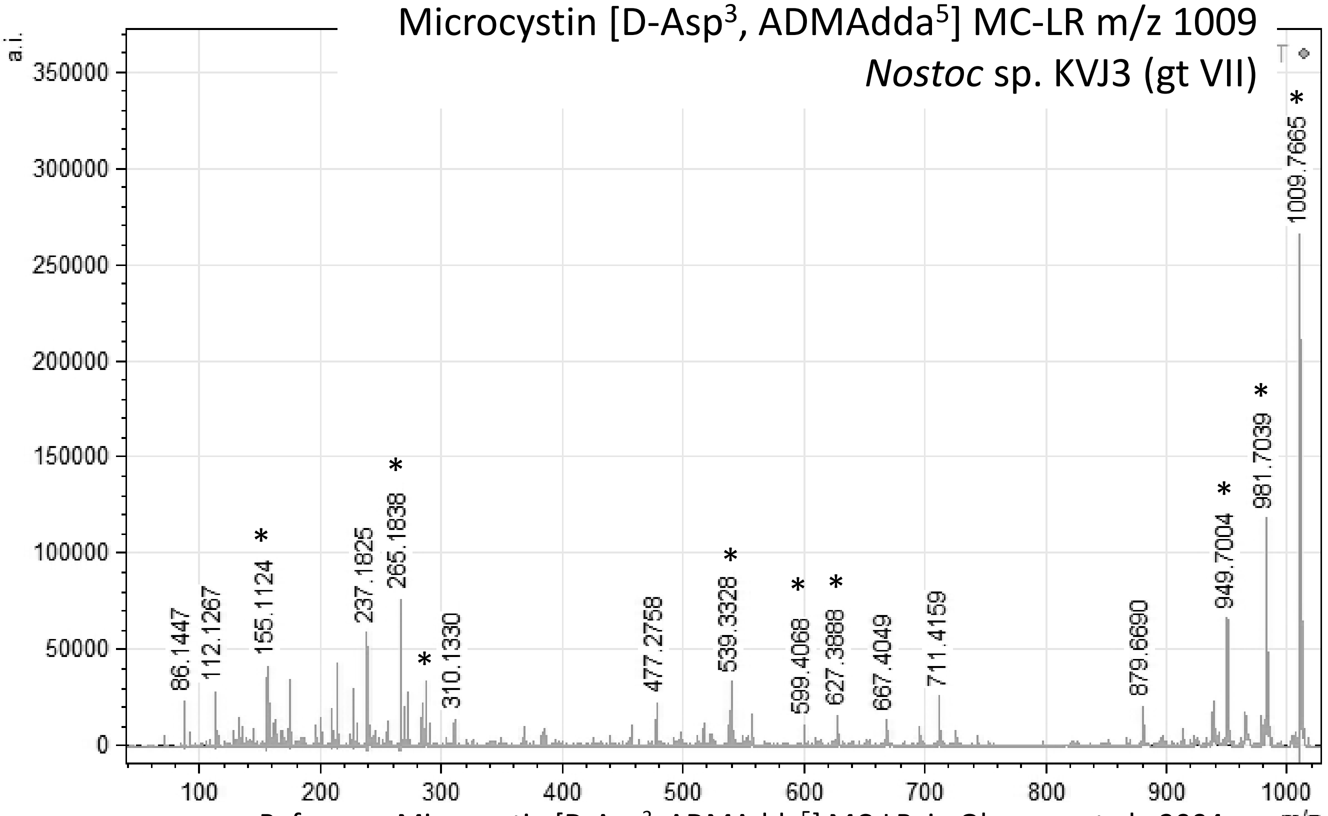

Reference Microcystin [D-Asp^{3}, ADMAdda^{5}] MC-LR in Oksanen et al., 2004

H

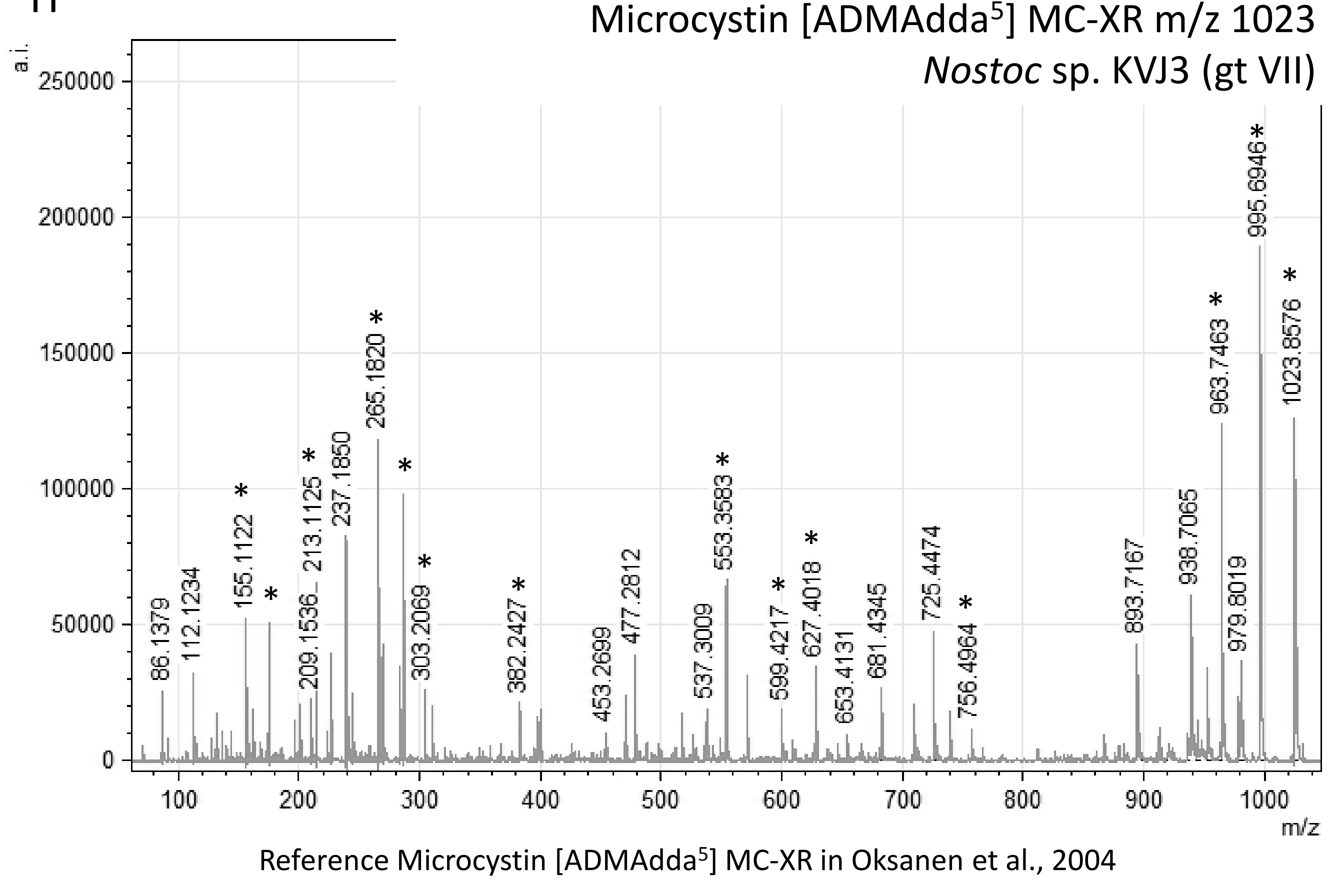

Reference Microcystin [ADMAdda^{5}] MC-XR in Oksanen et al., 2004

I

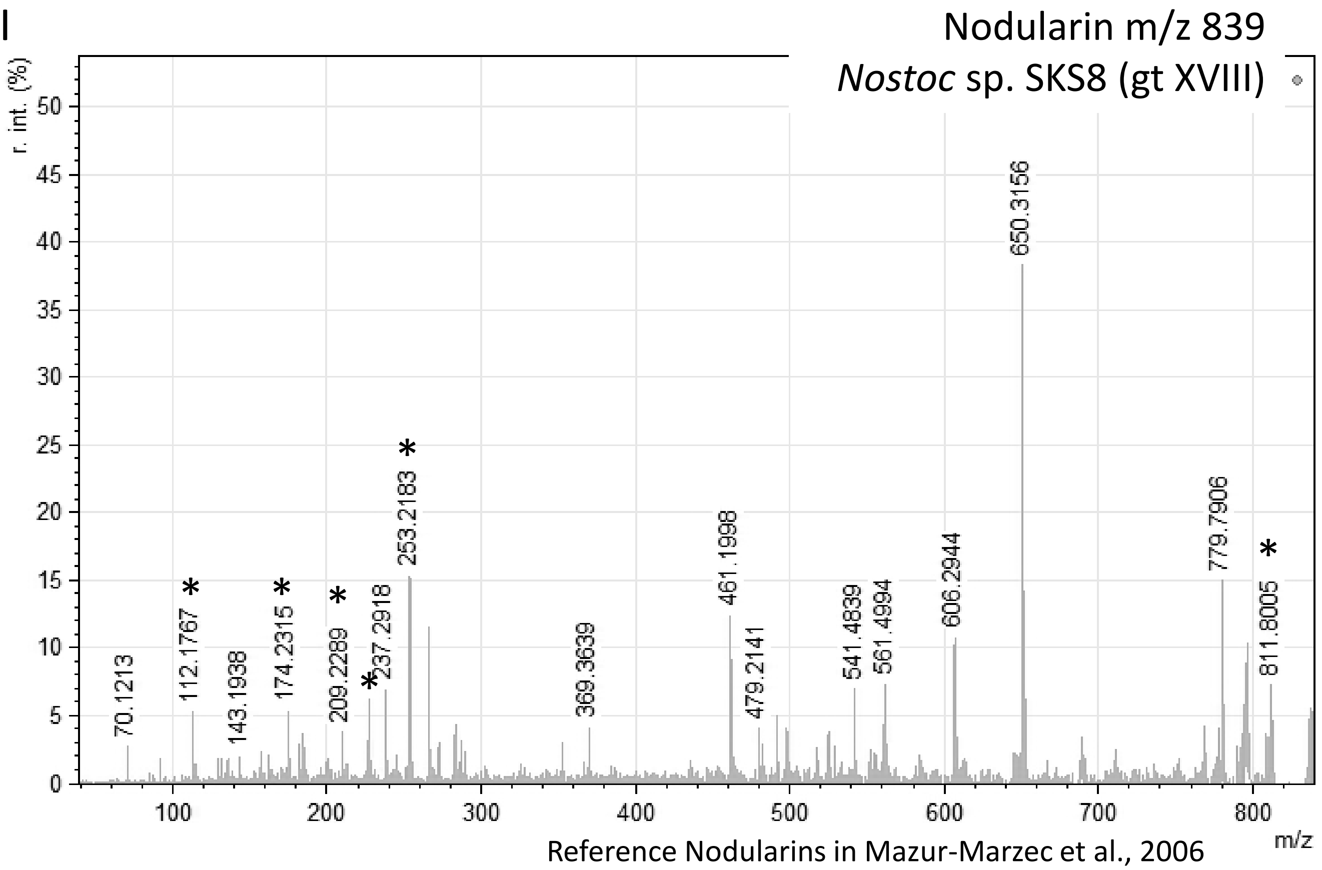
Nodularin m/z 839
Nostoc sp. SKS8 (gt XVIII)
r. int. (%)
70.1213
112.1767
143.1938
174.2315
209.2289
237.2918
253.2183
369.3639
461.1998
479.2141
541.4839
561.4994
606.2944
650.3156
779.7906
811.8005
m/z
Reference Nodularins in Mazur-Marzec et al., 2006

J

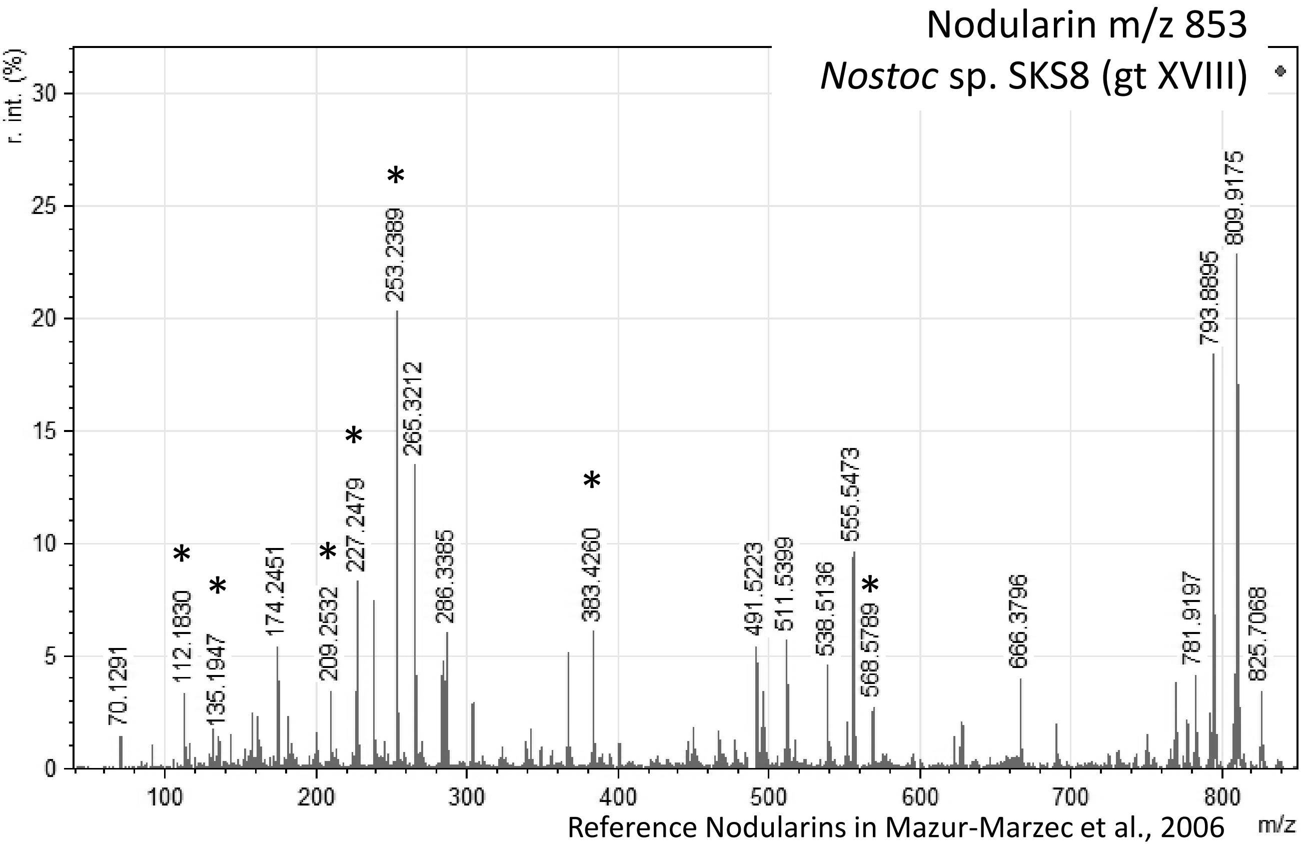
Nodularin m/z 853
Nostoc sp. SKS8 (gt XVIII)
r. int. (%)
70.1291
112.1830
135.1947
174.2451
209.2532
227.2479
253.2389
265.3212
286.3385
383.4260
491.5223
511.5399
538.5136
555.5473
568.5789
666.3796
781.9197
793.8895
809.9175
825.7068
m/z
Reference Nodularins in Mazur-Marzec et al., 2006

K

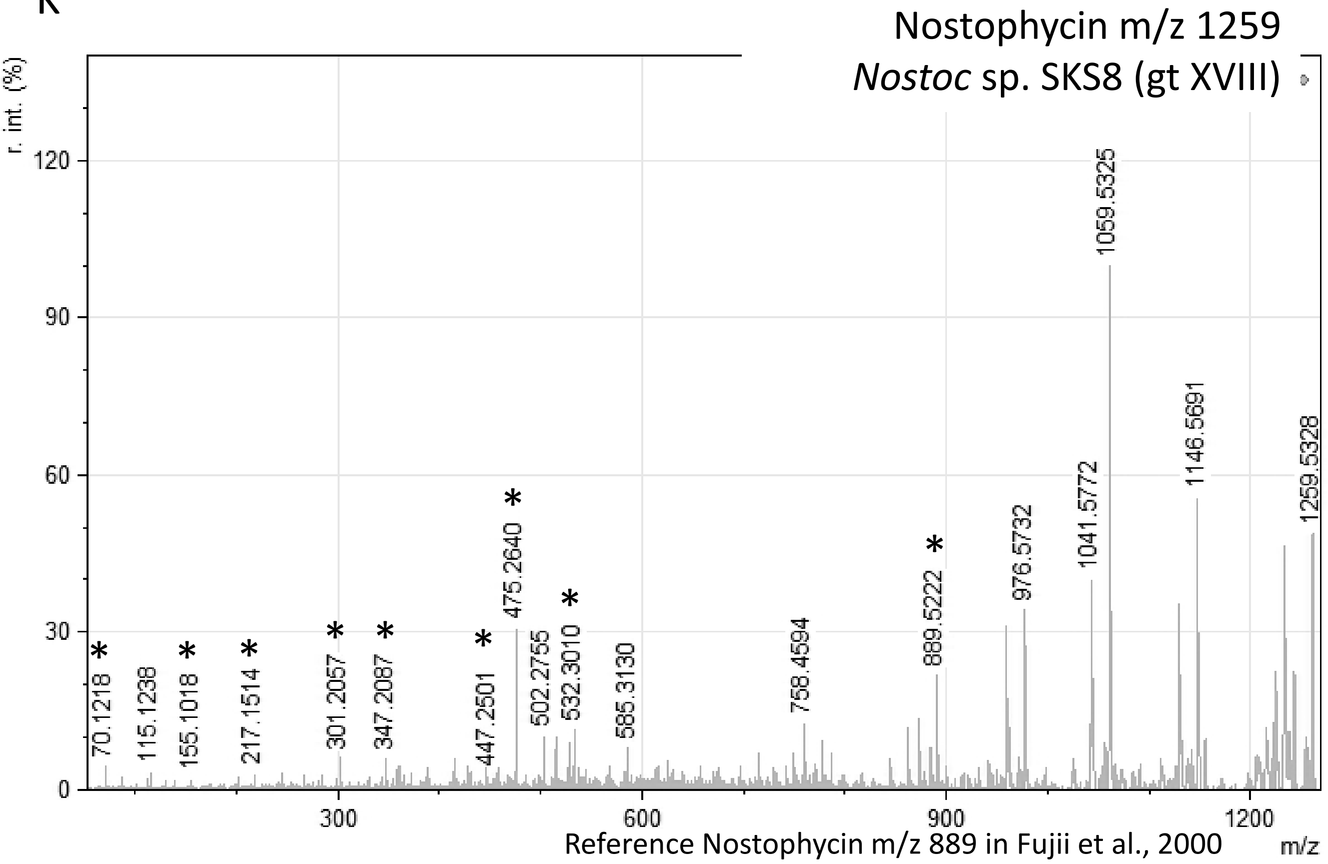
Nostophycin m/z 1259
Nostoc sp. SKS8 (gt XVIII)
r. int. (%)
0
30
60
90
120
300
600
900
1200
m/z
70.1218 *
115.1238
155.1018 *
217.1514 *
301.2057 *
347.2087 *
447.2501 *
475.2640 *
502.2755
532.3010 *
585.3130
758.4594
889.5222 *
976.5732
1041.5772
1059.5325
1146.5691
1259.5328
Reference Nostophycin m/z 889 in Fujii et al., 2000

L

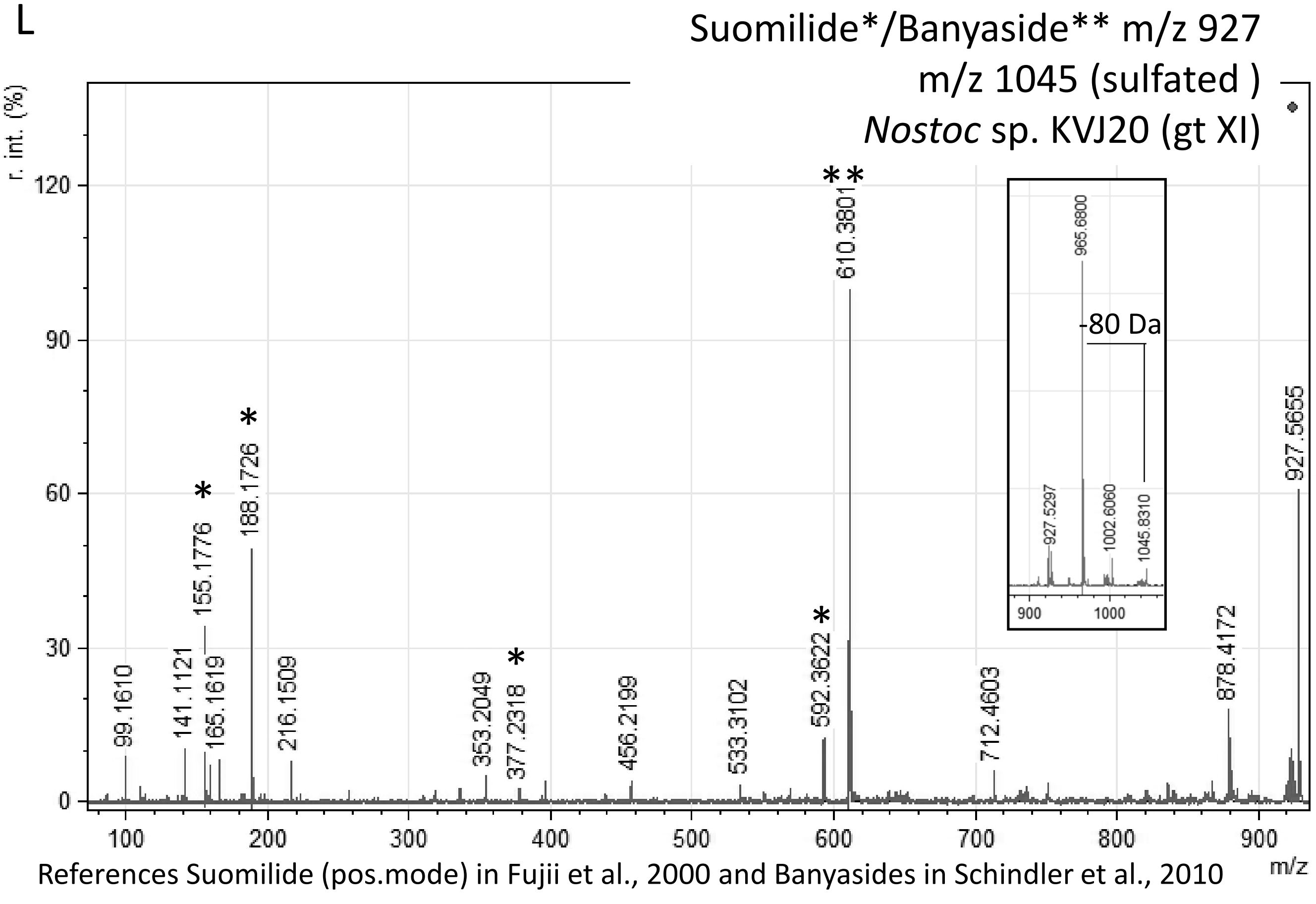
Suomilide*/Banyaside** m/z 927
m/z 1045 (sulfated)
Nostoc sp. KVJ20 (gt XI)
r. int. (%)
0
30
60
90
120
100
200
300
400
500
600
700
800
900
m/z
99.1610
141.1121
155.1776 *
165.1619
188.1726 *
216.1509
353.2049
377.2318 *
456.2199
533.3102
592.3622 *
610.3801 **
712.4603
878.4172
927.5655
965.6800
-80 Da
927.5297
1002.6060
1045.8310
900
1000
References Suomilide (pos.mode) in Fujii et al., 2000 and Banyasides in Schindler et al., 2010
