# Supplementary figures and images for: A Genetic and Chemical Perspective on Symbiotic Recruitment of Cyanobacteria of the Genus Nostoc into the Host Plant Blasia pusilla L
Source: Front Microbiol. 2016 Nov 1;7:1693. doi: 10.3389/fmicb.2016.01693 (PMC5088731; doi:10.3389/fmicb.2016.01693)

Figure S4. An overview of the workflow implemented in this study

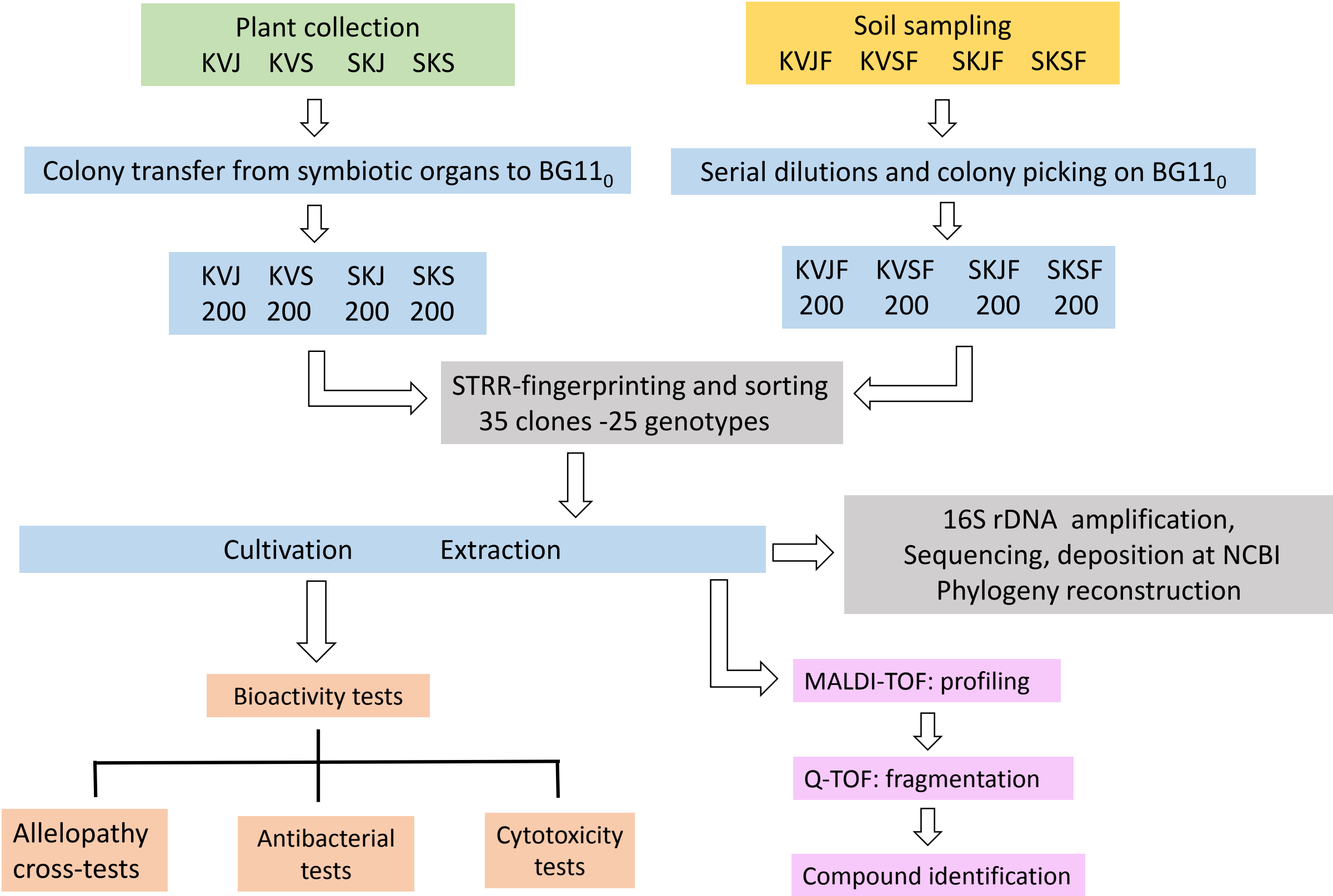

Supplement: Supplementary file 5 [file Image_4.PDF]
