# Supplementary material for: A Genetic and Chemical Perspective on Symbiotic Recruitment of Cyanobacteria of the Genus Nostoc into the Host Plant Blasia pusilla L
Source: Front Microbiol. 2016 Nov 1;7:1693. doi: 10.3389/fmicb.2016.01693 (PMC5088731; doi:10.3389/fmicb.2016.01693)
Supplement: Supplementary file 6 [file Image_5.PDF]

Figure S5. ClustalW alignement of 16S rDNA sequnces used for the phylogeny reconstruction. For the detailes on sequnces' source see Table S1. The phylogenetic tree is presented in Figure 3.

```
                    10        20        30        40        50        60        70        80        90       100
            ....|....|....|....|....|....|....|....|....|....|....|....|....|....|....|....|....|....|....|....|
KVJ10       -------AGAGTTTGATCCTGGCTCAGGATGAACGCTGGCGGTATGCTTAACACATGCAAGTCGAACGGTGT--CTTC--GGACACAGTGGCGGACGGGT
KVJF8       -------AGAGTTTGATCCTGGCTCAGGATGAACGCTGGCGGTATGCTTAACACATGCAAGTCGAACGGTGT--CTTC--GGACACAGTGGCGGACGGGT
KVJ3        -------AGAGTTTGATCCTGGCTCAGGATGAACGCTGGCGGTATGCTTAACACATGCAAGTCGAACGGTGC--TTTC--GGGCACAGTGGCGGACGGGT
SKS1        -------AGAGTTTGATCCTGGCTCAGGATGAACGCTGGCGGTATGCTTAACACATGCAAGTCGAACGGTGC--TTTC--GGGCACAGTGGCGGACGGGT
KVS1        -------AGAGTTTGATCCTGGCTCAGGATGAACGCTGGCGGTATGCTTAACACATGCAAGTCGAACGGTGC--TTTC--GGGCACAGTGGCGGACGGGT
SKJ1        -------AGAGTTTGATCCTGGCTCAGGATGAACGCTGGCGGTATGCTTAACACATGCAAGTCGAACGGTGC--TTTC--GGGCACAGTGGCGGACGGGT
KVJF4       -------AGAGTTTGATCCTGGCTCAGGATGAACGCTGGCGGTATGCTTAACACATGCAAGTCGAACGGTGT--CTTC--GGACACAGTGGCGGACGGGT
KVS11       -------AGAGTTTGATCCTGGCTCAGGATGAACGCTGGCGGTATGCTTAACACATGCAAGTCGAACGGTGT--CTTC--GGACACAGTGGCGGACGGGT
KVJ20       -------AGAGTTTGATCCTGGCTCAGGATGAACGCTGGCGGTATGCTTAACACATGCAAGTCGAAATAAAT--CTTC--GGATTTAGTGGCGGACGGGT
SKSF3       -------AGAGTTTGATCCTGGCTCAGGATGAACGCTGGCGGTATGCTTAACACATGCAAGTCGAACGGTAT--CTTG--GGATTTAGTGGCGGACGGGT
SKSL1       -------AGAGTTTGATCCTGGCTCAGGATGAACGCTGGCGGTATGCTTAACACATGCAAGTCGAACGGTGC--TTTC--GGGCACAGTGGCGGACGGGT
SKSL2       -------AGAGTTTGATCCTGGCTCAGGATGAACGCTGGCGGTATGCTTAACACATGCAAGTCGAACGGTGC--TTTC--GGGCACAGTGGCGGACGGGT
KVJ4        -------AGAGTTTGATCCTGGCTCAGGATGAACGCTGGCGGTATGCTTAACACATGCAAGTCGAACGGTGT--CTTC--GGACACAGTGGCGGACGGGT
KVJ2        -------AGAGTTTGATCCTGGCTCAGGATGAACGCTGGCGGTATGCTTAACACATGCAAGTCGAACGGTGT--CTTC--GGACACAGTGGCGGACGGGT
KVSF4       -------AGAGTTTGATCCTGGCTCAGGATGAACGCTGGCGGTATGCTTAACACATGCAAGTCGAACGGTGT--CTTC--GGACACAGTGGCGGACGGGT
SKS2        -------AGAGTTTGATCCTGGCTCAGGATGAACGCTGGCGGTATGCTTAACACATGCAAGTCGAACGGTGT--CTTC--GGACACAGTGGCGGACGGGT
SKJF1       -------AGAGTTTGATCCTGGCTCAGGATGAACGCTGGCGGTATGCTTAACACATGCAAGTCGAACGGAAT--CTTC--GGATTTAGTGGCGGACGGGT
SKS3        -------AGAGTTTGATCCTGGCTCAGGATGAACGCTGGCGGTATGCTTAACACATGCAAGTCGAACGGAAT--CTTC--GGATTTAGTGGCGGACGGGT
SKJ6        -------AGAGTTTGATCCTGGCTCAGGATGAACGCTGGCGGTATGCTTAACACATGCAAGTCGAACGGAAT--CTTC--GGATTTAGTGGCGGACGGGT
SKJ2        -------AGAGTTTGATCCTGGCTCAGGATGAACGCTGGCGGTATGCTTAACACATGCAAGTCGAACGGAAT--CTTTCGGGATTTAGTGGCGGACGGGT
SKS5        -------AGAGTTTGATCCTGGCTCAGGATGAACGCTGGCGGTATGCTTAACACATGCAAGTCGAACGGAAT--CTTTCGGGATTTAGTGGCGGACGGGT
SKS9        -------AGAGTTTGATCCTGGCTCAGGATGAACGCTGGCGGTATGCTTAACACATGCAAGTCGAACGGAAT--CTTTCGGGATTTAGTGGCGGACGGGT
KVJ18       -------AGAGTTTGATCCTGGCTCAGGATGAACGCTGGCGGTATGCTTAACACATGCAAGTCGAACGAAAT--CTTTCGGGATTTAGTGGCGGACGGGT
SKJ4        -------AGAGTTTGATCCTGGCTCAGGATGAACGCTGGCGGTATGCTTAACACATGCAAGTCGAACGGAAT--CTTC--GGATTTAGTGGCGGACGGGT
SKSF1       -------AGAGTTTGATCCTGGCTCAGGATGAACGCTGGCGGTATGCTTAACACATGCAAGTCGAACGGAGT--ACTTAGGTACTTAGTGGCGGACGGGT
KVSF7       -------AGAGTTTGATCCTGGCTCAGGATGAACGCTGGCGGTATGCTTAACACATGCAAGTCGAACGGAAT--CCTTAGGGATTTAGTGGCGGACGGGT
SKS8        -------AGAGTTTGATCCTGGCTCAGGATGAACGCTGGCGGTATGCTTAACACATGCAAGTCGAACGGAAT--CTTC--GGATTTAGTGGCGGACGGGT
DQ185249    ---------------------GCTCAGGATGAACGCTGGCGGTATGCTTAACACATGCAAGTCGAACGGTGT--CTTC--GGACACAGTGGCGGACGGGT
DQ185255    -----------------------TCAGGATGAACGCTGGCGGTATGCTTAACACATGCAAGTCGAACGGTGT--CTTC--GGACACAGTGGCGGACGGGT
AY566855    ----------------------CTCAGGATGAACGCTGGCGGTATGCTTAACACATGCAAGTCGAACGGTGC--TTTC--GGGCACAGTGGCGGACGGGT
AM711545    ---------------------------GATGAACGCTGGCGGTATGCTTAACACATGCAAGTCGAACGGTGT--CTTC--GGACACAGTGGCGGACGGGT
AM711536    ---------------------------GATGAACGCTGGCGGTATGCTTAACACATGCAAGTCGAACGGTGT--CTTC--GGACACAGTGGCGGACGGGT
DQ185256    --------------------------GGATGAACGCTGGCGGTATGCTTAACACATGCAAGTCGAACGGTGT--CTTC--GGACACAGTGGCGGACGGGT
DQ185245    -------------------------AGGATGAACGCTGGCGGTATGCTTAACACATGCAAGTCGAACGGTGT--CTTC--GGACACAGTGGCGGACGGGT
AM711527    ---------------------------GATGAACGCTGGCGGTATGCTTAACACATGCAAGTCGAACGGTGT--CTTC--GGACACAGTGGCGGACGGGT
DQ185212    -------------------------------AACGCTGGCGGTATGCTTAACACATGCAAGTCGAACGGTGT--CTTC--GGACACAGTGGCGGACGGGT
AF062638    -------AGAGTTTGATCCTGGCTCAGGATGAACGCTGGCGGTATGCTTAACACATGCAAGTCGAACGGTGT--CTTC--GGACACAGTGGCGGACGGGT
HM623781    -------AGAGTTTGATCCTGGCTCAGGATGAACGCTGGCGGTATGCTTAACACATGCAAGTCGAACGAAAT--CTTC--GGATTTAGTGGCGGACGGGT
AM711540    ---------------------------GATGAACGCTGGCGGTATGCTTAACACATGCAAGTCGAACGAAAT--CTTC--GGATTTAGTGGCGGACGGGT
```

```
DQ185254     --------------------------GGATGAACGCTGGCGGTATGCTTAACACATGCAAGTCGAACGAAAT--CTTTCGGGATTTAGTGGCGGACGGGT
DQ185251     ----------------------CTCAGGATGAACGCTGGCGGTATGCTTAACACATGCAAGTCGAACGAAAT--CTTC--GGATTTAGTGGCGGACGGGT
AM711535     ---------------------------GATGAACGCTGGCGGTATGCTTAACACATGCAAGTCGAACGAAAT--CTTC--GGATTTAGTGGCGGACGGGT
AF027653     ---------------------------GATGAACGCTGGCGGTATGCTTAACACATGCAAGTCGAACGGTGC--TTTC--GGGCACAGTGGCGGACGGGT
DQ185244     ---------------------------------CGCTGGCGGTATGCTTAACACATGCAAGTCGAACGGTGC--TTTC--GGGCACAGTGGCGGACGGGT
AM711531     ---------------------------GATGAACGCTGGCGGTATGCTTAACACATGCAAGTCGAACGGTGT--CTTC--GGACACAGTGGCGGACGGGT
AJ630449     ---------------------------GATGAACGCTGGCGGTATGCTTAACACATGCAAGTCGAACGGTGT--CTTC--GGACACAGTGGCGGACGGGT
AY328897     ---------------------------------------------------------------GAACGGAAT--CTTC--GGATTTAGTGGCGGACGGGT
AM230702     ---------------------GCTCAGGATGAACGCTGGCGGTATGCTTAACACATGCAAGTCGAACGGTCT--CTTCGGAGA--TAGTGGCGGACGGGT
EF568907     -------AGAGTTTGATCCTGGCTCAGGATGAACGCTGGCGGTATGCTTAACACATGCAAGTCGAACGGTCT--TTTCGGAGA--TAGTGGCGGACGGGT
AJ630458     ---------------------------GATGAACGCTGGCGGTATGCTTAACACATGCAAGTCGAACGGAAT--CTTTAGGGATTTAGTGGCGGACGGGT
GQ443447     ---------------------------GAT-AACGCTGGCGGTATGCTTAACACATGCAAGTCGAACGGAAT--CCTTAGGGATTTAGTGGCGGACGGGT
AJ630457     ---------------------------GATGAACGCTGGCGGTATGCTTAACACATGCAAGTCGAACGGAAT--CCTTAGGGATTTAGTGGCGGACGGGT
DQ234831     -------AGAGTTTGATCATGGCTCAGGATGAACGCTGGCGGTATGCTTAACACATGCAAGTCGAACGGAAT--CTTTAGGGATTTAGTGGCGGACGGGT
AJ133161     ------------------------CAGGATGAACGCTGGCGGTATGCTTAACACATGCAAGTCGAACGGAAT--CTTC--GGATTTAGTGGCGGACGGGT
gi|47118302  AAAACGGAGAGTTTGATCCTGGCTCAGGATGAACGCTGGCGGTATGCTTAACACATGCAAGTCGAACGGTCT--CTTC--GGAGATAGTGGCGGACGGGT
DQ185209     ------------------------------GAACGCTGGCGGTATGCTTAACACATGCAAGTCGAACGGTGT--CTTC--GGACACAGTGGCGGACGGGT
AY742451     ---------------ATCCTGGCTCAGGATGAACGCTGGCGGTATGCTTAACACATGCAAGTCGAACGAAAT--CTTC--GGATTTAGTGGCGGACGGGT
AF027655     ---------------------------GATGAACGCTGGCGGTATGCTTAACACATGCAAGTCGAACGGTGT--CTTC--GGACACAGTGGCGGACGGGT
DQ185211     --------------------------------------------TGCTTAACACATGCAAGTCGAACGGTGT--CTTC--GGACACAGTGGCGGACGGGT
AY742449     ---------------ATCCTGGCTCAGGATGAACGCTGGCGGTATGCTTAACACATGCAAGTCGAACGGTGT--CTTC--GGACACAGTGGCGGACGGGT
EF174212     -------AGAGTTTGATCCTGGCTCAGGATGAACGCTGGCGGTATGCTTAACACATGCAAGTCGAACGGTGT--CTTC--GGACACAGTGGCGGACGGGT
DQ185213     ---------------------------------CGCTGGCGGTATGCTTAACACATGCAAGTCGAACGGTGT--CTTC--GGACACAGTGGCGGACGGGT
AF506237     ----------------------------------------------------------------------------------AGATAGTGGCGGACGGGT
AM230690     -------------------------------------------------TTACCATGCAAGTCGACGGGCAC---TTTAGGGTGTTAGTGGCGGACGGGT
AB039002     ---------------------------GATGAACGCTGGCGGTATGCTTAACACATGCAAGTCGAACGGTGT--CTTC--GGACATAGTGGCGGACGGGT
AJ293131     ------------------------CAGGATGAACGCTGGCGGTATGCTTAACACATGCAAGTCGAACGGTCT--TTTCGGAGA--TAGTGGCGGACGGGT
AJ133169     ------------------------CAGGATGAACGCTGGCGGTCTGCTTAACACATGCAAGTCGAACGGAAT--CCTTCGGGATTTAGTGGCGGACGGGT
DQ279769     -------AGAGTTTGATCCTGGCTCAGGATGAACGCTGGCGGTCTGCTTAACACATGCAAGTCGAACGGGCT--CTTC--GGAGCTAGTGGCGGACGGGT
AJ000714     -----------------------TCAGGATGAACGCTGGCGGTCTGCTTAACACATGCAAGTCGAACGGACT--CTTC--GGAGTTAGTGGCGGACGGGT
KVSF5        -------AGAGTTTGATCCTGGCTCAGGATGAACACTGGCGGTCTGCTTAACACATGCAAGTCGAACGGAATGCTTTCGGGCATTTAGTGGCGGACGGGT

                      110       120       130       140       150       160       170       180       190       200
             ....|....|....|....|....|....|....|....|....|....|....|....|....|....|....|....|....|....|....|....|
KVJ10        GAGTAACGCGTGAGAATCTGGCTTTAGGTCTGGGACAACCACTGGAAACGGTGGCTAATACCGGATGTGCCCGTAATTGGGTGAAAGGTTAACTGCCTGA
KVJF8        GAGTAACGCGTGAGAATCTGGCTTTAGGTCTGGGACAACCACTGGAAACGGTGGCTAATACCGGATGTGCCCTTCG--GGGTGAAAGGTTAACTGCCTGA
KVJ3         GAGTAACGCGTGAGAATCTGGCTCTAGGTCTGGGACAACCACTGGAAACGGTGGCTAATACCGGATGTGCCTTAATT--GGTGAAAGGTTAACTGCCTAG
SKS1         GAGTAACGCGTGAGAATCTGGCTCTAGGTCTGGGACAACCACTGGAAACGGTGGCTAATACCGGATGTGCCTTAATT--GGTGAAAGGTTAACTGCCTAG
KVS1         GAGTAACGCGTGAGAATCTGGCTCTAGGTCTGGGACAACCACTGGAAACGGTGGCTAATACCGGATGTGCCTTAATT--GGTGAGAGGTTAACTGCCTAG
SKJ1         GAGTAACGCGTGAGAATCTGGCTCTAGGTCTGGGACAACCACTGGAAACGGTGGCTAATACCGGATGTGCCTTAATT--GGTGAAAGGTTAACTGCCTAG
KVJF4        GAGTAACGCGTGAGAATCTGGCTTTAGGTTTGGGACAACCACTGGAAACGGTGGCTAATACCGGATGTGCCGAGA----GGTGAAAGGTTAACTGCCTAG
KVS11        GAGTAACGCGTGAGAATCTGGCTCTAGGTCTGGGACAACCACTGGAAACGGTGGCTAATACCGGATGTGCCGAGA----GGTGAAAGGTTAACTGCCTAG
KVJ20        GAGTAACGCGTGAGAATCTGGCTTCAGGTCTGGGACAACCACTGGAAACGGTGGCTAATACCGGATGTGCCGAGA----GGTGAAAGATTAATTGCCTGA
SKSF3        GAGTAACGCGTGAGAATCTGGCTTCAGGTCTGGGACAACCACTGGAAACGGTGGCTAATACCGGATGTCCCGAGA----GGTGAAAGATTAATTGCCTGA
SKSL1        GAGTAACGCGTGAGAATCTGGCTCTAGGTCTGGGACAACCACTGGAAACGGTGGCTAATACCGGATGTGCCGAGA----GGTGAAAGGTTAACTGCCTAG
SKSL2        GAGTAACGCGTGAGAATCTGGCTCTAGGTCTGGGACAACCACTGGAAACGGTGGCTAATACCGGATGTGCCGAGA----GGTGAAAGGTTAACTGCCTAG
```

```
KVJ4        GAGTAACGCGTGAGAATCTGCCTTCAGGTCTGGGACAACCACTGGAAACGGTGGCTAATACCGGATGTGCCTTTAGG--GGTGAAAGATTAATTGCCTGA
KVJ2        GAGTAACGCGTGAGAATCTGCCTTCAGGTCTGGGACAACCACTGGAAACGGTGGCTAATACCGGATGTGCCTTTAGG--GGTGAAAGATTAATTGCCTGA
KVSF4       GAGTAACGCGTGAGAATCTGCCTTCAGGTCTGGGACAACCACTGGAAACGGTGGCTAATACCGGATGTGCCTTTCGG--GGTGAAAGATTAATTGCCTGA
SKS2        GAGTAACGCGTGAGAATCTGCCTTCAGGTCTGGGACAACCACTGGAAACGGTGGCTAATACCGGATGTGCCTTTCGG--GGTGAAAGATTAATTGCCTGA
SKJF1       GAGTAACGCGTGAGAATCTGGCTTCAGGTCTGGGACAACCACTGGAAACGGTGGCTAATACCGGATGTGCCGAGA----GGTGAAAGGTTAACTGCCTGA
SKS3        GAGTAACGCGTGAGAATCTGGCTTCAGGTCTGGGACAACCACTGGAAACGGTGGCTAATACCGGATGTGCCGAGA----GGTGAAAGGTTAACTGCCTGA
SKJ6        GAGTAACGCGTGAGAATCTGGCTTCAGGTCTGGGACAACCACTGGAAACGGTGGCTAATACCGGATGTGCCGAGA----GGTGAAAGGTTAACTGCCTGA
SKJ2        GAGTAACGCGTGAGAATCTGGCTTCAGGTCTGGGACAACCACTGGAAACGGTGGCTAATACCGGATGTGCCGAGA----GGTGAAAGGTTAACTGCCTGA
SKS5        GAGTAACGCGTGAGAATCTGGCTTCAGGTCTGGGACAACCACTGGAAACGGTGGCTAATACCGGATGTGCCGAGA----GGTGAAAGGTTAACTGCCTGA
SKS9        GAGTAACGCGTGAGAATCTGGCTTCAGGTCTGGGACAACCACTGGAAACGGTGGCTAATACCGGATGTGCCGAGA----GGTGAAAGGTTAACTGCCTGA
KVJ18       GAGTAACGCGTGAGAATCTGGCTTCAGGTCTGGGACAACCACTGGAAACGGTGGCTAATACCGGATGTGCCGAGA----GGTGAAAGGTTAACTGCCTGA
SKJ4        GAGTAACGCGTGAGAATCTGGCTTCAGGTCTGGGACAACCACTGGAAACGGTGGCTAATACCGGATGTGCCGAGA----GGTGAAAGGTTAACTGCCTGA
SKSF1       GAGTAACGCGTGAGAATCTGGCTTCAGGTCGGGGACAACAGTTGGAAACGACTGCTAATACCGGATATGCCGAAA----GGTGAAAGGCTTGCTGCCTGA
KVSF7       GAGTAACGCGTGAGAATCTGGCTTCAGGTCTGGGACAACCACTGGAAACGGTGGCTAATACCGGATGTGCCGAGA----GGTGAAAGGCTTGCTGCCTGA
SKS8        GAGTAACGCGTGAGAATCTGGCTTCAGGTTCGGGACAACCACTGGAAACGGTGGCTAATACCGAATGTGCCGAGA----GGTGAAAGGCTTGCTGCCTGA
DQ185249    GAGTAACGCGTGAGAATCTGGCTTTAGGTCTGGGACAACCACTGGAAACGGTGGCTAATACCGGATGTGCCCGTAATTGGGTGAAAGGTTAACTGCCTGA
DQ185255    GAGTAACGCGTGAGAATCTGGCTTTAGGTCTGGGACAACCACTGGAAACGGTGGCTAATACCGGATGTGCCCGTAATTGGGTGAAAGGTTAACTGCCTGA
AY566855    GAGTAACGCGTGAGAATCTGGCTCTAGGTCTGGGACAACCACTGGAAACGGTGGCTAATACCGGATGTGCCGTAG----GGTGAAAGGTTAACTGCCTAG
AM711545    GAGTAACGCGTGAGAATCTGGCTCTAGGTCTGGGACAACCACTGGAAACGGTGGCTAATACCGGATGTGCCGAGA----GGTGAAAGGTTAACTGCCTAG
AM711536    GAGTAACGCGTGAGAATCTGGCTCTAGGTCTGGGACAACCACTGGAAACGGTGGCTAATACCGGATGTGCCGAGA----GGTGAAAGGTTAACTGCCTAG
DQ185256    GAGTAACGCGTGAGAATCTGGCTCTAGGTCTGGGACAACCACTGGAAACGGTGGCTAATACCGGATGTGCCGAGA----GGTGAAAGGTTAACTGCCTAG
DQ185245    GAGTAACGCGTGAGAATCTGGCTCTAGGTCTGGGACAACCACTGGAAACGGTGGCTAATACCGGATGTGCCGAGA----GGTGAAAGGTTAACTGCCTAG
AM711527    GAGTAACGCGTGAGAATCTGGCTCTAGGTCTGGGACAACCACTGGAAACGGTGGCTAATACCGGATGTGCCGAGA----GGTGAAAGGTTAACTGCCTAG
DQ185212    GAGTAACGCGTGAGAATCTGGCTCTAGGTCTGGGACAACCACTGGAAACGGTGGCTAATACCGGATGTGCCGAGA----GGTGAAAGGTTAACTGCCTAG
AF062638    GAGTAACGCGTGAGAATCTGGCTCTAGGTCTGGGACAACCACTGGAAACGGTGGCTAATACCGGATGTGCCGAGA----GGTGAAAGGTTAACTGCCTAG
HM623781    GAGTAACGCGTGAGAATCTGGCTTCAGGTCTGGGACAACCACTGGAAACGGTGGCTAATACCGGATGTGCCGAGA----GGTGAAAGATTAATTGCCTGA
AM711540    GAGTAACGCGTGAGAATCTGGCTTCAGGTCTGGGACAACCACTGGAAACGGTGGCTAATACCGGATGTGCCGAGA----GGTGAAAGATTAATTGCCTGA
DQ185254    GAGTAACGCGTGAGAATCTGCCTTCAGGTCTGGGACAACCACTGGAAACGGTGGCTAATACCGGATGTGCCGAAA----GGTGAAAGGTTAACTGCCTGA
DQ185251    GAGTAACGCGTGAGAATCTGGCTTCAGGTCTGGGACAACCACTGGAAACGGTGGCTAATACCGGATGTGCCGAAA----GGTGAAAGATTAATTGCCTGA
AM711535    GAGTAACGCGTGAGAATCTGGCTTCAGGTCTGGGACAACCACTGGAAACGGTGGCTAATACCGGATGTGCCGAGA----GGTGAAAGATTAATTGCCTGA
AF027653    GAGTAACGCGTGAGAATCTGGCTTCAGGTCTGGGACAACCACTGGAAACGGTGGCTAATACCGGATGTGCCGAGA----GGTGAAAGGTTAACTGCCTGA
DQ185244    GAGTAACGCGTGAGAATCTGGCTTCAGGTCTGGGACAACCACTGGAAACGGTGGCTAATACCGGATGTGCCGAGA----GGTGAAAGGTTAACTGCCTGA
AM711531    GAGTAACGCGTGAGAATCTGCCTTCAGGTCTGGGACAACCACTGGAAACGGTGGCTAATACCGGATGTGCCTTTCGG--GGTGAAAGATTAATTGCCTGA
AJ630449    GAGTAACGCGTGAGAATCTGGCTTCAGGTCTGGGACAACCACTGGAAACGGTGGCTAATACCGGATGTGCCCTTAGG--GGTGAAAGATTAATTGCCTGA
AY328897    GAGTAACGCGTGAGAATCTGGCTTCAGGTCTGGGACAACCACTGGAAACGGTGGCTAATACCGGATGTGCCGAGA----GGTGAAAGGTTAACTGCCTGA
AM230702    GAGTAACGCGTGAGAATCTAGCTTCAGGTCGGGGACAACAGTGGGAAACSGATGCTAATACCGGATRTGCCGAAA----GGTGAAAGGCTTGCTGCCTGA
EF568907    GAGTAACGCGTGAGAATCTAGCTTCAGGTCGGGGACAACAGTTGGAAACGACTGCTAATACCGGATATGCCGAGA----GGTGAAAGGCTTGCTGCCTGA
AJ630458    GAGTAACGCGTGAGAATCTGGCTTCAGGTCGGGGACAACAGTTGGAAACGACTGCTAATACCGGATATGCCGAGA----GGTGAAAGATTAATTGCCTGG
GQ443447    GAGTAACGCGTGAGAATCTGGCTTCAGGTCGGGGACAACAGTTGGAAACGACTGCTAATACCGGATATGCCGAGA----GGTGAAAGATTAATTGCCTGG
AJ630457    GAGTAACGCGTGAGAATCTGGCTTCAGGTCTGGGACAACCACTGGAAACGGTGGCTAATACCGGATGTGCCGAGA----GGTGAAAGGCTTGCTGCCTGA
DQ234831    GAGTAACGCGTGAGAATCTGGCTTCAGGTCTGGGACAACCACTGGAAACGGTGGCTAATACCGGATGTGCCGAGA----GGTGAAAGGCTTGCTGCCTGA
AJ133161    GAGTAACGCGTGAGAATCTGGCTTCAGGTTCGGGACAACCACTGGAAACGGTGGCTAATACCGAATGTGCCGAGA----GGTGAAAGGCTTGCTGCCTGA
gi|47118302 GAGTAACGCGTGAGAATCTAGCTTCAGGTCGGGGACAACCACTGGAAACGGTGGCTAATACCGGATGTGCCGAAA----GGTGAAAGATTTATTGCCTGA
DQ185209    GAGTAACGCGTGAGAATCTGGCTTTAGGTCTGGGACAACCACTGGAAACGGTGGCTAATACCGGATGTGCCGAAA----GGTGAAAGATTAATTGCCTGA
AY742451    GAGTAACGCGTGAGAATCTGGCTTCAGGTCTGGGACAACCACTGGAAACGGTGGCTAATACCGGATGTGCCGAGA----GGTGAAAGATTAATTGCCTGA
AF027655    GAGTAACGCGTGAGAATCTGGCTCTAGGTCTGGGACAACCACTGGAAACGGTGGCTAATACCGGATGTGCCGAGA----GGTGAAAGGTTAACTGCCTAG
```

```
DQ185211     GAGTAACGCGTGAGAATCTGGCTCTAGGTCTGGGACAACCACTGGAAACGGTGGCTAATACCGGATGTGCCGAGA----GGTGAAAGGTTAACTGCCTAG
AY742449     GAGTAACGCGTGAGAATCTGGCTCTAGGTCTGGGACAACCACTGGAAACGGTGGCTAATACCGGATGTGCCGAGA----GGTGAAAGGTTAACTGCCTAG
EF174212     GAGTAACGCGTGAGAATCTGCCATCAGGTCTGGGACAACCACTGGAAACGGTGGCTAATACCGGATGTGCCCTTCGG--GGTGAAAGGTTAACTGCCTGT
DQ185213     GAGTAACGCGTGAGAATCTGGCTCTAGGTCTGGGACAACCACTGGAAACGGTGGCTAATACCGGATGTGCCGAGA----GGTGAAAGGTTAACTGCCTAG
AF506237     GAGTAACGCGTGAGAATCTAGCTCTAGGTCTAGGACAACCATTGGAAACGGTGGCTAATACTGGATGTGCCGAGA----GGTGAAAGGCTTGCTGCCTAG
AM230690     GAGTAACGCGTGAGAATCTAGCTCTAGGTCTAGGACAACCATTGGAAACGGTGGCTAATACTGGATGTGCCGAGA----GGTAAAAGGCTTGCTGCCTAG
AB039002     GAGTAACGCGTGAGAATCTGGCTTCAGGTCGGGGACAACCACTGGAAACGGTGGCTAATACCGGATATGCCGAGA----GGTGAAAGGCTTGCTGCCTGA
AJ293131     GAGTAACGCGTAAGAATCTACCTTCAGGTTGGGGACAACCACTGGAAACGGTGGCTAATACCCAATGTGCCGAGA----GGTGAAAGGCTTGCTGCCTGA
AJ133169     GAGTAACACGTAAGAACCTGCCTCTAGGACGGGGACAACAGTTGGAAACGACTGCTAAACCCGGATGAGCCGAAA----GGTAAAAGATTAATCGCCTAG
DQ279769     GAGTAACACGTGAGAATCTGGCTCCCGGTCGGGGACAACAGAGGGAAACTTCTGCTAATCCCGGATGAGCCGAAA----GGTAAAAGATTTATCGCCGGG
AJ000714     GAGTAACGCGTGAGAATCTGCCTCTAGAACGGGGACAACAGAGGGAAACTTCTGCTAATCCCGGATGAGCCGAAA----GGTCAAAGATTTATCGTCTAG
KVSF5        GAGTAACGCGTGAGAATCTGCCCTTAGGATGGGGACAACAGTTGGAAACGACTGCTAAGACCCGATGTGCCGAGA----GGTGAAATATTTATAGCCTGA

                       210       220       230       240       250       260       270       280       290       300
             ....|....|....|....|....|....|....|....|....|....|....|....|....|....|....|....|....|....|....|....|
KVJ10        AGATGAGCTCGCGTCTGATTAGCTAGTTGGAAGTGTAATGGACTCCCAAGGCGACGATCAGTAGCTGGTCTGAGAGGACGATCAGCCACACTGGGACTGA
KVJF8        AGATGAGCTCGCGTCTGATTAGCTAGTTGGAAGTGTAATGGACTCCCAAGGCGACGATCAGTAGCTGGTCTGAGAGGACGATCAGCCACACTGGGACTGA
KVJ3         AGATGAGCTCGCGTCTGATTAGCTAGTTGGAAGTGTAATGGACTCCCAAGGCGACGATCAGTAGCTGGTCTGAGAGGACGATCAGCCACACTGGGACTGA
SKS1         AGATGAGCTCGCGTCTGATTAGCTAGTTGGAAGTGTAATGGACTCCCAAGGCGACGATCAGTAGCTGGTCTGAGAGGACGATCAGCCACACTGGGACTGA
KVS1         AGATGAGCTCGCGTCTGATTAGCTAGTTGGAAGTGTAATGGACTCCCAAGGCGACGATCAGTAGCTGGTCTGAGAGGACGATGAGCCACACTGGGACTGA
SKJ1         AGATGAGCTCGCGTCTGATTAGCTAGTTGGAAGTGTAATGGACTCCCAAGGCGACGATCAGTAGCTGGTCTGAGAGGACGATCAGCCACACTGGGACTGA
KVJF4        AGATGAGCTCGCGTCTGATTAGCTAGTTGGAAGTGTAATGGACTCCCAAGGCGACGATCAGTAGCTGGTCTGAGAGGACGATCAGCCACACTGGGACTGA
KVS11        AGATGAGCTCGCGTCTGATTAGCTAGTTGGAAGTGTAATGGACTCCCAAGGCGACGATCAGTAGCTGGTCTGAGAGGACGATCAGCCACACTGGGACTGA
KVJ20        AGATGAGCTCGCGTCTGATTAGCTAGTAGGTAGTGTAAAGGAGCACCTAGGCGACGATCAGTAGCTGGTCTGAGAGGACGATCAGCCACACTGGGACTGA
SKSF3        AGATGAGCTCGCGTCTGATTAGCTAGTAGGTAGTGTAAAGGACCACCTAGGCGACGATCAGTAGCTGGTCTGAGAGGACGATCAGCCACACTGGGACTGA
SKSL1        AGATGAGCTCGCGTCTGATTAGCTAGTTGGAAGTGTAATGGACTCCCAAGGCGACGATCAGTAGCTGGTCTGAGAGGACGATCAGCCACACTGGGACTGA
SKSL2        AGATGAGCTCGCGTCTGATTAGCTAGTTGGAAGTGTAATGGACTCCCAAGGCGACGATCAGTAGCTGGTCTGAGAGGACGATCAGCCACACTGGGACTGA
KVJ4         AGATGAGCTCGCGTCTGATTAGCTAGTTGGTAGTGTAATGGACAACCAAGGCGACGATCAGTAGCTGGTCTGAGAGGACGATCAGCCACACTGGGACTGA
KVJ2         AGATGAGCTCGCGTCTGATTAGCTAGTTGGTAGTGTAATGGACAACCAAGGCGACGATCAGTAGCTGGTCTGAGAGGACGATCAGCCACACTGGGACTGA
KVSF4        AGATGAGCTCGCGTCTGATTAGCTAGTTGGTAGTGTAATGGACAACCAAGGCGACGATCAGTAGCTGGTCTGAGAGGACGATCAGCCACACTGGGACTGA
SKS2         AGATGAGCTCGCGTCTGATTAGCTAGTTGGTAGTGTAATGGACAACCAAGGCGACGATCAGTAGCTGGTCTGAGAGGACGATCAGCCACACTGGGACTGA
SKJF1        AGATGAGCTCGCGTCTGATTAGCTAGTTGGAAGTGTAACGGACTCCCAAGGCGACGATCAGTAGCTGGTCTGAGAGGACGATCAGCCACACTGGGACTGA
SKS3         AGATGAGCTCGCGTCTGATTAGCTAGTTGGAAGTGTAACGGACTCCCAAGGCGACGATCAGTAGCTGGTCTGAGAGGACGATCAGCCACACTGGGACTGA
SKJ6         AGATGAGCTCGCGTCTGATTAGCTAGTTGGAAGTGTAACGGACTCCCAAGGCGACGATCAGTAGCTGGTCTGAGAGGACGATCAGCCACACTGGGACTGA
SKJ2         AGATGAGCTCGCGTCTGATTAGCTAGTTGGAAGTGTAATGGACTCCCAAGGCGACGATCAGTAGCTGGTCTGAGAGGACGATCAGCCACACTGGGACTGA
SKS5         AGATGAGCTCGCGTCTGATTAGCTAGTTGGAAGTGTAATGGACTCCCAAGGCGACGATCAGTAGCTGGTCTGAGAGGACGATCAGCCACACTGGGACTGA
SKS9         AGATGAGCTCGCGTCTGATTAGCTAGTTGGAAGTGTAATGGACTCCCAAGGCGACGATCAGTAGCTGGTCTGAGAGGACGATCAGCCACACTGGGACTGA
KVJ18        AGATGAGCTCGCGTCTGATTAGCTAGTTGGAAGTGTAATGGACTCCCAAGGCGACGATCAGTAGCTGGTCTGAGAGGACGATCAGCCACACTGGGACTGA
SKJ4         AGATGAGCTCGCGTCTGATTAGCTAGTTGGAAGTGTAACGGACTCCCAAGGCGACGATCAGTAGCTGGTCTGAGAGGACGATCAGCCACACTGGGACTGA
SKSF1        AGATGAGCTCGCGTCTGATTAGCTAGTTGGTGGGGTAAGAGCCTACCAAGGCGACGATCAGTAGCTGGTCTGAGAGGATGATCAGCCACACTGGGACTGA
KVSF7        AGATGAGCTTGCGTCTGATTAGCTAGTTGGTGGGGTAAGAGCCTACCAAGGCGACGATCAGTAGCTGGTCTGAGAGGATGATCAGCCACACTGGGACTGA
SKS8         AGATGAGCTCGCGTCTGATTAGCTAGTAGGTGTGGTAAAGGCGCACCTAGGCGACGATCATTAGCTGGTCTGAGAGGATGATCAGCCACACTGGGACTGA
DQ185249     AGATGAGCTCGCGTCTGATTAGCTAGTTGGAAGTGTAATGGACTCCCAAGGCGACGATCAGTAGCTGGTCTGAGAGGACGATCAGCCACACTGGGACTGA
DQ185255     AGATGAGCTCGCGTCTGATTAGCTAGTTGGAAGTGTAATGGACTCCCAAGGCGACGATCAGTAGCTGGTCTGAGAGGACGATCAGCCACACTGGGACTGA
AY566855     AGATGAGCTCGCGTCTGATTAGCTAGTTGGAAGTGTAATGGACTCCCAAGGCGACGATCAGTAGCTGGTCTGAGAGGACGATCAGCCACACTGGGACTGA
AM711545     AGATGAGCTCGCGTCTGATTAGCTAGTTGGAAGTGTAATGGACTCCCAAGGCGACGATCAGTAGCTGGTCTGAGAGGACGATCAGCCACACTGGGACTGA
```

```
AM711536      AGATGAGCTCGCGTCTGATTAGCTAGTTGGAAGTGTAATGGACTCCCAAGGCGACGATCAGTAGCTGGTCTGAGAGGACGATCAGCCACACTGGGACTGA
DQ185256      AGATGAGCTCGCGTCTGATTAGCTAGTTGGAAGTGTAATGGACTCCCAAGGCGACGATCAGTAGCTGGTCTGAGAGGACGATCAGCCACACTGGGACTGA
DQ185245      AGATGAGCTCGCGTCTGATTAGCTAGTTGGAAGTGTAATGGACTCCCAAGGCGACGATCAGTAGCTGGTCTGAGAGGACGATCAGCCACACTGGGACTGA
AM711527      AGATGAGCTCGCGTCTGATTAGCTAGTTGGAAGTGTAATGGACTCCCAAGGCGACGATCAGTAGCTGGTCTGAGAGGACGATCAGCCACACTGGGACTGA
DQ185212      AGATGAGCTCGCGTCTGATTAGCTAGTTGGAAGTGTAATGGACTCCCAAGGCGACGATCAGTAGCTGGTCTGAGAGGACGATCAGCCACACTGGGACTGA
AF062638      AGATGAGCTCGCGTCTGATTAGCTAGTTGGAAGTGTAATGGACTCCCAAGGCGACGATCAGTAGCTGGTCTGAGAGGACGATCAGCCACACTGGGACTGA
HM623781      AGATGAGCTCGCGTCTGATTAGCTAGTAGGTGTGGTAAAGGCGCACCTAGGCGACGATCAGTAGCTGGTCTGAGAGGACGATCAGCCACACTGGGACTGA
AM711540      AGATGAGCTCGCGTCTGATTAGCTAGTAGGTGTGGTAAAGGCGCACCTAGGCGACGATCAGTAGCTGGTCTGAGAGGACGATCAGCCACACTGGGACTGA
DQ185254      AGATGAGCTCGCGTCTGATTAGCTAGTAGGTAGTGTAAAGGACTACCTAGGCGACGATCAGTAGCTGGTCTGAGAGGACGATCAGCCACACTGGGACTGA
DQ185251      AGATGAGCTCGCGTCTGATTAGCTAGTAGGTGTGGTAAAGGCGCACCTAGGCGACGATCAGTAGCTGGTCTGAGAGGACGATCAGCCACACTGGGACTGA
AM711535      AGATGAGCTCGCGTCTGATTAGCTAGTAGGTGTGGTAAAGGCGCACCTAGGCGACGATCAGTAGCTGGTCTGAGAGGACGATCAGCCACACTGGGACTGA
AF027653      AGATGAGCTCGCGTCTGATTAGCTAGTTGGAAGTGTAATGGACTCCCAAGGCGACGATCAGTAGCTGGTCTGAGAGGACGATCAGCCACACTGGGACTGA
DQ185244      AGATGAGCTCGCGTCTGATTAGCTAGTTGGAAGTGTAATGGACTCCCAAGGCGACGATCAGTAGCTGGTCTGAGAGGACGATCAGCCACACTGGGACTGA
AM711531      AGATGAGCTCGCGTCTGATTAGCTAGTTGGTAGTGTAATGGACAACCAAGGCGACGATCAGTAGCTGGTCTGAGAGGACGATCAGCCACACTGGGACTGA
AJ630449      AGATGAGCTCGCGTCTGATTAGCTAGTAGGTGTGGTAAAGGCGCACCTAGGCGACGATCAGTAGCTGGTCTGAGAGGACGATCAGCCACACTGGGACTGA
AY328897      AGATGAGCTCGCGTCTGATTAGCTAGTTGGTAGAGTAAGAGCCTACCAAGGCGACGATCAGTAGCTGGTCTGAGAGGACGATCAGCCACACTGGGACTGA
AM230702      AGATGAGCTCGCGTCAGATTAGCTAGTTGGTGGGGTAAGAGCCTACCAAGGCGACGATCAGTAGCTGGTCTGAGAGGATGATCAGCCACACTGGGACTGA
EF568907      AGATGAGCTCGCGTCTGATTAGCTAGTTGGTGGGGTAAGAGCCTACCAAGGCGACGATCAGTAGCTGGTCTGAGAGGATGATCAGCCACACTGGGACTGA
AJ630458      AGATGAGCTCGCGTCTGATTAGCTAGTTGGTGGGGTAAGAGCCTACCAAGGCGACGATCAGTAGCTGGTCTGAGAGGATGATCAGCCACACTGGGACTGA
GQ443447      AGATGAGCTCGCGTCTGATTAGCTAGTTGGTGGGGTAAGAGCCTACCAAGGCGACGATCAGTAGCTGGTCTGAGAGGATGATCAGCCACACTGGGACTGA
AJ630457      AGATGAGCTTGCGTCTGATTAGCTAGTTGGTGGGGTAAGAGCCTACCAAGGCGACGATCAGTAGCTGGTCTGAGAGGATGATCAGCCACACTGGGACTGA
DQ234831      AGATGAGCTTGCGTCTGATTAGCTAGTTGGTGGGGTAAGAGCCTACCAAGGCGACGATCAGTAGCTGGTCTGAGAGGATGATCAGCCACACTGGGACTGA
AJ133161      AGATGAGCTCGCGTCTGATTAGCTAGTAGGTGTGGTAAAGGCGCACCTAGGCGACGATCAGTAGCTGGTCTGAGAGGATGATCAGCCACACTGGGACTGA
gi|47118302   AGATGAGCTCGCGTCTGATTAGCTAGTTGGTGTGGTAAGAGCGCACCAAGGCGACGATCAGTAGCTGGTCTGAGAGGATGATCAGCCACACTGGGACTGA
DQ185209      AGATGAGCTCGCGTCTGATTAGCTAGTTGGAAGTGTAATGGACTCCCAAGGCGACGATCAGTAGCTGGTCTGAGAGGACGATCAGCCACACTGGGACTGA
AY742451      AGATGAGCTCGCGTCTGATTAGCTAGTAGGTGTGGTAAAGGCGCACCTAGGCGACGATCAGTAGCTGGTCTGAGAGGACGATCAGCCACACTGGGACTGA
AF027655      AGATGAGCTCGCGTCTGATTAGCTAGTAGGTAGTGTAATGGACTACCTAGGCGACGATCAGTAGCTGGTCTGAGAGGACGATCAGCCACACTGGGACTGA
DQ185211      AGATGAGCTCGCGTCTGATTAGCTAGTAGGTAGTGTAATGGACTACCTAGGCGACGATCAGTAGCTGGTCTGAGAGGACGATCAGCCACACTGGGACTGA
AY742449      AGATGAGCTCGCGTCTGATTAGCTAGTTGGAAGTGTAATGGACTCCCAAGGCGACGATCAGTAGCTGGTCTGAGAGGACGATCAGCCACACTGGGACTGA
EF174212      TGATGAGCTCGCGTCTGATTAGCTAGTTGGTGTGGTAAGAGCGCACCAAGGCGACGATCAGTAGCTGGTCTGAGAGGACGATCAGCCACACTGGGACTGA
DQ185213      AGATGAGCTCGCGTCTGATTAGCTAGTAGGTGGTGTAAAGGACTACCTAGGCGACGATCAGTAGCTGGTCTGAGAGGACGATCAGCCACACTGGGACTGA
AF506237      AGATGAGCTTGCGTCAGATTAGCTAGTAGGTGTGGTAAGAGCGCACCTAGGCGACGATCTGTAGCTGGTCTGAGAGGACGATCAGCCACACTGGAACTGA
AM230690      AGAAGAGCTTGCGTCAGATTAGCTAGTAGGTGTGGTAAGAGCGCACCTAGGCGACGATCTGTAGCTGGTCTGAGAGGACGATCAGCCACACTGGAACTGA
AB039002      AGATGAGCTCGCGTCTGATTAGCTAGTAGGTGTGGTAAAAGCGCACCTAGGCGACGATCAGTAGCTGGTCTGAGAGGATGATCAGCCACACTGGGACTGA
AJ293131      AGAAGAGCTTGCGTCTGATTAGCTAGTTGGTGGGGTAAGAGCCTACCAAGGCGACGATCAGTAGCTGGTCTGAGAGGATGATCAGCCACACTGGGACTGA
AJ133169      AGAGGGGCTTGCGTCTGATTAGCTAGTTGGTGAGGTAAGAGCCCACCAAGGCGACGATCAGTAGCTGGTCTGAGAGGATGATCAGCCACACTGGGACTGA
DQ279769      AGATGAGCTCGCGTCTGATTAGCTAGTTGGTGAGGTAAAGGCTCACCAAGGCGACGATCAGTAGCTGGTCTGAGAGGATGATCAGCCACACTGGGACTGA
AJ000714      AGATGAGCTCGCGTCTGATTAGCTAGTTGGTAAGGTAAARGCTTACCAAGGCGACGATCAGTAGCTGGTCTGAGAGGATGATCAGCCACACTGGGACTGA
KVSF5         GGATGAGCTCGCGTCAGATTAGCTAGTTGGTGGGGTAATGGCCTACCAAGGCGACGATCTGTAGCTGGTCTGAGAGGATGATCAGCCACACTGGAACTGA

                       310       320       330       340       350       360       370       380       390       400
              ....|....|....|....|....|....|....|....|....|....|....|....|....|....|....|....|....|....|....|....|
KVJ10         GACACGGCCCAGACTCCTACGGGAGGCAGCAGTGGGGAATTTTCCGCAATGGGCGAAAGCCTGACGGAGCAATACCGCGTGAGGGAGGAAGGCTCTTGGG
KVJF8         GACACGGCCCAGACTCCTACGGGAGGCAGCAGTGGGGAATTTTCCGCAATGGGCGAAAGCCTGACGGAGCAATACCGCGTGAGGGAGGAAGGCTCTTGGG
KVJ3          GACACGGCCCAGACTCCTACGGGAGGCAGCAGTGGGGAATTTTCCGCAATGGGCGAAAGCCTGACGGAGCAATACCGCGTGAGGGAGGAAGGCTCTTGGG
SKS1          GACACGGCCCAGACTCCTACGGGAGGCAGCAGTGGGGAATTTTCCGCAATGGGCGAAAGCCTGACGGAGCAATACCGCGTGAGGGAGGAAGGCTCTTGGG
```

KVS1	GACACGGCCCAGACTCCTACGGGAGGCAGCAGTGGGGAATTTTCCGCAATGGGCGAAAGCCTGACGGAGCAATACCGCGTGAGGGAGGAAGGCTCTTGGG
SKJ1	GACACGGCCCAGACTCCTACGGGAGGCAGCAGTGGGGAATTTTCCGCAATGGGCGAAAGCCTGACGGAGCAATACCGCGTGAGGGAGGAAGGCTCTTGGG
KVJF4	GACACGGCCCAGACTCCTACGGGAGGCAGCAGTGGGGAATTTTCCGCAATGGGCGAAAGCCTGACGGAGCAATACCGCGTGAGGGAGGAAGGCTCTTGGG
KVS11	GACACGGCCCAGACTCCTACGGGAGGCAGCAGTGGGGAATTTTCCGCAATGGGCGAAAGCCTGACGGAGCAATACCGCGTGAGGGAGGAAGGCTCTTGGG
KVJ20	GACACGGCCCAGACTCCTACGGGAGGCAGCAGTGGGGAATTTTCCGCAATGGGCGAAAGCCTGACGGAGCAATACCGCGTGAGGGAGGAAGGCTCTTGGG
SKSF3	GACACGGCCCAGACTCCTACGGGAGGCAGCAGTGGGGAATTTTCCGCAATGGGCGAAAGCCTGACGGAGCAATACCGCGTGAGGGAGGAAGGCTCTTGGG
SKSL1	GACACGGCCCAGACTCCTACGGGAGGCAGCAGTGGGGAATTTTCCGCAATGGGCGAAAGCCTGACGGAGCAATACCGCGTGAGGGAGGAAGGCTCTTGGG
SKSL2	GACACGGCCCAGACTCCTACGGGAGGCAGCAGTGGGGAATTTTCCGCAATGGGCGAAAGCCTGACGGAGCAATACCGCGTGAGGGAGGAAGGCTCTTGGG
KVJ4	GACACGGCCCAGACTCCTACGGGAGGCAGCAGTGGGGAATTTTCCGCAATGGGCGAAAGCCTGACGGAGCAATACCGCGTGAGGGAGGAAGGCTCTTGGG
KVJ2	GACACGGCCCAGACTCCTACGGGAGGCAGCAGTGGGGAATTTTCCGCAATGGGCGAAAGCCTGACGGAGCAATACCGCGTGAGGGAGGAAGGCTCTTGGG
KVSF4	GACACGGCCCAGACTCCTACGGGAGGCAGCAGTGGGGAATTTTCCGCAATGGGCGAAAGCCTGACGGAGCAATACCGCGTGAGGGAGGAAGGCTCTTGGG
SKS2	GACACGGCCCAGACTCCTACGGGAGGCAGCAGTGGGGAATTTTCCGCAATGGGCGAAAGCCTGACGGAGCAATACCGCGTGAGGGAGGAAGGCTCTTGGG
SKJF1	GACACGGCCCAGACTCCTACGGGAGGCAGCAGTGGGGAATTTTCCGCAATGGGCGAAAGCCTGACGGAGCAATACCGCGTGAGGGAGGAAGGCTCTTGGG
SKS3	GACACGGCCCAGACTCCTACGGGAGGCAGCAGTGGGGAATTTTCCGCAATGGGCGAAAGCCTGACGGAGCAATACCGCGTGAGGGAGGAAGGCTCTTGGG
SKJ6	GACACGGCCCAGACTCCTACGGGAGGCAGCAGTGGGGAATTTTCCGCAATGGGCGAAAGCCTGACGGAGCAATACCGCGTGAGGGAGGAAGGCTCTTGGG
SKJ2	GACACGGCCCAGACTCCTACGGGAGGCAGCAGTGGGGAATTTTCCGCAATGGGCGAAAGCCTGACGGAGCAATACCGCGTGAGGGAGGAAGGCTCTTGGG
SKS5	GACACGGCCCAGACTCCTACGGGAGGCAGCAGTGGGGAATTTTCCGCAATGGGCGAAAGCCTGACGGAGCAATACCGCGTGAGGGAGGAAGGCTCTTGGG
SKS9	GACACGGCCCAGACTCCTACGGGAGGCAGCAGTGGGGAATTTTCCGCAATGGGCGAAAGCCTGACGGAGCAATACCGCGTGAGGGAGGAAGGCTCTTGGG
KVJ18	GACACGGCCCAGACTCCTACGGGAGGCAGCAGTGGGGAATTTTCCGCAATGGGCGAAAGCCTGACGGAGCAATACCGCGTGAGGGAGGAAGGCTCTTGGG
SKJ4	GACACGGCCCAGACTCCTACGGGAGGCAGCAGTGGGGAATTTTCCGCAATGGGCGAAAGCCTGACGGAGCAATACCGCGTGAGGGAGGAAGGCTCTTGGG
SKSF1	GACACGGCCCAGACTCCTACGGGAGGCAGCAGTGGGGAATTTTCCGCAATGGGCGAAAGCCTGACGGAGCAATACCGCGTGAGGGAGGAAGGCTCTTGGG
KVSF7	GACACGGCCCAGACTCCTACGGGAGGCAGCAGTGGGGAATTTTCCGCAATGGGCGAAAGCCTGACGGAGCAATACCGCGTGAGGGAGGAAGGCTCTTGGG
SKS8	GACACGGTCCAGACTCCTACGGGAGGCAGCAGTGGGGAATTTTCCGCAATGGGCGAAAGCCTGACGGAGCAATACCGCGTGAGGGAGGAAGGCTCTTGGG
DQ185249	GACACGGCCCAGACTCCTACGGGAGGCAGCAGTGGGGAATTTTCCGCAATGGGCGAAAGCCTGACGGAGCAATACCGCGTGAGGGAGGAAGGCTCTTGGG
DQ185255	GACACGGCCCAGACTCCTACGGGAGGCAGCAGTGGGGAATTTTCCGCAATGGGCGAAAGCCTGACGGAGCAATACCGCGTGAGGGAGGAAGGCTCTTGGG
AY566855	GACACGGCCCAGACTCCTACGGGAGGCAGCAGTGGGGAATTTTCCGCAATGGGCGAAAGCCTGACGGAGCAATACCGCGTGAGGGAGGAAGGCTCTTGGG
AM711545	GACACGGCCCAGACTCCTACGGGAGGCAGCAGTGGGGAATTTTCCGCAATGGGCGAAAGCCTGACGGAGCAATACCGCGTGAGGGAGGAAGGCTCTTGGG
AM711536	GACACGGCCCAGACTCCTACGGGAGGCAGCAGTGGGGAATTTTCCGCAATGGGCGAAAGCCTGACGGAGCAATACCGCGTGAGGGAGGAAGGCTCTTGGG
DQ185256	GACACGGCCCAGACTCCTACGGGAGGCAGCAGTGGGGAATTTTCCGCAATGGGCGAAAGCCTGACGGAGCAATACCGCGTGAGGGAGGAAGGCTCTTGGG
DQ185245	GACACGGCCCAGACTCCTACGGGAGGCAGCAGTGGGGAATTTTCCGCAATGGGCGAAAGCCTGACGGAGCAATACCGCGTGAGGGAGGAAGGCTCTTGGG
AM711527	GACACGGCCCAGACTCCTACGGGAGGCAGCAGTGGGGAATTTTCCGCAATGGGCGAAAGCCTGACGGAGCAATACCGCGTGAGGGAGGAAGGCTCTTGGG
DQ185212	GACACGGCCCAGACTCCTACGGGAGGCAGCAGTGGGGAATTTTCCGCAATGGGCGAAAGCCTGACGGAGCAATACCGCGTGAGGGAGGAAGGCTCTTGGG
AF062638	GACACGGCCCAGACTCCTACGGGAGGCAGCAGTGGGGAATTTTCCGCAATGGGCGAAAGCCTGACGGAGCAATACCGCGTGAGGGAGGAAGGCTCTTGGG
HM623781	GACACGGCCCAGACTCCTACGGGAGGCAGCAGTGGGGAATTTTCCGCAATGGGCGAAAGCCTGACGGAGCAATACCGCGTGAGGGAGGAAGGCTCTTGGG
AM711540	GACACGGCCCAGACTCCTACGGGAGGCAGCAGTGGGGAATTTTCCGCAATGGGCGAAAGCCTGACGGAGCAATACCGCGTGAGGGAGGAAGGCTCTTGGG
DQ185254	GACACGGCCCAGACTCCTACGGGAGGCAGCAGTGGGGAATTTTCCGCAATGGGCGAAAGCCTGACGGAGCAATACCGCGTGAGGGAGGAAGGCTCTTGGG
DQ185251	GACACGGCCCAGACTCCTACGGGAGGCAGCAGTGGGGAATTTTCCGCAATGGGCGAAAGCCTGACGGAGCAATACCGCGTGAGGGAGGAAGGCTCTTGGG
AM711535	GACACGGCCCAGACTCCTACGGGAGGCAGCAGTGGGGAATTTTCCGCAATGGGCGAAAGCCTGACGGAGCAATACCGCGTGAGGGAGGAAGGCTCTTGGG
AF027653	GACACGGCCCAGACTCCTACGGGAGGCAGCAGTGGGGAATTTTCCGCAATGGGCGAAAGCCTGACGGAGCAATACCGCGTGAGGGAGGAAGGCTCTTGGG
DQ185244	GACACGGCCCAGACTCCTACGGGAGGCAGCAGTGGGGAATTTTCCGCAATGGGCGAAAGCCTGACGGAGCAATACCGCGTGAGGGAGGAAGGCTCTTGGG
AM711531	GACACGGCCCAGACTCCTACGGGAGGCAGCAGTGGGGAATTTTCCGCAATGGGCGAAAGCCTGACGGAGCAATACCGCGTGAGGGAGGAAGGCTCTTGGG
AJ630449	GACACGGCCCAGACTCCTACGGGAGGCAGCAGTGGGGAATTTTCCGCAATGGGCGAAAGCCTGACGGAGCAATACCGCGTGAGGGAGGAAGGCTCTTGGG
AY328897	GACACGGCCCAGACTCCTACGGGAGGCAGCAGTGGGGAATTTTCCGCAATGGGCGAAAGCCTGACGGAGCAATACCGCGTGAGGGAGGAAGGCTCTTGGG
AM230702	GACACGGYCCAGACTCCTACGGGAGGCAGCAGTGGGGAATTTTCCGCAATGGGCGAAAGCCTGACGGAGCAATACCGCGTGAGGGAGGAAGGCTCTTGGG
EF568907	GACACGGCCCAGACTCCTACGGGAGGCAGCAGTGGGGAATTTTCCGCAATGGGCGAAAGCCTGACGGAGCAATACCGCGTGAGGGAGGAAGGCTCTTGGG
AJ630458	GACACGGCCCAGACTCCTACGGGAGGCAGCAGTGGGGAATTTTCCGCAATGGGCGAAAGCCTGACGGAGCAATACCGCGTGAGGGAGGAAGGCTCTTGGG

```
GQ443447      GACACGGCCCAGACTCCTACGGGAGGCAGCAGTGGGGAATTTTCCGCAATGGGCGAAAGCCTGACGGAGCAATACCGCGTGAGGGAGGAAGGCTCTTGGG
AJ630457      GACACGGCCCAGACTCCTACGGGAGGCAGCAGTGGGGAATTTTCCGCAATGGGCGAAAGCCTGACGGAGCAATACCGCGTGAGGGAGGAAGGCTCTTGGG
DQ234831      GACACGGCCCAGACTCCTACGGGAGGCAGCAGTGGGGAATTTTCCGCAATGGGCGAAAGCCTGACGGAGCAATACCGCGTGAGGGAGGAAGGCTCTTGGG
AJ133161      GACACGGCCCAGACTCCTACGGGAGGCAGCAGTGGGGAATTTTCCGCAATGGGCGAAAGCCTGACGGAGCAATACCGCGTGAGGGAGGAAGGCTCTTGGG
gi|47118302   GACACGGCCCAGACTCCTACGGGAGGCAGCAGTGGGGAATTTTCCGCAATGGGCGAAAGCCTGACGGAGCAATACCGCGTGAGGGAGGAAGGCTCTTGGG
DQ185209      GACACGGCCCAGACTCCTACGGGAGGCAGCAGTGGGGAATTTTCCGCAATGGGCGAAAGCCTGACGGAGCAATACCGCGTGAGGGAGGAAGGCTCTTGGG
AY742451      GACACGGCCCAGACTCCTACGGGAGGCAGCAGTGGGGAATTTTCCGCAATGGGCGAAAGCCTGACGGAGCAATACCGCGTGAGGGAGGAAGGCTCTTGGG
AF027655      GACACGGCCCAGACTCCTACGGGAGGCAGCAGTGGGGAATTTTCCGCAATGGGCGAAAGCCTGACGGAGCAATACCGCGTGAGGGAGGAAGGCTCTTGGG
DQ185211      GACACGGCCCAGACTCCTACGGGAGGCAGCAGTGGGGAATTTTCCGCAATGGGCGAAAGCCTGACGGAGCAATACCGCGTGAGGGAGGAAGGCTCTTGGG
AY742449      GACACGGCCCAGACTCCTACGGGAGGCAGCAGTGGGGAATTTTCCGCAATGGGCGAAAGCCTGACGGAGCAATACCGCGTGAGGGAGGAAGGCTCTTGGG
EF174212      GACACGGCCCAGACTCCTACGGGAGGCAGCAGTGGGGAATTTTCCGCAATGGGCGAAAGCCTGACGGAGCAATACCGCGTGAGGGAGGAAGGCTCTTGGG
DQ185213      GACACGGCCCAGACTCCTACGGGAGGCAGCAGTGGGGAATTTTCCGCAATGGGCGAAAGCCTGACGGAGCAATACCGCGTGAGGGAGGAAGGCTCTTGGG
AF506237      GACACGGTCCAGACTCCTACGGGAGGCAGCAGTGGGGAATTTTCCGCCATGGGCGAAAGCCTGACGGAGCAATACCGCGTGAGGGAGGAAGGCTCTTGGG
AM230690      GACACGGTCCAGACTCCTACGGGAGGCAGCAGTGGGGAATTTTCCGCAATGGGCGAAAGCCTGACGGAGCAATACCGCGTGAGGGAGGAAGGCTCTTGGG
AB039002      GACACGGCCCAGACTCCTACGGGAGGCAGCAGTGGGGAATTTTCCGCAATGGGCGAAAGCCTGACGGAGCAATACCGCGTGAGGGAGGAAGGCTCTTGGG
AJ293131      GACACGGCCCAGACTCCTACGGGAGGCAGCAGTGGGGAATTTTCCGCAATGGGCGAAAGCCTGACGGAGCAATACCGCGTGAGGGAGGAAGGCTCTTGGG
AJ133169      GACACGGCCCAGACTCCTACGGGAGGCAGCAGTGGGGAATTTTCCGCAATGGGCGAAAGCCTGACGGAGCAAGACCGCGTGGGGGAGGAAGGTTCTTGGA
DQ279769      GACACGGCCCAGACTCCTACGGGAGGCAGCAGTGGGGAATTTTCCGCAATGGGCGCAAGCCTGACGGAGCAAGACCGCGTGGGGGAGGAAGGCTCTTGGG
AJ000714      GACACGGCCCAGACTCCTACGGGAGGCAGCAGTGGGGAATTTTCCGCAATGGGCGCAAGCCTGACGGAGCAAGACCGCGTGGGGGAGGAAAGCCTTTGGG
KVSF5         GACACGGTCCAGACTCCTACGGGAGGCAGCAGTGGGGAATTTTCCGCAATGGGCGAAAGCCTGACGGAGCAAGACCGCGTGGGGGATGAAGGTTTGTGGA

                      410       420       430       440       450       460       470       480       490       500
              ....|....|....|....|....|....|....|....|....|....|....|....|....|....|....|....|....|....|....|....|
KVJ10         TCGTAAACCTCTTTTCTCAGGGAAGAACACAATGACGGTACCTGAGGAATAAGCATCGGCTAACTCCGTGCCAGCAGCCGCGGTAATACGGAGGATGCAA
KVJF8         TCGTAAACCTCTTTTCTCAGGGAAGAACACAATGACGGTACCTGAGGAATAAGCATCGGCTAACTCCGTGCCAGCAGCCGCGGTAATACGGAGGATGCAA
KVJ3          TCGTAAACCTCTTTTCTCAGGGAAGAACAAAATGACGGTACTTGAGGAATAAGCATCGGCTAACTCCGTGCCAGCAGCCGCGGTAATACGGAGGATGCAA
SKS1          TCGTAAACCTCTTTTCTCAGGGAAGAACAAAATGACGGTACTTGAGGAATAAGCATCGGCTAACTCCGTGCCAGCAGCCGCGGTAATACGGAGGATGCAA
KVS1          TCGTAAACCTCTTTTCTCAGGGAAGAACACAATGACGGTACTTGAGGAATAAGCATCGGCTAACTCCGTGCCAGCAGCCGCGGTAATACGGAGGATGCAA
SKJ1          TCGTAAACCTCTTTTCTCAGGGAAGAACACAATGACGGTACTTGAGGAATAAGCATCGGCTAACTCCGTGCCAGCAGCCGCGGTAATACGGAGGATGCAA
KVJF4         TCGTAAACCTCTTTTCTCAGGGAAGAACACAATGACGGTACCTGAGGAATAAGCATCGGCTAACTCCGTGCCAGCAGCCGCGGTAATACGGAGGATGCAA
KVS11         TCGTAAACCTCTTTTCTCAGGGAAGAACAAAATGACGGTACCTGAGGAATAAGCATCGGCTAACTCCGTGCCAGCAGCCGCGGTAATACGGAGGATGCAA
KVJ20         TCGTAAACCTCTTTTCTCAGGGAAGAACACAATGACGGTACCTGAGGAATAAGCATCGGCTAACTCCGTGCCAGCAGCCGCGGTAATACGGAGGATGCAA
SKSF3         TCGTAAACCTCTTTTCTCAGGGAAGAACACAATGACGGTACCTGAGGAATAAGCATCGGCTAACTCCGTGCCAGCAGCCGCGGTAATACGGAGGATGCAA
SKSL1         TCGTAAACCTCTTTTCTCAGGGAAGAACAAAATGACGGTACCTGAGGAATAAGCATCGGCTAACTCCGTGCCAGCAGCCGCGGTAATACGGAGGATGCAA
SKSL2         TCGTAAACCTCTTTTCTCAGGGAAGAACAAAATGACGGTACCTGAGGAATAAGCATCGGCTAACTCCGTGCCAGCAGCCGCGGTAATACGGAGGATGCAA
KVJ4          TCGTAAACCTCTTTTCTCAGGGAAGAACAAAATGACGGTACCTGAGGAATAAGCATCGGCTAACTCCGTGCCAGCAGCCGCGGTAATACGGAGGATGCAA
KVJ2          TCGTAAACCTCTTTTCTCAGGGAAGAACAAAATGACGGTACCTGAGGAATAAGCATCGGCTAACTCCGTGCCAGCAGCCGCGGTAATACGGAGGATGCAA
KVSF4         TCGTAAACCTCTTTTCTCAGGGAAGAACAAAATGACGGTACCTGAGGAATAAGCATCGGCTAACTCCGTGCCAGCAGCCGCGGTAATACGGAGGATGCAA
SKS2          TCGTAAACCTCTTTTCTCAGGGAAGAACAAAATGACGGTACCTGAGGAATAAGCATCGGCTAACTCCGTGCCAGCAGCCGCGGTAATACGGAGGATGCAA
SKJF1         TCGTAAACCTCTTTTCTCAGGGAAGAACACAATGACGGTACCTGAGGAATAAGCATCGGCTAACTCCGTGCCAGCAGCCGCGGTAATACGGAGGATGCAA
SKS3          TCGTAAACCTCTTTTCTCAGGGAAGAACACAATGACGGTACCTGAGGAATAAGCATCGGCTAACTCCGTGCCAGCAGCCGCGGTAATACGGAGGATGCAA
SKJ6          TCGTAAACCTCTTTTCTCAGGGAAGAACACAATGACGGTACCTGAGGAATAAGCATCGGCTAACTCCGTGCCAGCAGCCGCGGTAATACGGAGGATGCAA
SKJ2          TCGTAAACCTCTTTTCTCAAGGAAGAACACAATGACGGTACTTGAGGAATAAGCATCGGCTAACTCCGTGCCAGCAGCCGCGGTAATACGGAGGATGCAA
SKS5          TCGTAAACCTCTTTTCTCAGGGAAGAACACAATGACGGTACCTGAGGAATAAGCATCGGCTAACTCCGTGCCAGCAGCCGCGGTAATACGGAGGATGCAA
SKS9          TCGTAAACCTCTTTTCTCAAGGAAGAACACAATGACGGTACTTGAGGAATAAGCATCGGCTAACTCCGTGCCAGCAGCCGCGGTAATACGGAGGATGCAA
KVJ18         TCGTAAACCTCTTTTCTCAGGGAAGAACAAAATGACGGTACCTGAGGAATAAGCATCGGCTAACTCCGTGCCAGCAGCCGCGGTAATACGGAGGATGCAA
```

```
SKJ4         TCGTAAACCTCTTTTCTCAGGGAAGAACACAATGACGGTACCTGAGGAATAAGCATCGGCTAACTCCGTGCCAGCAGCCGCGGTAATACGGAGGATGCAA
SKSF1        TCGTAAACCTCTTTTCTCAGGGAAGAAAAAAATGACGGTACCTGAGGAATAAGCATCGGCTAACTCCGTGCCAGCAGCCGCGGTAATACGGAGGATGCAA
KVSF7        TTGTAAACCTCTTTTCTCAGGGAAGAAAAAAATGACGGTACCTGAGGAATAAGCATCGGCTAACTCCGTGCCAGCAGCCGCGGTAATACGGAGGATGCAA
SKS8         TCGTAAACCTCTTTTCTCAGGGAAGAAAAAAATGACGGTACCTGAGGAATAAGCATCGGCTAACTCCGTGCCAGCAGCCGCGGTAATACGGAGGATGCAA
DQ185249     TCGTAAACCTCTTTTCTCAGGGAAGAACACAATGACGGTACCTGAGGAATAAGCATCGGCTAACTCCGTGCCAGCAGCCGCGGTAATACGGAGGATGCAA
DQ185255     TCGTAAACCTCTTTTCTCAGGGAAGAACACAATGACGGTACCTGAGGAATAAGCATCGGCTAACTCCGTGCCAGCAGCCGCGGTAATACGGAGGATGCAA
AY566855     TCGTAAACCTCTTTTCTCAGGGAAGAACACAATGACGGTACTTGAGGAATAAGCATCGGCTAACTCCGTGCCAGCAGCCGCGGTAATACGGAGGATGCAA
AM711545     TCGTAAACCTCTTTTCTCAGGGAAGAACAAAATGACGGTACCTGAGGAATAAGCATCGGCTAACTCCGTGCCAGCAGCCGCGGTAATACGGAGGATGCAA
AM711536     TCGTAAACCTCTTTTCTCAGGGAAGAACACAATGACGGTACCTGAGGAATAAGCATCGGCTAACTCCGTGCCAGCAGCCGCGGTAATACGGAGGATGCAA
DQ185256     TCGTAAACCTCTTTTCTCAGGGAAGAACANAATGACGGTACCTGAGGAATAAGCATCGGCTAACTCCGTGCCAGCAGCCGCGGTAATACGGAGGATGCAA
DQ185245     TCGTAAACCTCTTTTCTCAGGGAAGAACANAATGACGGTACCTGAGGAATAAGCATCGGCTAACTCCGTGCCAGCAGCCGCGGTAATACGGAGGATGCAA
AM711527     TCGTAAACCTCTTTTCTCAGGGAAGAACACAATGACGGTACCTGAGGAATAAGCATCGGCTAACTCCGTGCCAGCAGCCGCGGTAATACGGAGGATGCAA
DQ185212     TCGTAAACCTCTTTTCTCAGGGAAGAACACAATGACGGTACCTGAGGAATAAGCATCGGCTAACTCCGTGCCAGCAGCCGCGGTAATACGGAGGATGCAA
AF062638     TCGTAAACCTCTTTTCTCAGGGAAGAACACAATGACGGTACCTGAGGAATAAGCATCGGCTAACTCCGTGCCAGCAGCCGCGGTAATACGGAGGATGCAA
HM623781     TCGTAAACCTCTTTTCTCAGGGAAGAACACAATGACGGTACCTGAGGAATAAGCATCGGCTAACTCCGTGCCAGCAGCCGCGGTAATACGGAGGATGCAA
AM711540     TCGTAAACCTCTTTTCTCAGGGAAGAACACAATGACGGTACCTGAGGAATAAGCATCGGCTAACTCCGTGCCAGCAGCCGCGGTAATACGGAGGATGCAA
DQ185254     TCGTAAACCTCTTTTCTCAGGGAAGAACACAATGACGGTACCTGAGGAATAAGCATCGGCTAACTCCGTGCCAGCAGCCGCGGTAATACGGAGGATGCAA
DQ185251     TCGTAAACCTCTTTTCTCAGGGAAGAACACAATGACGGTACCTGAGGAATAAGCATCGGCTAACTCCGTGCCAGCAGCCGCGGTAATACGGAGGATGCAA
AM711535     TCGTAAACCTCTTTTCTCAGGGAAGAACACAATGACGGTACCTGAGGAATAAGCATCGGCTAACTCCGTGCCAGCAGCCGCGGTAATACGGAGGATGCAA
AF027653     TCGTAAACCTCTTTTCTCAGGGAAGAACACAATGACGGTACCTGAGGAATAAGCATCGGCTAACTCCGTGCCAGCAGCCGCGGTAATACGGAGGATGCAA
DQ185244     TCGTAAACCTCTTTTCTCAGGGAAGAACACAATGACGGTACCTGAGGAATAAGCATCGGCTAACTCCGTGCCAGCAGCCGCGGTAATACGGAGGATGCAA
AM711531     TCGTAAACCTCTTTTCTCAGGGAAGAACAAAATGACGGTACCTGAGGAATAAGCATCGGCTAACTCCGTGCCAGCAGCCGCGGTAATACGGAGGATGCAA
AJ630449     TCGTAAACCTCTTTTCTCAGGGAAGAACACAATGACGGTACCTGAGGAATAAGCATCGGCTAACTCCGTGCCAGCAGCCGCGGTAATACGGAGGATGCAA
AY328897     TCGTAAACCTCTTTTCTCAGGGAAGAACACAATGACGGTACCTGAGGAATAAGCATCGGCTAACTCCGTGCCAGCAGCCGCGGTAATACGGAGGATGCAA
AM230702     TCGTAAACCTCTTTTCTCAAGGAAGATAATGAYGACGGTACCTGAGGAATAAGCATCGGCTAACTCCGTGCCAGCAGCCGCGGTAATACGGAGGATGAAA
EF568907     TCGTAAACCTCTTTTCTCAGGGAAGAAAAAAATGACGGTACCTGAGGAATAAGCATCGGCTAACTCCGTGCCAGCAGCCGCGGTAATACGGAGGATGCAA
AJ630458     TCGTAAACCTCTTTTCTCAGGGAAGAAAAAAATGACGGTACCTGAGGAATAAGCATCGGCTAACTCCGTGCCAGCAGCCGCGGTAATACGGAGGATGCAA
GQ443447     TCGTAAACCTCTTTTCTCAGGGAAGAAAAAAATGACGGTACCTGAGGAATAAGCATCGGCTAACTCCGTGCCAGCAGCCGCGGTAATACGGAGGATGCAA
AJ630457     TTGTAAACCTCTTTTCTCAGGGAAGAAAAAAATGACGGTACCTGAGGAATAAGCATCGGCTAACTCCGTGCCAGCAGCCGCGGTAATACGGAGGATGCAA
DQ234831     TTGTAAACCTCTTTTCTCAGGGAAGAAAAAAATGACGGTACCTGAGGAATAAGCATCGGCTAACTCCGTGCCAGCAGCCGCGGTAATACGGAGGATGCAA
AJ133161     TCGTAAACCTCTTTTCTCAGGGAAGAAAAAAATGACGGTACCTGAGGAATAAGCATCGGCTAACTCCGTGCCAGCAGCCGCGGTAATACGGAGGATGCAA
gi|47118302  TTGTAAACCTCTTTTCTCAGGGAATAAAAAAATGAAGGTACCTGAGGAATAAGCATCGGCTAACTCCGTGCCAGCAGCCGCGGTAATACGGAGGATGCAA
DQ185209     TCGTAAACCTCTTTTCTCAGGGAAGAACACAATGACGGTACCTGAGGAATAAGCATCGGCTAACTCCGTGCCAGCAGCCGCGGTAATACGGAGGATGCAA
AY742451     TCGTAAACCTCTTTTCTCAGGGAAAAACACAATGACGGTACCTGAGGAATAAGCTTCGGCTAACTCCGTGCCAGCAGCCGCGGTAATACGGAGGATGCAA
AF027655     TCGTAAACCTCTTTTCTCAGGGAAGAACACAATGACGGTACCTGAGGAATAAGCATCGGCTAACTCCGTGCCAGCAGCCGCGGTAATACGGAGGATGCAA
DQ185211     TCGTAAACCTCTTTTCTCAGGGAAGAACACAATGACGGTACCTGAGGAATAAGCATCGGCTAACTCCGTGCCAGCAGCCGCGGTAATACGGAGGATGCAA
AY742449     TCGTAAACCTCTTTTCTCAGGGAAGAACACAATGACGGTACCTGAGGAATAAGCATCGGCTAACTCCGTGCCAGCAGCCGCGGTAATACGGAGGATGCAA
EF174212     TCGTAAACCTCTTTTCTCAGGGAAGAACAAAATGACGGTACCTGAGGAATAAGCATCGGCTAACTCCGTGCCAGCAGCCGCGGTAATACGGAGGATGCAA
DQ185213     TCGTAAACCTCTTTTCTCAGGGAAGAACACAATGACGGTACCTGAGGAATAAGCATCGGCTAACTCCGTGCCAGCAGCCGCGGTAATACGGAGGATGCAA
AF506237     TTGTAAACCTCTTTTCTCAGGGAAGA-TAA--TGACGGTACCTGAGGAATAAGCATCGGCTA-CTCCGTGCCAGCAGCCGCGGTAATACGGAGGATGCAA
AM230690     TTGTAAACCTCTTTTCTCTAGGAAGA-TAA--TGACGGTACTAGAGGAATAAGCATCGGCTAACTCCGTGCCAGCAGCCGCGGTAATACGGAGGATGCAA
AB039002     TTGTAAACCTCTTTTCTCAGGGAAGAAAAAAATGACGGTACCTGAGGAATAAGCATCGGCTAACTCCGTGCCAGCAGCCGCGGTAATACGGAGGATGCAA
AJ293131     TTGTAAACCTCTTTTCTCAGGGAAGAACAAAATGACGGTACCTGAGGAATAAGCATCGGCTAACTCCGTGCCAGCAGCCGCGGTAATACGGAGGATGCAA
AJ133169     TTGTCAACCCCTTTTCTCAGGGAAGAACACAATGACGGTACCTGAGGAAAAAGCATCGGCTAACTCCGTGCCAGCAGCCGCGGTAATACGGGGGATGCAA
DQ279769     TTGTAAACCCCTTTTCTCAAGGAAGAACACAATGACGGTACTTGAGGAATAAGCCTCGGCTAACTCCGTGCCAGCAGCCGCGGTAATACGGAGGAGGCAA
AJ000714     TTGTAAACCCCTTTTCTCAGGGAAGAAGAAAGTGACGGTACCTGAGGAATCAGCCTCGGCTAACTCCGTGCCAGCAGCCGCGGTAATACGGAGGAGGCAA
```

```
KVSF5      TTGTAAACCCCTTTTGTCAGGGAAGAATAAC-TGACGGTACCTGACGAATAAGCCTCGGCTAACTCCGTGCCAGCAGCCGCGGTAATACGGAGGAGGCAA

                    510       520       530       540       550       560       570       580       590       600
           ....|....|....|....|....|....|....|....|....|....|....|....|....|....|....|....|....|....|....|....|
KVJ10      GCGTTATCCGGAATGATTGGGCGTAAAGCGTCCGCAGGTGGCAATGTAAGTCTGCTGTTAAAGAGTCTAGCTCAACTAGATAAGAGCAGTGGAAACTACA
KVJF8      GCGTTATCCGGAATGATTGGGCGTAAAGCGTCCGCAGGTGGCAATGTAAGTCTGCTGTTAAAGAGTCTAGCTCAACTAGATAAGAGCAGTGGAAACTACA
KVJ3       GCGTTATCCGGAATGATTGGGCGTAAAGCGTCCGCAGGTGGCAATGTAAGTCTGCTGTTAAAGAGTCTAGCTCAACTAGATAAGAGCAGTGGAAACTACA
SKS1       GCGTTATCCGGAATGATTGGGCGTAAAGCGTCCGCAGGTGGCAATGTAAGTCTGCTGTTAAAGAGTCTAGCTCAACTAGATAAGAGCAGTGGAAACTACA
KVS1       GCGTTATCCGGAATGATTGGGCGTAAAGCGTCCGCAGGTGGCAATGTAAGTCTGCTGTTAAAGAGTCTAGCTCAACTAGATAAGAGCAGTGGAAACTACA
SKJ1       GCGTTATCCGGAATGATTGGGCGTAAAGCGTCCGCAGGTGGCAATGTAAGTCTGCTGTTAAAGAGTCTAGCTCAACTAGATAAGAGCAGTGGAAACTACA
KVJF4      GCGTTATCCGGAATGATTGGGCGTAAAGCGTCCGCAGGTGGCAATGTAAGTCTGCTGTTAAAGAGTCTAGCTCAACTAGATAAAAGCAGTGGAAACTACA
KVS11      GCGTTATCCGGAATGATTGGGCGTAAAGCGTCCGCAGGTGGCAATGTAAGTCTGCTGTTAAAGAGTCTAGCTCAACTAGATAAAAGCAGTGGAAACTACA
KVJ20      GCGTTATCCGGAATGATTGGGCGTAAAGCGTCCGCAGGTGGCTATGTAAGTCTGCTGTTAAAGAGTCTAGCTCAACTAGATAAGAGCAGTGGAAACTACA
SKSF3      GCGTTATCCGGAATGATTGGGCGTAAAGCGTCCGCAGGTGGCTATGTAAGTCTGCTGTTAAAGAGTCTAGCTCAACTAGATAAGAGCAGTGGAAACTACA
SKSL1      GCGTTATCCGGAATGATTGGGCGTAAAGCGTCCGCAGGTGGCTATGTAAGTCTGCTGTTAAAGAGTCTAGCTCAACTAGATAAGAGCAGTGGAAACTACA
SKSL2      GCGTTATCCGGAATGATTGGGCGTAAAGCGTCCGCAGGTGGCTATGTAAGTCTGCTGTTAAAGAGTCTAGCTCAACTAGATAAGAGCAGTGGAAACTACA
KVJ4       GCGTTATCCGGAATGATTGGGCGTAAAGCGTCCGCAGGTGGCTATGTAAGTCTGCTGTTAAAGAGTCTAGCTCAACTAGATAAGAGCAGTGGAAACTACA
KVJ2       GCGTTATCCGGAATGATTGGGCGTAAAGCGTCCGCAGGTGGCTATGTAAGTCTGCTGTTAAAGAGTCTAGCTCAACTAGATAAGAGCAGTGGAAACTACA
KVSF4      GCGTTATCCGGAATGATTGGGCGTAAAGCGTCCGCAGGTGGCTATATAAGTCTGCTGTTAAAGAGTCTAGCTCAACTAGATAAGAGCAGTGGAAACTACA
SKS2       GCGTTATCCGGAATGATTGGGCGTAAAGCGTCCGCAGGTGGCTATGTAAGTCTGCTGTTAAAGAGTCTAGCTCAACTAGATAAGAGCAGTGGAAACTACA
SKJF1      GCGTTATCCGGAATGATTGGGCGTAAAGCGTCCGCAGGTGGCAATGTAAGTCTGCTGTTAAAGAGTCTAGCTCAACTAGATAAGAGCAGTGGAAACTACA
SKS3       GCGTTATCCGGAATGATTGGGCGTAAAGCGTCCGCAGGTGGCAATGTAAGTCTGCTGTTAAAGAGTCTAGCTCAACTAGATAAGAGCAGTGGAAACTACA
SKJ6       GCGTTATCCGGAATGATTGGGCGTAAAGCGTCCGCAGGTGGCAATGTAAGTCTGCTGTTAAAGAGTCTAGCTCAACTAGATAAGAGCAGTGGAAACTACA
SKJ2       GCGTTATCCGGAATGATTGGGCGTAAAGCGTCCGCAGGTGGCAATGTAAGTCTGCTGTTAAAGAGTCTAGCTCAACTAGATAAGAGCAGTGGAAACTACA
SKS5       GCGTTATCCGGAATGATTGGGCGTAAAGCGTCCGCAGGTGGCAATGTAAGTCTGCTGTTAAAGAGTCTAGCTCAACTAGATAAGAGCAGTGGAAACTACA
SKS9       GCGTTATCCGGAATGATTGGGCGTAAAGCGTCCGCAGGTGGCAATGTAAGTCTGCTGTTAAAGAGTCTAGCTCAACTAGATAAGAGCAGTGGAAACTACA
KVJ18      GCGTTATCCGGAATGATTGGGCGTAAAGCGTCCGCAGGTGGCTATGTAAGTCTGCTGTTAAAGAGTCTAGCTCAACTAGATAAGAGCAGTGGAAACTACA
SKJ4       GCGTTATCCGGAATGATTGGGCGTAAAGCGTCCGCAGGTGGCAATGTAAGTCTGCTGTTAAAGAGTCTAGCTCAACTAGATAAGAGCAGTGGAAACTACA
SKSF1      GCGTTATCCGGAATGATTGGGCGTAAAGGGTCCGCAGGTTGCCATGTAAGTCTGCTGTTAAAGAGTGAGGCTCAACCTCATAAAAGCAGTGGAAACTACA
KVSF7      GCGTTATCCGGAATGATTGGGCGTAAAGGGTCCGCAGGTGGCTGTGTAAGTCTGCTGTTAAATAGTCATGCTCAACATGATAAAGGCAGTGGAAACTACA
SKS8       GCGTTATCCGGAATGATTGGGCGTAAAGCGTCCGCAGGTGGCTGTGTAAGTCTGCTGTTAAAGAGTCATGCTCAACATGATAAGAGCAGTGGAAACTACA
DQ185249   GCGTTATCCGGAATGATTGGGCGTAAAGCGTCCGCAGGTGGCAATGTAAGTCTGCTGTTAAAGAGTCTAGCTCAACTAGATAAGAGCAGTGGAAACTACA
DQ185255   GCGTTATCCGGAATGATTGGGCGTAAAGCGTCCGCAGGTGGCAATGTAAGTCTGCTGTTAAAGAGTCTAGCTCAACTAGATAAGAGCAGTGGAAACTACA
AY566855   GCGTTATCCGGAATGATTGGGCGTAAAGCGTCCGCAGGTGGCAATGTAAGTCTGCTGTTAAAGAGTCTAGCTCAACTAGATAAGAGCAGTGGAAACTACA
AM711545   GCGTTATCCGGAATGATTGGGCGTAAAGCGTCCGCAGGTGGCAATGTAAGTCTGCTGTTAAAGAGTCTAGCTCAACTAGATAAAAGCAGTGGAAACTACA
AM711536   GCGTTATCCGGAATGATTGGGCGTAAAGCGTCCGCAGGTGGCAATGTAAGTCTGCTGTTAAAGAGTCTAGCTCAACTAGATAAAAGCAGTGGAAACTACA
DQ185256   GCGTTATCCGGAATGATTGGGCGTAAAGCGTCCGCAGGTGGCAATGTAAGTCTGCTGTTAAAGAGTCTAGCTCAACTAGATAANAGCAGTGGAAACTACA
DQ185245   GCGTTATCCGGAATGATTGGGCGTAAAGCGTCCGCAGGTGGCAATGTAAGTCTGCTGTTAAAGAGTCTAGCTCAACTAGATAAAAGCAGTGGAAACTACA
AM711527   GCGTTATCCGGAATGATTGGGCGTAAAGCGTCCGCAGGTGGCAATGTAAGTCTGCTGTTAAAGAGTCTAGCTCAACTAGATAAAAGCAGTGGAAACTACA
DQ185212   GCGTTATCCGGAATGATTGGGCGTAAAGCGTCCGCAGGTGGCAATGTAAGTCTGCTGTTAAAGAGTCTAGCTCAACTAGATAAAAGCAGTGGAAACTACA
AF062638   GCGTTATCCGGAATGATTGGGCGTAAAGCGTCCGCAGGTGGCAATGTAAGTCTGCTGTTAAAGAGTCTAGCTCAACTAGATAAAAGCAGTGGAAACTACA
HM623781   GCGTTATCCGGAATGATTGGGCGTAAAGCGTCCGCAGGTGGCTATGTAAGTCTGCTGTTAAAGAGTCTAGCTCAACTAGATAAGAGCAGTGGAAACTACA
AM711540   GCGTTATCCGGAATGATTGGGCGTAAAGCGTCCGCAGGTGGCTATGTAAGTCTGCTGTTAAAGAGTCTAGCTCAACTAGATAAGAGCAGTGGAAACTACA
DQ185254   GCGTTATCCGGAATGATTGGGCGTAAAGCGTCCGCAGGTGGCTATGTAAGTCTGCTGTTAAAGAGTCTAGCTCAACTAGATAAGAGCAGTGGAAACTACA
DQ185251   GCGTTATCCGGAATGATTGGGCGTAAAGCGTCCGCAGGTGGCTATGTAAGTCTGCTGTTAAAGAGTCTAGCTCAACTAGATAAGAGCAGTGGAAACTACA
AM711535   GCGTTATCCGGAATGATTGGGCGTAAAGCGTCCGCAGGTGGCTATGTAAGTCTGCTGTTAAAGAGTCTAGCTCAACTAGATAAGAGCAGTGGAAACTACA
```

```
AF027653     GCGTTATCCGGAATGATTGGGCGTAAAGCGTCCGCAGGTGGCTATGTAAGTCTGCTGTTAAAGAGTCTAGCTCAACTAGATAAGAGCAGTGGAAACTACA
DQ185244     GCGTTATCCGGAATGATTGGGCGTAAAGCGTCCGCAGGTGGCTATGTAAGTCTGCTGTTAAAGAGTCTAGCTCAACTAGATAAGAGCAGTGGAAACTACA
AM711531     GCGTTATCCGGAATGATTGGGCGTAAAGCGTCCGCAGGTGGCTATGTAAGTCTGCTGTTAAAGAGTCTAGCTCAACTAGATAAGAGCAGTGGAAACTACA
AJ630449     GCGTTATCCGGAATGATTGGGCGTAAAGCGTCCGCAGGTGGCTATGTAAGTCTGCTGTTAAAGAGTCTAGCTCAACTAGATAAAAGCAGTGGAAACTACA
AY328897     GCGTTATCCGGAATGATTGGGCGTAAAGCGTCCGCAGGTGGCAATGTAAGTCTGCTGTTAAAGAGTCTAGCTCAACTAGATAAGAGCAGTGGAAACTACA
AM230702     GCGTTATCCGGAATGATTGGGCGTAAAGCGTCCGCAGGTTGYTATGTAAGTCTGCTGTTAAAGAGTGMGGCTCAACCTCATARGAGCAGTGGAAACTACA
EF568907     GCGTTATCCGGAATGATTGGGCGTAAAGGGTCCGCAGGTTGCAATGTAAGTCTGCTGTTAAAGAATGAGGCTCAACCTCATAAGAGCAGTGGAAACTACA
AJ630458     GCGTTATCCGGAATGATTGGGCGTAAAGCGTCCGCAGGTGGCCATGTAAGTCTGCTGTTAAAGAGTCATGCTCAACATGATAAAAGCAGTGGAAACTACA
GQ443447     GCGTTATCCGGAATGATTGGGCGTAAAGCGTCCGCAGGTGGCCATGTAAGTCTGCTGTTAAAGAGTCATGCTTAACATGATAAAAGCAGTGGAAACTACA
AJ630457     GCGTTATCCGGAATGATTGGGCGTAAAGGGTCCGCAGGTGGCTGTGTAAGTCTGCTGTTAAATAGTCATGCTCAACATGATAAAGGCAGTGGAAACTACA
DQ234831     GCGTTATCCGGAATGATTGGGCGTAAAGGGTCCGCAGGTGGCTGTGTAAGTCTGCTGTTAAATAGTCATGCTCAACATGATAAAGGCAGTGGAAACTACA
AJ133161     GCGTTATCCGGAATGATTGGGCGTAAAGCGTCCGCAGGTGGCTGTGTAAGTCTGCTGTTAAAGAGTCATGCTCAACATGATAAGAGCAGTGGAAACTACA
gi|47118302  GCGTTATCCGGAATGATTGGGCGTAAAGCGTCCGCAGGTGGCACTGTAAGTCTGCTGTTAAAGAGCAAGGCTCAACCTTGTAAAGGCAGTGGAAACTACA
DQ185209     GCGTTATCCGGAATGATTGGGCGTAAAGCGTCCGCAGGTGGCAATGTAAGTCTGCTGTTAAAGAGTCTAGCTCAACTAGATAAGAGCAGTGGAAACTACA
AY742451     GCGTTATCCGGAATGATTGGGCGTAAAGCGTCCGCAGGTGGCTATGTAAGTCTGCTGTTAAAGAGTCTAGCTCAACTAGATAAGAGCAGTGGAAACTACA
AF027655     GCGTTATCCGGAATGATTGGGCGTAAAGCGTCCGCAGGTGGCAATGTAAGTCTGCTGTTAAAGAGTCTAGCTCAACTAGATACAAGCAGTGGAAACTACA
DQ185211     GCGTTATCCGGAATGATTGGGCGTAAAGCGTCCGCAGGTGGCAATGTAAGTCTGCTGTTAAAGAGTCTAGCTCAACTAGATACAAGCAGTGGAAACTACA
AY742449     GCGTTATCCGGAATGATTGGGCGTAAAGCGTCCGCAGGTGGCAATGTAAGTCTGCTGTTAAAGAGTCTAGCTCAACTAGATAAAAGCAGTGGAAACTACA
EF174212     GCGTTATCCGGAATGATTGGGCGTAAAGCGTCCGCAGGTGGCTATGTAAGTCTGCTGTTAAAGAGTCTAGCTCAACTAGATAAGAGCAGTGGAAACTACA
DQ185213     GCGTTATCCGGAATGATTGGGCGTAAAGCGTCCGCAGGTGGCAATGTAAGTCTGCTGTTAAAGAGTCTAGCTCAACTAGATAAGAGCAGTGGAAACTACA
AF506237     GCGTTATCCGGAATGATTGGGCGTAAAGCGTCCGCAGGTGGTTAAGAAAGTCTGTTGTTAAAGMGTGAGGCTTAACCTCATAAGGGCAATGGAAACTGTT
AM230690     GCGTTATCCGGAATGATTGGGCGTAAAGCGTCCGCAGGTGGTTTTAAAAGTCTGTTGTTAAAGCGTGAGGCTTAACCTCATAAGGGCAATGGAAACTATA
AB039002     GCGTTATCCGGAATGATTGGGCGTAAAGGGTCCGCAG-TGGCTATGTAAGTCTGCTGTTAAAGAACCTAGCTTAACTAGGTAAAAGCAGTGGAAACTACA
AJ293131     GCGTTATCCGGAATGATTGGGCGTAAAGGGTCCGCAGGTGGCATTGTAAGTCTGCTGTTAAAGAGTTTGGCTCAACCAAATAAAAGCAGTGGAAACTACA
AJ133169     GCGTTATCCGGAATGATTGGGCGTAAAGAGTCCGTAGGTAGTCATCCAAGTCTGCTGTTAAAGAGCGAGGCTTAACCTCGTAAAGGCAGTGGAAACTGGA
DQ279769     GCGTTATCCGGAATGATTGGGCGTAAAGCGTCCGTAGGTGGCTGTTCAAGTCTGCTGTCAAAGACAGTGGCTTAACTACTGAAAGGCAGAGGAAACTGAA
AJ000714     GCGTTATCCGGAATGATTGGGCGTAAAGCGTTCGTAGGTGGCAATTCAAGTCTGCTGTCAAAGACAGTAGCTCAACTACTGCAAGGCAGTGGAAACTGAA
KVSF5        GCGTTATCCGGAATTATTGGGCGTAAAGCGTCCGTAGGTGGTTTATCAAGTCTGTTGTTAAAGAGTGGGGCTTAACCTCATAAAGGCAATGGAAACTGAT

                       610       620       630       640       650       660       670       680       690       700
             ....|....|....|....|....|....|....|....|....|....|....|....|....|....|....|....|....|....|....|....|
KVJ10        TAGCTAGAGTACGTTCGGGGCAGAGGGAATTCCTGGTGTAGCGGTGAAATGCGTAGAGATCAGGAAGAACACCAGTGGCGAAGGCGCTCTGCTAGGCCGT
KVJF8        TAGCTAGAGTACGTTCGGGGCAGAGGGAATTCCTGGTGTAGCGGTGAAATGCGTAGAGATCAGGAAGAACACCAGTGGCGAAGGCGCTCTGCTAGGCCGT
KVJ3         TAGCTAGAGTACGTTCGGGGCAGAGGGAATTCCTGGTGTAGCGGTGAAATGCGTAGAGATCAGGAAGAACACCGGTGGCGAAGGCGCTCTGCTAGGCCGT
SKS1         TAGCTAGAGTACGTTCGGGGCAGAGGGAATTCCTGGTGTAGCGGTGAAATGCGTAGAGATCAGGAAGAACACCGGTGGCGAAGGCGCTCTGCTAGGCCGT
KVS1         TAGCTAGAGTACGTTCGGGGCAGAGGGAATTCCTGGTGTAGCGGTGAAATGCGTAGAGATCAGGAAGAACACCGGTGGCGAAGGCGCTCTGCTAGGCCGT
SKJ1         TAGCTAGAGTACGTTCGGGGCAGAGGGAATTCCTGGTGTAGCGGTGAAATGCGTAGAGATCAGGAAGAACACCGGTGGCGAAGGCGCTCTGCTAGGCCGT
KVJF4        TAGCTAGAGTACGTTCGGGGCAGAGGGAATTCCTGGTGTAGCGGTGAAATGCGTAGAGATCAGGAAGAACACCGGTGGCGAAGGCGCTCTGCTAGGCCGT
KVS11        TAGCTAGAGTACGTTCGGGGCAGAGGGAATTCCTGGTGTAGCGGTGAAATGCGTAGAGATCAGGAAGAACACCGGTGGCGAAGGCGCTCTGCTAGGCCGT
KVJ20        TGGCTAGAGTGCGTTCGGGGCAGAGGGAATTCCTGGTGTAGCGGTGAAATGCGTAGAGATCAGGAAGAACACCGGTGGCGAAGGCGCTCTGCTAGGCCGC
SKSF3        TGGCTAGAGTGCGTTCGGGGCAGAGGGAATTCCTGGTGTAGCGGTGAAATGCGTAGAGATCAGGAAGAACACCGGTGGCGAAGGCGCTCTGCTAGGCCGC
SKSL1        AGGCTAGAGTACGTTCGGGGCAGAGGGAATTCCTGGTGTAGCGGTGAAATGCGTAGAGATCAGGAAGAACACCGGTGGCGAAGGCGCTCTGCTAGGCCGT
SKSL2        AGGCTAGAGTACGTTCGGGGCAGAGGGAATTCCTGGTGTAGCGGTGAAATGCGTAGAGATCAGGAAGAACACCGGTGGCGAAGGCGCTCTGCTAGGCCGT
KVJ4         AGGCTAGAGTACGTTCGGGGCAGAGGGAATTCCTGGTGTAGCGGTGAAATGCGTAGAGATCAGGAAGAACACCGGTGGCGAAGGCGCTCTGCTAGGCCGT
KVJ2         AGGCTAGAGTACGTTCGGGGCAGAGGGAATTCCTGGTGTAGCGGTGAAATGCGTAGAGATCAGGAAGAACACCGGTGGCGAAGGCGCTCTGCTAGGCCGT
KVSF4        AGGCTAGAGTACGTTCGGGGCAGAGGGAATTCCTGGTGTAGCGGTGAAATGCGTAGAGATCAGGAAGAACACCGGTGGCGAAGGCGCTCTGCTAGGCCGT
```

```
SKS2         AGGCTAGAGTACGTTCGGGGCAGAGGGAATTCCTGGTGTAGCGGTGAAATGCGTAGAGATCAGGAAGAACACCGGTGGCGAAGGCGCTCTGCTAGGCCGT
SKJF1        TAGCTAGAGTACGTTCGGGGCAGAGGGAATTCCTGGTGTAGCGGTGAAATGCGTAGAGATCAGGAAGAACACCGGTGGCGAAGGCGCTCTGCTAGGCCGT
SKS3         TAGCTAGAGTACGTTCGGGGCAGAGGGAATTCCTGGTGTAGCGGTGAAATGCGTAGAGATCAGGAAGAACACCGGTGGCGAAGGCGCTCTGCTAGGCCGT
SKJ6         TAGCTAGAGTACGTTCGGGGCAGAGGGAATTCCTGGTGTAGCGGTGAAATGCGTAGAGATCAGGAAGAACACCGGTGGCGAAGGCGCTCTGCTAGGCCGT
SKJ2         TAGCTAGAGTACGTTCGGGGCAGAGGGAATTCCTGGTGTAGCGGTGAAATGCGTAGAGATCAGGAAGAACACCGGTGGCGAAGGCGCTCTGCTAGGCCGT
SKS5         TAGCTAGAGTACGTTCGGGGCAGAGGGAATTCCTGGTGTAGCGGTGAAATGCGTAGAGATCAGGAAGAACACCGGTGGCGAAGGCGCTCTGCTAGGCCGT
SKS9         TAGCTAGAGTACGTTCGGGGCAGAGGGAATTCCTGGTGTAGCGGTGAAATGCGTAGAGATCAGGAAGAACACCGGTGGCGAAGGCGCTCTGCTAGGCCGT
KVJ18        AGGCTAGAGTACGTTCGGGGCAGAGGGAATTCCTGGTGTAGCGGTGAAATGCGTAGAGATCAGGAAGAACACCGGTGGCGAAGGCGCTCTGCTAGGCCGT
SKJ4         TAGCTAGAGTACGTTCGGGGCAGAGGGAATTCCTGGTGTAGCGGTGAAATGCGTAGAGATCAGGAAGAACACCGGTGGCGAAGGCGCTCTGCTAGGCCGT
SKSF1        CGGCTAGAGTACGTTCGGGGCAGAGGGAATTCCTGGTGTAGCGGTGAAATGCGTAGATATCAGGAAGAACACCGGTGGCGAAAGCGCTCTGCTAGGCCGT
KVSF7        TAGCTAGAGTGCGTTCGGGGCAGAGGGAATTCCTGGTGTAGCGGTGAAATGCGTAGATATCAGGAAGAACACCGGTGGCGAAAGCGCTCTGCTAGGCCGC
SKS8         CGGCTAGAGTGCGTTCGGGGCAGAGGGAATTCCTGGTGTAGCGGTGAAATGCGTAGAGATCAGGAAGAACACCGGTGGCGAAAGCGCTCTGCTAGGCCGC
DQ185249     TAGCTAGAGTACGTTCGGGGCAGAGGGAATTCCTGGTGTAGCGGTGAAATGCGTAGAGATCAGGAAGAACACCAGTGGCGAAGGCGCTCTGCTAGGCCGT
DQ185255     TAGCTAGAGTACGTTCGGGGCAGAGGGAATTCCTGGTGTAGCGGTGAAATGCGTAGAGATCAGGAAGAACACCAGTGGCGAAGGCGCTCTGCTAGGCCGT
AY566855     TAGCTAGAGTACGTTCGGGGCAGAGGGAATTCCTGGTGTAGCGGTGAAATGCGTAGAGATCAGGAAGAACACCGGTGGCGAAGGCGCTCTGCTAGGCCGT
AM711545     TAGCTAGAGTACGTTCGGGGCAGAGGGAATTCCTGGTGTAGCGGTGAAATGCGTAGAGATCAGGAAGAACACCGGTGGCGAAGGCGCTCTGCTAGGCCGT
AM711536     TAGCTAGAGTACGTTCGGGGCAGAGGGAATTCCTGGTGTAGCGGTGAAATGCGTAGAGATCAGGAAGAACACCGGTGGCGAAGGCGCTCTGCTAGGCCGT
DQ185256     TAGCTAGAGTACGTTCGGGGCAGAGGGAATTCCTGGTGTAGCGGTGAAATGCGTAGAGATCAGGAAGAACACCGGTGGCGAAGGCGCTCTGCTAGGCCGT
DQ185245     TAGCTAGAGTACGTTCGGGGCAGAGGGAATTCCTGGTGTAGCGGTGAAATGCGTAGAGATCAGGAAGAACACCGGTGGCGAAGGCGCTCTGCTAGGCCGT
AM711527     TAGCTAGAGTACGTTCGGGGCAGAGGGAATTCCTGGTGTAGCGGTGAAATGCGTAGAGATCAGGAAGAACACCGGTGGCGAAGGCGCTCTGCTAGGCCGT
DQ185212     TAGCTAGAGTACGTTCGGGGCAGAGGGAATTCCTGGTGTAGCGGTGAAATGCGTAGAGATCAGGAAGAACACCGGTGGCGAAGGCGCTCTGCTAGGCCGT
AF062638     TAGCTAGAGTACGTTCGGGGCAGAGGGAATTCCTGGTGTAGCGGTGAAATGCGTAGAGATCAGGAAGAACACCGGTGGCGAAGGCGCTCTGCTAGGCCGT
HM623781     TGGCTAGAGTGCGTTCGGGGCAGAGGGAATTCCTGGTGTAGCGGTGAAATGCGTAGAGATCAGGAAGAACACCGGTGGCGAAGGCGCTCTGCTAGGCCGC
AM711540     TGGCTAGAGTGCGTTCGGGGCAGAGGGAATTCCTGGTGTAGCGGTGAAATGCGTAGAGATCAGGAAGAACACCGGTGGCGAAGGCGCTCTGCTAGGCCGC
DQ185254     TGGCTAGAGTGCGTTCGGGGCAGAGGGAATTCCTGGTGTAGCGGTGAAATGCGTAGAGATCAGGAAGAACACCGGTGGCGAAGGCGCTCTGCTAGGCCGC
DQ185251     TGGCTAGAGTGCGTTCGGGGCAGAGGGAATTCCTGGTGTAGCGGTGAAATGCGTAGAGATCAGGAAGAACACCGGTGGCGAAGGCGCTCTGCTAGGCCGC
AM711535     TGGCTAGAGTGCGTTCGGGGCAGAGGGAATTCCTGGTGTAGCGGTGAAATGCGTAGAGATCAGGAAGAACACCGGTGGCGAAGGCGCTCTGCTAGGCCGC
AF027653     AGGCTAGAGTACGTTCGGGGCAGAGGGAATTCCTGGTGTAGCGGTGAAATGCGTAGAGATCAGGAAGAACACCGGTGGCGAAGGCGCTCTGCTAGGCCGT
DQ185244     AGGCTAGAGTACGTTCGGGGCAGAGGGAATTCCTGGTGTAGCGGTGAAATGCGTAGAGATCAGGAAGAACACCGGTGGCGAAGGCGCTCTGCTAGGCCGT
AM711531     AGGCTAGAGTACGTTCGGGGCAGAGGGAATTCCTGGTGTAGCGGTGAAATGCGTAGAGATCAGGAAGAACACCGGTGGCGAAGGCGCTCTGCTAGGCCGT
AJ630449     AGGCTAGAGTACGTTCGGGGCAGAGGGAATTCCTGGTGTAGCGGTGAAATGCGTAGAGATCAGGAAGAACACCGGTGGCGAAGGCGCTCTGCTAGGCCGT
AY328897     TAGCTAGAGTACGTTCGGGGCAGAGGGAATTCCTGGTGTAGCGGTGAAATGCGTAGAGATCAGGAAGAACACCGGTGGCGAAGGCGCTCTGCTAGGCCGT
AM230702     TAGCTAGAGTACGTTCGGGGCAGAGGGAATTCCTGGTGTAGCGGTGAAATGCGTAGATATCGGGAAGAACACCGGTGGCGAAAGCGCTCTGCTAGGCCGT
EF568907     TAGCTAGAGTACGTTCGGGGCAGAGGGAATTCCTGGTGTAGCGGTGAAATGCGTAGATATCAGGAAGAACACCGGTGGCGAAAGCGCTCTGCTAGGCCGT
AJ630458     CGGCTAGAGTACGTTCGGGGCAGAGGGAATTCCTGGTGTAGCGGTGAAATGCGTAGATATCAGGAAGAACACCGGTGGCGAAAGCGCTCTGCTAGGCCGT
GQ443447     GAGCTAGAGTACGTTCGGGGCAGAGGGAATTCCTGGTGTAGCGGTGAAATGCGTAGATATCAGGAAGAACACCGGTGGCGAAAGCGCTCTGCTAGGCCGT
AJ630457     TAGCTAGAGTGCGTTCGGGGCAGAGGGAATTCCTGGTGTAGCGGTGAAATGCGTAGATATCAGGAAGAACACCGGTGGCGAAAGCGCTCTGCTAGGCCGC
DQ234831     TAGCTAGAGTGCGTTCGGGGCAGAGGGAATTCCTGGTGTAGCGGTGAAATGCGTAGATATCAGGAAGAACACCGGTGGCGAAAGCGCTCTGCTAGGCCGC
AJ133161     CGGCTAGAGTGCGTTCGGGGCAGAGGGAATTCCTGGTGTAGCGGTGAAATGCGTAGAGATCAGGAAGAACACCGGTGGCGAAAGCGCTCTGCTAGGCCGC
gi|47118302  GAGCTAGAGTACGTTCGGGGCAGAGGGAATTCCTGGTGTAGCGGTGAAATGCGTAGAGATCAGGAAGAACACCGGTGGCGAAAGCGCTCTGCTAGGCCGT
DQ185209     TAGCTAGAGTACGTTCGGGGCAGAGGGAATTCCTGGTGTAGCGGTGAAATGCGTAGAGATCAGGAAGAACACCAGTGGCGAAGGCGCTCTGCTAGGCCGT
AY742451     TGGCTAGAGTGCGTTCGGGGCAGAGGGAATTCCTGGTGTAGCGGTGAAATGCGTAGAGATCAGGAAGAACACCGGTGGCGAAGGCGCTCTGCTAGGCCGC
AF027655     TAGCTAGAGTACGTTCGGGGCAGAGGGAATTCCTGGTGTAGCGGTGAAATGCGTAGAGATCAGGAAGAACACCGGTGGCGAAGGCGCTCTGCTAGGCCGT
DQ185211     TAGCTAGAGTACGTTCGGGGCAGAGGGAATTCCTGGTGTAGCGGTGAAATGCGTAGAGATCAGGAAGAACACCGGTGGCGAAGGCGCTCTGCTAGGCCGT
AY742449     TAGCTAGAGTACGTTTGGGGCAGAGGGAATTCCTGGTGTAGCGGTGAAATGCGTAGAGATCAGGAAGAACACCGGTGGCGAAGGCGCTCTGCTAGGCCGT
EF174212     AGGCTAGAGTGCGTTCGGGGCAGAGGGAATTCCTGGTGTAGCGGTGAAATGCGTAGAGATCAGGAAGAACACCGGTGGCGAAGGCGCTCTGCTAGGCCGC
```

```
DQ185213   TAGCTAGAGTACGTTCGGGGCAGAGGGAATTCCTGGTGTAGCGGTGAAATGCGTAGAGATCAGGAAGAACACCGGTGGCGAAGGCGCTCTGCTAGGCCGT
AF506237   TGACTAGAGTACGTTCGGGGCAAGGGGAATTCCCAGTGTAGCGGTGAAATGCGTAGAGATTGGGAAGAACACCGGTGGCGAAAGCGCCTTGCTAGGCCGT
AM230690   AGACTAGAGTACGTTCGGGGCAAGGGGAATTCCCAGTGTAGCGGTGAAATGCGTAGAGATTGGGAAGAACACCGGTGGCGAAAGCGCCTTGCTAGGCCGT
AB039002   AGGCTAGAGTGCGTTCGGGGTAGAGGGAATTCCTGGTGTAGCGGTGAAATGCGTAGATATCAGGAAGAACACCAGTGGCGAAGGCGCTCTACTAGGCCGC
AJ293131   AAGCTAGAGTGTGGTCGGGGCAGAGGGAATTCCTGGTGTAGCGGTGAAATGCGTAGATATCAGGAAGAACACCGGTGGCGAAGGCGCTCTGCTAGGCCAA
AJ133169   AGACTAGAGTGTAGTAGGGGCAGAGGGAATTCCTGGTGTAGCGGTGAAATGCGTAGAGATCAGGAAGAACACCGGTGGCGAAGGCGCTCTGCTGGGCTAT
DQ279769   CAGCTAGAGTACGGTAGGGGCAGAGGGAATTCCCGGTGTAGCGGTGAAATGCGTAGATATCGGGAAGAACACCGGTGGCGAAAGCGCTCTGCTGGGCCGT
AJ000714   TAGCTAGAGTGCGGTAGGGGCAGAGGGAATTCCCGGTGTAGCGGTGAAATGCGTAGAGATCGGGAAGAACACCGGTGGCGAAAGCGCTCTGCTGGGCCGC
KVSF5      GGACTTGAGTGCGGTAGGGGTAGAGGGAATTCCCAGTGTAGCGGTGAAATGCGTAGATATTGGGAAGAACACCGGTGGCGAAAGCGCTCTACTGGACCTG

                     710       720       730       740       750       760       770       780       790       800
           ....|....|....|....|....|....|....|....|....|....|....|....|....|....|....|....|....|....|....|....|
KVJ10      AACTGACACTGAGGGACGAAAGCTAGGGGAGCGAATGGGATTAGATACCCCAGTAGTCCTAGCCGTAAACGATGGATACTAGGCGTGGCTTGTATCGACC
KVJF8      AACTGACACTGAGGGACGAAAGCTAGGGGAGCGAATGGGATTAGATACCCCAGTAGTCCTAGCCGTAAACGATGGATACTAGGCGTGGCTTGTATCGACC
KVJ3       AACTGACACTGAGGGACGAAAGCTAGGGGAGCGAATGGGATTAGATACCCCAGTAGTCCTAGCCGTAAACGATGGATACTAGGCGTGGCTTGTATCGACC
SKS1       AACTGACACTGAGGGACGAAAGCTAGGGGAGCGAATGGGATTAGATACCCCAGTAGTCCTAGCCGTAAACGATGGATACTAGGCGTGGCTTGTATCGACC
KVS1       AACTGACACTGAGGGACGAAAGCTAGGGGAGCGAATGGGATTAGATACCCCAGTAGTCCTAGCCGTAAACGATGGATACTAGGCGTGGCTTGTATCGACC
SKJ1       AACTGACACTGAGGGACGAAAGCTAGGGGAGCGAATGGGATTAGATACCCCAGTAGTCCTAGCCGTAAACGATGGATACTAGGCGTGGCTTGTATCGACC
KVJF4      AACTGACACTGAGGGACGAAAGCTAGGGGAGCGAATGGGATTAGATACCCCAGTAGTCCTAGCCGTAAACGATGGATACTAGGCGTGGCTTGTATCGACC
KVS11      AACTGACACTGAGGGACGAAAGCTAGGGGAGCGAATGGGATTAGATACCCCAGTAGTCCTAGCCGTAAACGATGGATACTAGGCGTGGCTTGTATCGACC
KVJ20      AACTGACACTGAGGGACGAAAGCTAGGGGAGCGAATGGGATTAGATACCCCAGTAGTCCTAGCCGTAAACGATGGATACTAGGCGTGGCTTGTATCGACC
SKSF3      AACTGACACTGAGGGACGAAAGCTAGGGGAGCGAATGGGATTAGATACCCCAGTAGTCCTAGCCGTAAACGATGGATACTAGGCGTGGCTTGTATCGACC
SKSL1      AACTGACACTGAGGGACGAAAGCTAGGGGAGCGAATGGGATTAGATACCCCAGTAGTCCTAGCCGTAAACGATGGATACTAGGCGTGGCTTGTATCGACC
SKSL2      AACTGACACTGAGGGACGAAAGCTAGGGGAGCGAATGGGATTAGATACCCCAGTAGTCCTAGCCGTAAACGATGGATACTAGGCGTGGCTTGTATCGACC
KVJ4       AACTGACACTGAGGGACGAAAGCTAGGGGAGCGAATGGGATTAGATACCCCAGTAGTCCTAGCCGTAAACGATGGATACTAGGCGTGGCTTGTATCGACC
KVJ2       AACTGACACTGAGGGACGAAAGCTAGGGGAGCGAATGGGATTAGATACCCCAGTAGTCCTAGCCGTAAACGATGGATACTAGGCGTGGCTTGTATCGACC
KVSF4      AACTGACACTGAGGGACGAAAGCTAGGGGAGCGAATGGGATTAGATACCCCAGTAGTCCTAGCCGTAAACGATGGATACTAGGCGTGGCTTGTATCGACC
SKS2       AACTGACACTGAGGGACGAAAGCTAGGGGAGCGAATGGGATTAGATACCCCAGTAGTCCTAGCCGTAAACGATGGATACTAGGCGTGGCTTGTATCGACC
SKJF1      AACTGACACTGAGGGACGAAAGCTAGGGGAGCGAATGGGATTAGATACCCCAGTAGTCCTAGCCGTAAACGATGGATACTAGGCGTGGCTTGTATCGACC
SKS3       AACTGACACTGAGGGACGAAAGCTAGGGGAGCGAATGGGATTAGATACCCCAGTAGTCCTAGCCGTAAACGATGGATACTAGGCGTGGCTTGTATCGACC
SKJ6       AACTGACACTGAGGGACGAAAGCTAGGGGAGCGAATGGGATTAGATACCCCAGTAGTCCTAGCCGTAAACGATGGATACTAGGCGTGGCTTGTATCGACC
SKJ2       AACTGACACTGAGGGACGAAAGCTAGGGGAGCGAATGGGATTAGATACCCCAGTAGTCCTAGCCGTAAACGATGGATACTAGGCGTGGCTTGTATCGACC
SKS5       AACTGACACTGAGGGACGAAAGCTAGGGGAGCGAATGGGATTAGATACCCCAGTAGTCCTAGCCGTAAACGATGGATACTAGGCGTGGCTTGTATCGACC
SKS9       AACTGACACTGAGGGACGAAAGCTAGGGGAGCGAATGGGATTAGATACCCCAGTAGTCCTAGCCGTAAACGATGGATACTAGGCGTGGCTTGTATCGACC
KVJ18      AACTGACACTGAGGGACGAAAGCTAGGGGAGCGAATGGGATTAGATACCCCAGTAGTCCTAGCCGTAAACGATGGATACTAGGCGTGGCTTGTATCGACC
SKJ4       AACTGACACTGAGGGACGAAAGCTAGGGGAGCGAATGGGATTAGATACCCCAGTAGTCCTAGCCGTAAACGATGGATACTAGGCGTGGCTTGTATCGACC
SKSF1      AACTGACACTGAGGGACGAAAGCTAGGGGAGCGAATGGGATTAGATACCCCAGTAGTCCTAGCCGTAAACGATGGATACTAGGCGTGGCTTGTATCGACC
KVSF7      AACTGACACTGAGGGACGAAAGCTAGGGGAGCGAATGGGATTAGATACCCCAGTAGTCCTAGCCGTAAACGATGGATACTAGGCGTGGCTTGTATCGACC
SKS8       AACTGACACTGAGGGACGAAAGCTAGGGGAGCGAATGGGATTAGATACCCCAGTAGTCCTAGCCGTAAACGATGGATACTAGGCGTGGCTTGTATCGACC
DQ185249   AACTGACACTGAGGGACGAAAGCTAGGGGAGCGAATGGGATTAGATACCCCAGTAGTCCTAGCCGTAAACGATGGATACTAGGCGTGGCTTGTATCGACC
DQ185255   AACTGACACTGAGGGACGAAAGCTAGGGGAGCGAATGGGATTAGATACCCCAGTAGTCCTAGCCGTAAACGATGGATACTAGGCGTGGCTTGTATCGACC
AY566855   AACTGACACTGAGGGACGAAAGCTAGGGGAGCGAATGGGATTAGATACCCCAGTAGTCCTAGCCGTAAACGATGGATACTAGGCGTGGCTTGTATCGACC
AM711545   AACTGACACTGAGGGACGAAAGCTAGGGGAGCGAATGGGATTAGATACCCCAGTAGTCCTAGCCGTAAACGATGGATACTAGGCGTGGCTTGTATCGACC
AM711536   AACTGACACTGAGGGACGAAAGCTAGGGGAGCGAATGGGATTAGATACCCCAGTAGTCCTAGCCGTAAACGATGGATACTAGGCGTGGCTTGTATCGACC
DQ185256   AACTGACACTGAGGGACGAAAGCTAGGGGAGCGAATGGGATTAGATACCCCAGTAGTCCTAGCCGTAAACGATGGATACTAGGCGTGGCTTGTATCGACC
DQ185245   AACTGACACTGAGGGACGAAAGCTAGGGGAGCGAATGGGATTAGATACCCCAGTAGTCCTAGCCGTAAACGATGGATACTAGGCGTGGCTTGTATCGACC
```

```
AM711527     AACTGACACTGAGGGACGAAAGCTAGGGGAGCGAATGGGATTAGATACCCCAGTAGTCCTAGCCGTAAACGATGGATACTAGGCGTGGCTTGTATCGACC
DQ185212     AACTGACACTGAGGGACGAAAGCTAGGGGAGCGAATGGGATTAGATACCCCAGTAGTCCTAGCCGTAAACGATGGATACTAGGCGTGGCTTGTATCGACC
AF062638     AACTGACACTGAGGGACGAAAGCTAGGGGAGCGAATGGGATTAGATACCCCAGTAGTCCTAGCCGTAAACGATGGATACTAGGCGTGGCTTGTATCGACC
HM623781     AACTGACACTGAGGGACGAAAGCTAGGGGAGCGAATGGGATTAGATACCCCAGTAGTCCTAGCCGTAAACGATGGATACTAGGCGTGGCTTGTATCGACC
AM711540     AACTGACACTGAGGGACGAAAGCTAGGGGAGCGAATGGGATTAGATACCCCAGTAGTCCTAGCCGTAAACGATGGATACTAGGCGTGGCTTGTATCGACC
DQ185254     AACTGACACTGAGGGACGAAAGCTAGGGGAGCGAATGGGATTAGATACCCCAGTAGTCCTAGCCGTAAACGATGGATACTAGGCGTGGCTTGTATCGACC
DQ185251     AACTGACACTGAGGGACGAAAGCTAGGGGAGCGAATGGGATTAGATACCCCAGTAGTCCTAGCCGTAAACGATGGATACTAGGCGTGGCTTGTATCGACC
AM711535     AACTGACACTGAGGGACGAAAGCTAGGGGAGCGAATGGGATTAGATACCCCAGTAGTCCTAGCCGTAAACGATGGATACTAGGCGTGGCTTGTATCGACC
AF027653     AACTGACACTGAGGGACGAAAGCTAGGGGAGCGAATGGGATTAGATACCCCAGTAGTCCTAGCCGTAAACGATGGATACTAGGCGTGGCTTGTATCGACC
DQ185244     AACTGACACTGAGGGACGAAAGCTAGGGGAGCGAATGGGATTAGATACCCCAGTAGTCCTAGCCGTAAACGATGGATACTAGGCGTGGCTTGTATCGACC
AM711531     AACTGACACTGAGGGACGAAAGCTAGGGGAGCGAATGGGATTAGATACCCCAGTAGTCCTAGCCGTAAACGATGGATACTAGGCGTGGCTTGTATCGACC
AJ630449     AACTGACACTGAGGGACGAAAGCTAGGGGAGCGAATGGGATTAGATACCCCAGTAGTCCTAGCCGTAAACGATGGATACTAGGCGTGGCTTGTATCGACC
AY328897     AACTGACACTGAGGGACGAAAGCTAGGGGAGCGAATGGGATTAGATACCCCAGTAGTCCTAGCCGTAAACGATGGATACTAGGCGTGGCTTGTATCGACC
AM230702     TACTGACACTGAGGGACGAAAGCTAGGGGAGCGAATGGGATTAGATACCCCAGTAGTCCTAGCCGTAAACGATGGATACTAGGCGTGGYTTGTATCGACC
EF568907     AACTGACACTGAGGGACGAAAGCTAGGGGAGCGAATGGGATTAGATACCCCAGTAGTCCTAGCCGTAAACGATGGATACTAGGCGTGGCTTGTATCGACC
AJ630458     AACTGACACTGAGGGACGAAAGCTAGGGGAGCGAATGGGATTAGATACCCCAGTAGTCCTAGCCGTAAACGATGGATACTAGGCGTGGCTTGTATCGACC
GQ443447     AACTGACACTGAGGGACGAAAGCTAGGGGAGCGAATGGGATTAGATACCCCAGTAGTCCTAGCCGTAAACGATGGATACTAGGCGTGGCTTGTATCGACC
AJ630457     AACTGACACTGAGGGACGAAAGCTAGGGGAGCGAATGGGATTAGATACCCCAGTAGTCCTAGCCGTAAACGATGGATACTAGGCGTGGCTTGTATCGACC
DQ234831     AACTGACACTGAGGGACGAAAGCTAGGGGAGCGAATGGGATTAGATACCCCAGTAGTCCTAGCCGTAAACGATGGATACTAGGCGTGGCTTGTATCGACC
AJ133161     AACTGACACTGAGGGACGAAAGCTAGGGGAGCGAATGGGATTAGATACCCCAGTAGTCCTAGCCGTAAACGATGGATACTAGGCGTGGCTTGTATCGACC
gi|47118302  AACTGACACTGAGGGACGAAAGCTAGGGGAGCGAATGGGATTAGATACCCCAGTAGTCCTAGCCGTAAACGATGGATACTAGGCGTGGCTTGTATCGACC
DQ185209     AACTGACACTGAGGGACGAAAGCTAGGGGAGCGAATGGGATTAGATACCCCAGTAGTCCTAGCCGTAAACGATGGATACTAGGCGTGGCTTGTATCGACC
AY742451     AACTGACACTGAGGGACGAAAGCTAGGGGAGCGAATGGGATTAGATACCCCAGTAGTCCTAGCCGTAAACGATGGATACTAGGCGTGGCTTGTATCGACC
AF027655     AACTGACACTGAGGGACGAAAGCTAGGGGAGCGAATGGGATTAGATACCCCAGTAGTCCTAGCCGTAAACGATGGATACTAGGCGTGGCTTGTATCGACC
DQ185211     AACTGACACTGAGGGACGAAAGCTAGGGGAGCGAATGGGATTAGATACCCCAGTAGTCCTAGCCGTAAACGATGGATACTAGGCGTGGCTTGTATCGACC
AY742449     AACTGACACTGAGGGACGAAAGCTAGGGGAGCGAATGGGATTAGATACCCCAGTAGTCCTAGCCGTAAACGATGGATACTAGGCGTGGCTTGTATCGACC
EF174212     AACTGACACTGAGGGACGAAAGCTAGGGGAGCGAATGGGATTAGATACCCCAGTAGTCCTAGCCGTAAACGATGGATACTAGGCGTGGCTTGTATCGACC
DQ185213     AACTGACACTGAGGGACGAAAGCTAGGGGAGCGAATGGGATTAGATACCCCAGTAGTCCTAGCCGTAAACGATGGATACTAGGCGTGGCTTGTATCGACC
AF506237     AACTGACACTGAGGGACGAAAGCTAGGGGAGCGAATGGGATTAGATACCCCAGTAGTCCTAGCCGTAAACGATGGATACTAGGCGTTGCTTGTATCGACC
AM230690     AACTGACACTGAGGGACGAAAGCTAGGGGAGCGAATGGGATTAGATACCCCAGTAGTCCTAGCCGTAAACGATGGATACTAGGCGTTGTCTGTATCGACC
AB039002     AACTGACACTGAGGGACGAAAGCTAGGGGAGCGAATGGGATTAGATACCCCAGTAGTCCTAGCCGTAAACGATGGATACTAGGCGTGGCTTGTATCGACC
AJ293131     GACTGACACTGAGGGACGAAAGCTAGGGGAGCGAATGGGATTAGATACCCCAGTAGTCCTAGCCGTAAACGATGGATACTAGGCGTAGCTCGTATCGACC
AJ133169     AACTGACACTGAGGGACGAAAGCTAGGGGAGCGAATGGGATTAGATACCCCAGTAGTCCTAGCGGTAAACGATGGAAACTAGGTGTGGCCTGTATCGACC
DQ279769     AACTGACACTGAGGGACGAAAGCTAGGGGAGCGAATGGGATTAGATACCCCAGTAGTCCTAGCCGTAAACGATGGAAACTAGGTGTAGCCTGTATCGACC
AJ000714     AACTGACACTGAGGGACGAAAGCTAGGGGAGCGAATGGGATTAGATACCCCAGTAGTCCTAGCTGTAAACGATGGAAACTAGGTGTTGCCTGTATCGACC
KVSF5        CACTGACACTGAGGGACGAAAGCTAGGGGAGCGAAAGGGATTAGATACCCCAGTAGTCCTAGCCGTAAACGATGGATACTAGGCGTTGTCTGTATCGACC

                     810       820       830       840       850       860       870       880       890       900
             ....|....|....|....|....|....|....|....|....|....|....|....|....|....|....|....|....|....|....|....|
KVJ10        CG-AGCCGTGCCGTAGCTAACGCGTTAAGTATCCCGCCTGGGGAGTACGCCGGCAACGGTGAAACTCAAAGGAATTGACGGGGGCCCGCACAAGCGGTGG
KVJF8        CG-AGCCGTGCCGTAGCTAACGCGTTAAGTATCCCGCCTGGGGAGTACGCCGGCAACGGTGAAACTCAAAGGAATTGACGGGGGCCCGCACAAGCGGTGG
KVJ3         CG-AGCCGTGCCGTAGCTAACGCGTTAAGTATCCCGCCTGGGGAGTACGCCGGCAACGGTGAAACTCAAAGGAATTGACGGGGGCCCGCACAAGCGGTGG
SKS1         CG-AGCCGTGCCGTAGCTAACGCGTTAAGTATCCCGCCTGGGGAGTACGCCGGCAACGGTGAAACTCAAAGGAATTGACGGGGGCCCGCACAAGCGGTGG
KVS1         CG-AGCCGTGCCGTAGCTAACGCGTTAAGTATCCCGCCTGGGGAGTACGCCGGCAACGGTGAAACTCAAAGGAATTGACGGGGGCCCGCACAAGCGGTGG
SKJ1         CG-AGCCGTGCCGTAGCTAACGCGTTAAGTATCCCGCCTGGGGAGTACGCCGGCAACGGTGAAACTCAAAGGAATTGACGGGGGCCCGCACAAGCGGTGG
KVJF4        CG-AGCCGTGCCGTAGCTAACGCGTTAAGTATCCCGCCTGGGGAGTACGCCGGCAACGGTGAAACTCAAAGGAATTGAGGGGGGCCCGCACAAGCGGTGG
```

KVS11	CG-AGCCGTGCCGTAGCTAACGCGTTAAGTATCCCGCCTGGGGAGTACGCCGGCAACGGTGAAACTCAAAGGAATTGACGGGGGCCCGCACAAGCGGTGG
KVJ20	CG-AGCCGTGCCGTAGCTAACGCGTTAAGTATCCCGCCTGGGGAGTACGCCGGCAACGGTGAAACTCAAAGGAATTGACGGGGGCCCGCACAAGCGGTGG
SKSF3	CG-AGCCGTGCCGTAGCTAACGCGTTAAGTATCCCGCCTGGGGAGTACGCCGGCAACGGTGAAACTCAAAGGAATTGACGGGGGCCCGCACAAGCGGTGG
SKSL1	CG-AGCCGTGCCGTAGCTAACGCGTTAAGTATCCCGCCTGGGGAGTACGCCGGCAACGGTGAAACTCAAAGGAATTGACGGGGGCCCGCACAAGCGGTGG
SKSL2	CG-AGCCGTGCCGTAGCTAACGCGTTAAGTATCCCGCCTGGGGAGTACGCCGGCAACGGTGAAACTCAAAGGAATTGACGGGGGCCCGCACAAGCGGTGG
KVJ4	CG-AGCCGTGCCGTAGCTAACGCGTTAAGTATCCCGCCTGGGGAGTACGCCGGCAACGGTGAAACTCAAAGGAATTGACGGGGGCCCGCACAAGCGGTGG
KVJ2	CG-AGCCGTGCCGTAGCTAACGCGTTAAGTATCCCGCCTGGGGAGTACGCCGGCAACGGTGAAACTCAAAGGAATTGACGGGGGCCCGCACAAGCGGTGG
KVSF4	CG-AGCCGTGCCGTAGCTAACGCGTTAAGTATCCCGCCTGGGGAGTACGCCGGCAACGGTGAAACTCAAAGGAATTGACGGGGGCCCGCACAAGCGGTGG
SKS2	CG-AGCCGTGCCGTAGCTAACGCGTTAAGTATCCCGCCTGGGGAGTACGCCGGCAACGGTGAAACTCAAAGGAATTGACGGGGGCCCGCACAAGCGGTGG
SKJF1	CG-AGCCGTGCCGTAGCTAACGCGTTAAGTATCCCGCCTGGGGAGTACGCCGGCAACGGTGAAACTCAAAGGAATTGACGGGGGCCCGCACAAGCGGTGG
SKS3	CG-AGCCGTGCCGTAGCTAACGCGTTAAGTATCCCGCCTGGGGAGTACGCCGGCAACGGTGAAACTCAAAGGAATTGACGGGGGCCCGCACAAGCGGTGG
SKJ6	CG-AGCCGTGCCGTAGCTAACGCGTTAAGTATCCCGCCTGGGGAGTACGCCGGCAACGGTGAAACTCAAAGGAATTGACGGGGGCCCGCACAAGCGGTGG
SKJ2	CG-AGCCGTGCCGTAGCTAACGCGTTAAGTATCCCGCCTGGGGAGTACGCCGGCAACGGTGAAACTCAAAGGAATTGACGGGGGCCCGCACAAGCGGTGG
SKS5	CG-AGCCGTGCCGTAGCTAACGCGTTAAGTATCCCGCCTGGGGAGTACGCCGGCAACGGTGAAACTCAAAGGAATTGACGGGGGCCCGCACAAGCGGTGG
SKS9	CG-AGCCGTGCCGTAGCTAACGCGTTAAGTATCCCGCCTGGGGAGTACGCCGGCAACGGTGAAACTCAAAGGAATTGACGGGGGCCCGCACAAGCGGTGG
KVJ18	CG-AGCCGTGCCGTAGCTAACGCGTTAAGTATCCCGCCTGGGGAGTACGCCGGCAACGGTGAAACTCAAAGGAATTGACGGGGGCCCGCACAAGCGGTGG
SKJ4	CG-AGCCGTGCCGTAGCTAACGCGTTAAGTATCCCGCCTGGGGAGTACGCCGGCAACGGTGAAACTCAAAGGAATTGACGGGGGCCCGCACAAGCGGTGG
SKSF1	CG-AGCCGTGCCGTAGCTAACGCGTTAAGTATCCCGCCTGGGGAGTACGCACGCAAGTGTGAAACTCAAAGGAATTGACGGGGGCCCGCACAAGCGGTGG
KVSF7	CG-AGCCGTGCCGTAGCTAACGCGTTAAGTATCCCGCCTGGGGAGTACGCACGCAAGTGTGAAACTCAAAGGAATTGACGGGGGCCCGCACAAGCGGTGG
SKS8	CG-AGCCGTGCCGTAGCTAACGCGTTAAGTATCCCGCCTGGGGAGTACGCAGGCAACTGTGAAACTCAAAGGAATTGACGGGGGCCCGCACAAGCGGTGG
DQ185249	CG-AGCCGTGCCGTAGCTAACGCGTTAAGTATCCCGCCTGGGGAGTACGCCGGCAACGGTGAAACTCAAAGGAATTGACGGGGGCCCGCACAAGCGGTGG
DQ185255	CG-AGCCGTGCCGTAGCTAACGCGTTAAGTATCCCGCCTGGGGAGTACGCCGGCAACGGTGAAACTCAAAGGAATTGACGGGGGCCCGCACAAGCGGTGG
AY566855	CG-AGCCGTGCCGTAGCTAACGCGTTAAGTATCCCGCCTGGGGAGTACGCCGGCAACGGTGAAACTCAAAGGAATTGACGGGGGCCCGCACAAGCGGTGG
AM711545	CG-AGCCGTGCCGTAGCTAACGCGTTAAGTATCCCGCCTGGGGAGTACGCCGGCAACGGTGAAACTCAAAGGAATTGACGGGGGCCCGCACAAGCGGTGG
AM711536	CG-AGCCGTGCCGTAGCTAACGCGTTAAGTATCCCGCCTGGGGAGTACGCCGGCAACGGTGAAACTCAAAGGAATTGACGGGGGCCCGCACAAGCGGTGG
DQ185256	CG-AGCCGTGCCGTAGCTAACGCGTTAAGTATCCCGCCTGGGGAGTACGCCGGCAACGGTGAAACTCAAAGGAATTGACGGGGGCCCGCACAAGCGGTGG
DQ185245	CG-AGCCGTGCCGTAGCTAACGCGTTAAGTATCCCGCCTGGGGAGTACGCCGGCAACGGTGAAACTCAAAGGAATTGACGGGGGCCCGCACAAGCGGTGG
AM711527	CG-AGCCGTGCCGTAGCTAACGCGTTAAGTATCCCGCCTGGGGAGTACGCCGGCAACGGTGAAACTCAAAGGAATTGACGGGGGCCCGCACAAGCGGTGG
DQ185212	CG-AGCCGTGCCGTAGCTAACGCGTTAAGTATCCCGCCTGGGGAGTACGCCGGCAACGGTGAAACTCAAAGGAATTGACGGGGGCCCGCACAAGCGGTGG
AF062638	CG-AGCCGTGCCGTAGCTAACGCGTTAAGTATCCCGCCTGGGGAGTACGCCGGCCACGGTGAAACTCAAAGGAATTGACGGGGGCCCGCACAAGCGGTGG
HM623781	CG-AGCCGTGCCGTAGCTAACGCGTTAAGTATCCCGCCTGGGGAGTACGCCGGCAACGGTGAAACTCAAAGGAATTGACGGGGGCCCGCACAAGCGGTGG
AM711540	CG-AGCCGTGCCGTAGCTAACGCGTTAAGTATCCCGCCTGGGGAGTACGCCGGCAACGGTGAAACTCAAAGGAATTGACGGGGGCCCGCACAAGCGGTGG
DQ185254	CG-AGCCGTGCCGTAGCTAACGCGTTAAGTATCCCGCCTGGGGAGTACGCCGGCAACGGTGAAACTCAAAGGAATTGACGGGGGCCCGCACAAGCGGTGG
DQ185251	CG-AGCCGTGCCGTAGCTAACGCGTTAAGTATCCCGCCTGGGGAGTACGCCGGCAACGGTGAAACTCAAAGGAATTGACGGGGGCCCGCACAAGCGGTGG
AM711535	CG-AGCCGTGCCGTAGCTAACGCGTTAAGTATCCCGCCTGGGGAGTACGCCGGCAACGGTGAAACTCAAAGGAATTGACGGGGGCCCGCACAAGCGGTGG
AF027653	CG-AGCCGTGCCGTAGCTAACGCGTTAAGTATCCCGCCTGGGGAGTACGCCGGCAACGGTGAAACTCAAAGGAATTGACGGGGGCCCGCACAAGCGGTGG
DQ185244	CG-AGCCGTGCCGTAGCTAACGCGTTAAGTATCCCGCCTGGGGAGTACGCCGGCAACGGTGAAACTCAAAGGAATTGACGGGGGCCCGCACAAGCGGTGG
AM711531	CG-AGCCGTGCCGTAGCTAACGCGTTAAGTATCCCGCCTGGGGAGTACGCCGGCAACGGTGAAACTCAAAGGAATTGACGGGGGCCCGCACAAGCGGTGG
AJ630449	CG-AGCCGTGCCGTAGCTAACGCGTTAAGTATCCCGCCTGGGGAGTACGCCGGCAACGGTGAAACTCAAAGGAATTGACGGGGGCCCGCACAAGCGGTGG
AY328897	CG-ATCCGTGCCGTAGCTAACGCGTTAAGTATCCCGCCTGGGGAGTACGCCGGCAACGGTGAAACTCAAAGGAATTGACGGGGGCCCGCACAAGCGGTGG
AM230702	CG-CGCCGTGCCGTAGCTAACGCGTTAAGTATCCCGCCTGGGGAGTACGCACGCAAGTGTGAAACTCAAAGGAATTGACGGGGGCCCGCACAAGCGGTGG
EF568907	CG-AGCCGTGCCGTAGCTAACGCGTTAAGTATCCCGCCTGGGGAGTACGCACGCAAGTGTGAAACTCAAAGGAATTGACGGGGGCCCGCACAAGCGGTGG
AJ630458	CG-AGCCGTGCCGTAGCTAACGCGTTAAGTATCCCGCCTGGGGAGTACGCACGCAAGTGTGAAACTCAAAGGAATTGACGGGGGCCCGCACAAGCGGTGG
GQ443447	CG-AGCCGTGCCGTAGCTAACGCGTTAAGTATCCCGCCTGGGGAGTACGCACGCAAGTGTGAAACTCAAAGGAATTGACGGGGGCCCGCACAAGCGGTGG
AJ630457	CG-AGCCGTGCCGTAGCTAACGCGTTAAGTATCCCGCCTGGGGAGTACGCACGCAAGTGTGAAACTCAAAGGAATTGACGGGGGCCCGCACAAGCGGTGG
DQ234831	CG-AGCCGTGCCGTAGCTAACGCGTTAAGTATCCCGCCTGGGGAGTACGCACGCAAGTGTGAAACTCAAAGGAATTGACGGGGGCCCGCACAAGCGGTGG

```
AJ133161     CG-AGCCGTGCCGTAGCTAACGCGTTAAGTATCCCGCCTGGGGAGTACGCAGGCAACTGTGAAACTCAAAGGAATTGACGGGGGCCCGCACAAGCGGTGG
gi|47118302  CG-AGCCGTGCCGGAGCCAACGCGTTAAGTATCCCGCCTGGGGAGTACGCACGCAAGTGTGAAACTCAAAGGAATTGACGGGGGCCCGCACAAGCGGTGG
DQ185209     CG-AGCCGTGCCGTAGCTAACGCGTTAAGTATCCCGCCTGGGGAGTACGCCGGCAACGGTGAAACTCAAAGGAATTGACGGGGGCCCGCACAAGCGGTGG
AY742451     CG-AGCCGTGCCGTAGCTAACGCGTTAAGTATCCCGCCTGGGGAGTACGCCGGCAACGGTGAAACTCAAAGGAATTGACGGGGGCCCGCACAAGCGGTGG
AF027655     CG-AGCCGTGCCGTAGCTAACGCGTTAAGTATCCCGCCTGGGGAGTACGCCGGCAACGGTGAAACTCAAAGGAATTGACGGGGGCCCGCACAAGCGGTGG
DQ185211     CG-AGCCGTGCCGTAGCTAACGCGTTAAGTATCCCGCCTGGGGAGTACGCCGGCAACGGTGAAACTCAAAGGAATTGACGGGGGCCCGCACAAGCGGTGG
AY742449     CG-AGCCGTGCCGTAGCTAACGCGTTAAGTATCCCGCCTGGGGAGTACGCCGGCAACGGTGAAACTCAAAGGAATTGACGGGGGCCCGCACAAGCGGTGG
EF174212     CG-AGCCGTGCCGTAGCTAACGCGTTAAGTATCCCGCCTGGGGAGTACGCCGGCAACGGTGAAACTCAAAGGAATTGACGGGGGCCCGCACAAGCGGTGG
DQ185213     CG-AGCCGTGCCGTAGCTAACGCGTTAAGTATCCCGCCTGGGGAGTACGCCGGCAACGGTGAAACTCAAAGGAATTGACGGGGGCCCGCACAAGCGGTGG
AF506237     CGAGACAGTGCCGTAGCTAACGCGTTAAGTATCCCGCCTGGGGAGTACGCACGCAAGTGTGAAACTCAAAGGAATTGACGGGGGCCCGCACAAGCGGTGG
AM230690     CG-GACAGTGCCGTAGCTAACGCGTTAAGTATCCCGCCTGGGGAGTACGCACGCAAGTGTGAAACTCAAAGGAATTGACGGGGGCCCGCACAAGCGGTGG
AB039002     CA-AGCCGTGCCGTAGCTAACGCGTTAAGTATCCCGCCTGGGGAGTACGCACGCAAGTGTGAAACTCAAAGGAATTGACGGGGGCC-GCACAAGCGGTGG
AJ293131     CG-AGCTGTGCCGTAGCTAACGCGTTAAGTATCCCGCCTGGGGAGTACGCAGGCAACTGTGAAACTCAAAGGAATTGACGGGGGCCCGCACAAGCGGTGG
AJ133169     CG-GGCCGTGCCGAAGCAAACGCGTTAAGTTTCCCGCCTGGGGAGTACGCACGCAAGTGTGAAACTCAAAGGAATTGACGGGGGCCCGCACAAGCGGTGG
DQ279769     CG-GGCTGTGCCGAAGCTAACGCGTTAAGTTTCCCGCCTGGGGAGTACGCACGCAAGTGTGAAACTCAAAGGAATTGACGGGGGCCCGCACAAGCGGTGG
AJ000714     CA-GGCAGTGCCGTAGCTAACGCGTTAAGTTTCCCGCCTGGGGAGTACGCACGCAAGTGTGAAACTCAAAGGAATTGACGGGGGCCCGCACAAGCGGTGG
KVSF5        CG-GACAGTGCCGTAGCTAACGCGTTAAGTATCCCGCCTGGGGAGTACGCAGGCAAGTGTGAAACTCAAAGGAATTGACGGGGGCCCGCACAAGCGGTGG

                     910       920       930       940       950       960       970       980       990      1000
             ....|....|....|....|....|....|....|....|....|....|....|....|....|....|....|....|....|....|....|....|
KVJ10        AGTATGTGGTTTAATTCGATGCAACGCGAAGAACCTTACCAAGGCTTGACATGTCGCGAATCTTCTCGAAAGGGAAGAGTGCCTTCGGGAGCGCGAACAC
KVJF8        AGTATGTGGTTTAATTCGATGCAACGCGAAGAACCTTACCAAGGCTTGACATGTCGCGAATCTTCTCGAAAGGGAAGAGTGCCTTCGGGAGCGCGAACAC
KVJ3         AGTATGTGGTTTAATTCGATGCAACGCGAAGAACCTTACCAAGGCTTGACATGTCGCGAATCTTCTTGAAAGGGAAGAGTGCCTTCGGGAGCGCGAACAC
SKS1         AGTATGTGGTTTAATTCGATGCAACGCGAAGAACCTTACCAAGGCTTGACATGTCGCGAATCTTCTTGAAAGGGAAGAGTGCCTTCGGGAGCGCGAACAC
KVS1         AGTATGTGGTTTAATTCGATGCAACGCGAAGAACCTTACCAAGGCTTGACATGTCGCGAATCTTCTTGAAAGGGAAGAGTGCCTTCGGGAGCGCGAACAC
SKJ1         AGTATGTGGTTTAATTCGATGCAACGCGAAGAACCTTACCAAGGCTTGACATGTCGCGAATCTTCTTGAAAGGGAAGAGTGCCTTCGGGAGCGCGAACAC
KVJF4        AGTATGTGGTTTAATTCGATGCAACGCGAAGAACCTTACCAAGGCTTGACATGTCGCGAATCTTCTTGAAAGGGAAGAGTGCCTTCGGGAGCGCGAACAC
KVS11        AGTATGTGGTTTAATTCGATGCAACGCGAAGAACCTTACCAAGGCTTGACATGTCGCGAATCTTCTTGAAAGGGAAGAGTGCCTTCGGGAGCGCGAACAC
KVJ20        AGTATGTGGTTTAATTCGATGCAACGCGAAGAACCTTACCAAGGCTTGACATGTCGCGAATCCGGGTGAAAGCCGGGAGTGCCTTCGGGAGCGCGAACAC
SKSF3        AGTATGTGGTTTAATTCGATGCAACGCGAAGAACCTTACCAAGGCTTGACATGTCGCGAATCCGGGTGAAAGCCGGGAGTGCCTTCGGGAGCGCGAACAC
SKSL1        AGTATGTGGTTTAATTCGATGCAACGCGAAGAACCTTACCAAGGCTTGACATGTCGCGAATCCTGGTGAAAGCTGGGAGTGCCTTCGGGAGCGCGAACAC
SKSL2        AGTATGTGGTTTAATTCGATGCAACGCGAAGAACCTTACCAAGGCTTGACATGTCGCGAATCCTGGTGAAAGCTGGGAGTGCCTTCGGGAGCGCGAACAC
KVJ4         AGTATGTGGTTTAATTCGATGCAACGCGAAGAACCTTACCAAGGCTTGACATGTCGCGAATCTTCTTGAAAGGGAAGAGTGCCTTCGGGAGCGCGAACAC
KVJ2         AGTATGTGGTTTAATTCGATGCAACGCGAAGAACCTTACCAAGGCTTGACATGTCGCGAATCTTCTTGAAAGGGAAGAGTGCCTTCGGGAGCGCGAACAC
KVSF4        AGTATGTGGTTTAATTCGATGCAACGCGAAGAACCTTACCAAGGCTTGACATGTCGCGAATCTTCTTGAAAGGGAAGAGTGCCTTCGGGAGCGCGAACAC
SKS2         AGTATGTGGTTTAATTCGATGCAACGCGAAGAACCTTACCAAGGCTTGACATGTCGCGAATCTTCTTGAAAGGGAAGAGTGCCTTCGGGAGCGCGAACAC
SKJF1        AGTATGTGGTTTAATTCGATGCAACGCGAAGAACCTTACCAAGGCTTGACATGTCGCGAATCTTGATGAAAGTTGAGAGTGCCTTCGGGAGCGCGAACAC
SKS3         AGTATGTGGTTTAATTCGATGCAACGCGAAGAACCTTACCAAGGCTTGACATGTCGCGAATCTTGATGAAAGTTGAGAGTGCCTTCGGGAGCGCGAACAC
SKJ6         AGTATGTGGTTTAATTCGATGCAACGCGAAGAACCTTACCAAGGCTTGACATGTCGCGAATCTTGATGAAAGTTGAGAGTGCCTTCGGGAGCGCGAACAC
SKJ2         AGTATGTGGTTTAATTCGATGCAACGCGAAGAACCTTACCAAGGCTTGACATGTCGCGAATCTTTCTGAAAGGAGAGAGTGCCTTCGGGAGCGCGAACAC
SKS5         AGTATGTGGTTTAATTCGATGCAACGCGAAGAACCTTACCAAGGCTTGACATGTCGCGAATCTTCTTGAAAGGGAAGAGTGCCTTCGGGAGCGCGAACAC
SKS9         AGTATGTGGTTTAATTCGATGCAACGCGAAGAACCTTACCAAGGCTTGACATGTCGCGAATCTTCTTGAAAGGGAAGAGTGCCTTCGGGAGCGCGAACAC
KVJ18        AGTATGTGGTTTAATTCGATGCAACGCGAAGAACCTTACCAAGGCTTGACATGTCGCGAATCTCGGTGAAAGCTGAGAGTGCCTTCGGGAGCGCGAACAC
SKJ4         AGTATGTGGTTTAATTCGATGCAACGCGAAGAACCTTACCAAGGCTTGACATGTCGCGAATCCTGGTGAAAGCTGGGAGTGCCTTCGGGAGCGCGAACAC
SKSF1        AGTATGTGGTTTAATTCGATGCAACGCGAAGAACCTTACCAAGACTTGACATGTCGCGAATCTCTTTGAAAGGAGAGAGTGCCTTCGGGAGCGCGAACAC
KVSF7        AGTATGTGGTTTAATTCGATGCAACGCGAAGAACCTTACCAAGACTTGACATGTCGCGAATCTTCTTGAAAGGGGAGAGTGCCTTCGGGAGCGCGAACAC
```

SKS8	AGTATGTGGTTTAATTCGATGCAACGCGAAGAACCTTACCAAGACTTGACATGTCGCGAATCTTCTTGAAAGGGAAGAGTGCCTTCGGGAGCGCGAACAC
DQ185249	AGTATGTGGTTTAATTCGATGCAACGCGAAGAACCTTACCAAGGCTTGACATGTCGCGAATCTTCTCGAAAGGGAAGAGTGCCTTCGGGAGCGCGAACAC
DQ185255	AGTATGTGGTTTAATTCGATGCAACGCGAAGAACCTTACCAAGGCTTGACATGTCGCGAATCTTCTCGAAAGGGAAGAGTGCCTTCGGGAGCGCGAACAC
AY566855	AGTATGTGGTTTAATTCGATGCAACGCGAAGAACCTTACCAAGGCTTGACATGTCGCGAATCTTCTTGAAAGGGAAGAGTGCCTTCGGGAGCGCGAACAC
AM711545	AGTATGTGGTTTAATTCGATGCAACGCGAAGAACCTTACCAAGGCTTGACATGTCGCGAATCTTCTTGAAAGGGAAGAGTGCCTTCGGGAGCGCGAACAC
AM711536	AGTATGTGGTTTAATTCGATGCAACGCGAAGAACCTTACCAAGGCTTGACATGTCGCGAATCTTCTTGAAAGGGAAGAGTGCCTTCGGGAGCGCGAACAC
DQ185256	AGTATGTGGTTTAATTCGATGCAACGCGAAGAACCTTACCAAGGCTTGACATGTCGCGAATCTTCTTGAAAGGGAAGAGTGCCTTCGGGAGCGCGAACAC
DQ185245	AGTATGTGGTTTAATTCGATGCAACGCGAAGAACCTTACCAAGGCTTGACATGTCGCGAATCTTCTTGAAAGGGAAGAGTGCCTTCGGGAGCGCGAACAC
AM711527	AGTATGTGGTTTAATTCGATGCAACGCGAAGAACCTTACCAAGGCTTGACATGTCGCGAATCTTCTTGAAAGGGAAGAGTGCCTTCGGGAGCGCGAACAC
DQ185212	AGTATGTGGTTTAATTCGATGCAACGCGAAGAACCTTACCAAGGCTTGACATGTCGCGAATCTTCTTGAAAGGGAAGAGTGCCTTCGGGAGCGCGAACAC
AF062638	AGTATGTGGTTTAATTCGATGCAACGCGAAGAACCTTACCAAGGCTTGACATGTCGCGAATCTTCTTGAAAGGGAAGAGTGCCTTCGGGAGCGCGAACAC
HM623781	AGTATGTGGTTTAATTCGATGCAACGCGAAGAACCTTACCAAGGCTTGACATGTCGCGAATCCTGGTGAAAGCTGGGAGTGCCTTCGGGAGCGCGAACAC
AM711540	AGTATGTGGTTTAATTCGATGCAACGCGAAGAACCTTACCAAGGCTTGACATGTCGCGAATCCTGGTGAAAGCTGGGAGTGCCTTCGGGAGCGCGAACAC
DQ185254	AGTATGTGGTTTAATTCGATGCAACGCGAAGAACCTTACCAAGGCTTGACATGTCGCGAATCCTGGTGAAAGCCGGGAGTGCCTTCGGGAGCGCGAACAC
DQ185251	AGTATGTGGTTTAATTCGATGCAACGCGAAGAACCTTACCAAGGCTTGACATGTCGCGAATCCTGGTGAAAGCTGGGAGTGCCTTCGGGAGCGCGAACAC
AM711535	AGTATGTGGTTTAATTCGATGCAACGCGAAGAACCTTACCAAGGCTTGACATGTCGCGAATCCTGGTGAAAGCTGGGAGTGCCTTCGGGAGCGCGAACAC
AF027653	AGTATGTGGTTTAATTCGATGCAACGCGAAGAACCTTACCAAGGCTTGACATGTCGCGAATCCTGGTGAAAGCTGGGAGTGCCTTCGGGAGCGCGAACAC
DQ185244	AGTATGTGGTTTAATTCGATGCAACGCGAAGAACCTTACCAAGGCTTGACATGTCGCGAATCCTGGTGAAAGCTGGGAGTGCCTTCGGGAGCGCGAACAC
AM711531	AGTATGTGGTTTAATTCGATGCAACGCGAAGAACCTTACCAAGGCTTGACATGTCGCGAATCCTGGTGAAAGCTGGGAGTGCCTTCGGGAGCGCGAACAC
AJ630449	AGTATGTGGTTTAATTCGATGCAACGCGAAGAACCTTACCAAGGCTTGACATGTCGCGAATCTTCTTGAAAGGGAAGAGTGCCTTCGGGAGCGCGAACAC
AY328897	AGTATGTGGTTTAATTCGATGCAACGCGAAGAACCTTACCAAGGCTTGACATGTCGCGAATCTTGATGAAAGTTGAGAGTGCCTTCGGGAGCGCGAACAC
AM230702	AGTATGTGGTTTAATTCGATGCAACGCGAAGAACCTTACCAAGGCTTGACATGTCGCGAATCTTTCTGAAAGGTGARRRTGCCTTSGGGAGCTGGAACAC
EF568907	AGTATGTGGTTTAATTCGATGCAACGCGAAGAACCTTACCAAGGCTTGACATGTCGCGAATCTTTCTGAAAGGAGAGAGTGCCTTCGGGAGCGCGAACAC
AJ630458	AGTATGTGGTTTAATTCGATGCAACGCGAAGAACCTTACCAAGACTTGACATGTCGCGAATCTTTCTGAAAGGAGAGAGTGCCTTCGGGAGCGCGAACAC
GQ443447	AGTATGTGGTTTAATTCGATGCAACGCGAAGAACCTTACCAAGGCTTGACATGTCGCGAATCTTTCTGAAAGGAGAGAGTGCCTTCGGGAGCGCGAACAC
AJ630457	AGTATGTGGTTTAATTCGATGCAACGCGAAGAACCTTACCAAGACTTGACATGTCGCGAATCTTCTTGAAAGGGGAGAGTGCCTTCGGGAGCGCGAACAC
DQ234831	AGTATGTGGTTTAATTCGATGCAACGCGAAGAACCTTACCAAGACTTGACATGTCGCGAATCTTCTTGAAAGGGGAGAGTGCCTTCGGGAGCGCGAACAC
AJ133161	AGTATGTGGTTTAATTCGATGCAACGCGAAGAACCTTACCAAGACTTGACATGTCGCGAATCCCTGTGAAAGTAGGGAGTGCCTTCGGGAGCGCGAACAC
gi|47118302	AGTATGTGGTTTAATTCGATGCAACGCGAAGAACCTTACCAAGACTTGACATGTCGCGAATCTTCTTGAAAGGGAAGAGTGCCTTAGGGAGCGCGAACAC
DQ185209	AGTATGTGGTTTAATTCGATGCAACGCGAAGAACCTTACCAAGGCTTGACATGTCGCGAATCTTCTCGAAAGGGAAGAGTGCCTTCGGGAGCGCGAACAC
AY742451	AGTATGTGGTTTAATTCGATGCAACGCGAAGAACCTTACCAAGGCTTGACATGTCGCGAATCCTGGTGAAAGCTGGGAGTGCCTTCGGGAGCGCGAACAC
AF027655	AGTATGTGGTTTAATTCGATGCAACGCGAAGAACCTTACCAAGGCTTGACATGTCGCGAATCCTGGTGAAAGCCGGGAGTGCCTTCGGGAGCGCGAACAC
DQ185211	AGTATGTGGTTTAATTCGATGCAACGCGAAGAACCTTACCAAGGCTTGACATGTCGCGAATCTTGATGAAAGTTGAGAGTGCCTTCGGGAGCGCGAACAC
AY742449	AGTATGTGGTTTAATTCGATGCAACGCGAAGAACCTTACCAAGGCTTGACATGTCGCGAATCCTGGTGAAAGCTGGGAGTGCCTTCGGGAGCGCGAACAC
EF174212	AGTATGTGGTTTAATTCGATGCAACGCGAAGAACCTTACCAAGGCTTGACATGTCGCGAATCCTGCTGAAAGGCGGGAGTGCCTTCGGGAGCGCGAACAC
DQ185213	AGTATGTGGTTTAATTCGATGCAACGCGAAGAACCTTACCAAGGCTTGACATGTCGCGAATCTTCTTGAAAGGGAAGAGTGCCTTCGGGAGCGCGAACAC
AF506237	AGTATGTGGTTTAATTCGATGCAACGCGAAGAACCTTACCAGGGCTTGACATGTCCAGAATTCYTTGGAAACGGGGAAGTGCCTTCGGGAGCTGGAACAC
AM230690	AGTATGTGGTTTAATTCGATGCAACGCGAAGAACCTTACCAGGGCTTGACATGTCCAGAATCTTGCTGAAAGGTGAGAGTGCCTTAGGGAGCTGGAACAC
AB039002	AGTATGTGGTTTAATTCGATGCAACGCGAAGAACCTTACCAAGACTTGACATGTCGCGAATCTTCTTGAAAGGGAAGAGTGCCTTCGGGAGCGCGAACAC
AJ293131	AGTATGTGGTTTAATTCGATGCAACGCGAAGAACCTTACCAAGGCTTGACATGTCACGAGTTCCGTTGAAAGATGGAAGTGCCTTCGGGAGCGTGAACAC
AJ133169	AGTATGTGGTTTAATTCGATGCAACGCGAAGAACCTTACCAGGACTTGACATCTCTGGAATCTCCTTGAAAGGGGAGAGTGCCGTAAGGAACCAGAAGAC
DQ279769	AGTATGTGGTTTAATTCGATGCAACGCGAAGAACCTTACCAGGGCTTGACATGTCCGGAATCTTGGTGAAAGCTGAGAGTGCCTTCGGGAGCCGGAACAC
AJ000714	AGTATGTGGTTTAATTCGATGCAACGCGAAGAACCTTACCAGGGCTTGACATGTCCGGAATTCTGGGGAAACTCGGAAGTGCCTTCGGGAACCGGAACAC
KVSF5	AGTATGTGGTTTAATTCGATGCAACGCGAAGAAGCTTACCAGGGCTTGACGTGTCCAGAATCTTGATGAAAGTTGAGAGTGCCTTCGGGAGCTGGAACAC

1010 1020 1030 1040 1050 1060 1070 1080 1090 1100

```
              ....|....|....|....|....|....|....|....|....|....|....|....|....|....|....|....|....|....|....|....|
KVJ10         AGGTGGTGCATGGCTGTCGTCAGCTCGTGTCGTGAGATGTTGGGTTAAGTCCCGCAACGAGCGCAACCCTCGTTTTTAGTTGCCAGCATTAAGTTGGGCA
KVJF8         AGGTGGTGCATGGCTGTCGTCAGCTCGTGTCGTGAGATGTTGGGTTAAGTCCCGCAACGAGCGCAACCCTCGTTTTTAGTTGCCAGCATTAAGTTGGGCA
KVJ3          AGGTGGTGCATGGCTGTCGTCAGCTCGTGTCGTGAGATGTTGGGTTAAGTCCCGCAACGAGCGCAACCCTCGTTTTTAGTTGCCAGCATTAAGTTGGGCA
SKS1          AGGTGGTGCATGGCTGTCGTCAGCTCGTGTCGTGAGATGTTGGGTTAAGTCCCGCAACGAGCGCAACCCTCGTTTTTAGTTGCCAGCATTAAGTTGGGCA
KVS1          AGGTGGTGCATGGCTGTCGTCAGCTCGTGTCGTGAGATGTTGGGTTAAGTCCCGCAACGAGCGCAACCCTCGTTTTTAGTTGCCAGCATTAAGTTGGGCA
SKJ1          AGGTGGTGCATGGCTGTCGTCAGCTCGTGTCGTGAGATGTTGGGTTAAGTCCCGCAACGAGCGCAACCCTCGTTTTTAGTTGCCAGCATTAAGTTGGGCA
KVJF4         AGGTGGTCCATGGCTGTCGTCAGCTCGTGTCGTGAGATGTTGGGTTAATTCCCGCAACGAGCGCAACCCTCGTTTTTAGTTGCCAGCATTAAGTTGGGCA
KVS11         AGGTGGTGCATGGCTGTCGTCAGCTCGTGTCGTGAGATGTTGGGTTAAGTCCCGCAACGAGCGCAACCCTCGTTTTTAGTTGCCAGCATTAAGTTGGGCA
KVJ20         AGGTGGTGCATGGCTGTCGTCAGCTCGTGTCGTGAGATGTTGGGTTAAGTCCCGCAACGAGCGCAACCCTCGTTTTTAGTTGCCAGCATTAAGTTGGGCA
SKSF3         AGGTGGTGCATGGCTGTCGTCAGCTCGTGTCGTGAGATGTTGGGTTAAGTCCCGCAACGAGCGCAACCCTCGTTTTTAGTTGCCAGCATTAAGTTGGGCA
SKSL1         AGGTGGTGCATGGCTGTCGTCAGCTCGTGTCGTGAGATGTTGGGTTAAGTCCCGCAACGAGCGCAACCCTCGTTTTTAGTTGCCAGCATTAAGTTGGGCA
SKSL2         AGGTGGTGCATGGCTGTCGTCAGCTCGTGTCGTGAGATGTTGGGTTAAGTCCCGCAACGAGCGCAACCCTCGTTTTTAGTTGCCAGCATTAAGTTGGGCA
KVJ4          AGGTGGTGCATGGCTGTCGTCAGCTCGTGTCGTGAGATGTTGGGTTAAGTCCCGCAACGAGCGCAACCCTCGTTTTTAGTTGCCAGCATTAAGTTGGGCA
KVJ2          AGGTGGTGCATGGCTGTCGTCAGCTCGTGTCGTGAGATGTTGGGTTAAGTCCCGCAACGAGCGCAACCCTCGTTTTTAGTTGCCAGCATTAAGTTGGGCA
KVSF4         AGGTGGTGCATGGCTGTCGTCAGCTCGTGTCGTGAGATGTTGGGTTAAGTCCCGCAACGAGCGCAACCCTCGTTTTTAGTTGCCAGCATTAAGTTGGGCA
SKS2          AGGTGGTGCATGGCTGTCGTCAGCTCGTGTCGTGAGATGTTGGGTTAAGTCCCGCAACGAGCGCAACCCTCGTTTTTAGTTGCCAGCATTAAGTTGGGCA
SKJF1         AGGTGGTGCATGGCTGTCGTCAGCTCGTGTCGTGAGATGTTGGGTTAAGTCCCGCAACGAGCGCAACCCTCGTTTTTAGTTGCCAGCATTAAGTTGGGCA
SKS3          AGGTGGTGCATGGCTGTCGTCAGCTCGTGTCGTGAGATGTTGGGTTAAGTCCCGCAACGAGCGCAACCCTCGTTTTTAGTTGCCAGCATTAAGTTGGGCA
SKJ6          AGGTGGTGCATGGCTGTCGTCAGCTCGTGTCGTGAGATGTTGGGTTAAGTCCCGCAACGAGCGCAACCCTCGTTTTTAGTTGCCAGCATTAAGTTGGGCA
SKJ2          AGGTGGTGCATGGCTGTCGTCAGCTCGTGTCGTGAGATGTTGGGTTAAGTCCCGCAACGAGCGCAACCCTCGTTTTTAGTTGCCAGCATTAAGTTGGGCA
SKS5          AGGTGGTGCATGGCTGTCGTCAGCTCGTGTCGTGAGATGTTGGGTTAAGTCCCGCAACGAGCGCAACCCTCGTTTTTAGTTGCCAGCATTAAGTTGGGCA
SKS9          AGGTGGTGCATGGCTGTCGTCAGCTCGTGTCGTGAGATGTTGGGTTAAGTCCCGCAACGAGCGCAACCCTCGTTTTTAGTTGCCAGCATTAAGTTGGGCA
KVJ18         AGGTGGTGCATGGCTGTCGTCAGCTCGTGTCGTGAGATGTTGGGTTAAGTCCCGCAACGAGCGCAACCCTCGTTTTTAGTTGCCAGCATTAAGTTGGGCA
SKJ4          AGGTGGTGCATGGCTGTCGTCAGCTCGTGTCGTGAGATGTTGGGTTAAGTCCCGCAACGAGCGCAACCCTCGTTTTTAGTTGCCAGCATTAAGTTGGGCA
SKSF1         AGGTGGTGCATGGCTGTCGTCAGCTCGTGTCGTGAGATGTTGGGTTAAGTCCCGCAACGAGCGCAACCCTCGTTTTTAGTTGCCAGCATTAAGTTGGGCA
KVSF7         AGGTGGTGCATGGCTGTCGTCAGCTCGTGTCGTGAGATGTTGGGTTAAGTCCCGCAACGAGCGCAACCCTCGTTTCTAGTTGCCAGCATTAAGATGGGAA
SKS8          AGGTGGTGCATGGCTGTCGTCAGCTCGTGTCGTGAGATGTTGGGTTAAGTCCCGCAACGAGCGCAACCCTCGTTTTTAGTTGCCATCATTAAGTTGGGCA
DQ185249      AGGTGGTGCATGGCTGTCGTCAGCTCGTGTCGTGAGATGTTGGGTTAAGTCCCGCAACGAGCGCAACCCTCGTTTTTAGTTGCCAGCATTAAGTTGGGCA
DQ185255      AGGTGGTGCATGGCTGTCGTCAGCTCGTGTCGTGAGATGTTGGGTTAAGTCCCGCAACGAGCGCAACCCTCGTTTTTAGTTGCCAGCATTAAGTTGGGCA
AY566855      AGGTGGTGCATGGCTGTCGTCAGCTCGTGTCGTGAGATGTTGGGTTAAGTCCCGCAACGAGCGCAACCCTCGTTTTTAGTTGCCAGCATTAAGTTGGGCA
AM711545      AGGTGGTGCATGGCTGTCGTCAGCTCGTGTCGTGAGATGTTGGGTTAAGTCCCGCAACGAGCGCAACCCTCGTTTTTAGTTGCCAGCATTAAGTTGGGCA
AM711536      AGGTGGTGCATGGCTGTCGTCAGCTCGTGTCGTGAGATGTTGGGTTAAGTCCCGCAACGAGCGCAACCCTCGTTTTTAGTTGCCAGCATTAAGTTGGGCA
DQ185256      AGGTGGTGCATGGCTGTCGTCAGCTCGTGTCGTGAGATGTTGGGTTAAGTCCCGCAACGAGCGCAACCCTCGTTTTTAGTTGCCAGCATTAAGTTGGGCA
DQ185245      AGGTGGTGCATGGCTGTCGTCAGCTCGTGTCGTGAGATGTTGGGTTAAGTCCCGCAACGAGCGCAACCCTCGTTTTTAGTTGCCAGCATTAAGTTGGGCA
AM711527      AGGTGGTGCATGGCTGTCGTCAGCTCGTGTCGTGAGATGTTGGGTTAAGTCCCGCAACGAGCGCAACCCTCGTTTTTAGTTGCCAGCATTAAGTTGGGCA
DQ185212      AGGTGGTGCATGGCTGTCGTCAGCTCGTGTCGTGAGATGTTGGGTTAAGTCCCGCAACGAGCGCAACCCTCGTTTTTAGTTGCCAGCATTAAGTTGGGCA
AF062638      AGGTGGTGCATGGCTGTCGTCAGCTCGTGTCGTGAGATGTTGGGTTAAGTCCCGCAACGAGCGCAACCCTCGTTTTTAGTTGCCAGCATTAAGTTGGGCA
HM623781      AGGTGGTGCATGGCTGTCGTCAGCTCGTGTCGTGAGATGTTGGGTTAAGTCCCGCAACGAGCGCAACCCTCGTTTTTAGTTGCCAGCATTAAGTTGGGCA
AM711540      AGGTGGTGCATGGCTGTCGTCAGCTCGTGTCGTGAGATGTTGGGTTAAGTCCCGCAACGAGCGCAACCCTCGTTTTTAGTTGCCAGCATTAAGTTGGGCA
DQ185254      AGGTGGTGCATGGCTGTCGTCAGCTCGTGTCGTGAGATGTTGGGTTAAGTCCCGCAACGAGCGCAACCCTCGTTTTTAGTTGCCAGCATTAAGTTGGGCA
DQ185251      AGGTGGTGCATGGCTGTCGTCAGCTCGTGTCGTGAGATGTTGGGTTAAGTCCCGCAACGAGCGCAACCCTCGTTTTTAGTTGCCAGCATTAAGTTGGGCA
AM711535      AGGTGGTGCATGGCTGTCGTCAGCTCGTGTCGTGAGATGTTGGGTTAAGTCCCGCAACGAGCGCAACCCTCGTTTTTAGTTGCCAGCATTAAGTTGGGCA
AF027653      AGGTGGTGCATGGCTGTCGTCAGCTCGTGTCGTGAGATGTTGGGTTAAGTCCCGCAACGAGCGCAACCCTCGTTTTTAGTTGCCAGCATTAAGTTGGGCA
DQ185244      AGGTGGTGCATGGCTGTCGTCAGCTCGTGTCGTGAGATGTTGGGTTAAGTCCCGCAACGAGCGCAACCCTCGTTTTTAGTTGCCAGCATTAAGTTGGGCA
AM711531      AGGTGGTGCATGGCTGTCGTCAGCTCGTGTCGTGAGATGTTGGGTTAAGTCCCGCAACGAGCGCAACCCTCGTTTTTAGTTGCCAGCATTAAGTTGGGCA
```

```
AJ630449     AGGTGGTGCATGGC-GTCGTCAGCTCGTGTCGTGAGATGTTGGGTTAAGTCCCGCAACGAGCGCAACCCTCGTTTTTAGTTGCCAGCATTAAGTTGGGCA
AY328897     AGGTGGTGCATGGCTGTCGTCAGCTCGTGTCGTGAGATGTTGGGTTAAGTCCCGCAACGAGCGCAACCCTCGTTTTTAGTTGCCAGCATTAAGTTGGGCA
AM230702     AGGTGGTGCATGGCTGTCGTCAGCTCGTGTCGTGAGATGTTGGGTTAAGTCCCGCAACGAGCGCAACCCTCGTTTTTAGTTGCCAGCATTAAGTTGGGCA
EF568907     AGGTGGTGCATGGCTGTCGTCAGCTCGTGTCGTGAGATGTTGGGTTAAGTCCCGCAACGAGCGCAACCCTCGTTTTTAGTTGCCAGCATTAAGTTGGGCA
AJ630458     AGGTGGTGCATGGCTGTCGTCAGCTCGTGTCGTGAGATGTTGGGTTAAGTCCCGCAACGAGCGCAACCCTCGTTTTTAGTTGCCAGCATTAAGTTGGGCA
GQ443447     AGGTGGTGCATGGCTGTCGTCAGCTCGTGTCGTGAGATGTTGGGTTAAGTCCCGCAACGAGCGCAACCCTCGTTTTTAGTTGCCAGCATTAAGTTGGGCA
AJ630457     AGGTGGTGCATGGCTGTCGTCAGCTCGTGTCGTGAGATGTTGGGTTAAGTCCCGCAACGAGCGCAACCCTCGTTTCTAGTTGCCAGCATTAAGATGGGAA
DQ234831     AGGTGGTGCATGGCTGTCGTCAGCTCGTGTCGTGAGATGTTGGGTTAAGTCCCGCAACGAGCGCAACCCTCGTTTCTAGTTGCCAGCATTAAGATGGGAA
AJ133161     AGGTGGTGCATGGCTGTCGTCAGCTCGTGTCGTGAGATGTTGGGTTAAGTCCCGCAACGAGCGCAACCCTCGTTTTTAGTTGCCATCATTAAGTTGGGCA
gi|47118302  AGGTGGTGCATGGCTGTCGTCAGCTCGTGTCGTGAGATGTTGGGTTAAGTCCCGCAACGAGCGCAACCCTCGTTTTTAGTTGCCAGCATTAAGTTGGGCA
DQ185209     AGGTGGTGCATGGCTGTCGTCAGCTCGTGTCGTGAGATGTTGGGTTAAGTCCCGCAACGAGCGCAACCCTCGTTTTTAGTTGCCAGCATTAAGTTGGGCA
AY742451     AGGTGGTGCATGGCTGTCGTCAGCTCGTGTCGTGAGATGTTGGGTTAAGTCCCGCAACGAGCGCAACCCTCGTTTTTAGTTGCCAGCATTAAGTTGGGCA
AF027655     AGGTGGTGCATGGCTGTCGTCAGCTCGTGTCGTGAGATGTTGGGTTAAGTCCCGCAACGAGCGCAACCCTCGTTTTTAGTTGCCAGCATTAAGTTGGGCA
DQ185211     AGGTGGTGCATGGCTGTCGTCAGCTCGTGTCGTGAGATGTTGGGTTAAGTCCCGCAACGAGCGCAACCCTCGTTTTTAGTTGCCAGCATTAAGTTGGGCA
AY742449     AGGTGGTGCATGGCTGTCGTCAGCTCGTGTCGTGAGATGTTGGGTTAAGTCCCGCAACGAGCGCAACCCTCGTTTTTAGTTGCCAGCATTAAGTTGGGCA
EF174212     AGGTGGTGCATGGCTGTCGTCAGCTCGTGTCGTGAGATGTTGGGTTAAGTCCCGCAACGAGCGCAACCCTCGTTTTTAGTTGCCAGCATTAAGTTGGGCA
DQ185213     AGGTGGTGCATGGCTGTCGTCAGCTCGTGTCGTGAGATGTTGGGTTAAGTCCCGCAACGAGCGCAACCCTCGTTTTTAGTTGCCAGCATTAAGTTGGGCA
AF506237     AGGTGGTGCATGGCTGTCGTCAGCTCGTGTCGTGAGATGTTGGGTTAAGTCCCGCAACGAGCGCAACCCTCGTGCTTAGTTGCCATCATTAAGTTGGGAA
AM230690     AGGTGGTGCATGGCTGTCGTCAGCTCGTGTCGTGAGATGTTGGGTTAAGTCCCGCAACGAGCGCAACCCTCGTCCTTAGTTGCCATCATTAAGTTGGGAA
AB039002     AGGTGGTGCATGGCTGTCGTCAGCTCGTGTCGTGAGATGTTGGGTTAAGTCCCGCAACGAGCGCAACCCTCGTTTTTAGTTGCCAGCATTAAGTTGGGCA
AJ293131     AGGTGGTGCATGGCTGTCGTCAGCTCGTGTCGTGAGATGTTGGGTTAAGTCCCGCAACGAGCGCAACCCTCGTTTTTAGTTGCCAGCATTAAGTTGGGCA
AJ133169     AGGTGCTGCATGGCTGTCGTCAGCTCGTGTCGTGAGATGTTGGGTTAAGTCCCGCAACGAGCGCAACCCTCGTCGTTAGTTGCCATCATTAAGTTGGGAA
DQ279769     AGGTGGTGCATGGCTGTCGTCAGCTCGTGTCGTGAGATGTTGGGTTAAGTCCCGCAACGAGCGCAACCCTCGTCCTTAGTTGCCATCATTCAGTTGGGCA
AJ000714     AGGTGGTGCATGGCTGTCGTCAGCTCGTGTCGTGAGATGTTGGGTTAAGTCCCGCAACGAGCGCAACCCTCGTTTTTAGTTGCCATCATTAAGTTGGGCA
KVSF5        AGGTGGTGCATGGCTGTCGTCAGCTCGTGTCGTGAGATGTTGGGTTAAGTCCCGCAACGAGCGCAACCCTCGTCTTTAGTTGCCATCATTAAGTTGGGAA

                       1110      1120      1130      1140      1150      1160      1170      1180      1190      1200
             ....|....|....|....|....|....|....|....|....|....|....|....|....|....|....|....|....|....|....|....|
KVJ10        CTCTAGAGAGACTGCCGGTGACAAACCGGAGGAAGGTGGGGATGACGTCAAGTCAGCATGCCCCTTACGCCTTGGG-CTACACACGTACTACAATGCTCC
KVJF8        CTCTAGAGAGACTGCCGGTGACAAACCGGAGGAAGGTGGGGATGACGTCAAGTCAGCATGCCCCTTACGCCTTGGG-CTACACACGTACTACAATGCTCC
KVJ3         CTCTAGAGAGACTGCCGGTGACAAACCGGAGGAAGGTGGGGATGACGTCAAGTCAGCATGCCCCTTACGCCTTGGG-CTACACACGTACTACAATGCTCC
SKS1         CTCTAGAGAGACTGCCGGTGACAAACCGGAGGAAGGTGGGGATGACGTCAAGTCAGCATGCCCCTTACGCCTTGGG-CTACACACGTACTACAATGCTCC
KVS1         CTCTAGAGAGACTGCCGGTGACAAACCGGAGGAAGGTGGGGATGACGTCAAGTCAGCATGCCCCTTACGCCTTGGG-CTACACACGTACTACAATGCTCC
SKJ1         CTCTAGAGAGACTGCCGGTGACAAACCGGAGGAAGGTGGGGATGACGTCAAGTCAGCATGCCCCTTACGCCTTGGG-CTACACACGTACTACAATGCTCC
KVJF4        CTCTAGAGAGACTGCCGGTGACAAACCGGAGGAAGGTGGGGATGACGTCAAGTCAGCATGCCCCTTACGCCTTGGG-CTACACACGTACTACAATGCTCC
KVS11        CTCTAGAGAGACTGCCGGTGACAAACCGGAGGAAGGTGGGGATGACGTCAAGTCAGCATGCCCCTTACGCCTTGGG-CTACACACGTACTACAATGCTCC
KVJ20        CTCTAGAGAGACTGCCGGTGACAAACCGGAGGAAGGTGGGGATGACGTCAAGTCAGCATGCCCCTTACGCCTTGGG-CTACACACGTACTACAATGCTCC
SKSF3        CTCTAGAGAGACTGCCGGTGACAAACCGGAGGAAGGTGGGGATGACGTCAAGTCAGCATGCCCCTTACGCCTTGGG-CTACACACGTACTACAATGCTCC
SKSL1        CTCTAGAGAGACTGCCGGTGACAAACCGGAGGAAGGTGGGGATGACGTCAAGTCAGCATGCCCCTTACGCCTTGGG-CTACACACGTACTACAATGCTCC
SKSL2        CTCTAGAGAGACTGCCGGTGACAAACCGGAGGAAGGTGGGGATGACGTCAAGTCAGCATGCCCCTTACGCCTTGGG-CTACACACGTACTACAATGCTCC
KVJ4         CTCTAGAGAGACTGCCGGTGACAAACCGGAGGAAGGTGGGGATGACGTCAAGTCAGCATGCCCCTTACGCCTTGGG-CTACACACGTACTACAATGCTCC
KVJ2         CTCTAGAGAGACTGCCGGTGACAAACCGGAGGAAGGTGGGGATGACGTCAAGTCAGCATGCCCCTTACGCCTTGGG-CTACACACGTACTACAATGCTCC
KVSF4        CTCTAGAGAGACTGCCGGTGACAAACCGGAGGAAGGTGGGGATGACGTCAAGTCAGCATGCCCCTTACGCCTTGGG-CTACACACGTACTACAATGCTCC
SKS2         CTCTAGAGAGACTGCCGGTGACAAACCGGAGGAAGGTGGGGATGACGTCAAGTCAGCATGCCCCTTACGCCTTGGG-CTACACACGTACTACAATGCTCC
SKJF1        CTCTAGAGAGACTGCCGGTGACAAACCGGAGGAAGGTGGGGATGACGTCAAGTCAGCATGCCCCTTACGCCTTGGG-CTACACACGTACTACAATGCTCC
SKS3         CTCTAGAGAGACTGCCGGTGACAAACCGGAGGAAGGTGGGGATGACGTCAAGTCAGCATGCCCCTTACGCCTTGGG-CTACACACGTACTACAATGCTCC
```

```
SKJ6         CTCTAGAGAGACTGCCGGTGACAAACCGGAGGAAGGTGGGGATGACGTCAAGTCAGCATGCCCCTTACGCCTTGGG-CTACACACGTACTACAATGCTCC
SKJ2         CTCTAGAGAGACTGCCGGTGACAAACCGGAGGAAGGTGGGGATGACGTCAAGTCAGCATGCCCCTTACGCCTTGGG-CTACACACGTACTACAATGCTCC
SKS5         CTCTAGAGAGACTGCCGGTGACAAACCGGAGGAAGGTGGGGATGACGTCAAGTCAGCATGCCCCTTACGCCTTGGG-CTACACACGTACTACAATGCTCC
SKS9         CTCTAGAGAGACTGCCGGTGACAAACCGGAGGAAGGTGGGGATGACGTCAAGTCAGCATGCCCCTTACGCCTTGGG-CTACACACGTACTACAATGCTCC
KVJ18        CTCTAGAGAGACTGCCGGTGACAAACCGGAGGAAGGTGGGGATGACGTCAAGTCAGCATGCCCCTTACGCCTTGGG-CTACACACGTACTACAATGCTCC
SKJ4         CTCTAGAGAGACTGCCGGTGACAAACCGGAGGAAGGTGGGGATGACGTCAAGTCAGCATGCCCCTTACGCCTTGGG-CTACACACGTACTACAATGCTCC
SKSF1        CTCTAGAGAGACTGCCGGTGACAAACCGGAGGAAGGTGAGGATGACGTCAAGTCAGCATGCCCCTTACGTCTTGGG-CTACACACGTACTACAATGCTAC
KVSF7        CTCTAGAGAGACTGCCGGTGACAAACCGGAGGAAGGTGGGGATGACGTCAAGTCAGCATGCCCCTTACGTCTTGGG-CTACACACGTACTACAATGCTAC
SKS8         CTCTAGAGAGACTGCCGGTGACAAACCGGAGGAAGGTGGGGATGACGTCAAGTCAGCATGCCCCTTACGTCTTGGG-CTACACACGTACTACAATGCTCC
DQ185249     CTCTAGAGAGACTGCCGGTGACAAACCGGAGGAAGGTGGGGATGACGTCAAGTCAGCATGCCCCTTACGCCTTGGG-CTACACACGTACTACAATGCTCC
DQ185255     CTCTAGAGAGACTGCCGGTGACAAACCGGAGGAAGGTGGGGATGACGTCAAGTCAGCATGCCCCTTACGCCTTGGG-CTACACACGTACTACAATGCTCC
AY566855     CTCTAGAGAGACTGCCGGTGACAAACCGGAGGAAGGTGGGGATGACGTCAAGTCAGCATGCCCCTTACGCCTTGGG-CTACACACGTACTACAATGCTCC
AM711545     CTCTAGAGAGACTGCCGGTGACAAACCGGAGGAAGGTGGGGATGACGTCAAGTCAGCATGCCCCTTACGCCTTGGG-CTACACACGTACTACAATGCTCC
AM711536     CTCTAGAGAGACTGCCGGTGACAAACCGGAGGAAGGTGGGGATGACGTCAAGTCAGCATGCCCCTTACGCCTTGGG-CTACACACGTACTACAATGCTCC
DQ185256     CTCTAGAGAGACTGCCGGTGACAAACCGGAGGAAGGTGGGGATGACGTCAAGTCAGCATGCCCCTTACGCCTTGGG-CTACACACGTACTACAATGCTCC
DQ185245     CTCTAGAGAGACTGCCGGTGACAAACCGGAGGAAGGTGGGGATGACGTCAAGTCAGCATGCCCCTTACGCCTTGGG-CTACACACGTACTACAATGCTCC
AM711527     CTCTAGAGAGACTGCCGGTGACAAACCGGAGGAAGGTGGGGATGACGTCAAGTCAGCATGCCCCTTACGCCTTGGG-CTACACACGTACTACAATGCTCC
DQ185212     CTCTAGAGAGACTGCCGGTGACAAACCGGAGGAAGGTGGGGATGACGTCAAGTCAGCATGCCCCTTACGCCTTGGG-CTACACACGTACTACAATGCTCC
AF062638     CTCTAGAGAGACTGCCGGTGACAAACCGGAGGAAGGTGGGGATGACGTCAAGTCAGCATGCCCCTTACGCCTTGGG-CTACACACGTACTACAATGCTCC
HM623781     CTCTAGAGAGACTGCCGGTGACAAACCGGAGGAAGGTGGGGATGACGTCAAGTCAGCATGCCCCTTACGCCTTGGG-CTACACACGTACTACAATGCTCC
AM711540     CTCTAGAGAGACTGCCGGTGACAAACCGGAGGAAGGTGGGGATGACGTCAAGTCAGCATGCCCCTTACGCCTTGGG-CTACACACGTACTACAATGCTCC
DQ185254     CTCTAGAGAGACTGCCGGTGACAAACCGGAGGAAGGTGGGGATGACGTCAAGTCAGCATGCCCCTTACGCCTTGGG-CTACACACGTACTACAATGCTCC
DQ185251     CTCTAGAGAGACTGCCGGTGACAAACCGGAGGAAGGTGGGGATGACGTCAAGTCAGCATGCCCCTTACGCCTTGGG-CTACACACGTACTACAATGCTCC
AM711535     CTCTAGAGAGACTGCCGGTGACAAACCGGAGGAAGGTGGGGATGACGTCAAGTCAGCATGCCCCTTACGCCTTGGG-CTACACACGTACTACAATGCTCC
AF027653     CTCTAGAGAGACTGCCGGTGACAAACCGGAGGAAGGTGGGGATGACGTCAAGTCAGCATGCCCCTTACGCCTTGGG-CTACACACGTACTACAATGCTCC
DQ185244     CTCTAGAGAGACTGCCGGTGACAAACCGGAGGAAGGTGGGGATGACGTCAAGTCAGCATGCCCCTTACGCCTTGGG-CTACACACGTACTACAATGCTCC
AM711531     CTCTAGAGAGACTGCCGGTGACAAACCGGAGGAAGGTGGGGATGACGTCAAGTCAGCATGCCCCTTACGCCTTGGG-CTACACACGTACTACAATGCTCC
AJ630449     CTCTAGAGAGACTGCCGGTGACAAACCGGAGGAAGGTGGGGATGACGTCAAGTCAGCATGCCCCTTACGCCTTGGG-CTACACACGTACTACAATGCTCC
AY328897     CTCTAGAGAGACTGCCGGTGACAAACCGGAGGAAGGTGGGGATGACGTCAAGTCAGCATGCCCCTTACGCCTTGGG-CTACACACGTACTACAATGCTCC
AM230702     CTSTAGAGAGACTGCCGGTGACAAACCGGAGGAAGGTGAGGATGACGTCAAGTCAGCATGCCCCTTACGTCTTGGG-CTACACACGTACTACAATGCTAC
EF568907     CTCTAGAGAGACTGCCGGTGACAAACCGGAGGAAGGTGAGGATGACGTCAAGTCAGCATGCCCCTTACGCCTTGGG-CTACACACGTACTACAATGCTAC
AJ630458     CTCTAGAGAGACTGCCGGTGACAAACCGGAGGAAGGTGAGGATGACGTCAAGTCAGCATGCCCCTTACGTCTTGGG-CTACACACGTACTACAATGCTAC
GQ443447     CTCTAGAGAGACTGCCGGTGACAAACCGGAGGAAGGTGAGGATGACGTCAAGTCAGCATGCCCCTTACGTCTTGGG-CTACACACGTACTACAATGCTAC
AJ630457     CTCTAGAGAGACTGCCGGTGACAAACCGGAGGAAGGTGGGGATGACGTCAAGTCAGCATGCCCCTTACGTCTTGGG-CTACACACGTACTACAATGCTAC
DQ234831     CTCTAGAGAGACTGCCGGTGACAAACCGGAGGAAGGTGGGGATGACGTCAAGTCAGCATGCCCCTTACGTCTTGGG-CTACACACGTACTACAATGCTAC
AJ133161     CTCTAGAGAGACTGCCGGTGACAAACCGGAGGAAGGTGGGGATGACGTCAAGTCAGCATGCCCCTTACGTCTTGGG-CTACACACGTACTACAATGCTCC
gi|47118302  CTCTAGAGAGACTGCCGGTGACAAACCGGAGGAAGGTGGGGATGACGTCAAGTCAGCATGCCCCTTACGTCTTGGG-CTACACACGTACTACAATGCTAC
DQ185209     CTCTAGAGAGACTGCCGGTGACAAACCGGAGGAAGGTGGGGATGACGTCAAGTCAGCATGCCCCTTACGCCTTGGG-CTACACACGTACTACAATGCTCC
AY742451     CTCTAGAGAGACTGCCGGTGACAAACCGGAGGAAGGTGGGGATGACGTCAAGTCAGCATGCCCCTTACGCCTTGGG-CTACACACGTACTACAATGCTCC
AF027655     CTCTAGAGAGACTGCCGGTGACAAACCGGAGGAAGGTGGGGATGACGTCAAGTCAGCATGCCCCTTACGCCTTGGG-CTACACACGTACTACAATGCTCC
DQ185211     CTCTAGAGAGACTGCCGGTGACAAACCGGAGGAAGGTGGGGATGACGTCAAGTCAGCATGCCCCTTACGCCTTGGG-CTACACACGTACTACAATGCTCC
AY742449     CTCTAGAGAGACTGCCGGTGACAAACCGGAGGAAGGTGGGGATGACGTCAAGTCAGCATGCCCCTTACGCCTTGGG-CTACACACGTACTACAATGCTCC
EF174212     CTCTAGAGAGACTGCCGGTGACAAACCGGAGGAAGGTGGGGATGACGTCAAGTCAGCATGCCCCTTACGCCTTGGG-CTACACACGTACTACAATGCTCC
DQ185213     CTCTAGAGAGACTGCCGGTGACAAACCGGAGGAAGGTGGGGATGACGTCAAGTCAGCATGCCCCTTACGCCTTGGG-CTACACACGTACTACAATGCTCC
AF506237     CTCTAGGGAGACTGCCGGTGACAAACCGGAGGAAGGTGGGGATGACGTCAAGTCAGCATGCCCCTTACGTCCTGGG-CTACACACGTACTACAATGCTAC
AM230690     CTCTAGGGAGACTGCCGGTGACAAACCGGAGGAAGGTGGGGATGACGTCAAGTCAGCATGCCCCTTACGTCCTGGG-CTACACACGTACTACAATGCTAC
```

```
AB039002    CTCTAGAGAGACTGCCGGTGACAAACCGGAGGAAGGTGGGGATGACGTCAAGTCAGCATGCCCCTTACGTCTTGGG-CTACACACGTACTACAATGCTAC
AJ293131    CTCTAGAGAGACTGCCGGTGACAAACCGGAGGAAGGTGGGGATGACGTCAAGTCAGCATGCCCCTTACGTCTTGGG-CTACACACGTACTACAATGCTAC
AJ133169    CTCTAGCGAGACTGCCGGTGACAAACCGGAGGAAGGTGAGGATGACGTCAAGTCAGCATGGCCCTTACGTCCTGGG-CGACACACGTACTACAATGCGAG
DQ279769    CTTTAGGGAGACTGCCGGTGACAAACCGGAGGAAGGTGGGGATGACGTCAAGTCATCATGCCCCTTACGTCCTGGGGCTACACACGTACTACAATGGGGG
AJ000714    CTCTAGAGAGACTGCCGGTGACAAACCGGAGGAAGGTGGGGATGACGTCAAGTCATCATGCCCCTTACGTCCTGGG-CTACACACGTACTACAATGGTAA
KVSF5       CTCTAGGGAGACTGCCGGTGACAAACCGGAGGAAGGTGGGGATGACGTCAAGTCAGCATGCCCCTTACGTCTTGGG-CTACACACGTACTACAATGCTAC

                     1210      1220      1230      1240      1250      1260      1270      1280      1290      1300
            ....|....|....|....|....|....|....|....|....|....|....|....|....|....|....|....|....|....|....|....|
KVJ10       GG-ACAGAGGGCAGCAAGCATGCGAATGCAAGCAAATCCCGTAAACCGGAGCTCAGTTCAGATCGCAGGCTGCAACTCGCCTGCGTGAAGGAGGAATCGC
KVJF8       GG-ACAGAGGGCAGCAAGCATGCGAATGCAAGCAAATCCCGTAAACCGGAGCTCAGTTCAGATCGCAGGCTGCAACTCGCCTGCGTGAAGGAGGAATCGC
KVJ3        GG-ACAGAGGGCAGCAAGCATGCGAATGCAAGCAAATCCCGTAAACCGGAGCTCAGTTCAGATCGCAGGCTGCAACTCGCCTGCGTGAAGGAGGAATCGC
SKS1        GG-ACAGAGGGCAGCAAGCATGCGAATGCAAGCAAATCCCGTAAACCGGAGCTCAGTTCAGATCGCAGGCTGCAACTCGCCTGCGTGAAGGAGGAATCGC
KVS1        GG-ACAGAGGGCAGCAAGCATGCGAATGCAAGCAAATCCCGTAAACCGGAGCTCAGTTCAGATCGCAGGCTGCAACTCGCCTGCGTGAAGGAGGAATCGC
SKJ1        GG-ACAGAGGGCAGCAAGCATGCGAATGCAAGCAAATCCCGTAAACCGGAGCTCAGTTCAGATCGCAGGCTGCAACTCGCCTGCGTGAAGGAGGAATCGC
KVJF4       GG-ACAGAGGGCAGCAAGCATGCGAATGCAAGCAAATCCCGTAAACCGGAGCTCAGTTCAGATCGCAGGCTGCAACTCGCCTGCGTGAAGGAGGAATCGC
KVS11       GG-ACAGAGGGCAGCAAGCATGCGAATGCAAGCAAATCCCGTAAACCGGAGCTCAGTTCAGATCGCAGGCTGCAACTCGCCTGCGTGAAGGAGGAATCGC
KVJ20       GG-ACAGAGGGCAGCAAGCATGCGAATGCAAGCAAATCCCGTAAACCGGAGCTCAGTTCAGATCGCAGGCTGCAACTCGCCTGCGTGAAGGAGGAATCGC
SKSF3       GG-ACAGAGGGCAGCAAGCATGCGAATGCAAGCAAATCCCGTAAACCGGAGCTCAGTTCAGATCGCAGGCTGCAACTCGCCTGCGTGAAGGAGGAATCGC
SKSL1       GG-ACAGAGGGCAGCAAGCATGCGAATGCAAGCAAATCCCGGAAACCGGAGCTCAGTTCAGATCGCAGGCTGCAACTCGCCTGCGTGAAGGAGGAATCGC
SKSL2       GG-ACAGAGGGCAGCAAGCATGCGAATGCAAGCAAATCCCGGAAACCGGAGCTCAGTTCAGATCGCAGGCTGCAACTCGCCTGCGTGAAGGAGGAATCGC
KVJ4        GG-ACAGAGGGCAGCAAGCATGCGAATGCAAGCAAATCCCGTAAACCGGAGCTCAGTTCAGATCGCAGGCTGCAACTCGCCTGCGTGAAGGAGGAATCGC
KVJ2        GG-ACAGAGGGCAGCAAGCATGCGAATGCAAGCAAATCCCGTAAACCGGAGCTCAGTTCAGATCGCAGGCTGCAACTCGCCTGCGTGAAGGAGGAATCGC
KVSF4       GG-ACAGAGGGCAGCAAGCATGCGAATGCAAGCAAATCCCGTAAACCGGAGCTCAGTTCAGATCGCAGGCTGCAACTCGCCTGCGTGAAGGAGGAATCGC
SKS2        GG-ACAGAGGGCAGCAAGCATGCGAATGCAAGCAAATCCCGTAAACCGGAGCTCAGTTCAGATCGCAGGCTGCAACTCGCCTGCGTGAAGGAGGAATCGC
SKJF1       GG-ACAGAGGGCAGCAAGCATGCGAATGCAAGCAAATCCCGGAAACCGGAGCTCAGTTCAGATCGCAGGCTGCAACTCGCCTGCGTGAAGGAGGAATCGC
SKS3        GG-ACAGAGGGCAGCAAGCATGCGAATGCAAGCAAATCCCGGAAACCGGAGCTCAGTTCAGATCGCAGGCTGCAACTCGCCTGCGTGAAGGAGGAATCGC
SKJ6        GG-ACAGAGGGCAGCAAGCATGCGAATGCAAGCAAATCCCGGAAACCGGAGCTCAGTTCAGATCGCAGGCTGCAACTCGCCTGCGTGAAGGAGGAATCGC
SKJ2        GG-ACAGAGGGCAGCAAGCATGCGAATGCAAGCAAATCCCGGAAACCGGAGCTCAGTTCAGATCGCAGGCTGCAACTCGCCTGCGTGAAGGAGGAATCGC
SKS5        GG-ACAGAGGGCAGCAAGCATGCGAATGCAAGCAAATCCCGGAAACCGGAGCTCAGTTCAGATCGCAGGCTGCAACTCGCCTGCGTGAAGGAGGAATCGC
SKS9        GG-ACAGAGGGCAGCAAGCATGCGAATGCAAGCAAATCCCGTAAACCGGAGCTCAGTTCAGATCGCAGGCTGCAACTCGCCTGCGTGAAGGAGGAATCGC
KVJ18       GG-ACAGAGGGCAGCAAGCATGCGAATGCAAGCAAATCCCGTAAACCGGAGCTCAGTTCAGATCGCAGGCTGCAACTCGCCTGCGTGAAGGAGGAATCGC
SKJ4        GG-ACAGAGGGCAGCAAGCATGCGAATGCAAGCAAATCCCGGAAACCGGAGCTCAGTTCAGATCGCAGGCTGCAACTCGCCTGCGTGAAGGAGGAATCGC
SKSF1       GG-ACAAAGGGCAGCTACACAGCGATGTGATGCAAATCTCATAAACCGTAGCTCAGTTCAGATCGCAGGCTGCAACTCGCCTGCGTGAAGGAGGAATCGC
KVSF7       GG-ACAAAGGGCAGCTACACAGCGATGTGATGCAAATCTCATAAACCGTAGCTCAGTTCAGATCGAAGGCTGCAACTCGCCTTCGTGAAGGAGGAATCGC
SKS8        GG-ACAAAGGGCAGCAAGCTAGTGATAGCAAGCAAATCCCATAAACCGGAGCTCAGTTCAGATCGAAGGCTGCAACTCGCCTTCGTGAAGTCGGAATCGC
DQ185249    GG-ACAGAGGGCAGCAAGCATGCGAATGCAAGCAAATCCCGTAAACCGGAGCTCAGTTCAGATCGCAGGCTGCAACTCGCCTGCGTGAAGGAGGAATCGC
DQ185255    GG-ACAGAGGGCAGCAAGCATGCGAATGCAAGCAAATCCCGTAAACCGGAGCTCAGTTCAGATCGCAGGCTGCAACTCGCCTGCGTGAAGGAGGAATCGC
AY566855    GG-ACAGAGGGCAGCAAGCATGCGAATGCAAGCAAATCCCGTAAACCGGAGCTCAGTTCAGATCGCAGGCTGCAACTCGCCTGCGTGAAGGAGGAATCGC
AM711545    GG-ACAGAGGGCAGCAAGCATGCGAATGCAAGCAAATCCCGTAAACCGGAGCTCAGTTCAGATCGCAGGCTGCAACTCGCCTGCGTGAAGGAGGAATCGC
AM711536    GG-ACAGAGGGCAGCAAGCATGCGAATGCAAGCAAATCCCGTAAACCGGAGCTCAGTTCAGATCGCAGGCTGCAACTCGCCTGCGTGAAGGAGGAATCGC
DQ185256    GG-ACAGAGGGCAGCAAGCATGCGAATGCAAGCAAATCCCGTAAACCGGAGCTCAGTTCAGATCGCAGGCTGCAACTCGCCTGCGTGAAGGAGGAATCGC
DQ185245    GG-ACAGAGGGCAGCAAGCATGCGAATGCAAGCAAATCCCGTAAACCGGAGCTCAGTTCAGATCGCAGGCTGCAACTCGCCTGCGTGAAGGAGGAATCGC
AM711527    GG-ACAGAGGGCAGCAAGCATGCGAATGCAAGCAAATCCCGTAAACCGGAGCTCAGTTCAGATCGCAGGCTGCAACTCGCCTGCGTGAAGGAGGAATCGC
DQ185212    GG-ACAGAGGGCAGCAAGCATGCGAATGCAAGCAAATCCCGTAAACCGGAGCTCAGTTCAGATCGCAGGCTGCAACTCGCCTGCGTGAAGGAGGAATCGC
AF062638    GG-ACAGAGGGCAGCAAGCATGCGAATGCAAGCAAATCCCGTAAACCGGAGCTCAGTTCAGATCGCAGGCTGCAACTCGCCTGCGTGAAGGAGGAATCGC
```

```
HM623781     GG-ACAGAGGGCAGCAAGCATGCGAATGCAAGCAAATCCCGTAAACCGGAGCTCAGTTCAGATCGCAGGCTGCAACTCGCCTGCGTGAAGGAGGAATCGC
AM711540     GG-ACAGAGGGCAGCAAGCATGCGAATGCAAGCAAATCCCGTAAACCGGAGCTCAGTTCAGATCGCAGGCTGCAACTCGCCTGCGTGAAGGAGGAATCGC
DQ185254     GG-ACAGAGGGCAGCAAGCATGCGAATGCAAGCAAATCCCGTAAACCGGAGCTCAGTTCAGATCGCAGGCTGCAACTCGCCTGCGTGAAGGAGGAATCGC
DQ185251     GG-ACAGAGGGCAGCAAGCATGCGAATGCAAGCAAATCCCGTAAACCGGAGCTCAGTTCAGATCGCAGGCTGCAACTCGCCTGCGTGAAGGAGGAATCGC
AM711535     GG-ACAGAGGGCAGCAAGCATGCGAATGCAAGCAAATCCCGTAAACCGGAGCTCAGTTCAGATCGCAGGCTGCAACTCGCCTGCGTGAAGGAGGAATCGC
AF027653     GG-ACAGAGGGCAGCAAGCATGCGAATGCAAGCAAATCCCGGAAACCGGAGCTCAGTTCAGATCGCAGGCTGCAACTCGCCTGCGTGAAGGAGGAATCGC
DQ185244     GG-ACAGAGGGCAGCAAGCATGCGAATGCAAGCAAATCCCGGAAACCGGAGCTCAGTTCAGATCGCAGGCTGCAACTCGCCTGCGTGAAGGAGGAATCGC
AM711531     GG-ACAGAGGGCAGCAAGCATGCGAATGCAAGCAAATCCCGTAAACCGGAGCTCAGTTCAGATCGCAGGCTGCAACTCGCCTGCGTGAAGGAGGAATCGC
AJ630449     GG-ACAGAGGGCAGCAAGCATGCGAATGCAAGCAAATCCCGTAAACCGGAGCTCAGTTCAGATCGCAGGCTGCAACTCGCCTGCGTGAAGGAGGAATCGC
AY328897     GG-ACAGAGGGCAGCAAGCATGCGAATGCAAGCAAATCCCGGAAACCGGAGCTCAGTTCAGATCGCAGGCTGCAACTCGCCTGCGTGAAGGAGGAATCGC
AM230702     RG-ACAAAGGGCAGCTACACAGCGATGTGATGYTWATCTCATAAACSGTAGCTCAGTTCAGATCGCAGGSTGCAACTYGCATGCMTGAAGGAGGAATCGC
EF568907     GG-ACAAAGGGCAGCTACACAGCGATGTGATGCTAATCTCATAAACCGTAGCTCAGTTCAGATCGCAGGCTGCAACTCGCCTGCGTGAAGGAGGAATCGC
AJ630458     GG-ACAAAGGGCAGCTACACAGCGATGTGATGCTAATCTCATAAACCGTAGCTCAGTTCAGATCGCAGGCTGCAACTCGCCTGCGTGAAGGAGGAATCGC
GQ443447     GG-ACAAAGGGCAGCTACACAGCGATGTGATGCTAATCTCATAAACCGTAGCTCAGTTCAGATCGCAGGCTGCAACTCGCCTGCGTGAAGGAGGAATCGC
AJ630457     GG-ACAAAGGGCAGCTACACAGCGATGTGATGCAAATCTCATAAACCGTAGCTCAGTTCAGATCGAAGGCTGCAACTCGCCTTCGTGAAGGAGGAATCGC
DQ234831     GG-ACAAAGGGCAGCTACACAGCGATGTGATGCAAATCTCATAAACCGTAGCTCAGTTCAGATCGAAGGCTGCAACTCGCCTTCGTGAAGGAGGAATCGC
AJ133161     GG-ACAAAGGGCAGCAAGCTAGTGATAGCAAGCAAATCCCATAAACCGGAGCTCAGTTCAGATCGAAGGCTGCAACTCGCCTTCGTGAAGTCGGAATCGC
gi|47118302  GG-ACAGAGGGCAGCAAGCTAGCGATAGCAAGCAAATCCCGTAAACCGTAGCTCAGTTCAGATCGCAGGCTGCAACTCGCCTGCGTGAAGGAGGAATCGC
DQ185209     GG-ACAGAGGGCAGCAAGCATGCGAATGCAAGCAAATCCCGTAAACCGGAGCTCAGTTCAGATCGCAGGCTGCAACTCGCCTGCGTGAAGGAGGAATCGC
AY742451     GG-ACAGAGGGCAGCAAGCATGCGAATGCAAGCAAATCCCGTAAACCGGAGCTCAGTTCAGATCGCAGGCTGCAACTCGCCTGCGTGAAGGAGGAATCGC
AF027655     GG-ACAGAGGGCAGCAAGCATGCGAATGCAAGCAAATCCCGTAAACCGGAGCTCAGTTCAGATCGCAGGCTGCAACTCGCCTGCGTGAAGGAGGAATCGC
DQ185211     GG-ACAGAGGGCAGCAAGCATGCGAATGCAAGCAAATCCCGTAAACCGGAGCTCAGTTCAGATCGCAGGCTGCAACTCGCCTGCGTGAAGGAGGAATCGC
AY742449     GG-ACAGAGGGCAGCAAGCATGCGAATGCAAGCAAATCCCGTAAACCGGAGCTCAGTTCAGATCGCAGGCTGCAACTCGCCTGCGTGAAGGAGGAATCGC
EF174212     GG-ACAGAGGGCAGCAAGCGGGCGACCGCAAGCAAATCCCGTAAACCGGAGCTCAGTTCAGATCGCAGGCTGCAACTCGCCTGCGTGAAGGAGGAATCGC
DQ185213     GG-ACAGAGGGCAGCAAGCATGCGAATGCAAGCAAATCCCGTAAACCGGAGCTCAGTTCAGATCGCAGGCTGCAACTCGCCTGCGTGAAGGAGGAATCGC
AF506237     AG-ACAATGGGAAGCTACACAGCGATGTGATGCGAAACTCATAAACTGTGGCTCAGTTCAGATTGCAGGCTGCAACTCGCCTGCATGAAGGAGGAATCGC
AM230690     AG-ACAATGGGAAGCTACACAGCGATGTGATACGAAACTCAGAAACTGTGGCTCAGTTCAGATTGCAGGCTGCAACTCGCCTGCATGAAGGAGGAATCGC
AB039002     GG-ACAAAGGGTAGCTACACAGCAATGTGATGCCAATCTCAGAAACCGTAGCTCAGTTCAGATCGCAGGCTGCAACTCGCCTGCGTGAAGTCGGAATCGC
AJ293131     GG-ACAAAGGGCAGCTACACAGCGATGTGATGCGAATCTCATAAACCGTAGCTCAGTTCAGATCGAAGGCTGCAACTCGCCTTCGTGAAGGAGGAATCGC
AJ133169     GG-ACAGAGAGCAGCCAACCCGCGAGGGAGAGCGAATCTCATAAACCCTGGCACAGTTCAGATTGCAGGCTGCAACTCGCCTGCATGAAGGAGGAATCGC
DQ279769     GGGACAAAGGGTAGCCAAGACGCGAGTCTGAGCCAATCCCGTAAACCTCTCCTCAGTTCAGATTGCAGGCTGCAACTCGCCTGCATGAAGGAGGAATCGC
AJ000714     GG-ACAAAGGGCAGCCACTTCGCGAGGAGGAGCTAATCCCGTAAACCTTGCCTCAGTTCAGATTGCAGGCTGCAACTCGCCTGCATGAAGGAGGAATCGC
KVSF5        AG-ACAATGGGAAGCTACACAGCGATGTGATGCGAAACTCAGAAACTGTGGCTCAGTTCAGATTGCAGGCTGCAACTCGCCTGCATGAAGGAGGAATCGC

                      1310      1320      1330      1340      1350      1360      1370      1380      1390      1400
             ....|....|....|....|....|....|....|....|....|....|....|....|....|....|....|....|....|....|....|....|
KVJ10        TAGTAATTGCAGGTCAGCATACTGCAGTGAATTCGTTCCCGGGCCTTGTACACACCGCCCGTCACACCATGGAAGCTGGTAGTGCCCGAAGTCATTACTC
KVJF8        TAGTAATTGCAGGTCAGCATACTGCAGTGAATTCGTTCCCGGGCCTTGTACACACCGCCCGTCACACCATGGAAGCTGGTAGTGCCCGAAGTCATTACTC
KVJ3         TAGTAATTGCAGGTCAGCATACTGCAGTGAATTCGTTCCCGGGCCTTGTACACACCGCCCGTCACACCATGGAAGCTGGTAGTGCCCGAAGTCATTACTC
SKS1         TAGTAATTGCAGGTCAGCATACTGCAGTGAATTCGTTCCCGGGCCTTGTACACACCGCCCGTCACACCATGGAAGCTGGTAGTGCCCGAAGTCATTACTC
KVS1         TAGTAATTGCAGGTCAGCATACTGCAGTGAATTCGTTCCCGGGCCTTGTACACACCGCCCGTCACACCATGGAAGCTGGTAGTGCCCGAAGTCATTACTC
SKJ1         TAGTAATTGCAGGTCAGCATACTGCAGTGAATTCGTTCCCGGGCCTTGTACACACCGCCCGTCACACCATGGAAGCTGGTAGTGCCCGAAGTCATTACTC
KVJF4        TAGTAATTGCAGGTCAGCATACTGCAGTGAATTCGTTCCCGGGCCTTGTACACACCGCCCGTCACACCATGGAAGCTGGTAGTGCCCGAAGTCATTACTC
KVS11        TAGTAATTGCAGGTCAGCATACTGCAGTGAATTCGTTCCCGGGCCTTGTACACACCGCCCGTCACACCATGGAAGCTGGTAGTGCCCGAAGTCATTACTC
KVJ20        TAGTAATTGCAGGTCAGCATACTGCAGTGAATTCGTTCCCGGGCCTTGTACACACCGCCCGTCACACCATGGAAGCTGGTAGTGCCCGAAGTCATTACTC
SKSF3        TAGTAATTGCAGGTCAGCATACTGCAGTGAATTCGTTCCCGGGCCTTGTACACACCGCCCGTCACACCATGGAAGCTGGTAGTGCCCGAAGTCATTACTC
```

```
SKSL1        TAGTAATTGCAGGTCAGCATACTGCAGTGAATTCGTTCCCGGGCCTTGTACACACCGCCCGTCACACCATGGAAGCTGGTAGTGCCCGAAGTCATTACTC
SKSL2        TAGTAATTGCAGGTCAGCATACTGCAGTGAATTCGTTCCCGGGCCTTGTACACACCGCCCGTCACACCATGGAAGCTGGTAGTGCCCGAAGTCATTACTC
KVJ4         TAGTAATTGCAGGTCAGCATACTGCAGTGAATTCGTTCCCGGGCCTTGTACACACCGCCCGTCACACCATGGAAGCTGGTAGTGCCCGAAGTCATTACTC
KVJ2         TAGTAATTGCAGGTCAGCATACTGCAGTGAATTCGTTCCCGGGCCTTGTACACACCGCCCGTCACACCATGGAAGCTGGTAGTGCCCGAAGTCATTACTC
KVSF4        TAGTAATTGCAGGTCAGCATACTGCAGTGAATTCGTTCCCGGGCCTTGTACACACCGCCCGTCACACCATGGAAGCTGGTAGTGCCCGAAGTCATTACTC
SKS2         TAGTAATTGCAGGTCAGCATACTGCAGTGAATTCGTTCCCGGGCCTTGTACACACCGCCCGTCACACCATGGAAGCTGGTAGTGCCCGAAGTCATTACTC
SKJF1        TAGTAATTGCAGGTCAGCATACTGCAGTGAATTCGTTCCCGGGCCTTGTACACACCGCCCGTCACACCATGGAAGCTGGTAGTGCCCGAAGTCATTACTC
SKS3         TAGTAATTGCAGGTCAGCATACTGCAGTGAATTCGTTCCCGGGCCTTGTACACACCGCCCGTCACACCATGGAAGCTGGTAGTGCCCGAAGTCATTACTC
SKJ6         TAGTAATTGCAGGTCAGCATACTGCAGTGAATTCGTTCCCGGGCCTTGTACACACCGCCCGTCACACCATGGAAGCTGGTAGTGCCCGAAGTCATTACTC
SKJ2         TAGTAATTGCAGGTCAGCATACTGCAGTGAATTCGTTCCCGGGCCTTGTACACACCGCCCGTCACACCATGGAAGCTGGTAGTGCCCGAAGTCATTACTC
SKS5         TAGTAATTGCAGGTCAGCATACTGCAGTGAATTCGTTCCCGGGCCTTGTACACACCGCCCGTCACACCATGGAAGCTGGTAGTGCCCGAAGTCATTACTC
SKS9         TAGTAATTGCAGGTCAGCATACTGCAGTGAATTCGTTCCCGGGCCTTGTACACACCGCCCGTCACACCATGGAAGCTGGTAGTGCCCGAAGTCATTACTC
KVJ18        TAGTAATTGCAGGTCAGCATACTGCAGTGAATTCGTTCCCGGGCCTTGTACACACCGCCCGTCACACCATGGAAGCTGGTAGTGCCCGAAGTCATTACTC
SKJ4         TAGTAATTGCAGGTCAGCATACTGCAGTGAATTCGTTCCCGGGCCTTGTACACACCGCCCGTCACACCATGGAAGCTGGTAGTGCCCGAAGTCATTACTC
SKSF1        TAGTAATTGCAGGTCAGCATACTGCAGTGAATTCGTTCCCGGGCCTTGTACACACCGCCCGTCACACCATGGAAGTTGGTCACGCCCGAAGTCATTACCC
KVSF7        TAGTAATTGCAGGTCAGCATACTGCAGTGAATTCGTTCCCGGGCCTTGTACACACCGCCCGTCACACCATGGAAGTCGGTCACGCCCGAAGTCATTACCC
SKS8         TAGTAATTGCAGGTCAGCATACTGCAGTGAATTCGTTCCCGGGCCTTGTACACACCGCCCGTCACACCATGGAAGCTGGCAACGCCCGAAGTCATTACCC
DQ185249     TAGTAATTGCAGGTCAGCATACTGCAGTGAATTCGTTCCCGGGCCTTGTACACACCGCCCGTCACACCATGGAAGCTGGTAGTGCCCGAAGTCATTACTC
DQ185255     TAGTAATTGCAGGTCAGCATACTGCAGTGAATTCGTTCCCGGGCCTTGTACACACCGCCCGTCACACCATGGAAGCTGGTAGTGCCCGAAGTCATTACTC
AY566855     TAGTAATTGCAGGTCAGCATACTGCAGTGAATTCGTTCCCGGGCCTTGTACACACCGCCCGTCACACCATGGAAGCTGGTAGTGCCCGAAGTCATTACTC
AM711545     TAGTAATTGCAGGTCAGCATACTGCAGTGAATTCGTTCCCGGGCCTTGTACACACCGCCCGTCACACCATGGAAGCTGGTAGTGCCCGAAGTCATTACTC
AM711536     TAGTAATTGCAGGTCAGCATACTGCAGTGAATTCGTTCCCGGGCCTTGTACACACCGCCCGTCACACCATGGAAGCTGGTAGTGCCCGAAGTCATTACTC
DQ185256     TAGTAATTGCAGGTCAGCATACTGCAGTGAATTCGTTCCCGGGCCTTGTACACACCGCCCGTCACACCATGGAAGCTGGTAGTGCCCGAAGTCATTACTC
DQ185245     TAGTAATTGCAGGTCAGCATACTGCAGTGAATTCGTTCCCGGGCCTTGTACACACCGCCCGTCACACCATGGAAGCTGGTAGTGCCCGAAGTCATTACTC
AM711527     TAGTAATTGCAGGTCAGCATACTGCAGTGAATTCGTTCCCGGGCCTTGTACACACCGCCCGTCACACCATGGAAGCTGGTAGTGCCCGAAGTCATTACTC
DQ185212     TAGTAATTGCAGGTCAGCATACTGCAGTGAATTCGTTCCCGGGCCTTGTACACACCGCCCGTCACACCATGGAAGCTGGTAGTGCCCGAAGTCATTACTC
AF062638     TAGTAATTGCAGGTCAGCATACTGCAGTGAATTCGTTCCCGGGCCTTGTACACACCGCCCGTCACACCATGGAAGGTGGTAGTGCCCGAAGTCATTACTC
HM623781     TAGTAATTGCAGGTCAGCATACTGCAGTGAATTCGTTCCCGGGCCTTGTACACACCGCCCGTCACACCATGGAAGCTGGTAGTGCCCGAAGTCATTACTC
AM711540     TAGTAATTGCAGGTCAGCATACTGCAGTGAATTCGTTCCCGGGCCTTGTACACACCGCCCGTCACACCATGGAAGCTGGTAGTGCCCGAAGTCATTACTC
DQ185254     TAGTAATTGCAGGTCAGCATACTGCAGTGAATTCGTTCCCGGGCCTTGTACACACCGCCCGTCACACCATGGAAGCTGGTAGTGCCCGAAGTCATTACTC
DQ185251     TAGTAATTGCAGGTCAGCATACTGCAGTGAATTCGTTCCCGGGCCTTGTACACACCGCCCGTCACACCATGGAAGCTGGTAGTGCCCGAAGTCATTACTC
AM711535     TAGTAATTGCAGGTCAGCATACTGCAGTGAATTCGTTCCCGGGCCTTGTACACACCGCCCGTCACACCATGGAAGCTGGTAGTGCCCGAAGTCATTACTC
AF027653     TAGTAATTGCAGGTCAGCATACTGCAGTGAATTCGTTCCCGGGCCTTGTACACACCGCCCGTCACACCATGGAAGCTGGTAGTGCCCGAAGTCATTACTC
DQ185244     TAGTAATTGCAGGTCAGCATACTGCAGTGAATTCGTTCCCGGGCCTTGTACACACCGCCCGTCACACCATGGAAGCTGGTAGTGCCCGAAGTCATTACTC
AM711531     TAGTAATTGCAGGTCAGCATACTGCAGTGAATTCGTTCCCGGGCCTTGTACACACCGCCCGTCACACCATGGAAGCTGGTAGTGCCCGAAGTCATTACTC
AJ630449     TAGTAATTGCAGGTCAGCATACTGCAGTGAATTCGTTCCCGGGCCTTGTACACACCGCCCGTCACACCATGGAAGCTGGTAGTGCCCGAAGTCATTACTC
AY328897     TAGTAATTGCAGGTCAGCATACTGCAGTGAATTCGTTCCCGGGCCTTGTACACACCGCCCGTCACACCATGGAAGCTGGTAGTGCCCGAAGTCATTACTC
AM230702     TAGTAATTGCAGGTCAGCATACTGCAGTGAATTCGTTCSCGGGCCTTGTACACACCGCCCGTCACACCATGGAAGTYGGTCACGCCCGAAGTCGTTACCC
EF568907     TAGTAATTGCAGGTCAGCATACTGCAGTGAATTCGTTCCCGGGCCTTGTACACACCGCCCGTCACACCATGGAAGTTGGTCACGCCCGAAGTCGTTACCC
AJ630458     TAGTAATTGCAGGTCAGCATACTGCAGTGAATTCGTTCCCGGGCCTTGTACACACCGCCCGTCACACCATGGAAGTTGGTCACGCCCGAAGTCATTACCC
GQ443447     TAGTAATTGCAGGTCAGCATACTGCAGTGAATTCGTTCCCGGGCCTTGTACACACCGCCCGTCACACCATGGAAGTTGGTCACGCCCGAAGTCATTACCC
AJ630457     TAGTAATTGCAGGTCAGCATACTGCAGTGAATTCGTTCCCGGGCCTTGTACACACCGCCCGTCACACCATGGAAGTCGGTCACGCCCGAAGTCATTACCC
DQ234831     TAGTAATTGCAGGTCAGCATACTGCAGTGAATTCGTTCCCGGGCCTTGTACACACCGCCCGTCACACCATGGAAGTCGGTCACGCCCGAAGTCATTACCC
AJ133161     TAGTAATTGCAGGTCAGCATACTGCAGTGAATTCGTTCCCGGGCCTTGTACACACCGCCCGTCACACCATGGAAGCTGGCAACGCCCGAAGTCATTACCC
gi|47118302  TAGTAATTGCAGGTCAGCATACTGCAGTGAATTCGTTCCCGGGCCTTGTACACACCGCCCGTCACACCATGGAAGCTGGCAACGCCCGAAGTCATTACTC
DQ185209     TAGTAATTGCAGGTCAGCATACTGCAGTGAATTCGTTCCCGGGCCTTGTACACACCGCCCGTCACACCATGGAAGCTGGTAGTGCCCGAAGTCATTACTC
```

```
AY742451   TAGTAATTGCAGGTCAGCATACTGCAGTGAATTCGTTCCCGGGCCTTGTACACACCGCCCGTCACACCATGGAAGCTGGTAGTGCCCGAAGTCATTACTC
AF027655   TAGTAATTGCAGGTCAGCATACTGCAGTGAATTCGTTCCCGGGCCTTGTACACACCGCCCGTCACACCATGGAAGCTGGTAGTGCCCGAAGTCATTACTC
DQ185211   TAGTAATTGCAGGTCAGCATACTGCAGTGAATTCGTTCCCGGGCCTTGTACACACCGCCCGTCACACCATGGAAGCTGGTAGTGCCCGAAGTCATTACTC
AY742449   TAGTAATTGCAGGTCAGCATACTGCAGTGAATTCGTTCCCGGGCCTTGTACACACCGCCCGTCACACCATGGAAGCTGGTAGTGCCCGAAGTCATTACTC
EF174212   TAGTAATTGCAGGTCAGCATACTGCAGTGAATTCGTTCCCGGGCCTTGTACACACCGCCCGTCACACCATGGAAGCTGGTAGTGCCCGAAGTCATTACTC
DQ185213   TAGTAATTGCAGGTCAGCATACTGCAGTGAATTCGTTCCCGGGCCTTGTACACACCGCCCGTCACACCATGGAAGCTGGTAGTGCCCGAAGTCATTACTC
AF506237   TAGTAATTGCAGGTCAGCATACTGCAGTGAATTCGTTCCCGGGCCTTGTACACACCGCCCGTCACACCATGGAAGCTGGTAGTGCCCGAAGTCGTTACCC
AM230690   TAGTAATTGCAGGTCAGCATACTGCAGTGAATTCGTTCCCGGGCCTTGTACACACCGCCCGTCACACCATGGAAGCTGGTAGTGCCCGAAGTCGTTACCC
AB039002   TAGTAATTGCAGGTCAGCATACTGCAGTGAATTCGTTCCCGGGCCTTGTACACACCGCCCGTCACACCATGGAAGCTGGTCACGCCCGAAGTCGTTACCC
AJ293131   TAGTAATTGCAGGTCAGCATACTGCAGTGAATTCGTTCCCGGGCCTTGTACACACCGCCCGTCACACCATGGAAGTTGGTCACGCCCGAAGTCGTTACCC
AJ133169   TAGTAATCGCAGGTCAGCATACTGCGGTGAATCCGTTCCCGGGCCTTGTACACACCGCCCGTCACACCATGGAAGTGAGCCACGCCCGAAGTCATTACTC
DQ279769   TAGTAATCGCAGGTCAGCATACTGCGGTGAATCCGTTCCCGGGCCTTGTACACACCGCCCGTCACACCATGGAAGTTAGCCACGCCCGAAGTCGTTACTC
AJ000714   TAGTAATCGCAGGTCAGCATACTGCGGTGAATCCGTTCCCGGGCCTTGTACACACCGCCCGTCACACCATGGAAGTTAACCATGCCCGAAGTCATTACTC
KVSF5      TAGTAATTGCAGGTCAGCATACTGCAGTGAATTCGTTCCCGGGCCTTGTACACACCGCCCGTCACACCATGGAAGCTGGTAGTGCCCGAAGTCGTTACCC

                   1410      1420      1430      1440      1450      1460      1470      1480      1490      1500
           ....|....|....|....|....|....|....|....|....|....|....|....|....|....|....|....|....|....|....|....|
KVJ10      CAACCTTTCGGGGAGGAGGATGCCTAAGGCAGGACTGGTGACTGGGGTGAAGTCGTAACAAGGTAGCCGTACCGGAAGGTGTGGCTGGATCACCTCCTT-
KVJF8      CAACCTTTCGGGGAGGAGGATGCCTAAGGCAGGACTGGTGACTGGGGTGAAGTCGTAACAAGGTAGCCGTACCGGAAGGTGTGGCTGGATCACCTCCTT-
KVJ3       CAACCATTCGTGGAGGAGGATGCCTAAGGCAGGACTGGTGACTGGGGTGAAGTCGTAACAAGGTAGCCGTACCGGAAGGTGTGGCTGGATCACCTCCTT-
SKS1       CAACCATTCGTGGAGGAGGATGCCTAAGGCAGGACTGGTGACTGGGGTGAAGTCGTAACAAGGTAGCCGTACCGGAAGGTGTGGCTGGATCACCTCCTT-
KVS1       CAACCATTCGTGGAGGAGGATGCCTAAGGCAGGACTGGTGACTGGGGTGAAGTCGTAACAAGGTAGCCGTACCGGAAGGTGTGGCTGGATCACCTCCTT-
SKJ1       CAACCATTCGTGGAGGAGGATGCCTAAGGCAGGACTGGTGACTGGGGTGAAGTCGTAACAAGGTAGCCGTACCGGAAGGTGTGGCTGGATCACCTCCTT-
KVJF4      CAACCATTCGTGGAGGAGGATGCCTAAGGCAGGACTGGTGACTGGGGTGAAGTCGTAACAAGGTAGCCGTACCGGAAGGTGTGGCTGGATCACCTCCTT-
KVS11      CAACCATTCGTGGAGGAGGATGCCTAAGGCAGGACTGGTGACTGGGGTGAAGTCGTAACAAGGTAGCCGTACCGGAAGGTGTGGCTGGATCACCTCCTT-
KVJ20      CAACCTTTCGGGGAGGAGGATGCCTAAGGCAGGACTGGTGACTGGGGTGAAGTCGTAACAAGGTAGCCGTACCGGAAGGTGTGGCTGGATCACCTCCTT-
SKSF3      CAACCTTTCGGGGAGGAGGATGCCTAAGGCAGGACTGGTGACTGGGGTGAAGTCGTAACAAGGTAGCCGTACCGGAAGGTGTGGCTGGATCACCTCCTT-
SKSL1      CAACCCTTCGGGGAGGAGGATGCCTAAGGCAGGACTGGTGACTGGGGTGAAGTCGTAACAAGGTAGCCGTACCGGAAGGTGTGGCTGGATCACCTCCTT-
SKSL2      CAACCCTTCGGGGAGGAGGATGCCTAAGGCAGGACTGGTGACTGGGGTGAAGTCGTAACAAGGTAGCCGTACCGGAAGGTGTGGCTGGATCACCTCCTT-
KVJ4       CAACCATTCGTGGAGGAGGATGCCTAAGGCAGGACTGGTGACTGGGGTGAAGTCGTAACAAGGTAGCCGTACCGGAAGGTGTGGCTGGATCACCTCCTT-
KVJ2       CAACCATTCGTGGAGGAGGATGCCTAAGGCAGGACTGGTGACTGGGGTGAAGTCGTAACAAGGTAGCCGTACCGGAAGGTGTGGCTGGATCACCTCCTT-
KVSF4      CAACCATTCGTGGAGGAGGATGCCTAAGGCAGGACTGGTGACTGGGGTGAAGTCGTAACAAGGTAGCCGTACCGGAAGGTGTGGCTGGATCACCTCCTT-
SKS2       CAACCTTTCGGGGAGGAGGATGCCTAAGGCAGGACTGGTGACTGGGGTGAAGTCGTAACAAGGTAGCCGTACCGGAAGGTGTGGCTGGATCACCTCCTT-
SKJF1      CAACCATTCGTGGAGGAGGATGCCTAAGGCAGGACTGGTGACTGGGGTGAAGTCGTAACAAGGTAGCCGTACCGGAAGGTGTGGCTGGATCACCTCCTT-
SKS3       CAACCATTCGTGGAGGAGGATGCCTAAGGCAGGACTGGTGACTGGGGTGAAGTCGTAACAAGGTAGCCGTACCGGAAGGTGTGGCTGGATCACCTCCTT-
SKJ6       CAACCATTCGTGGAGGAGGATGCCTAAGGCAGGACTGGTGACTGGGGTGAAGTCGTAACAAGGTAGCCGTACCGGAAGGTGTGGCTGGATCACCTCCTT-
SKJ2       CAACCTTTCGGGGAGGAGGATGCCTAAGGCAGGACTGGTGACTGGGGTGAAGTCGTAACAAGGTAGCCGTACCGGAAGGTGTGGCTGGATCACCTCCTT-
SKS5       CAACCTTTCGGGGAGGAGGATGCCTAAGGCAGGACTGGTGACTGGGGTGAAGTCGTAACAAGGTAGCCGTACCGGAAGGTGTGGCTGGATCACCTCCTT-
SKS9       CAACCATTCGTGGAGGAGGATGCCTAAGGCAGGACTGGTGACTGGGGTGAAGTCGTAACAAGGTAGCCGTACCGGAAGGTGTGGCTGGATCACCTCCTT-
KVJ18      CAACCATTCGTGGAGGAGGATGCCTAAGGCAGGACTGGTGACTGGGGTGAAGTCGTAACAAGGTAGCCGTACCGGAAGGTGTGGCTGGATCACCTCCTT-
SKJ4       CAACCATTCGTGGAGGAGGATGCCTAAGGCAGGACTGGTGACTGGGGTGAAGTCGTAACAAGGTAGCCGTACCGGAAGGTGTGGCTGGATCACCTCCTT-
SKSF1      CAACCGTAAGGAG-GGGG-ATGCCTAAGGTAGGACTAGTGACTGGGGTGAAGTCGTAACAAGGTAGCCGTACCGGAAGGTGTGGCTGGATCACCTCCTT-
KVSF7      CAACCTTTTTGGA-GGGGGATGCCTAAGGTAGGACTGGTGACTGGGGTGAAGTCGTAACAAGGTAGCCGTACCGGAAGGTGTGGCTGGATCACCTCCTT-
SKS8       CAACTTTTCGGAGAGGGGGATGCCTAAGGCAGTGCTGGTGACTGGGGTGAAGTCGTAACAAGGTAGCCGTACCGGAAGGTGTGGCTGGATCACCTCCTT-
DQ185249   CAACCTTTCGGGGGGGAGGATGCCTAAGGCAGGACTGGTGACTGGGGTGAAGTCGTAACAAGGTAGCCGTAC----------------------------
DQ185255   CAACCTTTCGGGGAGGAGGATGCCTAAGGCAGGACTGGTGACTGGGGTGAAGTCGTAACAAGGTAGCCGTACCGGAAGG----------------------
```

```
AY566855      CAACCATTCGTGGAGGAGGATGCCTAAGGCAGGACTGGTGACTGGGGTGAAGTCGTAACAAGGTAGCCGTA-----------------------------
AM711545      CAACCATTCGTGGAGGAGGATGCCTAAGGCAGGACTGGTGACTGGGGTGAAGTCGTAACAAGGTAGCCGTACCGGAAGGTGTGGCTGGATCACCTCCTTT
AM711536      CAACCATTCGTGGAGGAGGATGCCTAAGGCAGGACTGGTGACTGGGGTGAAGTCGTAACAAGGTAGCCGTACCGGAAGGTGTGGCTGGATCACCTCCTTT
DQ185256      CAACCATTCGTGGAGGAGGATGCCTAAGGCAGGACTGGTGACTGGGGTGAAGTCGTAACAA---------------------------------------
DQ185245      CAACCATTCGTGGAGGAGGATGCCTAAGGCAGGACTGGTGACTGGGGTGAAGTCGTAACAAGGTAGCCGTACCGGAAGG---------------------
AM711527      CAACCATTCGTGGAGGAGGATGCCTAAGGCAGGACTGGTGACTGGGGTGAAGTCGTAACAAGGTAGCCGTACCGGAAGGTGTGGCTGGATCACCTCCTTT
DQ185212      CAACCATTCGTGGAGGAGGATGCCTAAGGCAGGACTGGTGACTGGGGTGAAGTCGTAACAAGGTAGCCGTACCGGAA-----------------------
AF062638      CAACCATTCGTGGAGGAGGATGCCTAAGGCAGGACTGGTGACTGGGGTGAAGTCGTAACAAGGTAGCCGTACCGGAAGGTGCGGCTGGATCACCTCCTT-
HM623781      CAACCTTTCGGGGAGGAGGATGCCTAAGGCAGGACTGGTGACTGGGGTGAAGTCGTAACAAGGTAGCCGTACC---------------------------
AM711540      CAACCTTTCGGGGAGGAGGATGCCTAAGGCAGGACTGGTGACTGGGGTGAAGTCGTAACAAGGTAGCCGTACCGGAAGGTGTGGCTGGATCACCTCCTTT
DQ185254      CAACCTTTCGGGGAGGAGGATGCCTAAGGCAGGACTGGTGACTGGGGTGAAGTCGTAACAA---------------------------------------
DQ185251      CAACCTTTCGGGGGGGAGGATGCCTAAGGCAGGACTGGTGACTGGGGTGAAGTCGTAACAAGGTAGCCGTACCGGAA-----------------------
AM711535      CAACCTTTCGGGGGGGAGGATGCCTAAGGCAGGACTGGTGACTGGGGTGAAGTCGTAACAAGGTAGCCGTACCGGAAGGTGTGGCTGGATCACCTCCTTT
AF027653      CAACCCTTCGGGGAGGAGGATGCCTAAGGCAGGACTGGTGACTGGGGT----------------------------------------------------
DQ185244      CAACCCTTCGGGGAGGAGGATGCCTAAGGCAGGACTGGTGACTGGGGTGAAGTCGTAACAAGGTAGCCGTACC---------------------------
AM711531      CAACCTTTCGGGGAGGAGGATGCCTAAGGCAGGACTGGTGACTGGGGTGAAGTCGTAACAAGGTAGCCGTACCGGAAGGTGTGGCTGGATCACCTCCTTT
AJ630449      CAACCTTTCGGGGAGGAGGATGCCTAAGGCAGGACTGGTGACTGGGGTGAAGTCGTAACAAGGTAGCCGTACCGGAAGGTGTGGCTGGATCACCTCCTTT
AY328897      CAACCATTCGTGGAGGAGGATGCCTAAGGCAGGACTGGTGACTGGGGT----------------------------------------------------
AM230702      CAACCGCWWGGAG-GGGGGATGCCTAAGGYAGGACTGGTGACTGGGGTGAAGTCGAACAAGGA-------------------------------------
EF568907      CAACCGAAAGGAG-GGGG-ATGCCTAA-------------------------------------------------------------------------
AJ630458      CAACCGTAAGGAG-GGGG-ATGCCTAAGGTAGGACTGGTGACTGGGGTGAAGTCGTAACAAGGTAGCCGTACCGGAAGGTGTGGCTGGATCACCTCCTTT
GQ443447      CAACCGAAAGGAG-GGGG-ATGCCTAAGGTAGGACTGGTGACTGGGGTGAAGT-----------------------------------------------
AJ630457      CAACCTTTTTGGA-GGGGGATGCCTAAGGTAGGACTGGTGACTGGGGTGAAGTCGTAACAAGGTAGCCGTACCGGAAGGTGTGGCTGGATCACCTCCTTT
DQ234831      CAACCTTTT-GGA-GGGGGATGCCTAAGGTAGGACTGGTGACTGGGGTGAAGTCGTAACAAGGTAGCCGTACCGGAAGGTGTGGCTGGATCACCTCC---
AJ133161      CAACTTTTCGGAGAGGGGGATGCCTAAGGCAGTGCTGGTGACTGGGGTGAAATCGTAACAAGGTAGCCGTACCGGAAGG---------------------
gi|47118302   CAACTTTTAGGAGAGGAGGATGCCTAAGGCAGTGCTGGTGACTGGGGTGAAGTCGTAACAAGGTAGCCGTACCGGAAGGTGTGGCTGGATCACCTCCTTT
DQ185209      CAACCTTTCGGGGAGGAGGATGCCTAAGGCAGGACTGGTGACTGGGGTGAAGTC----------------------------------------------
AY742451      CAACCTTTCGGGGAGGAGGATGCCTAAGGCAGGACTGGTGACTGGGGTGAAGTCGTAACAAGGTAGCCGTACCGGAAGGTGTGGCTGGATACCTCCTT--
AF027655      CAACCTTTCGGGGAGGAGGATGCCTAAGGCAGGATTGGTGACTGGGGT----------------------------------------------------
DQ185211      CAACCTTTCGGGGAGGAGGATGCCTAAGGCAGGACTGGTGACTGGGGTGAAGTCGTAACAAG--------------------------------------
AY742449      CAACCTTTCGGGGAGGAGGATGCCTAAGGCAGGACTGGTGACTGGGGTGAAGTCGTAACAAGGTACCGTACCGGAAGGTGTGGCTTGGATACCCTTCCT-
EF174212      CAACCTTTCGGGGAGGAGGATGCCTAAGGCAGGACTGGTGACTGGGGTGAAGTCGTAACAAGGTAGCCGTACCGGAAGGTGTGGCTGGATCACCTCCTT-
DQ185213      CAACCTTTCGGGGAGGAGGATGCCTAAGGCAGGACTGGTGACTGGGGTGAAGTCGTAACAAGGTAGC---------------------------------
AF506237      TAACTGNNNNNNGAGGGGGACGCCTAAGGCNNNACTGGTG------------------------------------------------------------
AM230690      TAACTGCTTGCAGAGGGGGATGCCTAAGGCGAGACTGGTGACTGGGGTGAAGTCCTAACAAGGTAGCCGTACCGGAAGGTGTGGCTGGATCACCTCCTTT
AB039002      CAACTTTTCGGAGAGGGGGATGCCGAAGGCAGGTCTGGTGACTGGGGTGAAGTCGTAACAAGGTAGC-GTACCGGAAGG---------------------
AJ293131      CAACCTTTTGGAG-GGGG-ATGCCTAAGGTAGGACTGATGACTGGGGTGAAGTCGTAACAAGGTAGCCGTACCGGAAGGTGTGGCTGGATCACCTCCTTT
AJ133169      TAACCCGAAAGGGAGGAGGGTGCCGAAGGCGGGGTTGATGACTGGGGTGAAGTCGTAACAAGGTAGCCGTACCGGAAGG---------------------
DQ279769      TAACCGTTCGCGGAGGAGGATGCCGAAGGCAGGGCTGATGACTGGGGTGAAGTCGTAACAAGGTAGCCGTACCGGAAGGTGTGGCTGGATCACCTCCTTT
AJ000714      TAACCTTTCGAGGAGGAGGGTGCCGAAGGCAGGGYTGATGACTGGGGTGAAGTCGTAACAAGGTAGCCGTACCGGAAGGTGTGGC---------------
KVSF5         TAACCTTTCGAGGAGGGGGATGCCGAAGGCGAGACTGGTGACTGGGGTGAAGTCGTAACAAGGTAGCCGTACCGGAAGGTGTGGCTGGATCACCTCCTT-
```
